# Triggering and Preventing Cyclization of *o*‐Amino‐Guanidinobenzenes to 2‐Amino‐Benzimidazoles

**DOI:** 10.1002/chem.202503147

**Published:** 2025-12-12

**Authors:** Carmen L. M. Henel, Edwin Michel, Devin Zeitler, Olaf Hübner, Elisabeth Kaifer, Hans‐Jörg Himmel

**Affiliations:** ^1^ Ruprecht‐Karls‐Universität Heidelberg Germany

**Keywords:** amines, benzimidazoles, guanidines, isomerization, redox‐active ligands

## Abstract

Aromatic compounds in which a primary or secondary amino group is positioned next (in ortho position) to a guanidino group have been reported as intermediates in a variety of reactions, but are generally prone to cyclization to give 2‐amino‐imidazoles. Here, we present a comprehensive analysis, based on experiments and quantum‐chemical calculations, of the stability and reactivity of these compounds. It is shown that cyclization reactions are triggered by Brønsted and Lewis acids. The analysis discloses strategies allowing to prevent cyclization by careful choice of the guanidino group and/or substituent at the amino group. The results of this analysis allowed the synthesis of first unsymmetrical diguanidinobenzene molecules and coordination compounds with *o*‐amino‐guanidinobenzene ligands, paving the way for the development of aromatic compounds with adjacent amino and guanidino groups as a versatile class of redox‐active organic molecules.

## Graphical abstract

o‐Amino‐guanidinobenzenes are generally prone to cyclization to 2‐amino‐benzimidazoles. In this work, strategies to prevent such cyclizations are developed, allowing to use these compounds as ligands in coordination chemistry and for the design of novel (unsymmetrical) redox‐active ligands.

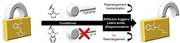

## 1 Introduction

**FIGURE 1:**
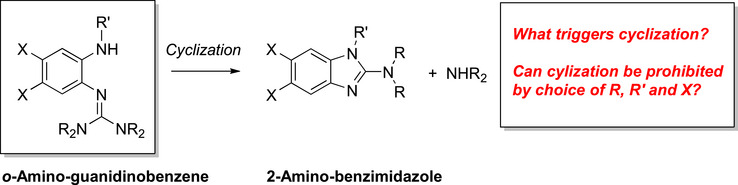
Cyclization reactions of o‐amino‐guanidinobenzenes to give 2‐amino‐benzimidazole derivatives, being the prime reason for their instability (R, R′ = organic groups, X = halide or other substituent).

This work is devoted to benzene derivatives exhibiting an amino group in ortho position to a guanidino group. Previous work showed that such molecules are prone to cyclization reactions leading to 2‐amino‐benzimidazole derivatives (Figure 1). For example, reaction of *o*‐diaminobenzene with 2‐chloro‐1,1,3,3‐tetramethylformamidinium chloride leads to 2‐dimethylamino‐benzimidazole, with *o*‐amino‐tetramethylguanidinobenzene (L1) as intermediate [1]. The reaction provides a convenient access to 2‐dimethylamino‐benzimidazole in a reported yield of 84%. The authors also showed that similar cyclization reactions are possible for *o*‐guanidino‐phenol and *o*‐guanidino‐thiophenol. Later the scope of this reaction was extended, leading to other 2‐dialkylaminobenzimidazole compounds [2]. Although 2‐amino‐benzimidazole and 2‐guanidino‐benzimidazole molecules are of interest as ligands in coordination chemistry and due to their biological activity that motivates a variety of applications in medicine [3], the cyclization prohibits the synthesis of stable o‐amino‐guanidinobenzenes. Recently, Herres‐Pawlis and coworkers reported the reduction of *o*‐tetramethylguanidino‐nitrobenzene to obtain *o*‐amino‐tetramethylguanidinobenzene, that was further converted by reaction with an aldehyde to a novel tridentate guanidino‐imino ligand [4]. In this case, the cyclization to a 2‐amino‐imidazole derivative was an unwanted side‐reaction. These results demonstrate the high interest in *o*‐amino‐guanidinobenzenes as stating reagents for the synthesis of multifunctional compounds.

Our group reported similar ring‐closing reactions during the synthesis of redox‐active aromatic compounds substituted with several guanidino groups, termed guanidino‐functionalized aromatics (GFAs). Since guanidines are conveniently synthesized from the corresponding amine precursors [5], the intermediate formation of aromatic compounds in which an amino group is positioned next to a guanidino group is often inevitable. The cyclization reactions that these intermediates undergo diminish the GFA yield [6, 7, 8].

The combination of amino and guanidino groups in ortho position to each other has several attractive features. Aromatic amino groups are important functional groups in organic chemistry. The lone‐pair at the amino group strongly interacts with the aromatic π‐system. The strong +M effect supports electrophilic aromatic substitutions. Moreover, compounds such as 1,4‐bis(dimethylamino)benzene, 1,2,4,5‐tetrakis(dimethylamino)benzene [9, 10, 11] and hexakis(dimethylamino)benzene [12] are versatile electron donors. Wurster's cation, the blue radical monocation that forms upon one‐electron oxidation of 1,4‐bis(dimethylamino)benzene, was already discovered in 1879 [13, 14]. On the other hand, these compounds are less interesting as redox‐active ligands in coordination chemistry. The strong +M effect causes the N atom to be generally bound in a trigonal‐planar rather than a pyramidal fashion, and leads to a significant attenuation of the Brønsted and Lewis basic character. Moreover, dimethylamino groups positioned ortho to each other experience a steric repulsion, that manifests itself in a twisted conformation of the C_6_ ring upon two‐electron oxidation of 1,2,4,5‐tetrakis(dimethylamino)benzene [11] and hexakis(dimethylamino)benzene [12]. Important redox‐active compounds suitable for coordination chemistry are obtained from aromatic compounds substituted by secondary amino groups. In their deprotonated versions, these compounds are widely applied as redox‐active ligands.

Guanidino‐functionalized aromatic compounds (GFAs) are even stronger electron donors than amino‐functionalized aromatics [15]. In recent years, a variety of 1,4‐diguanidino‐benzene and 1,2,4,5‐tetraguanidino‐benzene molecules, as well as a hexaguanidino‐benzene were reported [8]. With respect to amino‐functionalized aromatic compounds, GFAs distinguish themselves by several attractive features. i) The nitrogen atom directly attached to the C_6_ ring is connected to only two atoms, and the central N_3_C plane of the guanidino groups is highly tilted with respect to the C_6_ ring plane. Consequently, there are no steric constraints; neutral and dicationic redox states exhibit a flat C_6_ ring. ii) Due to charge delocalization, the dicationic, oxidized redox states are generally stable and can be isolated. iii) A guanidino group bound to an aromatic core still is a strong Lewis base. Consequently, GFAs bind strongly to metals, even in their oxidized, cationic redox state [16, 17], in difference to dialkylamino‐substituted aromatics. iv) Guanidino groups attached to aromatic cores still have a very high Brønsted basicity, being a key feature for their use in proton‐coupled electron‐transfer (PCET) reactions [15] and redox catalysis [18]. By contrast, amino groups attached to aromatic cores are generally much weaker Brønsted bases than alkyl amines.

Herein, a systematic analysis of the stability of *o*‐amino‐guanidinobenzene molecules and their reactivity toward Brønsted and metal Lewis acids, that will be shown to trigger cyclization, is carried out. The guanidino and amino groups are varied to track systems that are stable with respect to (Brønsted‐ or Lewis‐acid induced) cyclization reactions. Then, the new *o*‐amino‐guanidinobenzenes are used for the synthesis of unsymmetrical diguanidinobenzenes, and as ligands in coordination chemistry.

## 2 Results and Discussion

### 2.1 Synthesis and Reactivity of Benzene Derivatives With an Amino (NH_2_) Group in Ortho Position to a Guanidino Group

**FIGURE 2:**
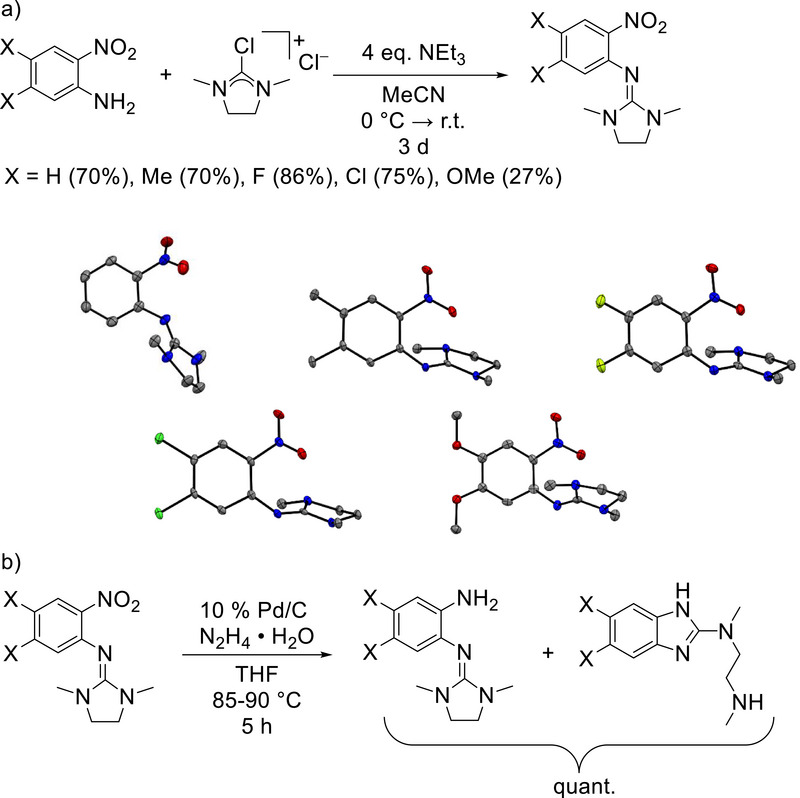
(a) Reaction to give the 1‐nitro‐2‐guanidino intermediates, and illustrations of the structures of the molecules with X = H, Me, F, Cl, and OMe. Displacement ellipsoids shown at the 50% probability level. Color code: C dark‐grey, N blue, O red, Cl green, F pale green. (b) Reduction of the nitro group to give quantitatively a mixture of *o*‐amino‐guanidinobenzene molecules with primary amino groups and their cyclization products.

We started our analysis with 2‐amino‐1‐nitrobenzene and four derivatives substituted in 4 and 5 positions with electron‐withdrawing (X = F or Cl) or electron‐donating (X = Me or OMe) substituents. Reaction with 2‐chloro‐1,3‐dimethyl‐4,5‐dihydro‐1*H*‐imidazolium chloride gave the 1‐nitro‐2‐*N*,*N*′‐dimethyl(ethylene)guanidinobenzene molecules (Figure 2a). The analytical data for the compound with X = H are similar to those reported recently by Herres‐Pawlis and coworkers [4]. All these five 1‐nitro‐2‐guanidinobenzene molecules were crystallized, either by partial solvent (Et_2_O) removal under vacuum or by solvent (acetone) evaporation. In Figure 2a, the structures of the compounds with X = H, Me, F, Cl and OMe are shown. The imino N = C bond lengths measure 1.297(4) Å (X = H), 1.295(1) Å (X = Me), 1.301(2) Å (X = F), 1.306(2) Å (X = Cl), and 1.294(8) Å (X = OMe), falling in a typical region for such C = N double bonds [19]. In all molecules, the CN_3_ plane of the guanidino group is tilted with respect to the benzene ring plane (57° for X = H, 69.6° for X = Me, 66.5° for X = F, 68.8° X = Cl, 67.4° X = OMe). This conformation is generally favored in guanidino‐substituted aromatics [19, 20]. The NO_2_ plane is tilted by 41° (X = H), 19.6° (X = Me), 22.78° (X = F), 18.9° (X = Cl), 10.49° (X = OMe) from the benzene ring plane.

Then, the nitro group was reduced with hydrazine and a sub‐stoichiometric (10%) amount of Pd/C in THF solution. For comparability, all reactions were carried out at 85–90°C and with a reaction time of 5 hours. The reaction time of 5 hours was chosen to ensure complete reduction, especially with R = F and R = Cl. The THF was removed in vacuum and the product re‐dissolved in CDCl_3_ for NMR analysis. The reactions led in quantitative yield to mixtures of the *o*‐amino‐guanidinobenzene and the isomeric 2‐amino‐benzimidazole cyclization product (Figure 2b). Using ^1^H NMR spectroscopy, the ratio of the two products was estimated (see Table 1 and Supporting Information for details). Interestingly, the ratio can be significantly varied by choice of the substituents X at the aromatic backbone. With X = H, the ratio is approximately 4:6. With X = F, the *o*‐amino‐guanidinobenzene product is favored (83%–85%), By contrast, the 2‐amino‐imidazole product is favored for X = Me. There is no clear correlation between the product ratio and the properties of the substituents X, but electron‐withdrawing substituents seem to favour the formation of *o*‐amino‐guanidinobenzene. These substituents are likely to increase the rate for reduction of the nitro group to the amino group, and thereby mitigate side reactions.

**TABLE 1:** Ratios of the *o*‐amino‐guanidinobenzene and 2‐amino‐benzimidazole products in dependence of the substituents X for the reaction in Figure 2b, estimated from ^1^H NMR spectroscopy (CDCl_3_). The Δ*G*_calc_ value (in kJ mol^−1^) from B3LYP+D3/def2‐TZVP calculations for the isomerization of the *o*‐amino‐guanidinobenzene to the 2‐amino‐benzimidazole is also included. A negative value implies that the 2‐amino‐benzimidazole is energetically preferred.

X	*o*‐amino‐guanidinobenzene 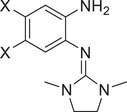	2‐amino‐benzimidazole 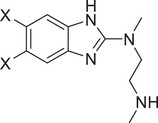	Δ*G*_calc_
H	approx. 43%	approx. 57%	−5.7
Me	approx. 15–20%	approx. 80–85%	−6.9
F	approx. 83–85%	approx. 15–17%	−13.7
Cl	approx. 62–85%	approx. 15–38%	−5.7
OMe	approx. 38–41%	approx.59%–62%	−8.6

Moreover, the product ratio does not change significantly with temperature. Interestingly, when a 4:6 product mixture with X = H was dissolved in water, the NMR spectra proved instant full conversion to the 2‐amino‐benzimidazole (see Supporting Information). When the water was removed under vacuum and the compound re‐dissolved in chloroform, the signals of the *o*‐amino‐guanidinobenzene did not reappear. By contrast, the ratio of *o*‐amino‐guanidinobenzene and 2‐amino‐benzimidazole in CDCl_3_ did not change upon addition of a small amount of D_2_O. Also, no changes were observed when the mixture was dissolved in benzene, DMSO or MeOH. In the following, it will be shown that cyclization of *o*‐amino‐guanidinobenzene to give 2‐amino‐benzimidazole is triggered by Brønsted and Lewis acids.

### 2.2 Quantum‐chemical Calculations

Next, quantum‐chemical calculations were carried out to obtain additional information about the relative stabilities of the two isomers and the mechanism of their interconversion. The calculated Δ*G*_calc_ values for isomerization are collected in Table 1. In these reactions, an N‐H and a C‐H bond are cleaved, and new N‐H and C‐H bonds are formed. The Δ*G*_calc_ values are all negative and relatively small. In all cases the 2‐amino‐benzimidazole is slightly more stable than the *o*‐amino‐guanidinobenzene isomer. However, there is no obvious correlation between the Δ*G*_calc_ values and the properties of the substituents X, in line with the experimental results. Obviously, the small effect that the substituents have on the relative Gibbs free energy of the two products does not play a decisive role for the ratio they are formed in the reaction sketched in Figure 2b. Moreover, the experiments show that the two isomers are not in a thermal equilibrium.

**SCHEME 1:**
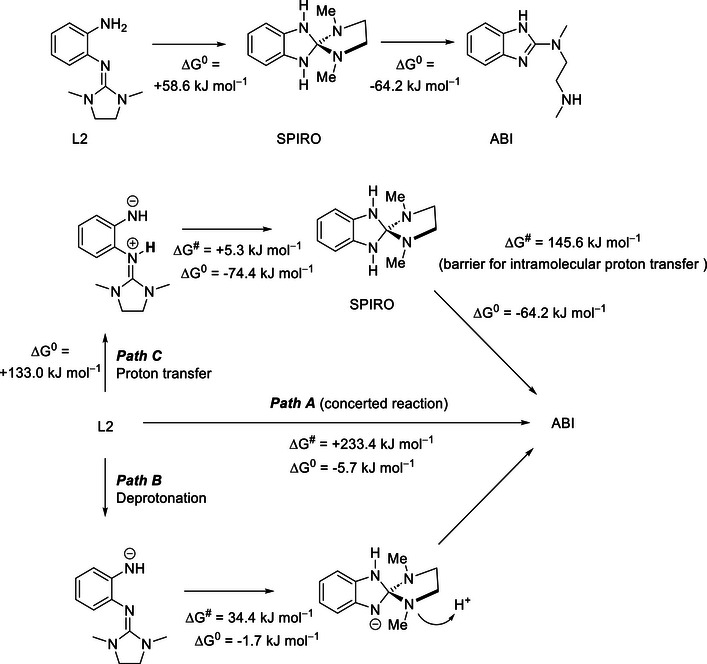
Cyclization of L2 to give ABI with the intermediate formation of a spiro compound. The three possible pathways are sketched, together with the calculated Δ*G* values (at 298 K, 1 bar) from B3LYP+D3/def2‐TZVP calculations and the reaction barriers. Deprotonation and reprotonation as well as proton transfer are likely to occur intermolecularly.

Subsequently, the question is addressed if the cyclization is a one‐step process or involves intermediates, and if it is catalyzed by an acid or a base. These calculations focused on the cyclization of L2 to the 2‐amino‐benzimidazole (abbreviated ABI, Scheme 1). For the isomerization L2 → ABI, the calculations (with the def2‐TZVP basis set) yield a value for the electronic energy of −10.1 kJ mol^−1^ and values for the enthalpy at 0 K and the Gibbs free energy at 298 K of −6.9 and −5.7 kJ mol^−1^, respectively.

To determine the transition structures, calculations with the method of the nudged elastic band [21] (NEB) were performed, also combined with the procedure of the climbing image [22] (NEB‐CI), in which the highest‐lying structure of the path is moved uphill along the tangent to the path. Then the transition structure was optimized (NEB‐TS) [23]. For a concerted course of the isomerization with simultaneous formation of the C−N bond and proton transfer from the NH_2_ group to one of the amino nitrogen atoms of the guanidino group (path A in Scheme 1), the NEB‐TS calculations yield an electronic energy of 234.6 kJ mol^−1^ and values for the enthalpy at 0 K and the Gibbs free energy at 298 K of 228.8 and 233.4 kJ mol^−1^, respectively, for the transition state with respect to the reactant. These values indicate a relatively high barrier, being in line with the observation that cyclization cannot be initiated by a temperature change. Thus, a concerted course of the reaction is unlikely, and it is much more likely that the reaction proceeds in an acid or base catalyzed way. Therefore, also protonated and deprotonated forms have been studied.

For a protonated form of L2, with the additional proton being located at the imine nitrogen of the guanidino group (the most basic position), a scan of the potential energy surface has been performed. The distance between the carbon atom of the guanidino group and the nitrogen atom of the NH_2_ group has been successively decreased, down to a distance in the range of C−N bonds, whereas all other structure parameters have been optimised. The scan yielded but a continuous increase of the energy.

For the deprotonated form of L2 (path B, Scheme 1), in which one of the protons of the NH_2_ group has been removed, a spiro isomer C_6_H_4_N_2_HC(NCH_3_)_2_(C_2_H_4_)^−^ has been found. Its electronic energy with respect to the deprotonated form of L2 amounts to −5.3 kJ mol^−1^ and the corresponding values for the enthalpy at 0 K and the Gibbs free energy at 298 K to −5.0 and −1.7 kJ mol^−1^, respectively. The electronic energy of the barrier (NEB‐TS calculations) for the formation of this intermediate amounts to only 36.9 kJ mol^−1^, with values for the enthalpy at 0 K and the Gibbs free energy at 298 K of 32.3 and 34.4 kJ mol^−1^, respectively. Then, the protonation of this deprotonated species at the amino nitrogen atoms with the CH_3_ groups directly leads to the formation of the ABI product. The required proton might be abstracted from a second *o*‐amino‐guanidinobenzene molecule.

Calculations also have been performed for an isomer of L2 in which one of the protons of the NH_2_ group has been removed and instead the imino nitrogen atom protonated (path C, Scheme 1). The electronic energy of this tautomer with respect to L2 amounts to 130.8 kJ mol^−1^ and the enthalpy at 0 K and the Gibbs free energy at 298 K to 131.0 and 133.0 kJ mol^−1^, respectively. Starting from this tautomer, the electronic energy of the barrier for the formation of the C−N bond and thus the formation of a spiro isomer amounts to only 7.7 kJ mol^−1^ and the corresponding enthalpy at 0 K and Gibbs free energy at 298 K to 4.0 and 5.3 kJ mol^−1^, respectively. The spiro isomer has an electronic energy with respect to the tautomer of L2 of −77.1 kJ mol^−1^ and the values for the enthalpy at 0 K and the Gibbs free energy at 298 K amount to −76.8 and −74.4 kJ mol^−1^, respectively. For the final formation of ABI, it is necessary to transfer one of the protons of the amino nitrogen atoms from the central ring of the spiro isomer to the amino nitrogen atoms with the CH_3_ groups. The electronic energy for the formation of ABI from the spiro isomer amounts to −63.8 kJ mol^−1^ and the enthalpy at 0 K and the Gibbs free energy at 298 K to −61.1 and −64.2 kJ mol^−1^, respectively. For an intramolecular course of this reaction, the electronic energy of the barrier of 154.7 kJ mol^−1^ is relatively high and the enthalpy at 0 K and the Gibbs free energy at 298 K were calculated to be 143.8 and 145.6 kJ mol^−1^, respectively. However, a base catalysed course of this reaction, which is initialized by a proton transfer, is likely to decrease the barrier.

### 2.3 Applications for the Synthesis of Unsymmetrical *o*‐diguanidinobenzenes

**FIGURE 3:**
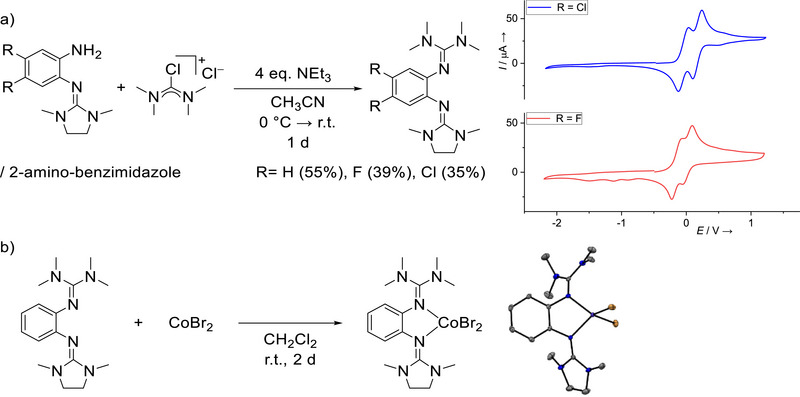
(a) Synthesis of unsymmetrical *o*‐diguanidinobenzenes and cyclovoltammogram of the fluoro and chloro bisguanidine ligands in CH_2_Cl_2_, (b) Synthesis of a cobalt complex with one of the new unsymmetrical *o*‐diguanidinobenzene ligands. An illustration of the solid‐state structure is shown on the right side. Displacement ellipsoids drawn at the 50% probability level. Color code: C dark‐grey, N blue, Br brown, Co purple.

Further experiments showed that the *o*‐amino‐guanidinobenzene derivatives are valuable starting compounds for the synthesis of *o*‐diguanidinobenzene derivatives with two different guanidino groups. Here, we synthesized three new *o*‐diguanidino‐benzene derivatives with an *N*,*N*′‐dimethyl(ethylene)guanidino and a tetramethylguanidino group (Figure 3a) and two substituents X in the backbone (X = H, F or Cl). In these experiments, we used the mixtures containing both the *o*‐amino‐guanidinobenzene and its isomer, the 2‐amino‐benzimidazole. For this reason, the yield was not very high. Please note that CH_3_CN was used as solvent in these experiments; therefore the ratios of the two isomers are somewhat different to those given in Table 1 for CDCl_3_ solutions. The residues R = H, F and Cl were chosen due to their high proportion of primary amine of up to 85% (for R = F). Due to the low proportion of less than 40% of primary amine and the resulting low yield for the diguanidine ligand, no conversion to unsymmetrical diguanidine was carried out for the compounds with R = Me or OMe.

The diguanidine ligand with R = H shows no reversible redox process in the cyclic voltammetry curve, but two oxidation events at ca. *E*_ox_ = ‐0.05 V and 0.14 V. On the other hand, two reversible redox events can be observed in the CV curves recorded for the molecules with X = F and Cl (Table 2 and Figure 3, as well as Supporting Information). These are assigned to reversible oxidation to mono‐ and dicationic compounds. Recently, our group reported a symmetrical ligand with X = Cl and two related guanidino groups (1,3‐dimethylimidazolidin‐2‐imino), exhibiting similar potentials of *E*_1/2_(1) = ‐0.08 V and *E*_1/2_(2) = 0.16 V [24]. In all cases, the first one‐electron redox event is slightly negative, and the second slightly positive with respect to the ferrocenium/ferrocene reference redox couple.

**TABLE 2:** Potentials (*E*_1/2_ and *E*_ox_ values, both in V relative to the reference redox couple ferrocenium/ferrocene) from cyclic voltammetry (CV) measurements in CH_2_Cl_2_ solutions.

Compound	*E*_1/2_(1)	*E*_ox_(1)	*E*_1/2_(2)	*E*_ox_(2)
X = F	−0.15	−0.07	0.03	0.10
X = Cl	−0.05	0.03	0.17	0.24

Subsequently, we used the unsymmetrical *o*‐diguanidinobenzene derivative with X = H for the synthesis of a first coordination compound. Such compounds with an unsymmetrical redox‐active ligand are of interest due to the additional possibilities they offer with respect to the fine tuning of the electronic and steric properties. The diguanidine was stirred for a period of 2 days at room temperature together with one equivalent of CoBr_2_ in CH_2_Cl_2_ solution to give the blue cobalt complex in quantitative yield (Figure 3b). Crystals were grown by layering a CH_2_Cl_2_ solution with *n*‐hexane and structurally characterized (Figure 3b). As expected, the Co^II^ atom is tetrahedrally coordinated, and the Co‐N and Co‐Br bond lengths of 2.014(2)/2.008(2) Å and 2.410(1)/2.389(1) Å are in typical regions for high‐spin Co^II^ atoms [19]. The imino N = C bond lengths measure 1.335(3) Å for the tetramethylguanidino group and 1.325(3) Å for the 1,3‐dimethylimidazolidin‐2‐imino group. Generally, due to π‐donor bond contributions to the metal‐nitrogen bond [25], the imino C = N bond is elongated, and guanidines evoke a weak ligand field. In the UV‐Vis spectrum, recorded in CH_2_Cl_2_ solution, weak and broad absorptions show at 571, 632 and 675 nm, that are responsible for the blue color and tentatively assigned to d‐d transitions.

In the CV curve of the cobalt complex (in CH_2_Cl_2_ solution, see Supporting Information), a reversible redox process at *E*_1/2_ = 0.21 V (*E*_ox_ = 0.26 V) appears, that is assigned to a one‐electron ligand oxidation with a potential moderately shifted to higher values with respect to the free ligand. A second, broader oxidation wave is visible near *E*_ox_ = 0.66 V and might belong to a metal‐centred redox event. The results demonstrate the possibility to integrate the radical‐monocationic redox state of the new unsymmetrical redox‐active diguanidine ligands in transition‐metal complexes. The approach used in this work is very different to the previously reported redox‐induced aromatic substitution pathway, that was used for the construction of unsymmetric tetraguanidines [26].

### 2.4 Synthesis of Benzene Derivatives With a Secondary Amino Group (NHPh or NHMe) in Ortho Position to a Guanidino Group

**FIGURE 4:**
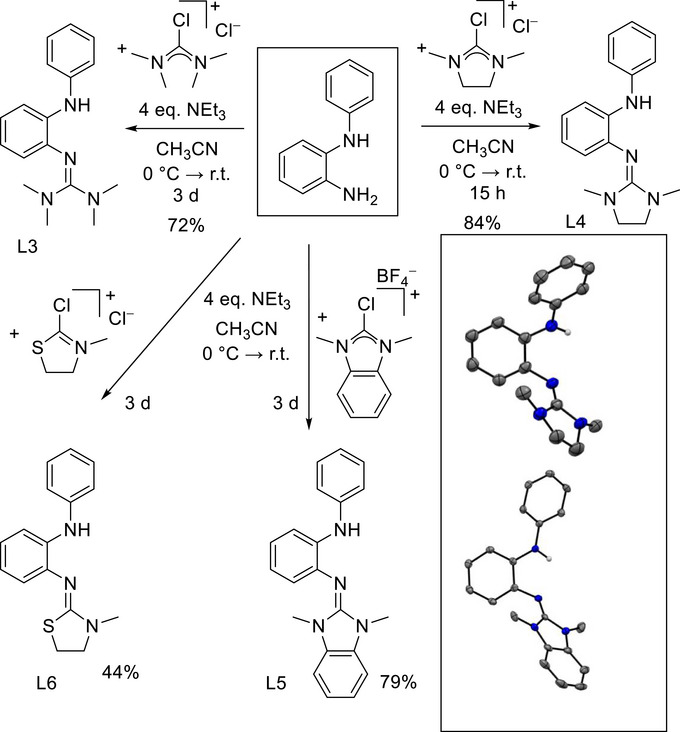
Synthesis of the new *o*‐guanidino‐phenylaminobenzene molecules L3‐L6. Illustrations of the structures of L4 and L5 in the solid state are included. Displacement ellipsoids drawn at the 50% probability level. C‐H hydrogen atoms omitted. Color code: C dark‐grey, N blue, H light‐grey.

Next, we studied the possibility to synthesize compounds with phenylamino‐ and methylamino groups in ortho position to a guanidino group. Such compounds are particularly interesting as ligands in coordination chemistry. Indeed, compounds with a secondary phenylamino group were directly synthesized starting with commercially available 1‐phenylamino‐2‐amino‐benzene (Figure 4). In our experiments, we varied the guanidino/thioguanidino group, thereby obtaining four different molecules (L3 – L6, Figure 4) in yields between 44% and 84%. In the case of L3, the product was only obtained together with the corresponding 2‐amino‐benzimidazole cyclization product. In all other cases, the ligands were isolated in pure form in 84% yield for L4, 79% for L5 and 44% for L6. Compound L4 crystallized from a CH_2_Cl_2_ solution at a temperature of ‐31°C, and crystals of L5 were obtained by layering a concentrated CH_2_Cl_2_ solution with *n*‐hexane at ‐31°C (Figure 4). The imino N═C bond lengths measure 1.295(4) Å for L4 and 1.288(2) Å for L5. In all molecules, the CN_3_ plane of the guanidino group is tilted with respect to the benzene ring plane (dihedral angle of 77° for L4 and 61.2° for L5). The NH‐Ph plane is tilted 46° for L4 and 43° for L5 with respect to the benzene plane. The packing in the unit cell of L4 (seems to be determined by intermolecular NH···N hydrogen bonds measuring 2.296 Å. There are 16 molecules in the unit cell. Eight molecules in the center of the unit cell form hydrogen‐bonded dimers, while the remaining eight are distributed along the axes with hydrogen bridges to molecules in the adjacent unit cells. This implies that all molecules form hydrogen‐bonded dimers. In contrast, L5 is the only secondary amine that does not establish hydrogen bonds to adjacent molecules. The packing in solid L5 is determined by π–π interactions of the benzimidazole units, which exhibit slipped facial arrangement. The intermolecular distance between the π‐systems amounts to 3.451 Å, falling within a typical range for π–π interactions [27, 28]. A strong band in the IR spectrum at 3242 cm^−1^ for L4, 3589 cm^−1^ for L5, and 3358 cm^−1^ for L6 is assigned to the stretching mode, ν(N‐H). The relatively low wavenumbers for L4 and L6 in comparison with L5 is in line with the hydrogen‐bonding in these compounds in the solid state.

**FIGURE 5:**
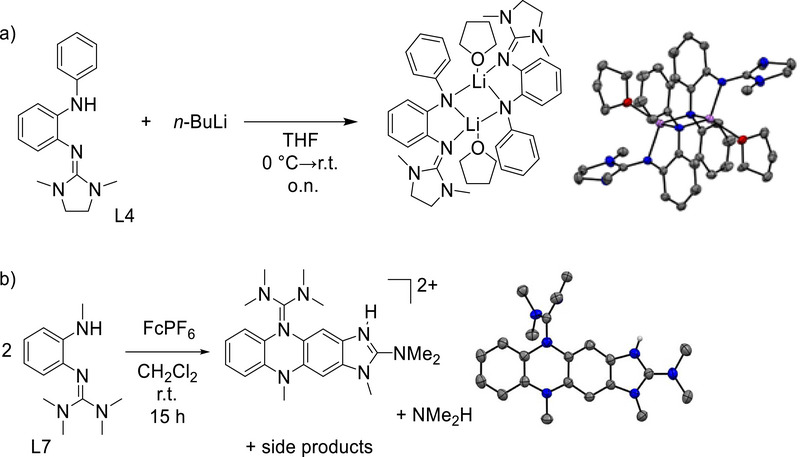
(a) Reactions leading to the dimer of the lithiated compound, and illustration of its structure in the solid state. (b) Dimerization of an *o*‐amino‐guanidinobenzene derivative upon oxidation, together with an illustration of the obtained product structure in the solid state. Solvent molecules and PF_6_^−^ ions are omitted. For both structures, displacement ellipsoids correspond to a 50% probability of occurrence. C‐H hydrogen atoms omitted. Color coding: C grey, N blue, O red, H light‐grey, Li violet.

Molecule L4 is stable toward Brønsted acids and bases. In a preliminary test experiment, we deprotonated L4 with *n*‐BuLi in THF. The product was isolated as a dimer with a four‐membered Li_2_N_2_ ring that connects two deprotonated ligands. Red crystals precipitated from the reaction mixture, suitable for structural characterization (Figure 5). Both lithium atoms are four‐coordinate due to the coordination of a THF molecule to each lithium. The structure is similar to lithiated aniline, that also displays Li_2_N_2_ four‐membered rings [29]. The complete characterization of this compound was hampered by its high sensitivity and reactivity. Therefore, we abstained from further analysis. The results show that deprotonation is possible; cyclization to 2‐amino‐benzimidazole derivatives was not observed. In future work, the compound can be deprotonated in situ, followed by a transmetallation step to give stable complexes of the deprotonated ligand.

**FIGURE 6:**
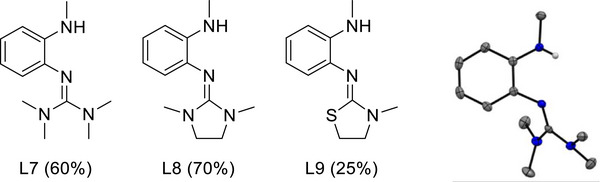
*o*‐Guanidino‐methylaminobenzene compounds that were synthesized in this work. The structure of L7 in the solid state is illustrated on the right side.

The three compounds L7 – L9 with methylamino groups were synthesized in a similar way as the compounds with phenylamino group (Figure 6 and Supporting Information). Compound L7 crystallized and was structurally characterized. The imino N═C bond length measures 1.297(2) Å. The CN_3_ plane of the guanidino group is tilted by 65.54° with respect to the benzene ring plane. Intermolecular NH···N hydrogen bonds (2.217 Å and 2.232 Å) determine the packing in the unit cell, leading to hydrogen‐bonded dimers. A strong band in the IR spectrum at 3269 cm^−1^ is assigned to the stretching mode ν(N‐H), and the relatively low wavenumber again is in line with the hydrogen‐bond motif.

Cyclic voltammetry measurements show that the compounds can be oxidized at relatively low potentials. However, the oxidation processes are not reversible. L4 and L5 show two strong oxidation waves in CH_2_Cl_2_, at *E*_ox_ = 0.03 V and 0.77 V for L4 and at *E*_ox_ = 0.02 V and 0.69 V for L5 (all potentials given vs ferrocenium/ferrocene). The CV curve of L6 contains oxidation waves at 0.27 and 0.75 V. In the CV curve of L7, the first oxidation is observed at ‐0.02 V and a second at 0.80 V. The irreversibility of the redox event is due to dimerization. L9 shows two oxidation waves at *E*_ox_ = 0.03 V and 0.68 V. Cyclic voltammetry measurements were not carried out for L2, L3, and L8, due to the presence of the 2‐amino‐benzimidazole cyclization product, that cannot be avoided.

The instability of the radical monocation arises from the unprotected aromatic backbone. After one‐electron oxidation to the radical monocation, the compounds dimerize (Figure 5b). It was not possible to isolate the oxidation product in pure form, but crystals suitable for a structural characterization were obtained upon oxidation of L7 with ferrocenium. We previously reported the synthesis of a dimerization product in pure form upon oxidation of an *o*‐diguanidinobenzene [26], in which the benzene rings were bridged by N atoms from two guanidino groups. Generally, ferrocenium seems to trigger cyclization like typical Lewis acids (see discussion in the next section). From reaction mixtures containing L3 or L8 together with FcPF_6_, only the rearranged and protonated benzimidazole derivatives with PF_6_^−^ counterion were crystallized. Only in the case of L4, ferrocenium does not initiate cyclization to a 2‐amino‐benzimidazole.

### 2.5 Brønsted‐ and Lewis‐acid Induced Cyclization

**FIGURE 7:**
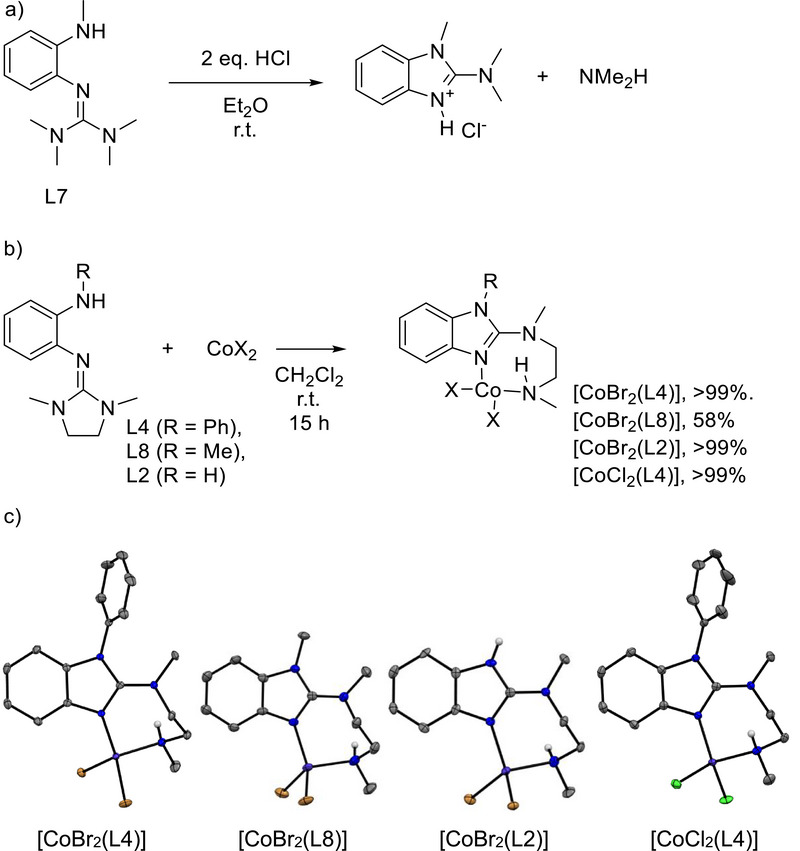
(a) Cyclization of L7 triggered by HCl. (b) Reaction of CoBr_2_ / CoCl_2_ with *o*‐*N*,*N*′‐dimethyl(ethylene)guanidinoaminobenzene derivatives. (c) Illustrations of the solid‐state structures of the four cobalt complexes. Displacement ellipsoids drawn at the 50% probability level. C‐H hydrogen atoms omitted. Color code: C dark‐grey, N blue, H light‐grey, Br brown, Cl green, Co violet.

In this section, we show that cyclization of the *o*‐amino‐guanidinobenzene to the 2‐amino‐benzimidazole isomer is generally triggered by Brønsted and Lewis acids. In a first experiment, we added two equivalents of HCl·Et_2_O to a solution of L7 in Et_2_O (Figure 7a). The NMR spectra showed immediate and quantitative formation of the 2‐amino‐benzimidazole derivative. Addition of HCl·Et_2_O to a solution of L8 also initiated quantitative rearrangement to the 2‐amino‐benzimidazole, as evidenced by NMR spectroscopy. Interestingly, all compounds with phenylamino groups show a different behavior. Precipitation of a mixture of both forms is observed for L3. In the case of L4 or L5, the spectra only show formation of the protonated *o*‐amino‐guanidinobenzene molecules.

Next, we reacted L2 and L4‐L9 with a variety of Lewis acidic metal complexes. Herein, we focus on the reactions with cobalt dihalides (CoCl_2_ and CoBr_2_) and zinc halides (ZnCl_2_), since these reactions yielded pure, fully characterized products. As shown in Figure 7b, coordination is accompanied by isomerization in the case of L2, L4 and L8. Crystals of the complexes [CoBr_2_(L4)], [CoBr_2_(L8)] and [CoCl_2_(L4)] were grown from CH_2_Cl_2_ solutions layered with *n*‐hexane (Figure 7c), whereas crystals of [CoBr_2_(L2)] were grown by cooling an acetonitrile suspension to −31°C. The 2‐amino‐benzimidazole binds to the Co atom with the imino N atom of the benzimidazole ring and with the terminal secondary amino group, leading to a seven‐membered CoN_3_C_3_ ring. The N═C bond length in the five‐membered imidazole ring measures 1.347(2) Å for [CoBr_2_(L4)], 1.341(2) Å for [CoCl_2_(L4)], 1.349(4) Å for [CoBr_2_(L8)] and 1.340(5) Å for [CoBr_2_(L2)], while the bond distance between cobalt and nitrogen of the five‐membered ring amounts to 2.010(2) Å for both [CoBr_2_(L4)] and [CoCl_2_(L4)], 1.999(2) Å for [CoBr_2_(L8)], and 1.986(4) Å for [CoBr_2_(L2)]. The bond lengths are similar to those in previously reported tetrahedrally coordinated high‐spin Co^II^ complexes with benzimidazole units [30]. The Co‐NH bond length are only slightly larger (2.055(2) Å for [CoBr_2_(L4)], 2.057(2) Å for [CoCl_2_(L4)], 2.051(3) Å for [CoBr_2_(L8)] and 2.061(4) Å for [CoBr_2_(L2)]). The Co‐X (X = Cl, Br) bond lengths also fit to high‐spin Co^II^ halide complexes [31]. A paramagnetic ^1^H NMR spectrum was measured for each of the compounds [CoBr_2_(L4)] and [CoBr_2_(L8)] (see Supporting Information).

**FIGURE 8:**
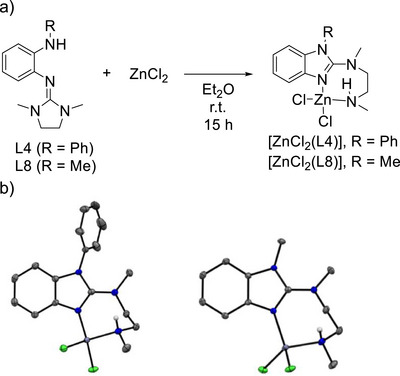
(a) Reaction between L4/L8 and ZnCl_2_. (b) Illustration of the structures of [ZnCl_2_(L4)] and [ZnCl_2_(L8)] in the solid state. Displacement ellipsoids drawn at the 50% probability level. C‐H hydrogens omitted. Color code: C dark‐grey, N blue, H light‐grey, Zn violet, Cl green.

Obviously, this type of reactivity (cyclization followed by coordination) is not restricted to Co^II^ compounds, but is generally observed in the presence of a Lewis acidic metal complex that triggers cyclization. Hence, reactions between L4 or L8 and ZnCl_2_ led to zinc complexes of the 2‐amino‐benzimidazole cyclization products (Figure 8a).

The complexes crystallized from acetone solution upon partial solvent removal (Figure 8b). The N═C bond lengths in the five‐membered benzimidazole ring measure 1.339(3) Å for [ZnCl_2_(L4)] and 1.3434(13) Å for [ZnCl_2_(L8)]. The Zn‐N bond lengths involving the nitrogen in the five‐membered ring amount to 2.0229(16) Å for [ZnCl_2_(L4)] and 2.0160(9) Å for [ZnCl_2_(L8)], being slightly longer than the Zn‐N bond lengths to the imidazole unit in 2‐guanidino‐benzimidazole zinc complexes [32]. The Zn‐NH bond length measures 2.0633(17) Å for [ZnCl_2_(L4)] and 2.0657(10) Å for [ZnCl_2_(L8)]. The packing in the unit cell is again determined by NH···Cl hydrogen bonds with a bond length of 2.501 Å for [ZnCl_2_(L4)] and 2.457 Å for [ZnCl_2_(L8)]. A strong band in the IR spectrum at 3196 cm^−1^ (R = Ph) and 3187.36 cm^−1^ (R = Me) is assigned to the stretching mode ν(N‐H), and the relatively low wavenumbers are in line with hydrogen bonding. In summary, the results in this section show that isomerization is generally triggered by a Lewis acid. The resulting 2‐amino‐benzimidazole molecules act as bidentate ligands.

Quantum‐chemical (DFT) calculations (B3LYP functional with D3 correction, in combination with the def2‐TZVP basis set) indicate that cyclization is endothermic (positive Δ*H* value), but exergonic (negative Δ*G* value) for L1 and L7, due to the duplication of the number of molecules (release of dimethylamine). In the case of L3 with a phenylamino group, the cyclization is mildly exothermic and also exergonic. On the other hand, it is significantly endothermic and endergonic for L5, also exhibiting a phenylamino group. This explains the observation that L5 is stable toward Lewis acids.

The Δ*G* values for cyclization to the 2‐amino‐benzimidazole molecules are all clearly negative for *o*‐amino‐guanidinobenzene molecules with a tetramethylguanidino group. The positive entropy change is the reason for the energetically favored cyclization. In the case of compounds with *N,N*‘‐dimethyl(ethylene)guanidino group, the Δ*G* values are either slightly negative or slightly positive. Finally, for the *o*‐guanidino‐amino‐benzene molecules with 1,3‐dimethylimidazolidin‐2‐imino group, the Δ*G* values are clearly positive. These results show that the tendency to cyclization could be controlled by the choice of the guanidino group.

### 2.6 Stable *o*‐amino‐guanidinobenzene Molecules

**FIGURE 9:**
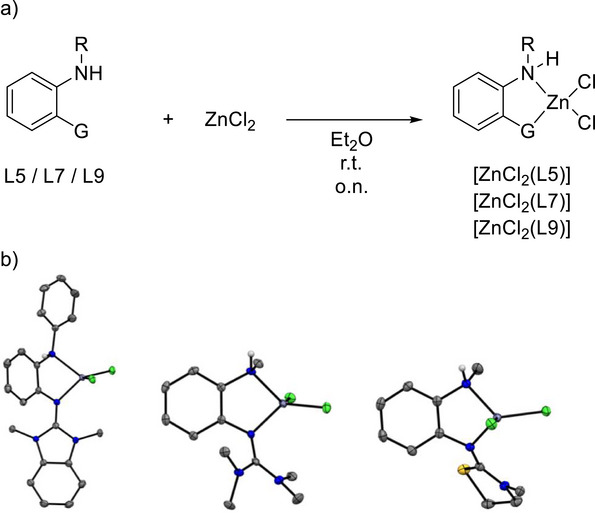
(a) Synthesis of the complexes [ZnCl_2_(L5)], [ZnCl_2_(L7)] and [ZnCl_2_(L9)]. (b) Illustrations of the structures in the solid state. Displacement ellipsoids drawn at the 50% probability level. C‐H hydrogens omitted. Color code: C dark‐grey, N blue, S yellow, Zn violet, Cl green.

Interestingly, L5, L7, and L9 are stable toward cyclization to the 2‐amino‐benzimidazole products upon addition of a metal Lewis acid. Please note that L10 was not obtained in pure form and therefore not considered in our experimental work. In the case of L5, cyclization is thermodynamically disfavored (Table 3). For L7 and L9, the Δ*G* values indicate a weak preference for the 2‐amino‐benzimidazole (Table 3); therefore the *o*‐amino‐guanidino form might be kinetically stabilized. Hence, it was possible to synthesize the zinc complexes [ZnCl_2_(L5)], [ZnCl_2_(L7)] and [ZnCl_2_(L9)] with intact *o*‐amino‐guanidinobenzene ligands (Figure 9a). Crystals of [ZnCl_2_(L5)] were grown by layering a concentrated dichloromethane solution with *n*‐hexane. The complexes [ZnCl_2_(L7)] and [ZnCl_2_(L9)] crystallized from acetone solutions upon partial solvent removal (Figure 9b). The imino N═C bond lengths measure 1.327(3) Å for [ZnCl_2_(L5)], 1.324(2) Å for [ZnCl_2_(L7)], and 1.309(2) Å for [ZnCl_2_(L9)]. Furthermore, the Zn‐N bond length involving the imino nitrogen of the guanidino group measures 2.026(2) Å for [ZnCl_2_(L5)], 2.021(2) Å for [ZnCl_2_(L7)], and 2.055(1) Å for [ZnCl_2_(L9)]. The other Zn‐N bond length to the secondary amine amounts to 2.104(2) Å for [ZnCl_2_(L5)], 2.095(2) Å for [ZnCl_2_(L7)], and 2.077(2) Å for [ZnCl_2_(L9)]. In the solid state, intermolecular N‐H^…^Cl‐Zn hydrogen bonds determine the packing motif. These hydrogen bonds measure 2.532 Å for [ZnCl_2_(L5)], 2.562 Å for [ZnCl_2_(L7)], and 2.454 Å for [ZnCl_2_(L9)]. The unit cell of [ZnCl_2_(L5)] contains a hydrogen‐bonded dimer. The hydrogen bond lengths are comparable with the values for short (2.52 Å) to intermediate (2.52‐2.95 Å) N‐H^…^Cl‐M (M = transition metal) bonds reported in the literature [33]. The IR spectrum of [ZnCl_2_(L5)] contains a sharp band at 3131 cm^−1^ due to the stretching mode ν(N‐H), while [ZnCl_2_(L7)] shows a sharp band at 3243.64 cm^−1^. These relatively low wavenumbers are in line with the presence of intermolecular hydrogen bonding.

**TABLE 3:** Calculated changes in the Gibbs free energy, enthalpy, and entropy upon cyclization of *o*‐aminoguanidinobenzene molecules (B3LYP+D3/def2‐TZVP).

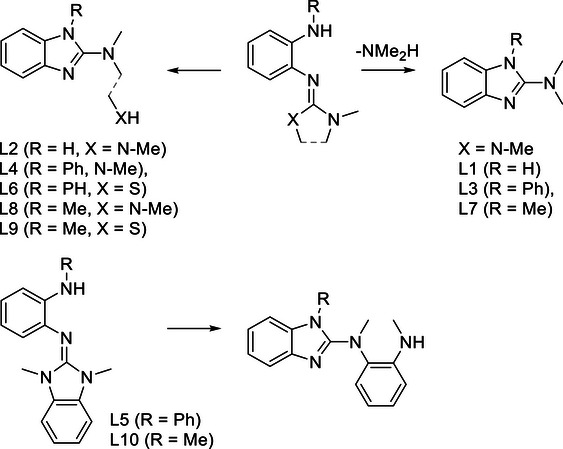			
Compound	Δ*G* / kJ mol^−1^	Δ*H* / kJ mol^−1^	Δ*S* / J·mol^−1^·K^−1^
L1 (R = H, X = N‐Me)	−49.9	+2.4	+175
L3 (R = Ph, X = N‐Me)	−52.5	−3.1	+165
L7 (R = Me, X = N‐Me)	−45.2	+3.5	+163
L2 (R = H, X = N‐Me)	−5.7	−8.0	−7.6
L4 (R = Ph, X = N‐Me)	+8.7	+7.9	−2.9
L6 (R = Ph, X = S)	+7.3	+4.5	−9.3
L8 (R = Me, X = N‐Me)	+1.1	−2.1	−10.7
L9 (R = Me, X = S)	−6.7	−8.7	−6.5
L5 (R = Ph)	+30.9	+27.4	−9.9
L10 (R = Me)	+23.2	+20.6	−8.7

In summary, our results show that compounds with specially designed guanidino groups (e.g. L5) are thermodynamically stable toward Lewis‐acid induced cyclization. Moreover, molecules L7 and L9 seem to be kinetically stabilized toward Lewis‐acid induced cyclization.

## 3 Conclusions

*o*‐Amino‐guanidinobenzene derivatives are involved as intermediates in a number of interesting reactions (see Introduction) [2, 3, 4, 6, 7, 8]. However, previous studies indicated that these species are unstable toward cyclization to give the corresponding 2‐amino‐benzimidazole molecules. Here, we presented a comprehensive analysis of the cyclization reactions of *o*‐amino‐guanidinobenzene derivatives to give 2‐amino‐imidazoles. A number of *o*‐amino‐guanidinobenzene derivatives with primary and secondary amino groups were synthesized, and their Brønsted‐ and Lewis‐acid catalysed conversion to 2‐amino‐benzimidazole derivatives analyzed. From these studies, strategies for the inhibition of cyclization to 2‐amino‐benzimidazole derivatives were developed.

The tendency for cyclization critically depends on the guanidino group. It decreases from the tetramethylguanidino (L1, L3, L7) to the *N*,*N*′‐dimethyl(ethylene)guanidino (L2, L4, L8) group and again to the 3‐methylthiazolidin‐2‐ylidene‐amino (L6, L9) and the 1,3‐dimethylimidazolidin‐2‐imino (L5) group. Cyclization of compounds with tetramethylguanidino groups is associated with a negative Δ*G* value due to the large entropy gain arising from elimination of dimethylamine in the course of cyclization. For compounds with N,N′‐dimethyl(ethylene)‐guanidino group, this driving force drops out, and the ΔG value is only slightly negative or even positive. For these compounds, cyclization is generally triggered by Brønsted or Lewis acids. On the other hand, cyclization of compounds with 1,3‐dimethylimidazolidin‐2‐imino group is associated with a clearly positive Δ*G* value; such compounds are thermodynamically stable in the *o*‐guanidino‐amino benzene form and show no tendency to isomerize.

Having several stable o‐amino‐guanidinobenzenes in hand, we tested their chemical properties. We demonstrated their use for the synthesis of redox‐active unsymmetrical diguanidinobenzenes, and analyzed their redox properties and oxidation‐induced dimerization processes. Finally, first stable coordination compounds of *o*‐amino‐guanidinobenzene derivatives were synthesized.

Based on the results of this work, it is now possible to apply *o*‐amino‐guanidinobenzene molecules as starting reagents for the synthesis of a variety of compounds, especially new redox‐active ligands with attractive properties. Cyclization of these molecules to give 2‐amino‐imidazoles is prohibited.

## 4 Experimental Details

The synthesis details and analytical data for all compounds, as well as information about the quantum‐chemical calculations are included in the Supporting Information. Deposition Number(s) 2485309 for L4, 2485310 for (L4+H)PF_6_, 2485311 for [CoBr_2_(L4)], 2485312 for [CoCl_2_(L4)], 2485313 for L5, 2485314 for 1,3‐dimethyl‐*N*‐(2‐nitrophenyl)imidazolidin‐2‐imine, 2485315 for [CoBr_2_(L8)], 2485316 for the complex of CoBr_2_ and 2‐(2‐((1,3‐dimethylimidazolidin‐2‐ylidene)amino)phenyl)‐1,1,3,3‐tetramethylguanidine, 2485317 for [CoBr_2_(L2)], 2485318 for [(L4)Li(thf)]_2_, 2485319 for *N*‐(4,5‐dimethoxy‐2‐nitrophenyl)‐1,3‐dimethylimidazolidin‐2‐imine, 2485320 for [ZnCl_2_(L4)], 2485321 for *N*‐(4,5‐dichloro‐2‐nitrophenyl)‐1,3‐dimethylimidazolidin‐2‐imine, 2485322 for [ZnCl_2_(L5)], 2485323 for the oxidation sproduct of L3, 2485324 for the oxidation product of L8, 2485325 for [ZnCl_2_(L8)], 2485326 for [ZnCl_2_(L9)], 2485327 for L7, 2485328 for the oxidation product of L7, 2485329 for *N*‐(4,5‐dimethyl‐2‐nitrophenyl)‐1,3‐dimethylimidazolidin‐2‐imine, 2485330 for *N*‐(4,5‐difluoro‐2‐nitrophenyl)‐1,3‐dimethylimidazolidin‐2‐imine, and 2491678 for [ZnCl_2_(L7)] contain(s) the supplementary crystallographic data for this paper. These data are provided free of charge by the joint Cambridge Crystallographic Data Centre and Fachinformationszentrum Karlsruhe Access Structures service.

## Conflicts of Interest

The authors declare no conflict of interest.

## Supporting information




**Supporting Information File 1**: The authors have cited additional references within the Supporting Information [34, 35, 36, 37, 38, 39, 40, 41, 42, 43, 44, 45, 46, 47, 48, 49, 50, 51, 52, 53, 54, 55, 56, 57, 58, 59, 60, 61, 62].

**Supporting Information File 2**: chem70511‐sup‐0002‐SuppMat.zip.

## Data Availability

The data that support the findings of this study are available in the supplementary material of this article.
