# Supplementary material for: Triggering and Preventing Cyclization of o‐Amino‐Guanidinobenzenes to 2‐Amino‐Benzimidazoles
Source: Chemistry. 2025 Dec 12;32(2):e03147. doi: 10.1002/chem.202503147 (PMC12790309; doi:10.1002/chem.202503147)
Supplement: Supplementary file 1 — Supporting Information File 1: The authors have cited additional references within the Supporting Information [34, 35, 36, 37, 38, 39, 40, 41, 42, 43, 44, 45, 46, 47, 48, 49, 50, 51, 52, 53, 54, 55, 56, 57, 58, 59, 60, 61, 62]. [file CHEM-32-e03147-s002.pdf]

Supporting Information

1 Table of contents

1 Table of contents i
2 General experimental details 1
3 Nitroguanidines 4
4 Reductions 34
5 Diguanidines 50
6 Diguanidine complex 63
7 Secondary amines (Phenyl residue) 67
8 Secondary (Methyl residue) 89
9 Protonation and Deprotonation Experiments 104
10 Oxidations 121
11 Complexes of secondary and primary amines 133
12 NMR Experiments 173
13 Cartesian coordinates of optimized structures 176
14 Quantum chemical calculations on the cyclisation pathway 236

2 General experimental details

All synthetic work was carried out using standard Schlenk techniques under argon atmosphere. The solvents acetonitrile, dichloromethane, diethyl ether and tetrahydrofurane were dried with a MBraun MB-SPS-800 Solvent Purification System and stored over molecular sieves. Other solvents were purchased from Acros Organics. The following chemicals were purchased and used as delivered: 2-nitroanaline (98%, Sigma Aldrich), 3,4-difluoro-6-nitroaniline (98%, BLD pharm), 4,5-dichloro-2-nitroaniline (95%, BLD pharm), 4,5-dimethyl-2-nitroaniline (98%, BLD pharm), 4,5-dimethoxy-2-nitroaniline (98%, BLD pharm), N^1-phenylbenzene-1,2-diamine (98%, BLD pharm), N^1-methylbenzene-1,2-diamine (97%, BLD pharm), triethylamine (Sigma-Aldrich), Pd on charcoal (10 wt %, Sigma-Aldrich), hydrochloride solution (2.0 M in diethylether, Sigma-Aldrich), hydrazinium hydroxid (about 100%, Sigma-Aldrich), 2-chloro-1-methyl-1H-benzo[d]imidazole (95%, BLD pharm), zinc chloride (>98%, Sigma-Aldrich), *n*-BuLi (2.5 M in hexanes, Sigma-Aldrich), 1,3-dimethyl-2-Imidazolidinone (99%, Sigma-Aldrich), 2-chloro-1H-benzo[*d*]imidazole (97%, BLD pharm), sodium hydride (90% dry, Sigma-Aldrich), methyliodid (99%, Sigma-Aldrich), trimethyloxonium tetrafluoroborate (>95%, Sigma-Aldrich) and thiazolin-2-one (97%, BLD pharm). The synthesis of ferrocenium hexafluorophosphate[1] followed the literature procedure. Elemental analysis were performed at the Microanalytical Laboratory of Heidelberg University using the vario EL and vario MICRO cube devices from Elementar Analysensysteme GmbH. Please note that the compounds are strong Brønsted bases and redox-active. Therefore, some deviations are caused by reactions with traces of water or dioxygen during the elemental analysis measurements. NMR spectra were recorded on a Bruker Avance II 400, Bruker AVANCE III 600 or Bruker 600 Ultrashield system. Solvent resonances were taken as references for all ^{1}H NMR spectra. UV-Vis spectra were recorded with a Cary 5000 spectrophotometer. CV measurements were carried out with a Metrohm Autolab PGSTAT 204 potentiostate/galvanostate and an Ag/AgCl reference electrode, Pt rod counter electrode and glassy carbon working electrode. All voltammograms were recorded at room temperature. CH_2Cl_2 and CH_3CN were used as solvents for the individual compounds (concentration around 10−3 M, if not stated otherwise), whereas $n$$Bu_4N(PF_6)$ (electrochemical grade (≥99.0%), Fluka) was employed as supporting electrolyte (c = 0.1 M). The potentials are given vs. the reference redox pair

ferrocenium/ferrocene (Fc^+ /Fc), measured at $E_{1/2}$ = 0.43 V in CH_3CN and 0.47 V in CH_2Cl_2. HR-ESI spectra were recorded with a Bruker ApexQe hybrid 9.4 T FT-ICR spectrometer and HR-EI spectra with a JEOL AccuTOF GCx. Infrared spectra were recorded as solids on an ATR crystal with a Bruker Alpha FTIR spectrometer.

2.1 X-ray crystallography

Suitable crystals for single-crystal structure determination were taken directly from the mother liquor, taken up in per-fluorinated polyether oil and fixed on a cryo loop. Full shells of intensity data were collected at low temperature with a Bruker D8 Venture, dual source (Mo-Kα radiation, microfocus X-ray tube, Photon III detector). Data were processed with the standard Bruker (SAINT, APEX3/4) software package.[2] Multiscan absorption correction was applied using the SADABS program.[3] The structures were solved by intrinsic phasing[4] and refined using the SHELXTL software package (Version 2018/3).[5] Graphical handling of the structural data during solution and refinement were performed with OLEX2.[6] All non-hydrogen atoms were given anisotropic displacement parameters. Hydrogen atoms bound to carbon were input at calculated positions and refined with a riding model. Hydrogen atoms bound to nitrogen were located in difference Fourier syntheses and refined, either fully or with appropriate distance and/or symmetry. Split atom models were used to refine disordered groups and/or solvent molecules. When found necessary, suitable geometry and adp restraints were applied.[7,8] Due to severe disorder and fractional occupancy, electron density attributed to the solvent of crystallization was removed from some of the structures with the BYPASS procedure,[9] as implemented in PLATON (squeeze/hybrid). The visualization of the structures was performed using Mercury 4.2.0. Displacement ellipsoids are depicted with 50% probability.[10]

Deposition Numbers 2485309 for L4, 2485310 for [L4+H]PF_6, 2485311 for [$CoBr_2$(L4)], 2485312 for [$CoCl_2$(L4)], 2485313 for L5, 2485314 for 1,3-dimethyl-*N*-(2-nitrophenyl)imidazolidin-2-imine, 2485315 for [$CoBr_2$(L8)], 2485316 for the complex of $CoBr_2$ and 2-(2-((1,3-dimethylimidazolidin-2-ylidene)amino)phenyl)-1,1,3,3-tetramethylguanidine, 2485317 for [$CoBr_2$(L2)], 2485318 for [Li(thf)(L4)]$_2$, 2485319 for *N*-(4,5-dimethoxy-2-nitrophenyl)-1,3-dimethylimidazolidin-2-imine, 2485320 for [$ZnCl_2$(L4)], 2485321 for *N*-(4,5-dichloro-2-nitrophenyl)-1,3-dimethylimidazolidin-2-imine, 2485322 for [$ZnCl_2$(L5)], 2485323 for the oxidation product of L3, 2485324 for

the oxidation product of L8, 2485325 for [$ZnCl_2$(L8)], 2485326 for [$ZnCl_2$(L9)], 2485327 for L7, 2485328 for the oxidation product of L7, 2485329 for *N*-(4,5-dimethyl-2-nitrophenyl)-1,3-dimethylimidazolidin-2-imine, 2485330 for *N*-(4,5-difluoro-2-nitrophenyl)-1,3-dimethylimidazolidin-2-imine, 2491678 for [$ZnCl_2$(L7)] contain the supplementary crystallographic data for this paper. These data are provided free of charge by the joint Cambridge Crystallographic Data Centre and Fachinformationszentrum Karlsruhe Access Structure service.

---

[1] U. Jahn, P. Hartmann, I. Dix, P.G. Jones, *Eur. J. Org. Chem.* **2001**, *17*, 3333–3355.

[2] SAINT (APEX III/IV) Bruker AXS GmbH, Karlsruhe, Germany **2016/2021**.

[3] a) G. M. Sheldrick, SADABS, Bruker AXS GmbH, Karlsruhe, Germany **2004-2014**; b) L. Krause, R. Herbst-Irmer, G. M. Sheldrick, D. Stalke, *J. Appl. Cryst*. **2015**, *48*, 3–10.

[4] a) G. M. Sheldrick, SHELXT, Program for Crystal Structure Solution, University of Göttingen, Germany **2014-2018**; b) G. M. Sheldrick, *Acta Cryst*. **2015**, *C71*, 3–8.

[5] a) G. M. Sheldrick, SHELXL-20xx, University of Göttingen and Bruker AXS GmbH, Karlsruhe, Germany **2012-2018**; b) W. Robinson, G. M. Sheldrick in: N. W. Isaaks, M. R. Taylor (eds.) *„Crystallographic Computing 4“*, Ch. 22, IUCr and Oxford University Press, Oxford, UK, **1988**; c) G. M. Sheldrick*, Acta Cryst.* **2008**, *A64*, 112–122.

[6] O. V. Dolomanov, L. J. Bourhis, R. J. Gildea, J. A. K. Howard, H. Puschmann, *J. Appl. Cryst.* **2009**, *42*, 339–341.

[7] A. Thorn, B. Dittrich, G. M. Sheldrick, *Acta Cryst.* **2012**, *A68*, 448–451.

[8] a) P. v. d. Sluis, A. L. Spek*, Acta Cryst.* **1990**, *A46*, 194–201; b) A. L. Spek, *Acta Cryst.* **2015**, *C71*, 9–18.

[9] A. L. Spek, PLATON, Utrecht University, The Netherlands; b) A. L. Spek, J. *Appl. Cryst.* **2003**, *36*, 7–13.

[10] C. F. Macrae, I. Sovago, S. J. Cottrell, P. T. A. Galek, P. McCabe, E. Pidcock, M. Platings, G. P. Shields, J. S. Stevens, M. Towler and P. A. Wood, *J. Appl. Cryst.* **2020**, *53*, 226–235.

3 Nitroguanidines

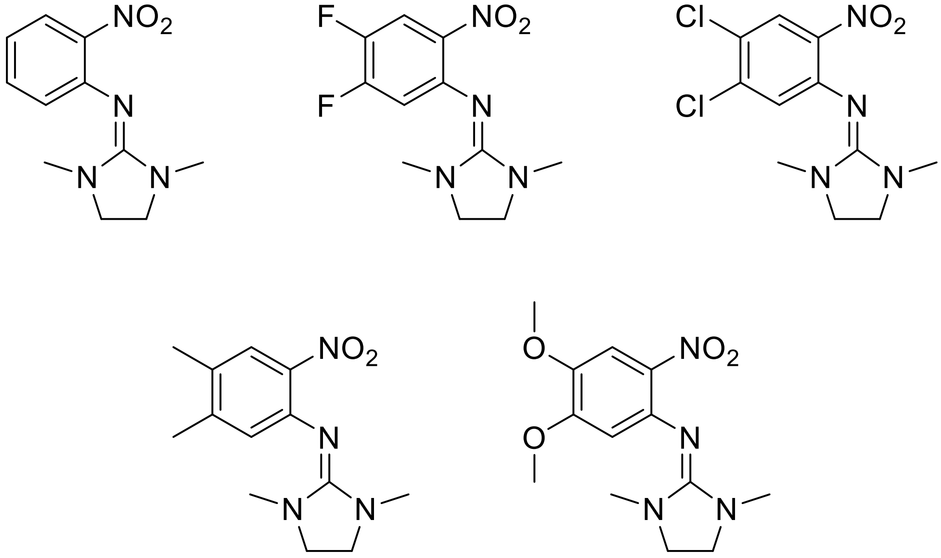

Scheme S1: Overview of the nitroguanidines shown with -H, -F, -Cl, -Me and -OMe in the backbone.

3.1 1,3-dimethyl-*N*-(2-nitrophenyl)imidazolidin-2-imine

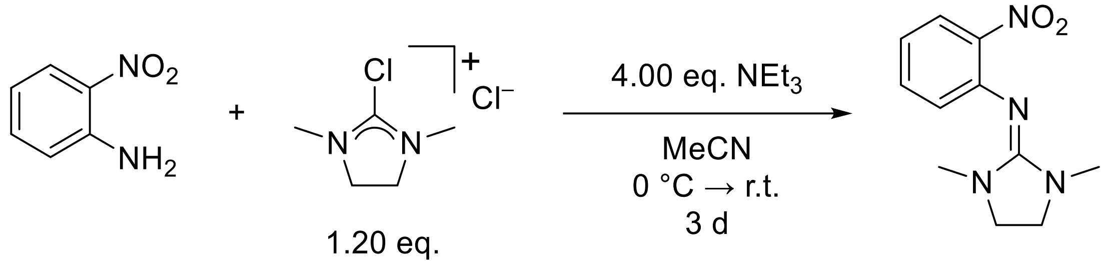

500 mg of 2-nitroaniline (1.00 eq., 3.62 mmol) and 1.20 eq. activated urea (734 mg, 4.34 mmol) were dissolved in 5.00 mL acetonitrile under ice-cooling and 2.02 mL triethylamine (1.47 mg, 4.00 eq., 14.5 mmol) was added. The reaction mixture was stirred overnight. The solution was filtered and removed *in vacuo*. Then, the residue was mixed with 8.00 mL of 15% degassed sodium hydroxide solution and extracted with 3 × 5.00 mL diethyl ether. The combined organic phases were dried over magnesium sulfate. The product was obtained as an orange solid in 70% yield (600 mg, 2.56 mmol). Crystals were obtained by scratching the glass at 35 °C (orange liquid) with a spatula.

^{1}H NMR (600 MHz, $CDCl_3$): δ = 7.83-7.81 (m, 1 H, CH_{arom}), 7.34-7.32 (m, 1 H, CH_{arom}), 7.02-7.00 (m, 1 H, CH_{arom}), 6.83-6.82 (m, 1 H, CH_{arom}), 3.35 (s, 4 H, 2×CH_2), 2.67 (s, 6 H, 2×CH_3) ppm.

^{13}C NMR (151 MHz, $CDCl_3$): δ = 157.11 ($C_{q,Guan}$), 146.42 ($C_{q,arom}$), 141.64 ($C_{q,arom}$), 133.26 (CH_{arom}), 126.52 (CH_{arom}), 125.34 (CH_{arom}), 119.23 (CH_{arom}), 48.35 (NCH_2), 34.75 (NCH_3) ppm.

UV-Vis (DCM, c = 1.786710^{-4} mol·l^{-1}, d = 1 cm): λ_{max} (ε [l·mol^{-1}·cm^{-1}]) = 262 (3576), 393 (broad, 827) nm.

IR (ATR): $\tilde{\nu}$ = 2931.89, 2859.77, 1623.36, 1588.96, 1556.77, 1502.10, 1471.00, 1441.59, 1394.68, 1231.62, 1197.63, 1154.17, 1139.65, 1075.48, 968.31, 945.27, 668.23, 642.08, 585.13, 505.78, 474.51, 420.18 cm^{-1}.

Elemental analysis (%):

calculated:	C: 56.40	H: 6.02	N: 23.92
found:	C: 56.46	H: 6.18	N: 23.87

Analytical data:

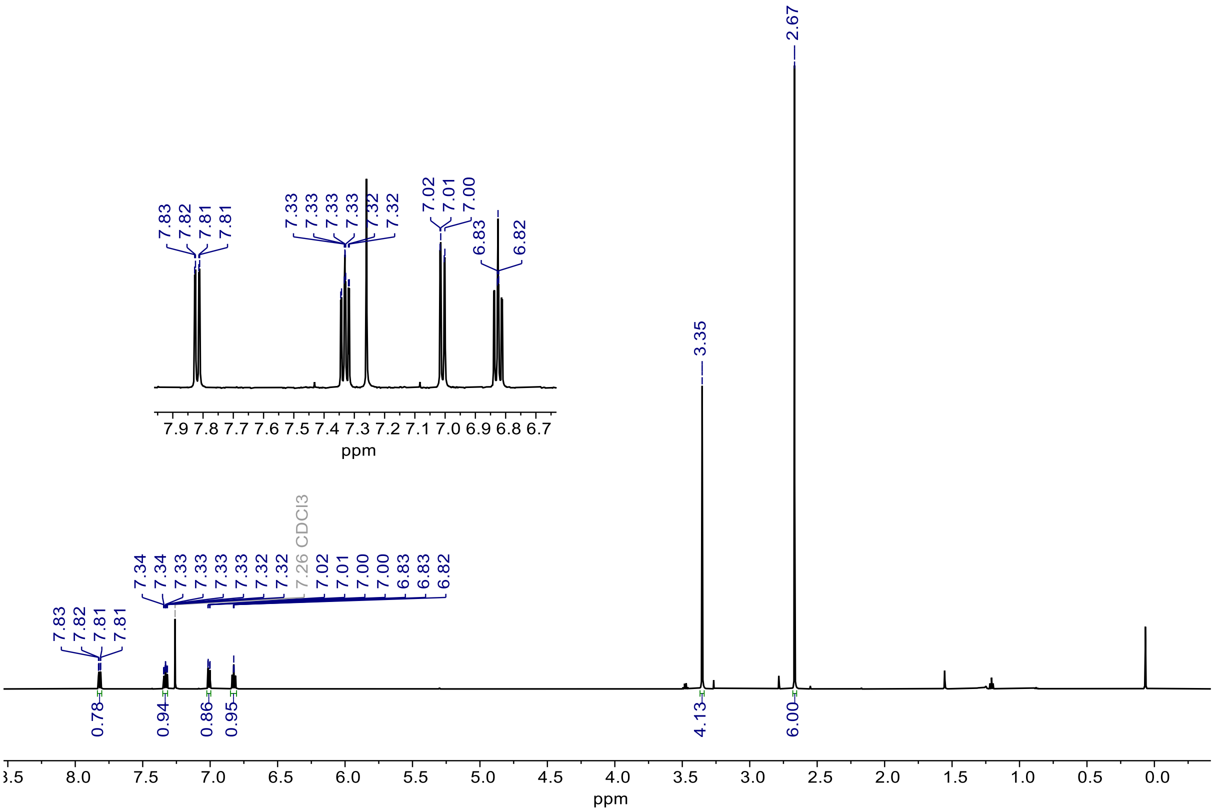

Figure S1: ^{1}H NMR spectrum (600 MHz, $CDCl_3$) of 1,3-dimethyl-*N*-(2-nitrophenyl)imidazolidin-2-imine. The impurities at 0.07 ppm and 1.21 ppm are due to grease, while the impurity at 1.55 ppm is due to water.

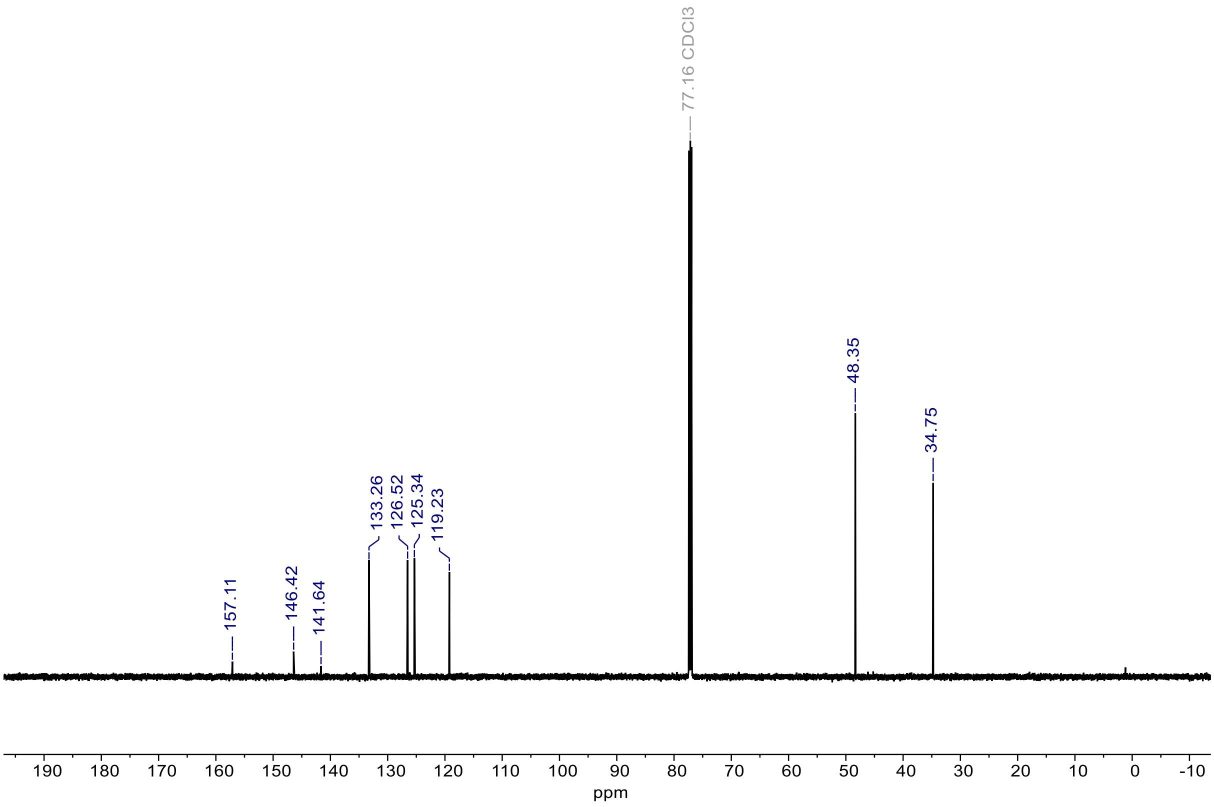

Figure S2: ^{13}C NMR spectrum (151 MHz, $CDCl_3$) of 1,3-dimethyl-*N*-(2-nitrophenyl)imidazolidin-2-imine.

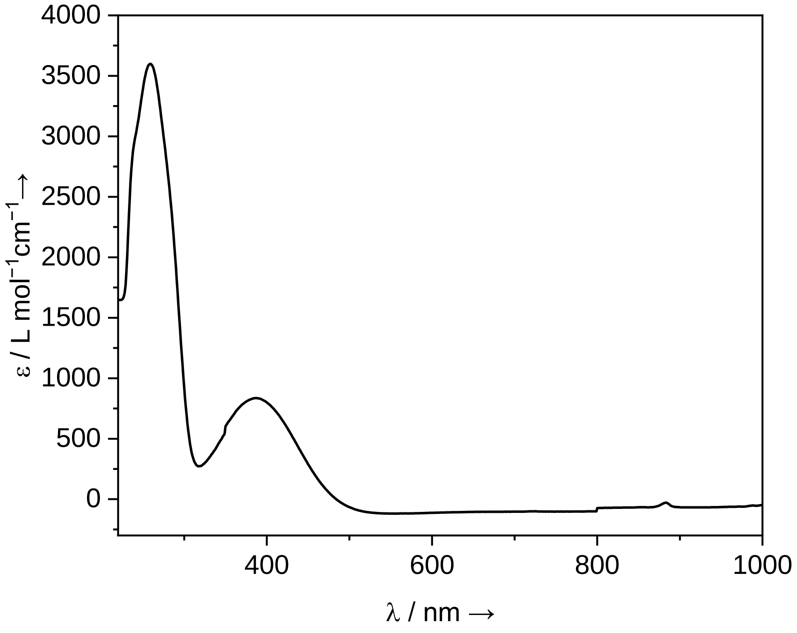

Figure S3: UV-Vis spectrum of 1,3-dimethyl-*N*-(2-nitrophenyl)imidazolidin-2-imine in DCM.

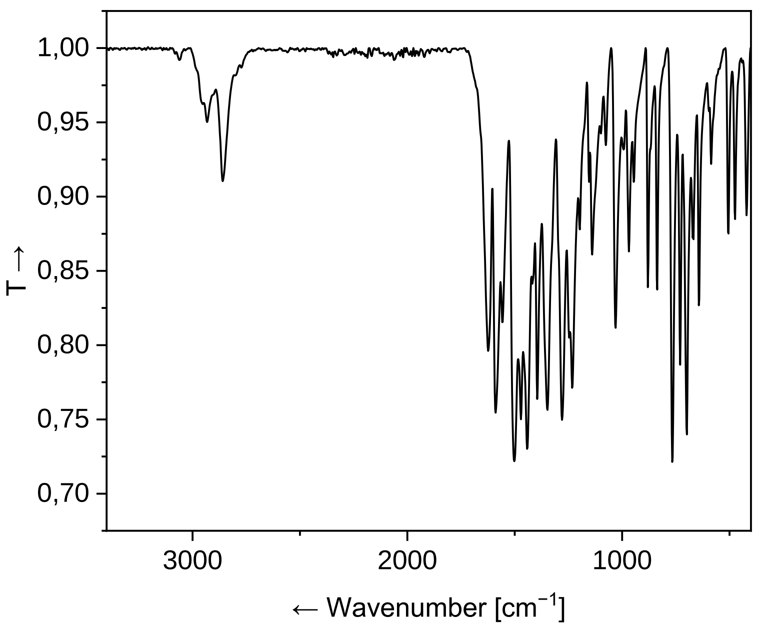

Figure S4: IR spectrum of 1,3-dimethyl-*N*-(2-nitrophenyl)imidazolidin-2-imine.

Table S1: Illustration of the structure of 1,3-dimethyl-*N*-(2-nitrophenyl)imidazolidin-2-imine in the solid state, together with crystallographic data. H atoms have been omitted for clarity. Displacement ellipsoids correspond to 50% probability of residence. Colour coding: C dark-grey, N blue, O red.

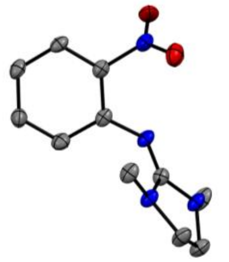

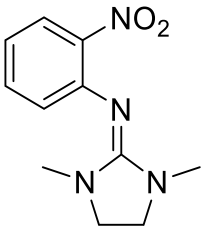

Empirical formula	$C_{11}H_{14}N_4O_2$
Formula weight	234.26
Temperature/K	100.00
Crystal system	monoclinic
Space group	Cc
a/Å	23.141(3)
b/Å	8.0678(10)
c/Å	15.122(2)
α/°	90
β/°	123.716(8)

γ/°	90
Volume/Å^3	2348.3(6)
Z	8
ρ_{calc}g/cm^3	1.325
μ/mm^{-1}	0.095
F(000)	992.0
Crystal size/mm^3	0.323 × 0.155 × 0.078
Radiation	MoKα (λ = 0.71073)
2Θ range for data collection/°	4.232 to 54.534
Index ranges	-29 ≤ h ≤ 29, -10 ≤ k ≤ 10, -19 ≤ l ≤ 19
Reflections collected	36715
Independent reflections	5231 [R_{int} = 0.0824, R_{sigma} = 0.0477]
Data/restraints/parameters	5231/3/320
Goodness-of-fit on F^2	1.104
Final R indexes [I>=2σ (I)]	R_1 = 0.0417, wR_2 = 0.0998
Final R indexes [all data]	R_1 = 0.0482, wR_2 = 0.1036
Largest diff. peak/hole / e Å^{-3}	0.21/-0.24
Flack parameter	0.0(6)

3.2 *N*-(4,5-difluoro-2-nitrophenyl)-1,3-dimethylimidazolidin-2-imine

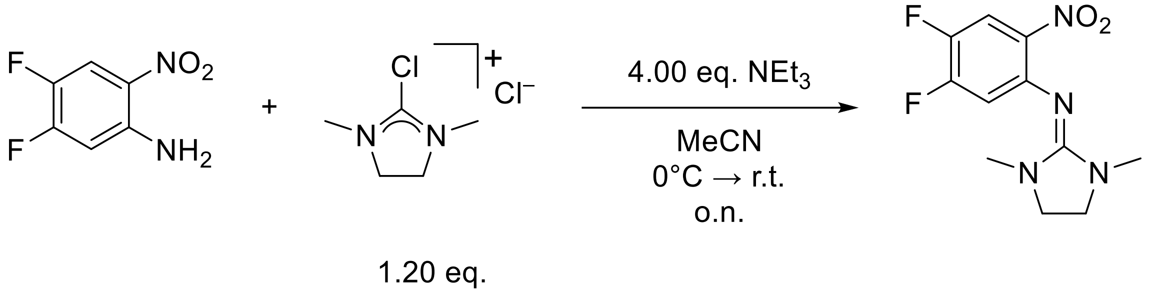

300 mg 4,5-difluoro-2-nitroaniline (1.00 eq., 1.72 mmol) and 1.20 eq. activated urea (378 mg, 2.35 mmol) were dissolved in 5.00 mL acetonitrile under ice-cooling, and 960 µL triethylamine (697 mg, 4.00 eq., 6.89 mmol) was added. The reaction mixture was stirred overnight. The solution was filtered and the solvent was removed *in vacuo*. The residue was mixed with 6.00 ml of 15% degassed sodium hydroxide solution and extracted with 5 × 2.00 mL diethyl ether. The combined organic phases were dried over magnesium sulfate. The product was obtained as an orange solid in 86% yield (398 mg, 1.47 mmol). Crystals were obtained after partial solvent removal under vacuum.

^{1}H NMR (600 MHz, $CDCl_3$): δ = 7.80 - 7.77(s, 1 H, CH_{arom}), 6.80-6.77 (s, 1 H, CH_{arom}), 3.39(s, 4 H, 2xCH_2), 2.68(s, 6 H, 2xCH_3) ppm.

^{13}C NMR (151 MHz, $CDCl_3$): δ = 157.86 ($C_{q,Guan}$), 154.68 ($C_{q,arom}$), 153.06 ($C_{q,arom}$), 114.24 - 114.08 (dd, *J* = 21.5 Hz, 3.1 Hz, CH$_{arom}$), 113.69 - 113.57 (d, *J* = 17.7 Hz, CH$_{arom}$), 48.23 (NCH$_2$), 34.62 (NCH$_3$) ppm.

^{19}F NMR (565 MHz, $CDCl_3$): δ = -128.23 – -128.31 (m, 1 F), -147.05 – -147.15 (m, 1 F) ppm.

UV-Vis (DCM, *c* = 1.406157·10^{-5} mol·l^{-1}, *d* = 1 cm): λ_{max} (*ε* [l·mol^{-1}·cm^{-1}]) = 255 (4347), 290 (3085), 400 (1975) nm.

IR (ATR): $\tilde{\nu}$ = 3063.45, 2960.90, 2874.66, 1609.83, 1567.47, 1403.82, 1216.49, 1200.65, 1178.03, 1133.98, 1076.03, 1048.99, 1025.74, 971.32, 887.15, 876.56, 736.27, 693.05, 681.27, 663.78, 638.85, 576.62, 535.00, 460.96, 420.70 cm^{-1}.

Elemental analysis (%):

calculated:	C: 48.89	H: 4.48	N: 20.73
found:	C: 48.69	H: 4.60	N: 20.45

Analytical data:

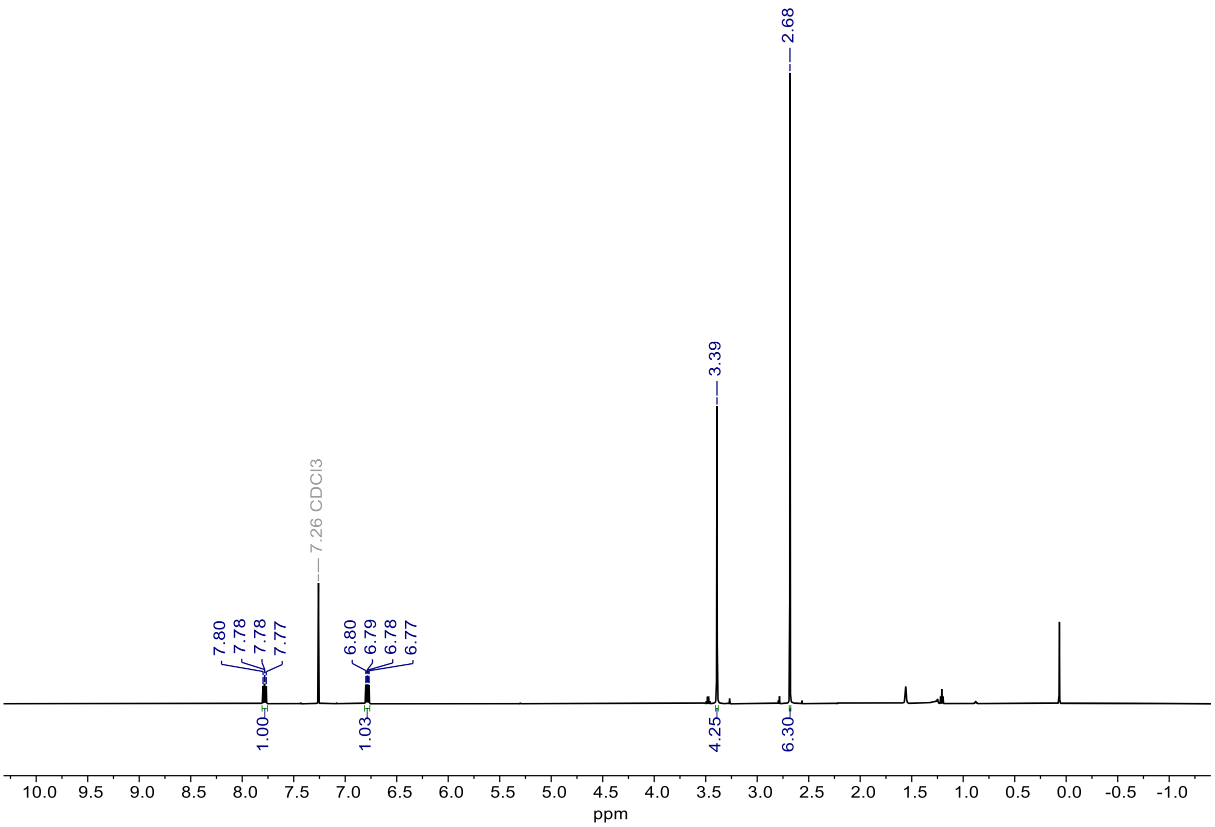

Figure S5: ^{1}H NMR spectrum (600 MHz, $CDCl_3$) of *N*-(4,5-difluoro-2-nitrophenyl)-1,3-dimethylimidazolidin-2-imine. The impurities at 0.07 ppm and 1.21 ppm are due to grease, while the impurity at 1.55 ppm is due to water.

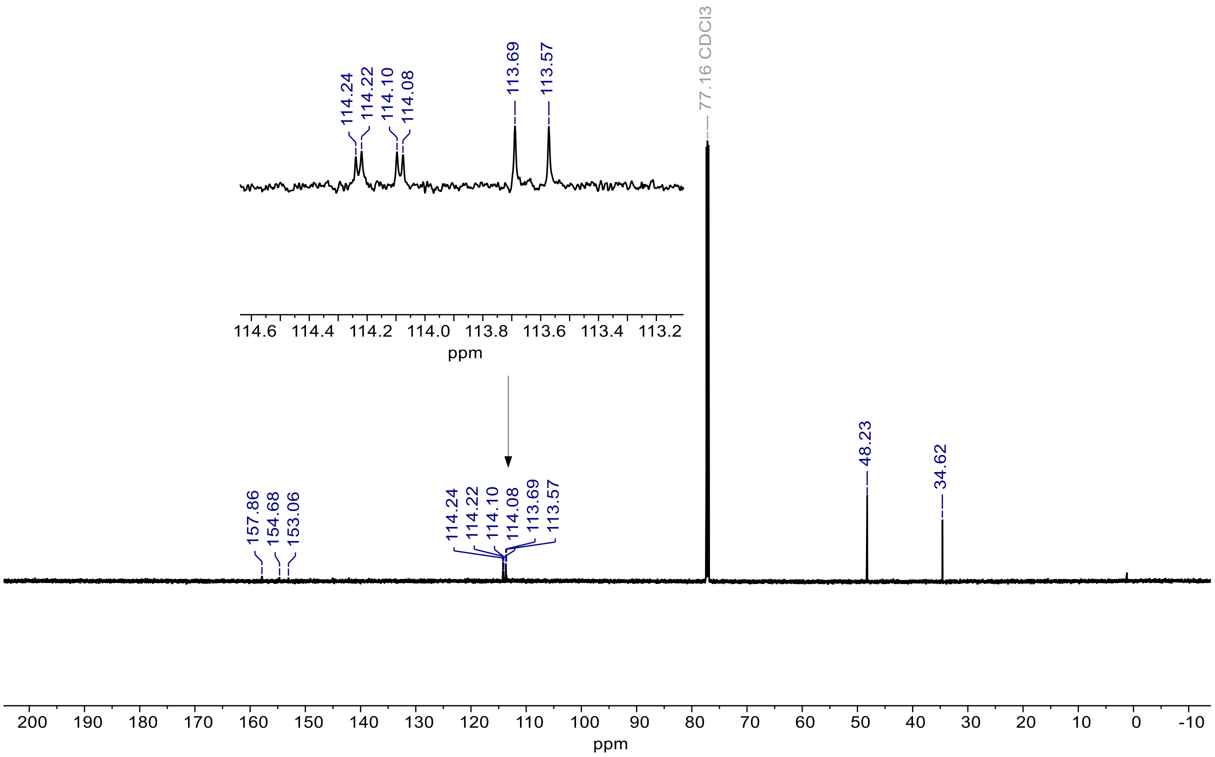

Figure S6: ^{13}C NMR spectrum (151 MHz, $CDCl_3$) of *N*-(4,5-difluoro-2-nitrophenyl)-1,3-dimethylimidazolidin-2-imine.

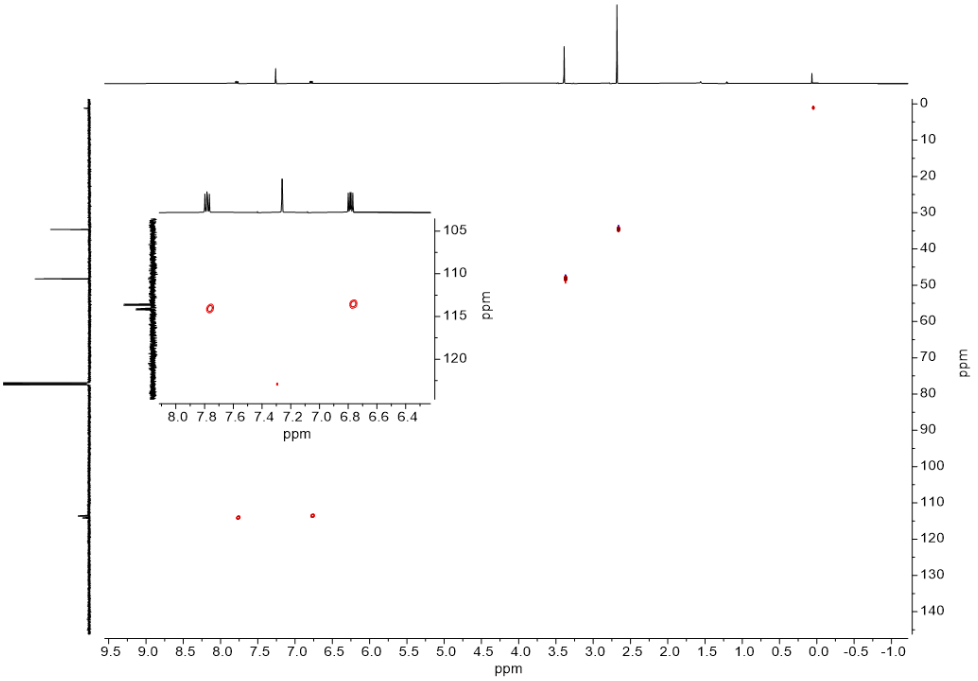

Figure S7: HSQC (^{1}H-^{13}C) spectrum of *N*-(4,5-difluoro-2-nitrophenyl)-1,3-dimethylimidazolidin-2-imine.

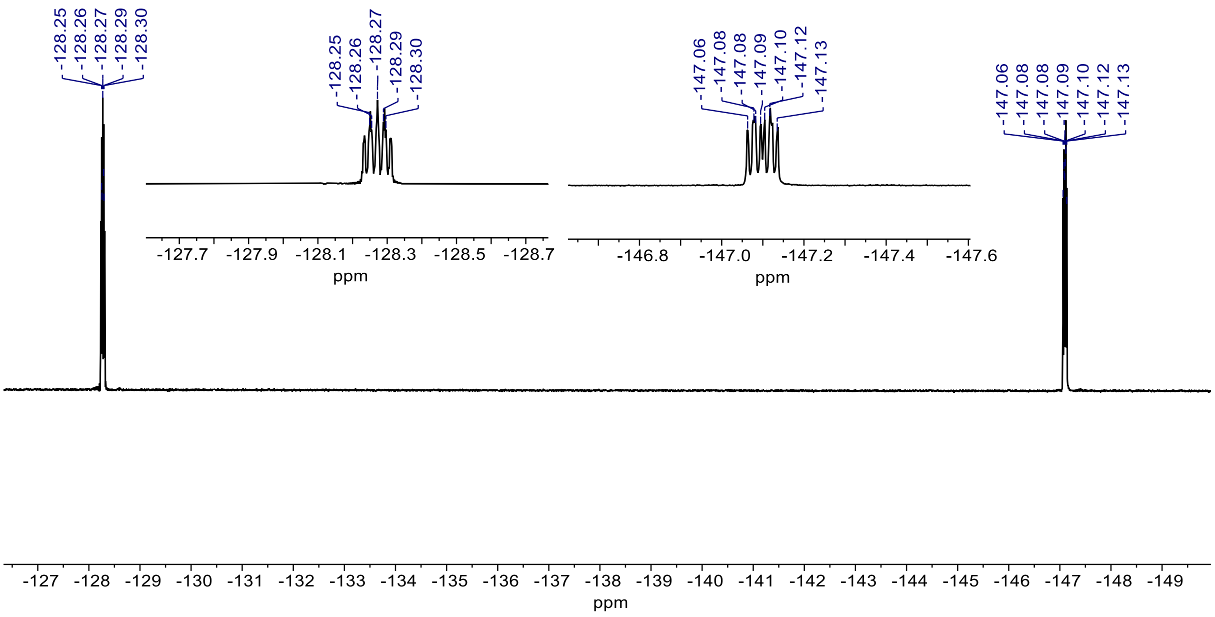

Figure S8: ^{19}F NMR spectrum (565 MHz, $CDCl_3$) of *N*-(4,5-difluoro-2-nitrophenyl)-1,3-dimethylimidazolidin-2-imine.

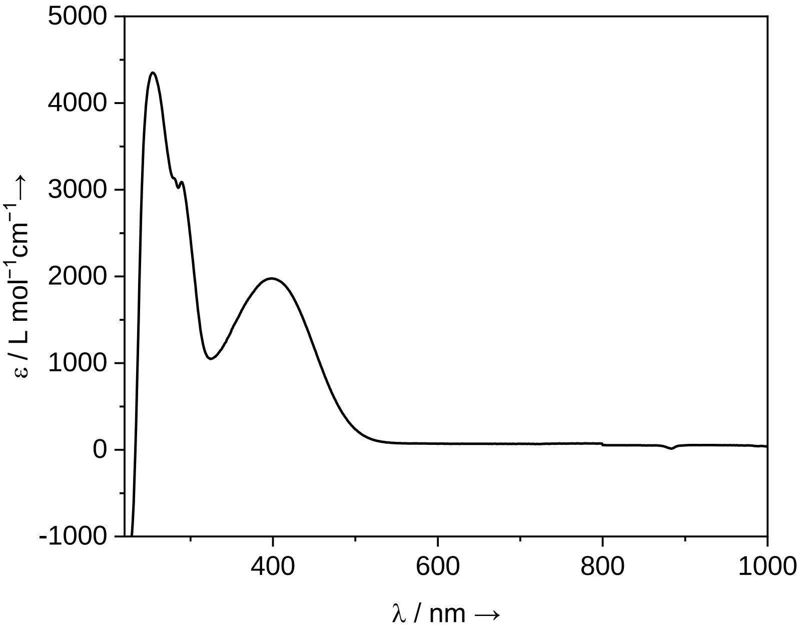

Figure S9: UV-Vis spectrum of *N*-(4,5-difluoro-2-nitrophenyl)-1,3-dimethylimidazolidin-2-imine in DCM.

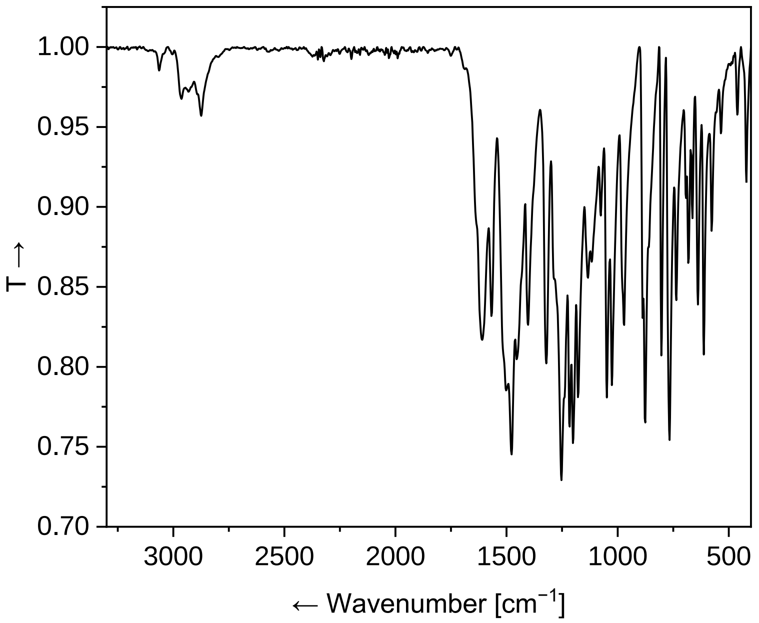

Figure S10: IR spectrum of *N*-(4,5-difluoro-2-nitrophenyl)-1,3-dimethylimidazolidin-2-imine.

Table S2: Illustration of the structure of *N*-(4,5-difluoro-2-nitrophenyl)-1,3-dimethylimidazolidin-2-imine in the solid state, together with crystallographic data. H atoms have been omitted for clarity. Displacement ellipsoids correspond to 50% probability of residence. Colour coding: C dark-grey, N blue, O red, F pale yellow.

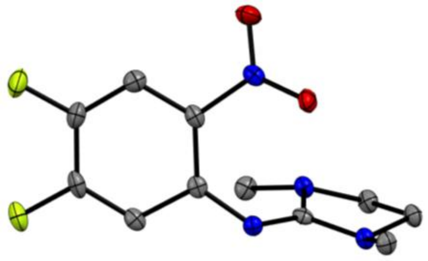

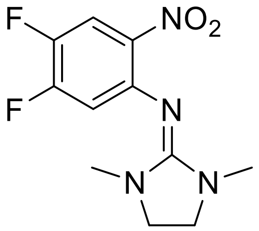

Empirical formula	$C_{11}H_{12}F_2N_4O_2$
Formula weight	270.25
Temperature/K	100.00
Crystal system	triclinic
Space group	P-1
a/Å	6.7088(6)
b/Å	8.0923(7)
c/Å	11.0629(10)
α/°	79.822(4)
β/°	76.130(4)

γ/°	89.225(4)
Volume/Å^3	573.65(9)
Z	2
ρ_{calc}g/cm^3	1.565
μ/mm^{-1}	0.133
F(000)	280.0
Crystal size/mm^3	0.17 × 0.15 × 0.08
Radiation	MoKα (λ = 0.71073)
2Θ range for data collection/°	5.116 to 54.38
Index ranges	-8 ≤ h ≤ 8, -10 ≤ k ≤ 10, -14 ≤ l ≤ 14
Reflections collected	17460
Independent reflections	2559 [R_{int} = 0.0843, R_{sigma} = 0.0470]
Data/restraints/parameters	2559/0/174
Goodness-of-fit on F^2	1.056
Final R indexes [I>=2σ (I)]	R_1 = 0.0471, wR_2 = 0.1201
Final R indexes [all data]	R_1 = 0.0605, wR_2 = 0.1286
Largest diff. peak/hole / e Å^{-3}	0.28/-0.30

3.3 *N*-(4,5-dichloro-2-nitrophenyl)-1,3-dimethylimidazolidin-2-imine

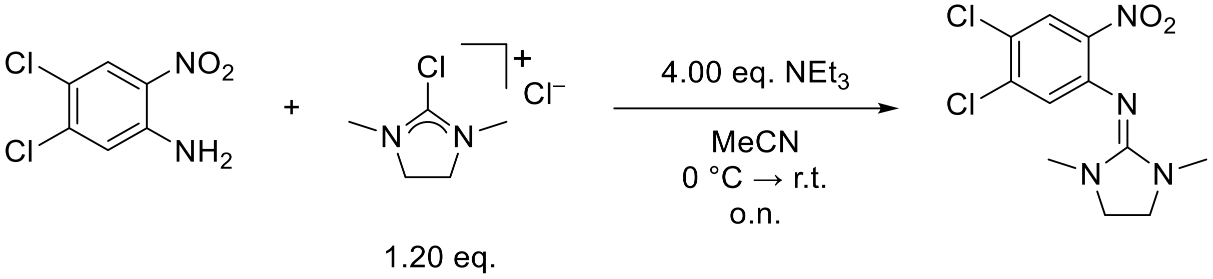

300 mg of 4,5-dichloro-2-nitroaniline (1.00 eq., 1.45 mmol) and 1.30 eq. activated urea (318 mg, 1.88 mmol) were dissolved in 3.00 mL acetonitrile under ice-cooling and 808 µL triethylamine (587 mg, 4.00 eq., 5.80 mmol) was added. The reaction mixture was stirred overnight. The solution was filtered and the solvent was removed *in vacuo*. The residue was mixed with 6.00 mL of 15% degassed sodium hydroxide solution and extracted with 3 × 2.00 mL diethyl ether. The combined organic phases were dried over magnesium sulfate. The product was obtained as an orange-brown solid in 75% yield (329 mg, 301 µmol). Crystals were obtained after partial solvent removal under vacuum.

^{1}H NMR (400 MHz, $CDCl_3$): δ = 7.96 (s, 1 H, CH_{arom}), 7.10 (s, 1 H, CH_{arom}), 3.40 (s, 4 H, 2xCH_2), 2.70(s, 6 H, 2xCH_3) ppm.

^{13}C NMR (101 MHz, $CDCl_3$): δ = 158.07 ($C_{q,Guan}$), 146.02 ($C_{q,arom}$), 139.29 ($C_{q,arom}$), 137.63 ($C_{q,arom}$), 127.20 (CH$_{arom}$), 126.60 (CH$_{arom}$), 121.36 ($C_{q,arom}$), 48.19 (NCH$_2$), 34.63 (NCH$_3$) ppm.

UV-Vis (DCM, c = 1.18·10^{-4} mol·l^{-1}, d = 1 cm): λ_{max} (ε [l·mol^{-1}·cm^{-1}]) = 266 (1553), 292 (1482, shoulder), 410 (755) nm.

IR (ATR): $\tilde{\nu}$ = 3085.05, 2965.15, 2862.39, 1620.82, 1581.15, 1528.61, 1503.42, 1487.82, 1438.81, 1401.10, 1328.73, 1281.42, 1254.23, 1221.88, 1193.33, 1165.72, 1126.53, 1070.40, 1034.30, 976.41, 853.60, 775.01, 764.16, 729.90, 687.45, 643.69, 618.41, 605.33, 567.54, 536.06, 490.30, 453.49, 425.41 cm^{-1}.

Elemental analysis (%):

calculated:	C: 43.58	H: 3.99	N: 18.48
found:	C: 43.21	H: 4.08	N: 18.32

Analytical data:

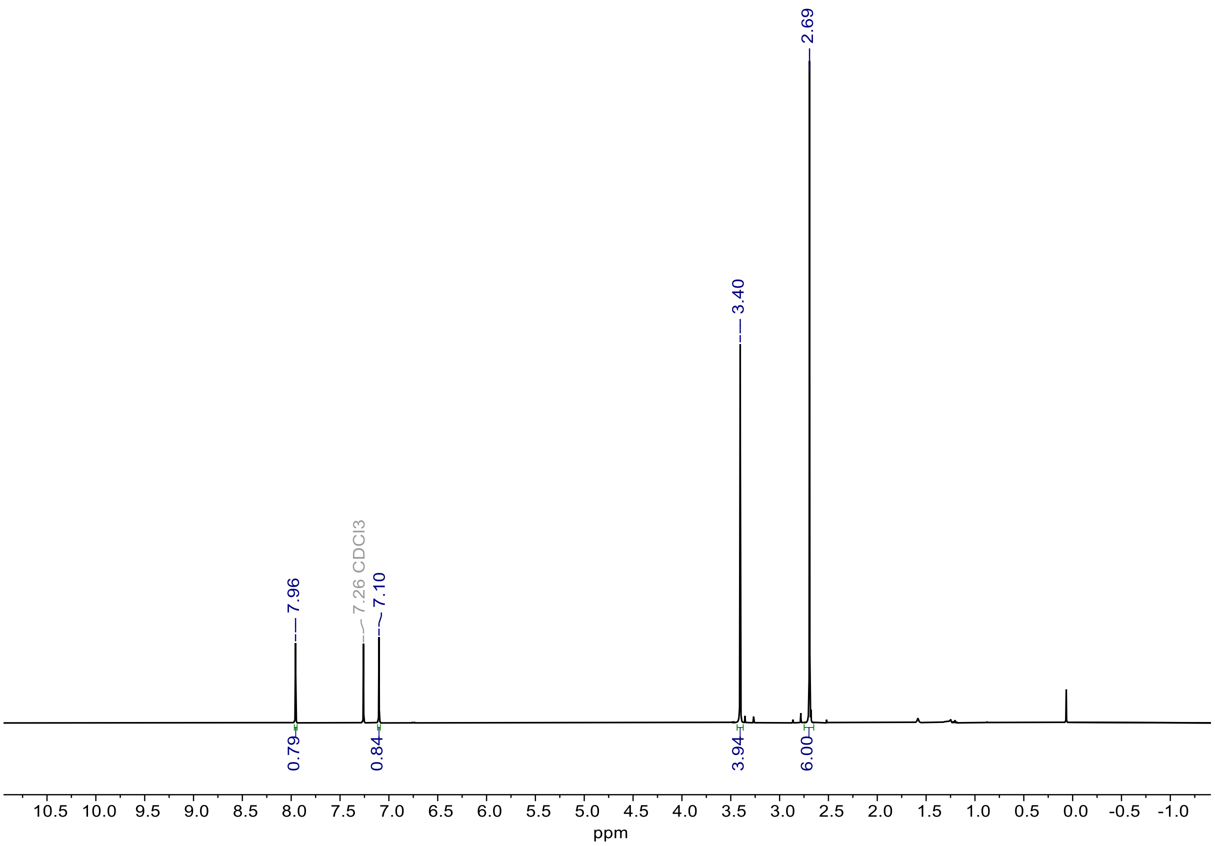

Figure S11: ^{1}H NMR spectrum (400 MHz, $CDCl_3$) of *N*-(4,5-dichloro-2-nitrophenyl)-1,3-dimethylimidazolidin-2-imine. The impurity at 0.07 ppm is due to grease.

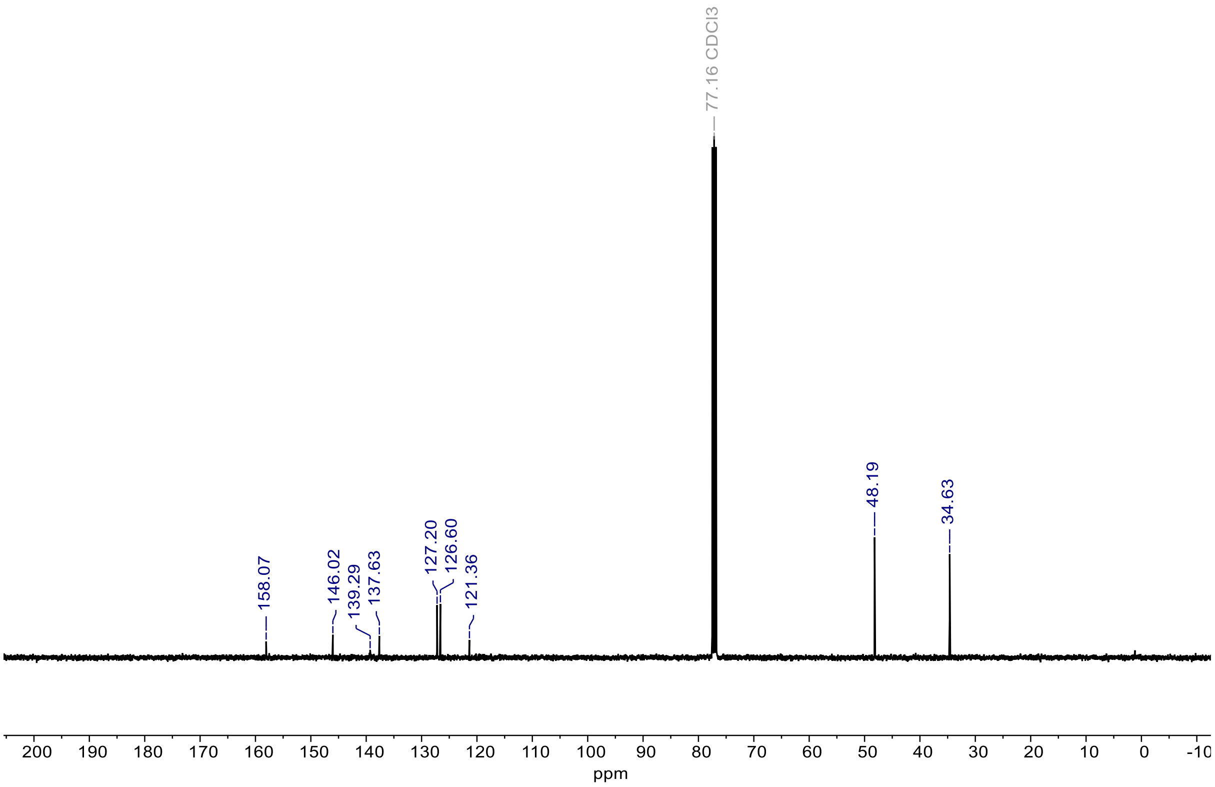

Figure S12: ^{13}C NMR spectrum (101 MHz, $CDCl_3$-*d1*) of *N*-(4,5-dichloro-2-nitrophenyl)-1,3-dimethylimidazolidin-2-imine.

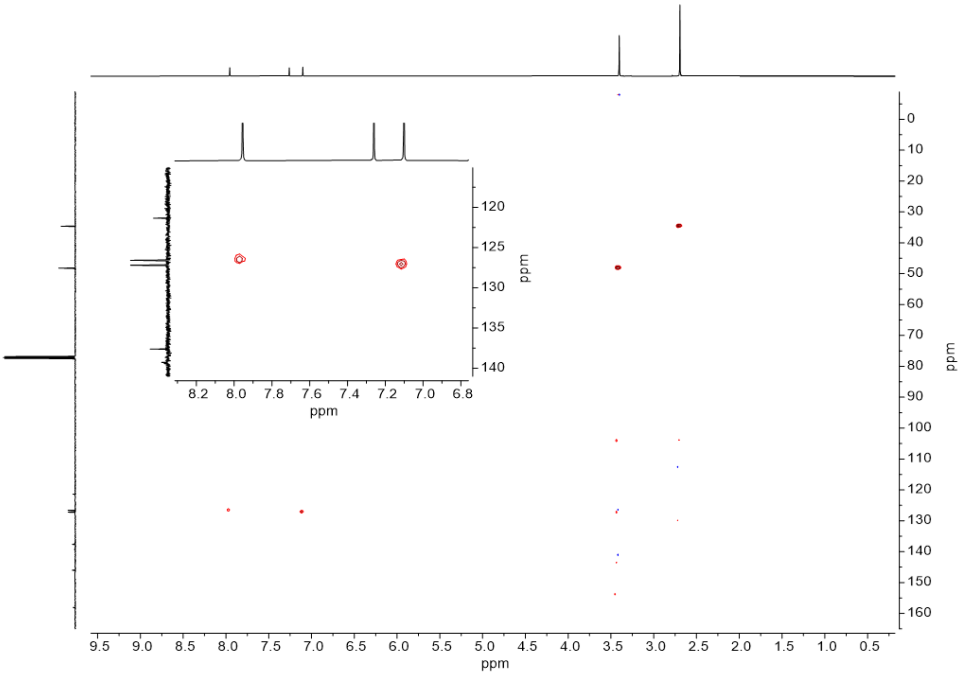

Figure S13: HSQC (^{1}H-^{13}C) spectrum of *N*-(4,5-dichloro-2-nitrophenyl)-1,3-dimethylimidazolidin-2-imine.

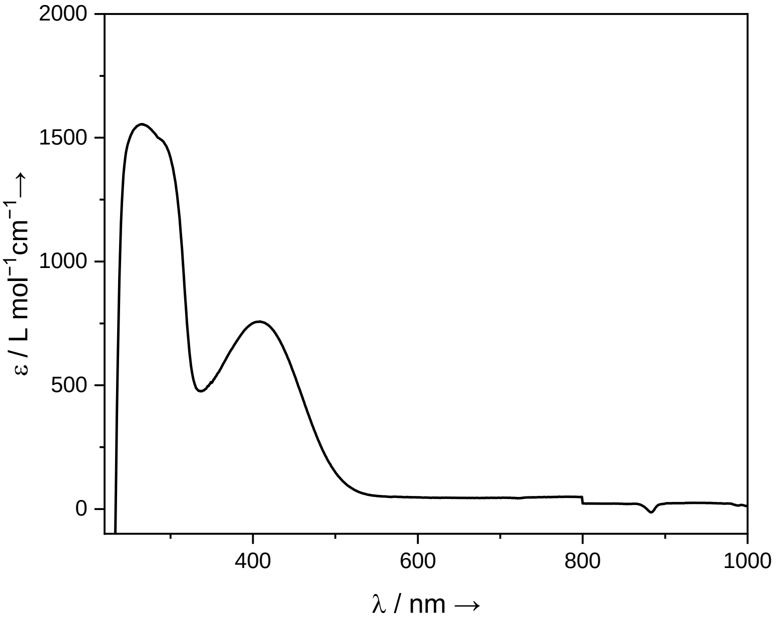

Figure S14: UV-Vis spectrum of *N*-(4,5-dichloro-2-nitrophenyl)-1,3-dimethylimidazolidin-2-imine in DCM.

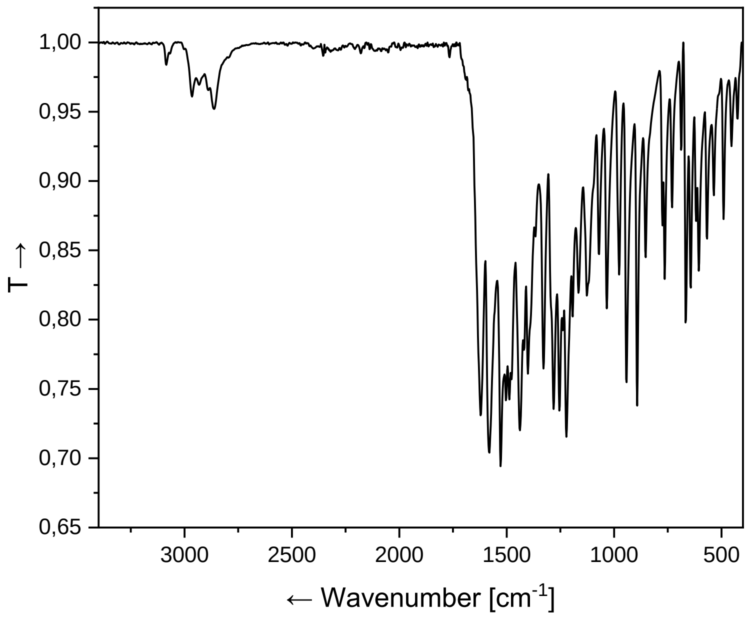

Figure S15: IR spectrum of *N*-(4,5-dichloro-2-nitrophenyl)-1,3-dimethylimidazolidin-2-imine.

Table S3: Illustration of the structure of *N*-(4,5-dichloro-2-nitrophenyl)-1,3-dimethylimidazolidin-2-imine in the solid state, together with crystallographic data. H atoms have been omitted for clarity. Displacement ellipsoids correspond to 50% probability of residence. Colour coding: C dark-grey, N blue, Cl green.

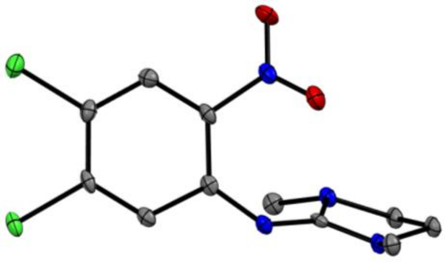

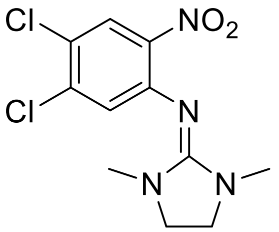

Empirical formula	$C_{11}H_{12}Cl_2N_4O_2$
Formula weight	303.15
Temperature/K	100.00
Crystal system	triclinic
Space group	P-1
a/Å	6.8162(8)
b/Å	8.1460(10)
c/Å	11.7276(14)
α/°	100.676(4)
β/°	100.862(4)
γ/°	91.556(5)
Volume/Å^3	627.14(13)
Z	2
ρ_{calc}g/cm^3	1.605
μ/mm^{-1}	0.521
F(000)	312.0
Crystal size/mm^3	0.169 × 0.168 × 0.093
Radiation	MoKα (λ = 0.71073)

2Θ range for data collection/°	5.1 to 53.258
Index ranges	$-8 \le h \le 8$, $-10 \le k \le 10$, $-14 \le l \le 14$
Reflections collected	26436
Independent reflections	2575 [R_{int} = 0.0613, R_{sigma} = 0.0356]
Data/restraints/parameters	2575/0/174
Goodness-of-fit on F^2	1.059
Final R indexes [I>=2σ (I)]	R_1 = 0.0375, wR_2 = 0.0979
Final R indexes [all data]	R_1 = 0.0422, wR_2 = 0.1021
Largest diff. peak/hole / e Å^{-3}	0.52/-0.31

3.4 *N*-(4,5-dimethyl-2-nitrophenyl)-1,3-dimethylimidazolidin-2-imine

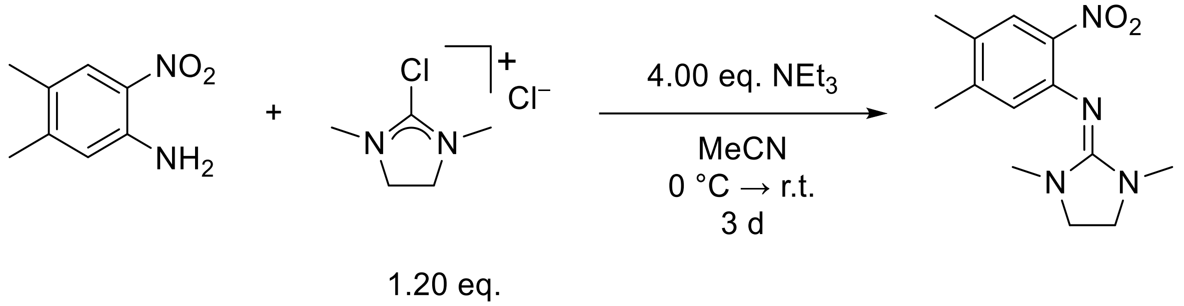

300 mg of 4,5-dimethyl-2-nitroaniline (1.00 eq., 1.81 mmol) and 1.20 eq. activated urea (397 mg, 2.35 mmol) were dissolved in 6.00 mL acetonitrile under ice-cooling and 1.01 mL triethylamine (730 mg, 4.00 eq., 7.22 mmol) was added. The reaction mixture was stirred overnight. The solution was filtered and the solvent was removed *in vacuo*. The residue was mixed with 8.00 mL of 15% degassed sodium hydroxide solution and extracted with 3 × 2.00 mL diethyl ether. The combined organic phases were dried over magnesium sulfate. The product was obtained as a deep red solid in 70% yield (330 mg, 1.26 mmol). Crystals were obtained after partial solvent removal under vacuum.

^{1}H NMR (600 MHz, $CDCl_3$): δ = 7.67 (s, 1 H, CH_{arom}), 6.81 (s, 1 H, CH_{arom}), 3.33 (s, 4 H, 2xCH_2), 2.65 (s, 6 H, 2xCH_3), 2.22 (s, 3 H, 1xC$H_{3,arom}$), 2.20 (s, 3 H, 1xC$H_{3,arom}$) ppm.

^{13}C NMR (151 MHz, $CDCl_3$): δ = 156.83 ($C_{q,Guan}$), 144.59 ($C_{q,arom}$), 143.61 ($C_{q,arom}$), 139.00 ($C_{q,arom}$), 128.29 ($C_{q,arom}$), 127.33 (CH$_{arom}$), 125.77 (CH$_{arom}$), 48.40 (NCH$_2$), 34.78 (NCH$_3$), 20.06 (CH$_{3,arom}$), 18.96 (CH$_{3,arom}$) ppm.

UV-Vis (DCM, c = 1.2199·10^{-4} mol·l^{-1}, d = 1 cm): λ_{max} (ε [l·mol^{-1}·cm^{-1}]) = 255 (1798), 395 (1160, broad) nm.

IR (ATR): $\tilde{\nu}$ = 2950.58, 2857.31, 1632.27, 1605.46, 1544.08, 1493.21, 1470.23, 1444.35, 1415.57, 1391.55, 1322.16, 1268.98, 1227.99, 1192.68, 1134.62, 1054.25, 1000.11, 982.25, 966.53, 892.36, 876.97, 863.66, 797.71, 773.89, 746.64, 733.45, 690.05, 664.95, 650.86, 617.19, 594.81, 557.04, 533.18, 456.96, 421.58 cm^{-1}.

Elemental analysis (%):

calculated:	C: 59.53	H: 6.92	N: 21.36
found:	C: 59.50	H: 6.94	N: 21.47

Analytical data:

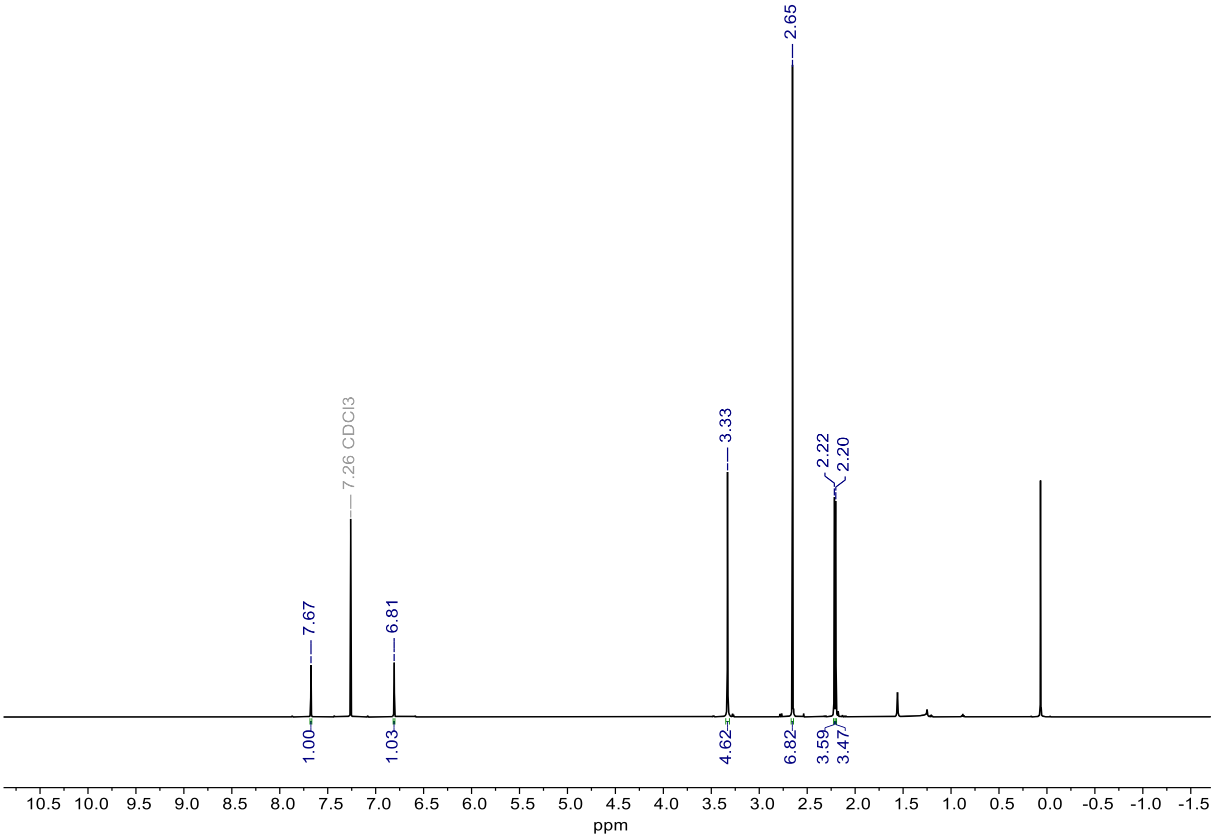

Figure S16: ^{1}H NMR spectrum (600 MHz, $CDCl_3$) of *N*-(4,5-dimethyl-2-nitrophenyl)-1,3-dimethylimidazolidin-2-imine. The impurities at 0.07 ppm and 1.21 ppm are due to grease, while the impurity at 1.55 ppm is due to water.

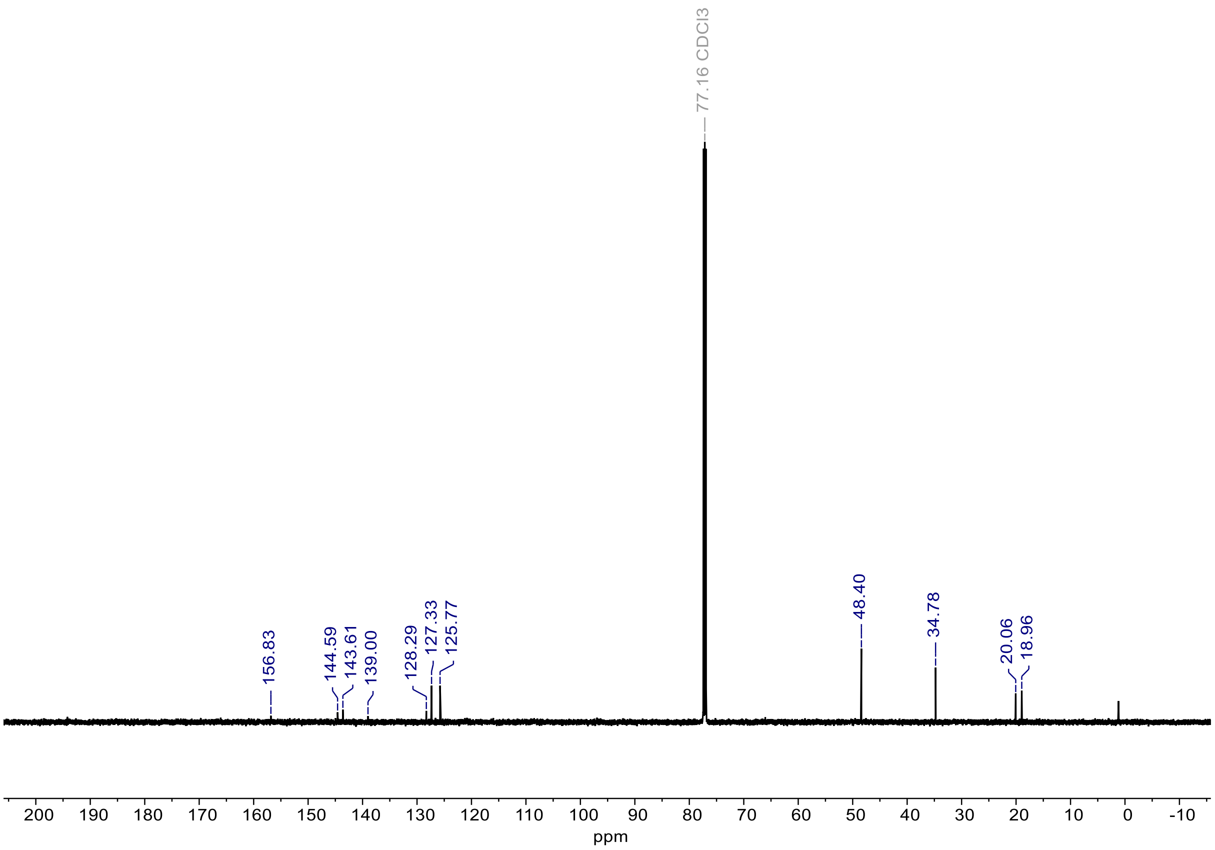

Figure S17: ^{13}C NMR spectrum (151 MHz, $CDCl_3$) of *N*-(4,5-dimethyl-2-nitrophenyl)-1,3-dimethylimidazolidin-2-imine.

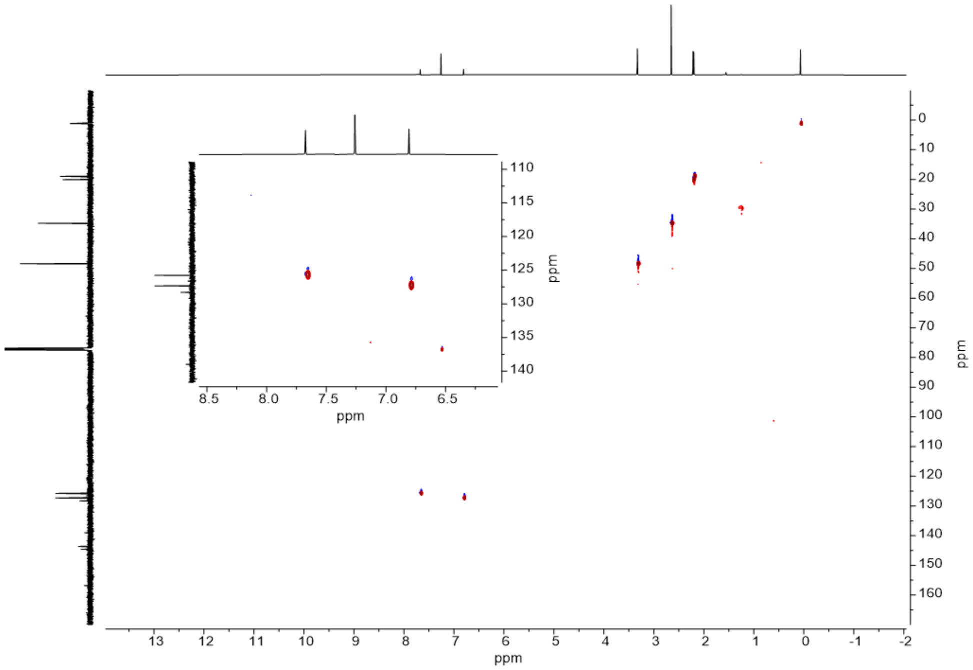

Figure S18: HSQC (^{1}H-^{13}C) spectrum of *N*-(4,5-dimethyl-2-nitrophenyl)-1,3-dimethylimidazolidin-2-imine.

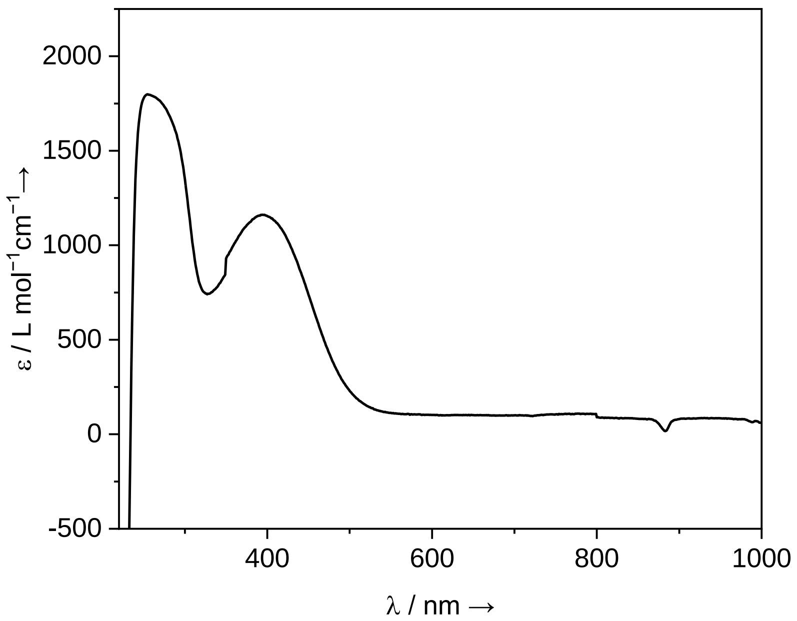

Figure S19: UV-Vis spectrum of *N*-(4,5-dimethyl-2-nitrophenyl)-1,3-dimethylimidazolidin-2-imine in DCM.

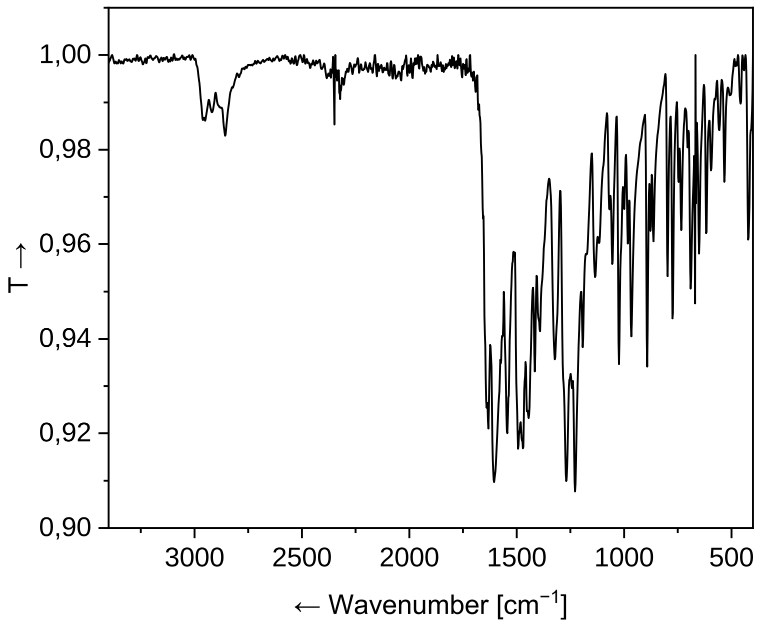

Figure S20: IR spectrum of *N*-(4,5-dimethyl-2-nitrophenyl)-1,3-dimethylimidazolidin-2-imine.

Table S4: Illustration of the structure of *N*-(4,5-dimethyl-2-nitrophenyl)-1,3-dimethylimidazolidin-2-imine in the solid state, as well as crystallographic data. H atoms have been omitted for clarity. Displacement ellipsoids correspond to 50% probability of residence. Colour coding: C dark-grey, N blue, O red.

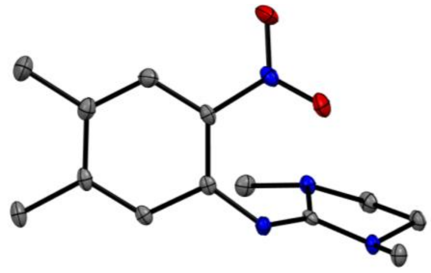

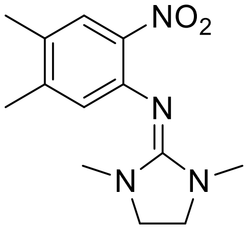

Empirical formula	$C_{13}H_{18}N_4O_2$
Formula weight	262.31
Temperature/K	100.00
Crystal system	triclinic
Space group	P-1
a/Å	6.8211(3)
b/Å	8.1858(4)
c/Å	11.8605(5)
α/°	99.429(2)
β/°	99.405(2)
γ/°	91.389(2)
Volume/Å^3	643.63(5)
Z	2
ρ_{calc}g/cm^3	1.354
μ/mm^{-1}	0.095
F(000)	280.0
Crystal size/mm^3	0.18 × 0.13 × 0.09
Radiation	MoKα (λ = 0.71073)
2Θ range for data collection/°	5.052 to 54.982
Index ranges	$-8 \le h \le 8$, $-10 \le k \le 10$, $-15 \le l \le 15$
Reflections collected	25076
Independent reflections	2952 [R_{int} = 0.0503, R_{sigma} = 0.0411]
Data/restraints/parameters	2952/0/176

Goodness-of-fit on F^2	1.038
Final R indexes [I>=2σ (I)]	R_1 = 0.0455, wR_2 = 0.1201
Final R indexes [all data]	R_1 = 0.0511, wR_2 = 0.1256
Largest diff. peak/hole / e $Å^{-3}$	0.48/-0.26

3.5 *N*-(4,5-dimethoxy-2-nitrophenyl)-1,3-dimethylimidazolidin-2-imine

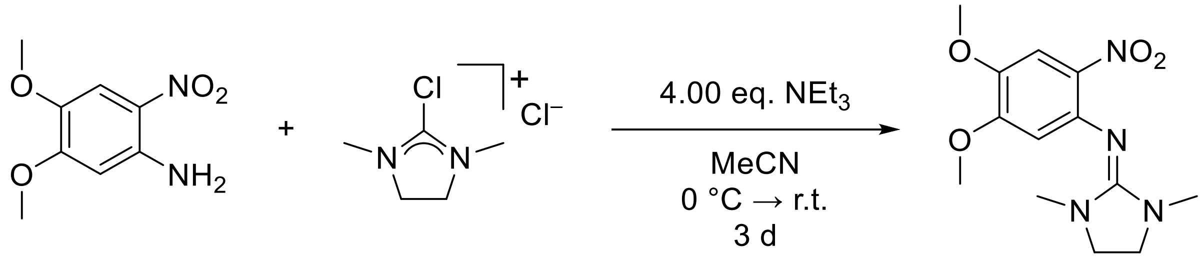

300 mg of 4,5-dimethoxy-2-nitroaniline (1.00 eq., 1.51 mmol) and 1.20 eq. activated urea (332 mg, 1.97 mmol) were dissolved in 5.00 mL acetonitrile under ice-cooling, and 843 µL triethylamine (612 mg, 4.00 eq., 6.06 mmol) was added. The reaction mixture was stirred overnight. The solution was filtered and the solvent was removed *in vacuo*. The residue was mixed with 4.00 mL of 15% degassed sodium hydroxide solution and extracted with 6 × 4.00 mL diethyl ether. The combined organic phases were dried over magnesium sulfate. The product was obtained as a red solid in 27% yield (122 mg, 414 µmol). Crystals were obtained after partial solvent removal under vacuum.

^{1}H NMR (600 MHz, $CDCl_3$): δ =7.54 (s, 1 H, CH_{arom}), 6.49 (s, 1 H, CH_{arom}), 3.90-3.88 (s, 6 H, 2xOCH_3), 3.37 (s, 4 H, 2xCH_2), 2.67 (s, 6 H, 2xCH_3) ppm.

^{13}C NMR (151 MHz, $CDCl_3$): δ =157.69 ($C_{q,Guan}$), 154.59 ($C_{q,arom}$), 144.09 ($C_{q,arom}$), 142.60 ($C_{q,arom}$), 132.36 ($C_{q,arom}$), 108.19 (CH$_{arom}$), 107.37 (CH$_{arom}$), 56.37 (OCH$_3$), 56.35 (OCH$_3$), 48.33 (NCH$_2$), 34.65 (NCH$_3$) ppm.

UV-Vis (DCM, c = 1.2115·10^{-4} mol·l^{-1}, d = 1 cm): λ_{max} (ε [l·mol^{-1}·cm^{-1}]) = 265 (2378), 413 (1550) nm.

IR (ATR): $\tilde{\nu}$ = 3009.01, 2955.29, 2851.46, 1607.55, 1553.65, 1494.20, 1477.79, 1454.99, 1436.03, 1397.62, 1342.27, 1312.46, 1276.14, 1233.71, 1207.63, 1178.72, 1028.52, 998.38, 967.29, 795.88, 680.33, 643.26, 604.01, 572.83, 438.47 cm^{-1}.

Elemental analysis (%):

calculated:	C: 53.05	H: 6.16	N: 19.04
found:	C: 52.89	H: 5.84	N: 18.78

Analytical data:

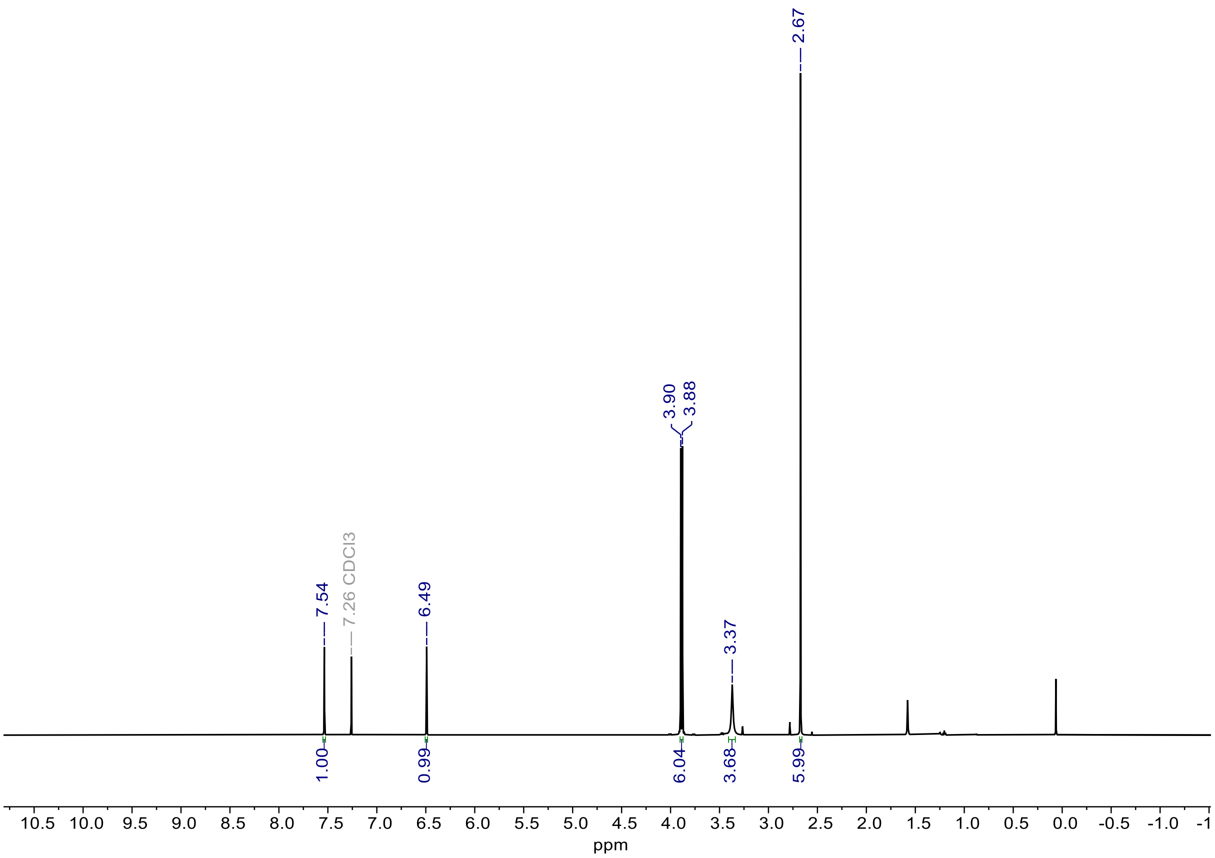

Figure S21: ^{1}H NMR spectrum (600 MHz, $CDCl_3$) of *N*-(4,5-dimethoxy-2-nitrophenyl)-1,3-dimethylimidazolidin-2-imine. The impurities at 0.07 ppm and 1.21 ppm are due to grease, while the impurity at 1.55 ppm is due to water.

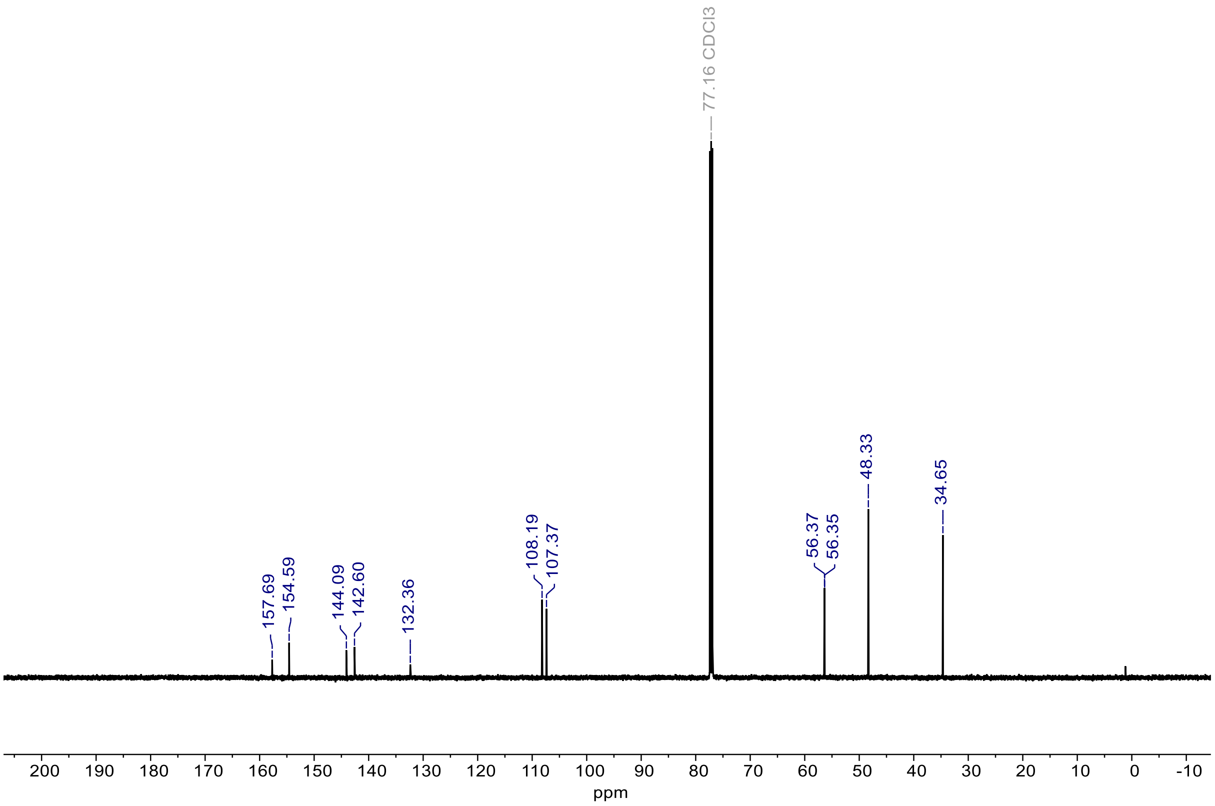

Figure S22: ^{13}C NMR spectrum (151 MHz, $CDCl_3$) of *N*-(4,5-dimethoxy-2-nitrophenyl)-1,3-dimethylimidazolidin-2-imine.

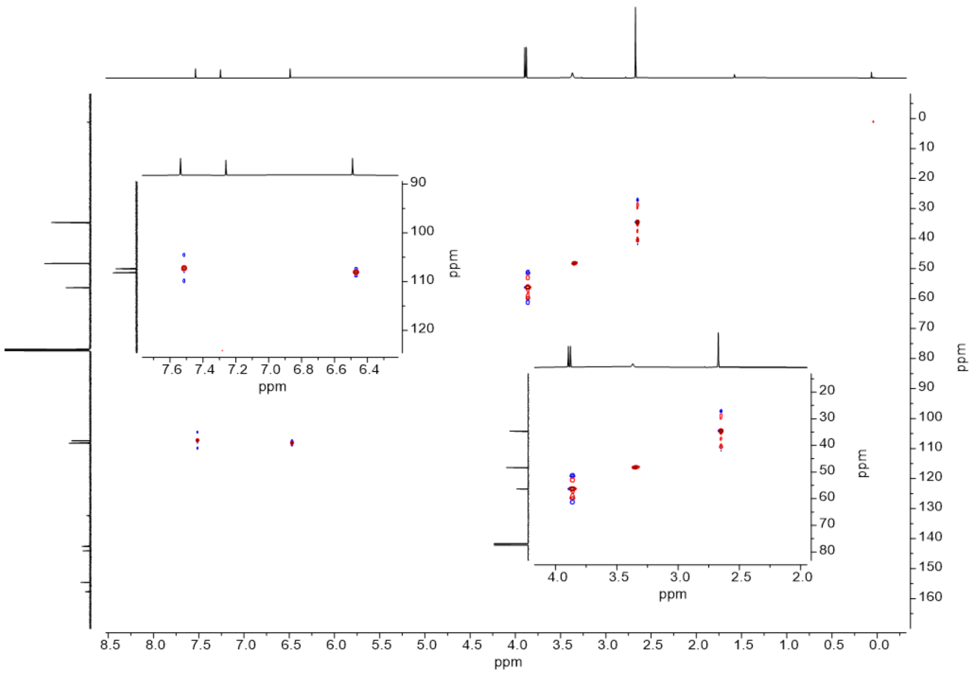

Figure S23: HSQC (^1H-^{13}C) spectrum of *N*-(4,5-dimethoxy-2-nitrophenyl)-1,3-dimethylimidazolidin-2-imine.

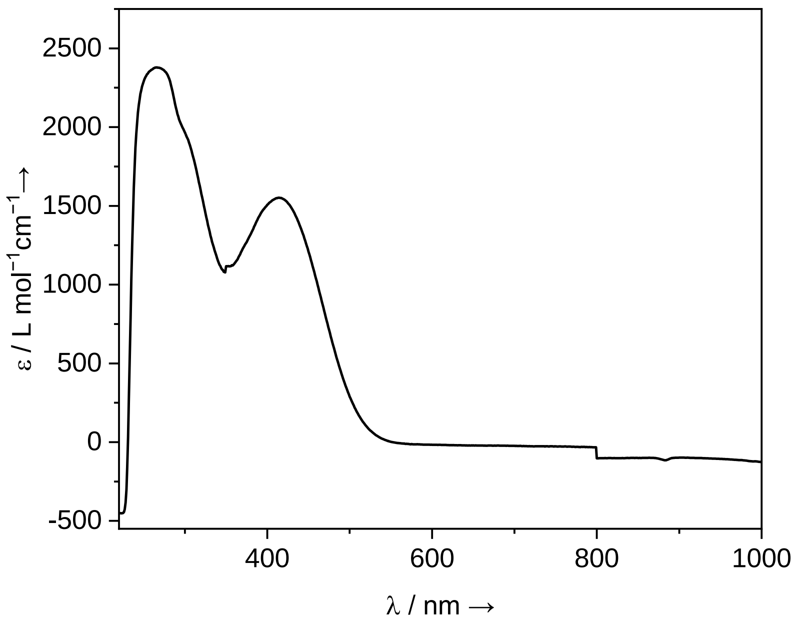

Figure S24: UV-Vis spectrum of *N*-(4,5-dimethoxy-2-nitrophenyl)-1,3-dimethylimidazolidin-2-imine in DCM.

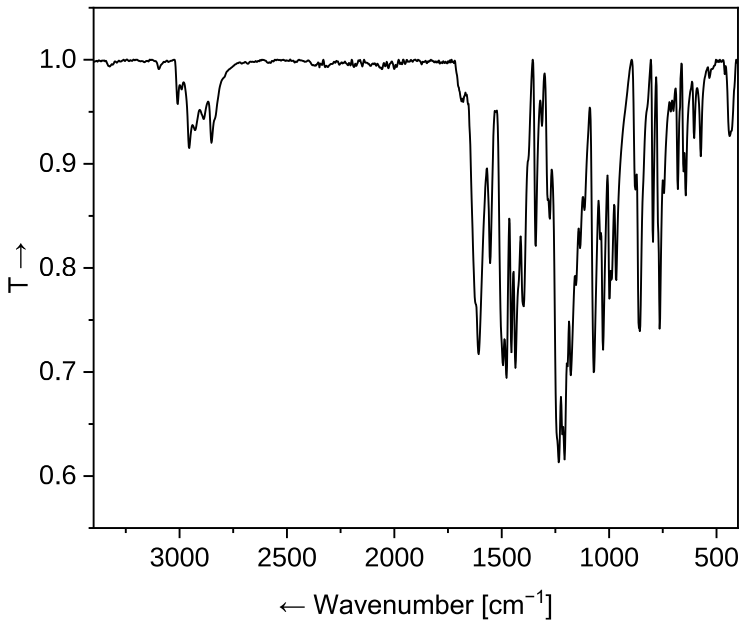

Figure S25: IR spectrum of *N*-(4,5-dimethoxy-2-nitrophenyl)-1,3-dimethylimidazolidin-2-imine.

Table S5: Illustration of the structure of *N*-(4,5-dimethoxy-2-nitrophenyl)-1,3-dimethylimidazolidin-2-imine in the solid state, together with crystallographic data. H atoms have been omitted for clarity. Displacement ellipsoids correspond to 50% probability of residence. Colour coding: C dark-grey, N blue, O red.

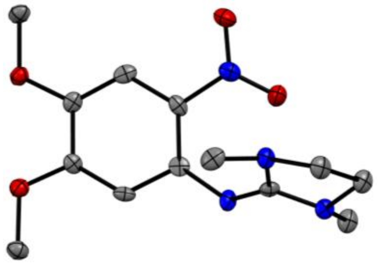

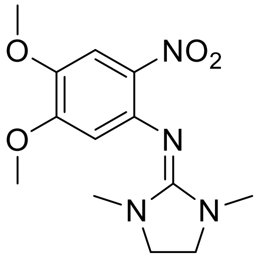

Empirical formula	$C_{13}H_{18}N_4O_4$
Formula weight	294.31
Temperature/K	100.00
Crystal system	triclinic
Space group	P-1
a/Å	6.7038(14)
b/Å	8.8038(17)
c/Å	11.771(2)
α/°	84.144(9)
β/°	81.701(9)
γ/°	89.968(9)
Volume/Å^3	683.8(2)
Z	2
ρ_{calc}g/cm^3	1.429
μ/mm^{-1}	0.108
F(000)	312.0
Crystal size/mm^3	0.37 × 0.2 × 0.126
Radiation	MoKα (λ = 0.71073)

2Θ range for data collection/°	4.652 to 51.99
Index ranges	-8 ≤ h ≤ 8, -10 ≤ k ≤ 10, -14 ≤ l ≤ 14
Reflections collected	17500
Independent reflections	17500 [R_{int} = ?, R_{sigma} = 0.1831]
Data/restraints/parameters	17500/0/196
Goodness-of-fit on F^2	1.081
Final R indexes [I>=2σ (I)]	R_1 = 0.0911, wR_2 = 0.2051
Final R indexes [all data]	R_1 = 0.1684, wR_2 = 0.2433
Largest diff. peak/hole / e $Å^{-3}$	0.51/-0.62

4 Reductions

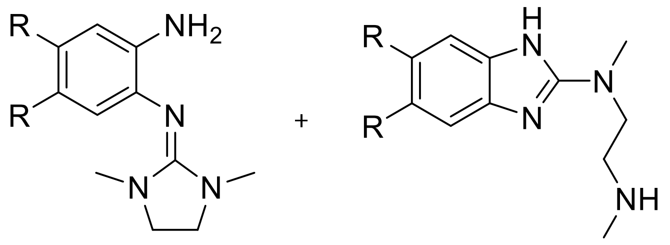

R = H, F, Cl, Me, OMe

Scheme S2: Overview of the *o*-guanidino-amino-benzenes with -H, -F, -Cl, -Me, and -OMe in the backbone.

4.1 Reduction of 1,3-dimethyl-*N*-(2-nitrophenyl)imidazolidin-2-imine

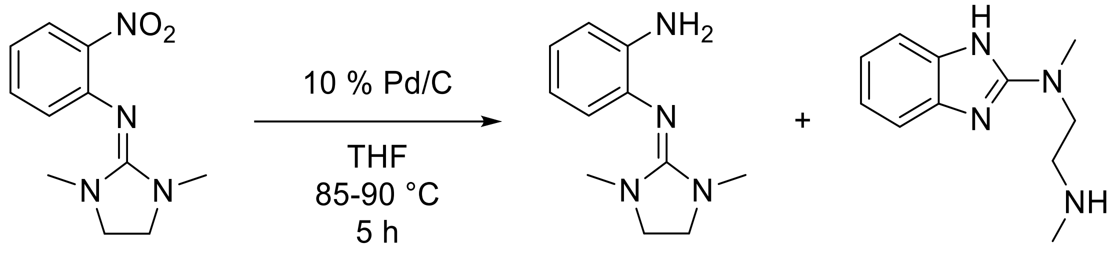

1,3-Dimethyl-*N*-(2-nitrophenyl)imidazolidin-2-imine (200 mg, 1.00 eq., 853 µmol) and 10% palladium on activated charcoal (90.9 mg, 10 mol%, 85.4 µmol) were suspended in 10.00 mL THF. Then, 416 ml of hydrazine monohydrate (427 mg, 10.0 eq., 8.54 mmol) was slowly added to the yellow solution and refluxed at 85-90 °C for 5 h. The Colourless solution was separated from the palladium and the solvent was removed in a fine vacuum. The product was obtained as a colourless viscous liquid in quantitative yield (174 mg, 853 µmol).

2-((1,3-dimethylimidazolidin-2-ylidene)amino)aniline:

^{1}H NMR (400 MHz, $CDCl_3$): δ [ppm] = 6.76 – 6.60 (m, 4 H, CH_{arom}), 3.26 (s, 4 H, 2xCH_2), 2.65 (s, 6H, 2xCH_3).

^{13}C NMR (101 MHz, $CDCl_3$): δ = 157.98 ($C_{q,Guan}$), 139.44 ($C_{q,arom}$), 136.66 ($C_{q,arom}$), 122.24 (CH_{arom}), 121.52 (CH_{arom}), 118.50 (CH_{arom}), 114.66 (CH_{arom}), 48.74 (NCH_2), 35.12 (NCH_3) ppm.

N^1-(1*H*-benzo[*d*]imidazol-2-yl)-*N*^{1},*N*^{2}-dimethylethane-1,2-diamine (rearranged species):

^{1}H NMR (400 MHz, $CDCl_3$): δ [ppm] = 7.29 (dd, J = 5.9, 3.2 Hz, 2 H, CH_{arom}), 7.01 (dd, J = 5.8, 3.2 Hz, 2 H, CH_{arom}), 3.47 – 3.42 (m, 2 H, CH_2), 3.21 (s, 3 H, CH_3), 2.92 – 2.86 (m, 2 H, 2xCH_2), 2.49 (s, 3 H, CH_3).

^{13}C NMR (101 MHz, $CDCl_3$): δ = 156.32 (C_q), 120.15 (CH_{arom}), 112.05 (CH_{arom}), 52.85 (NCH_2), 51.58 (NCH_2), 38.43 (NCH_3), 36.46 (NCH_3) ppm. Some signals are missing in the ^{13}C NMR spectrum due to low intensity or overlapping.

Analytical data:

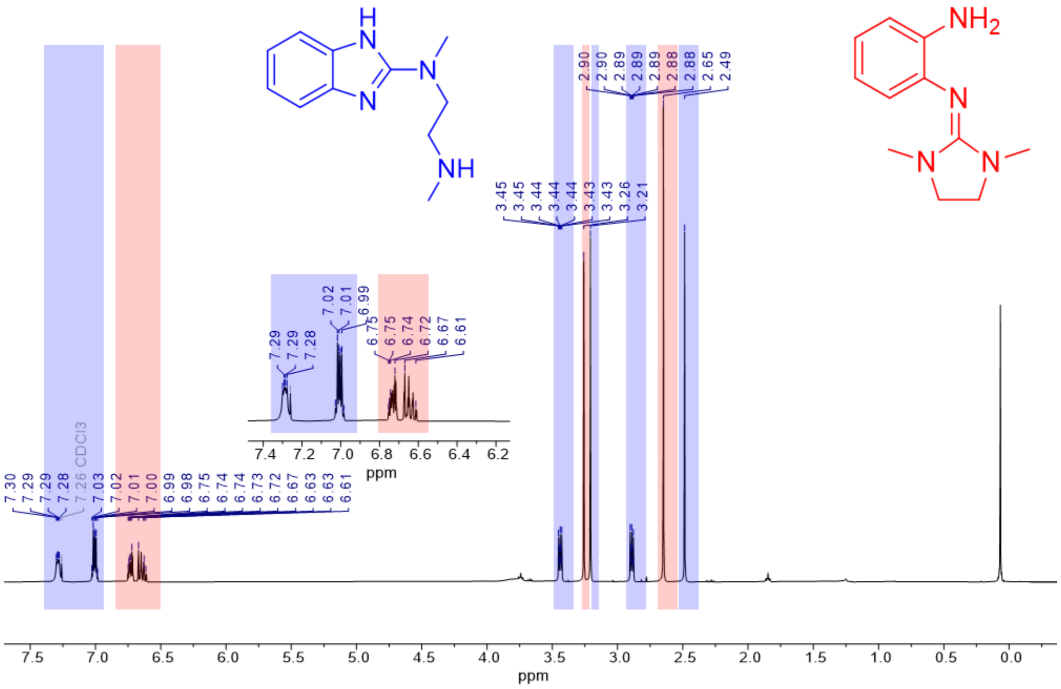

Figure S26: ^{1}H NMR spectrum (400 MHz, $CDCl_3$) of the two species 2-((1,3-dimethylimidazolidin-2-ylidene)amino)aniline (red) and *N*[1]-(1*H*-benzo[*d*]imidazol-2-yl)-*N*[1],*N*[2]-dimethylethane-1,2-diamine (blue). The impurities at 0.07 ppm and 1.21 ppm are due to grease.

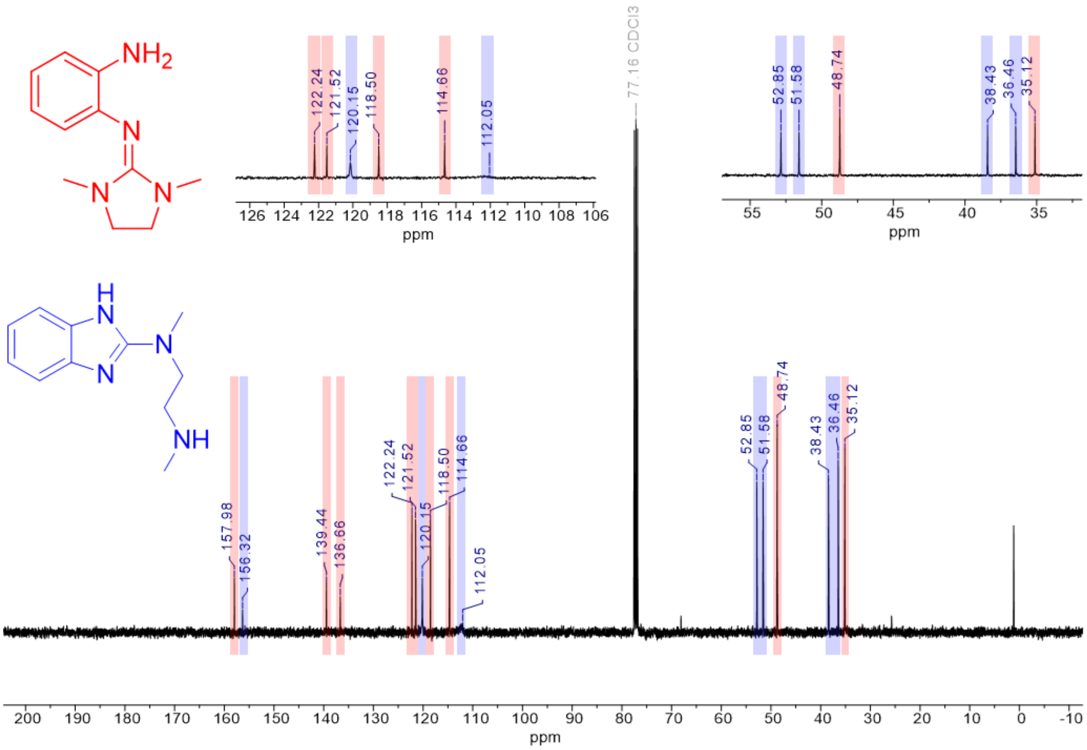

Figure S27: ^{13}C NMR spectrum (101 MHz, $CDCl_3$) of 2-((1,3-dimethylimidazolidin-2-ylidene)amino)aniline (red) and *N*[1]-(1*H*-benzo[*d*]imidazol-2-yl)-*N*[1],*N*[2]-dimethylethane-1,2-diamine (blue). The impurity at 1.15 ppm is due to grease, while the impurities at 68.10 ppm and 25.73 ppm are due to tetrahydrofuran.

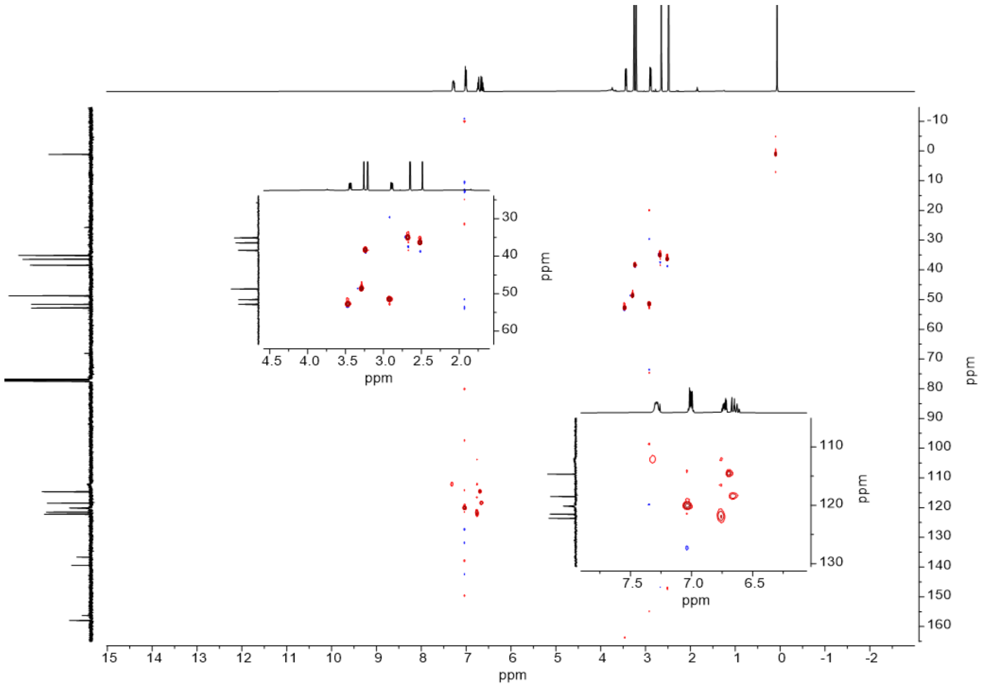

Figure S28: HSQC (^{1}H-^{13}C) spectrum of the two species 2-((1,3-dimethylimidazolidin-2-ylidene)amino)aniline and *N*^{1}-(1*H*-benzo[*d*]imidazol-2-yl)-*N*^{1},*N*^{2}-dimethylethane-1,2-diamine.

4.2 Reduction of *N*-(4,5-difluoro-2-nitrophenyl)-1,3-dimethylimidazolidin-2-imine

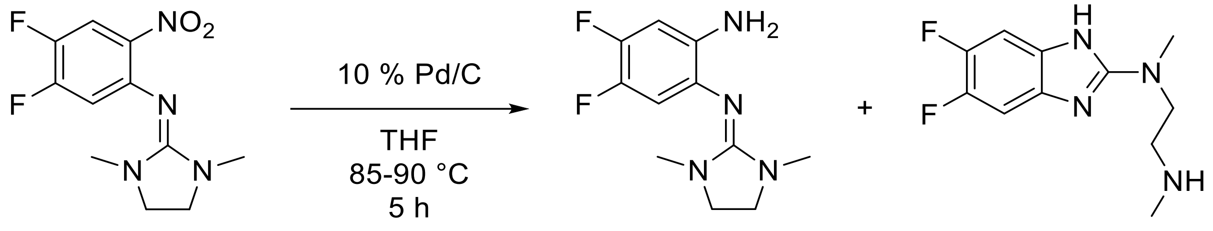

N-(4,5-Difluoro-2-nitrophenyl)-1,3-dimethylimidazolidin-2-imine (250 mg, 1.00 eq., 925 µmol) and 10 %palladium on activated charcoal (98.5 mg, 10 mol%, 92.5 µmol) were suspended in 10.00 mL THF. 450 µL hydrazine monohydrate (463 mg, 10.0 eq., 9.25 mmol) was slowly added to the yellow solution and refluxed at 85-90 °C for 5 h. The brownish solution was separated from the palladium and the solvent was removed in a fine vacuum. The product was obtained as a yellow viscous liquid in quantitative yield (222 mg, 925 µmol).

2-((1,3-dimethylimidazolidin-2-ylidene)amino)-4,5-difluoroaniline:

^{1}H NMR (600 MHz, $CDCl_3$): δ =6.54 (dd, *J* = 11.9, 8.2 Hz, 1 H, CH_{arom}), 6.44 (dd, *J* = 11.9, 8.0 Hz, 1 H, CH_{arom}), 3.75 (s, 2 H, NH_2) 3.28 (d, *J* = 0.8 Hz, 4 H, 2xCH_2), 2.65 (d, *J* = 0.8 Hz, 6 H, 2xCH_3) ppm.

^{13}C NMR (151 MHz, $CDCl_3$): δ =157.12 ($C_{q,Guan}$), 145.93 (d, *J* = 13.5 Hz, $C_{q,arom}$), 144.08 (dd, *J* = 84.3, 13.4 Hz, $C_{q,arom}$), 142.23 (d, *J* = 13.3 Hz, $C_{q,arom}$), 135.65 (dd, *J* = 8.0, 2.0 Hz, $C_{q,arom}$), 132.39 (dd, *J* = 6.9, 2.8 Hz, $C_{q,arom}$), 109.99 (dd, *J* = 17.8, 1.0 Hz, CH_{arom}), 102.58 (d, *J* = 20.6 Hz, CH_{arom}), 48.67(NCH_2), 35.07 (NCH_3) ppm.

^{19}F NMR (565 MHz, $CDCl_3$): δ = -148.34 (ddd, *J* = 23.2, 11.8, 7.8 Hz, 1 F), -152.02 – -152.12 (m, 1 F) ppm.

N[1]-(5,6-difluoro-1*H*-benzo[*d*]imidazol-2-yl)-*N*[1],*N*[2]-dimethylethane-1,2-diamine (rearranged species):

^{1}H NMR (600 MHz, $CDCl_3$): δ =7.04 (t, *J* = 9.1 Hz, 2 H, CH_{arom}), 3.43 – 3.40 (m, 2 H, CH_2), 3.19 (s, 3 H, CH_3), 2.94 – 2.89 (m, 2 H, CH_2), 2.50 (s, 3 H, CH_3) ppm.

^{13}C NMR (151 MHz, $CDCl_3$): δ = 159.26 ($C_{q,Guan}$), 52.83 (NCH_2), 51.68 (NCH_2), 38.60 (NCH_3), 36.29 (NCH_3) ppm. Due to low concentration or overlapping, signals are missing in the ^{13}C NMR.

^{19}F NMR (565 MHz, $CDCl_3$): δ = -146.06 (t, J = 8.1 Hz, 1 F), -149.63 (t, J = 9.5 Hz) ppm.

Analytical data:

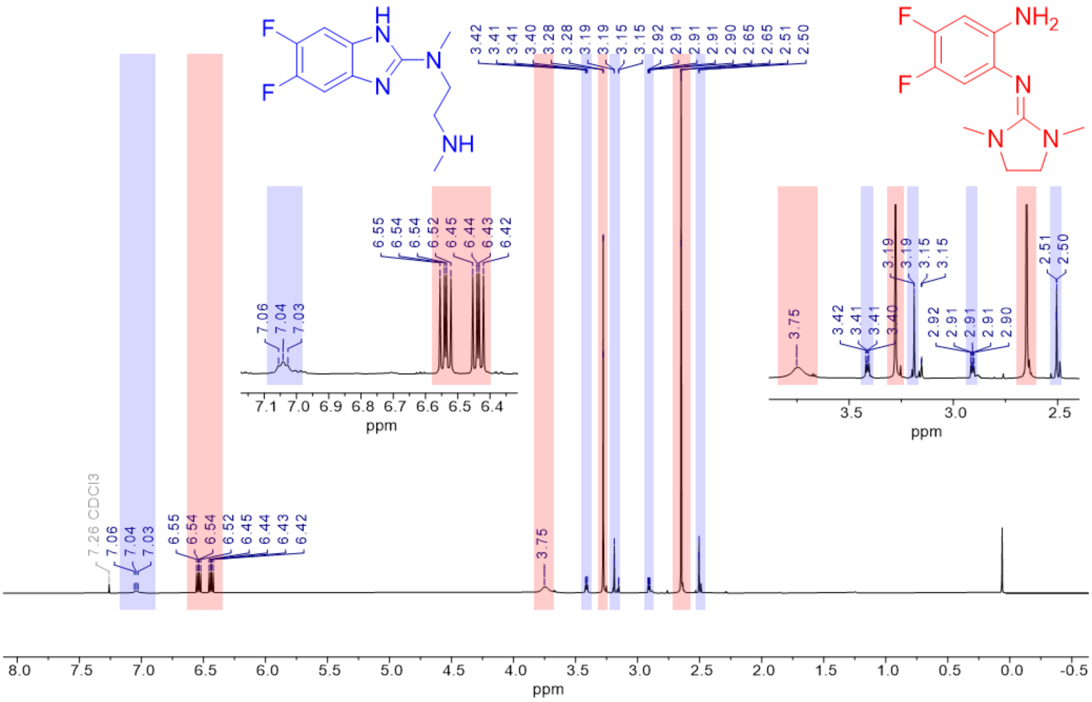

Figure S29: ^{1}H NMR spectrum (600 MHz, $CDCl_3$) of the two species 2-((1,3-dimethylimidazolidin-2-ylidene)amino)-4,5-difluoroaniline (red) and *N*[1]-(5,6-difluoro-1*H*-benzo[*d*]imidazol-2-yl)-*N*[1],*N*[2]-dimethylethane-1,2-diamine (blue). The impurity at 0.07 ppm is due to grease.

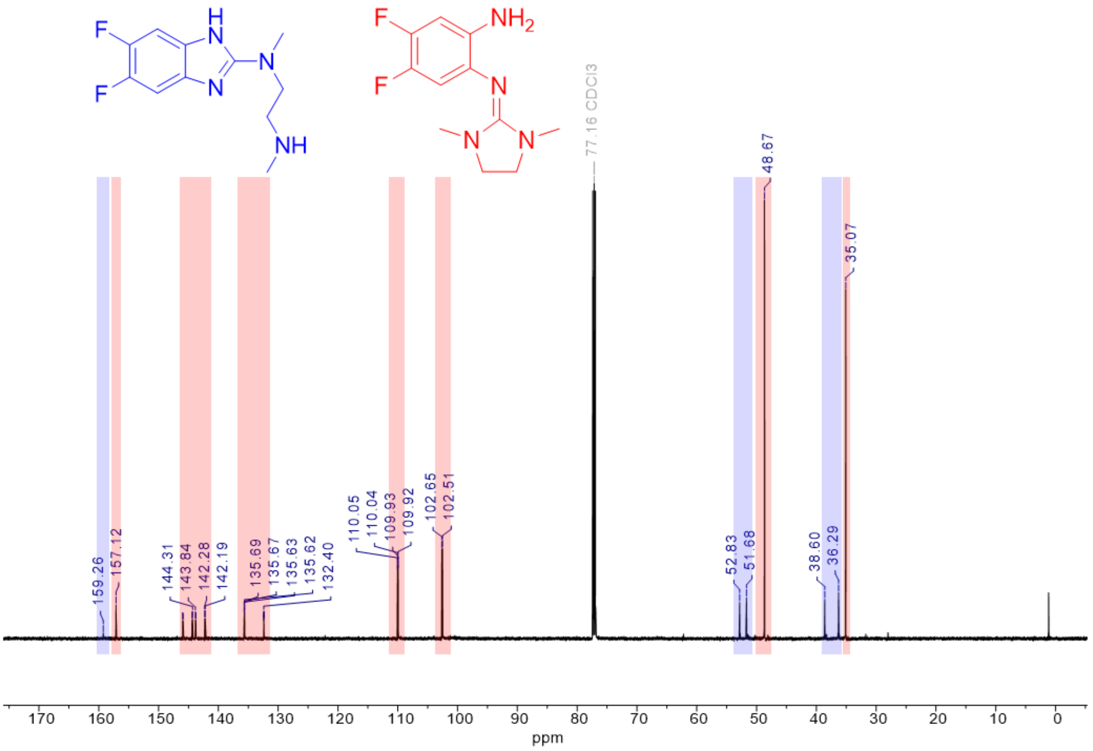

Figure S30: ^{13}C NMR spectrum (151 MHz, $CDCl_3$) of the two species 2-((1,3-dimethylimidazolidin-2-ylidene)amino)-4,5-difluoroaniline (red) and *N*[1]-(5,6-difluoro-1*H*-benzo[*d*]imidazol-2-yl)-*N*[1],*N*[2]-dimethylethane-1,2-diamine (blue). The impurity at 1.15 ppm is due to grease, while the impurities at 68.10 ppm and 25.73 ppm are due to tetrahydrofuran.

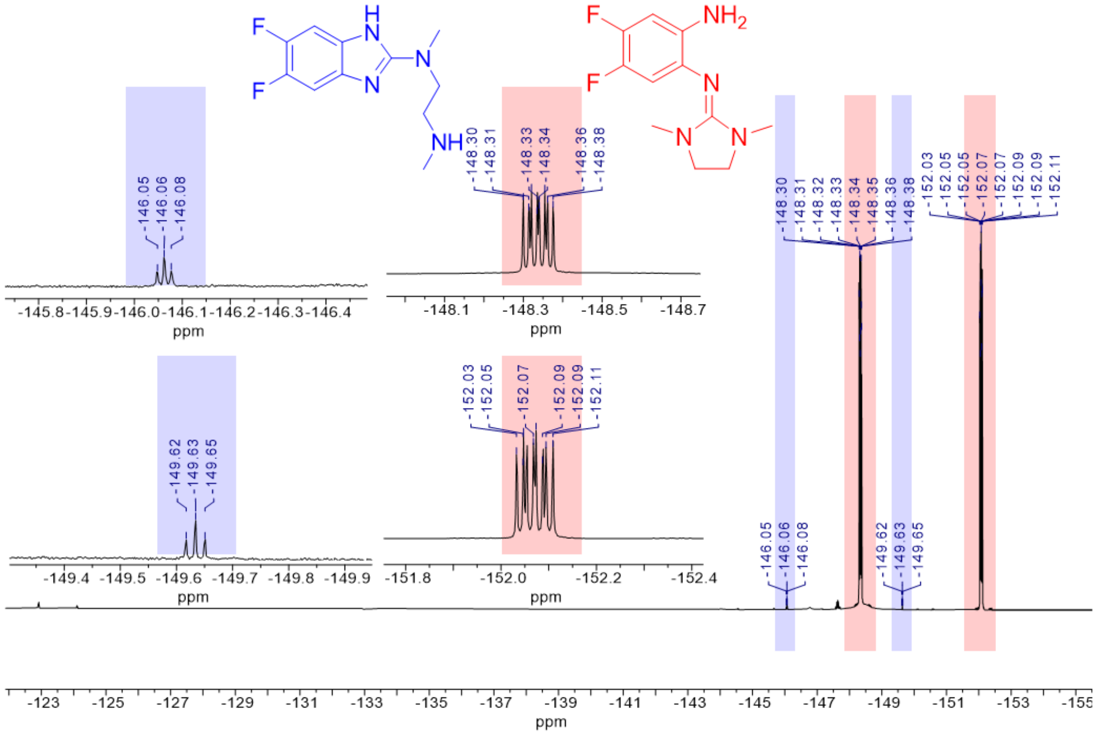

Figure S31: ^{19}F NMR spectrum (565 MHz, $CDCl_3$) of the two species 2-((1,3-dimethylimidazolidin-2-ylidene)amino)-4,5-difluoroaniline (red) and *N*[1]-(5,6-difluoro-1*H*-benzo[*d*]imidazol-2-yl)-*N*[1],*N*[2]-dimethylethane-1,2-diamine (blue). The impurities at -128.20 ppm and -147.13 ppm are *N*-(4,5-difluoro-2-nitrophenyl)-1,3-dimethylimidazolidin-2-imine.

4.3 Reduction of *N*-(4,5-dichloro-2-nitrophenyl)-1,3-dimethylimidazolidin-2-imine

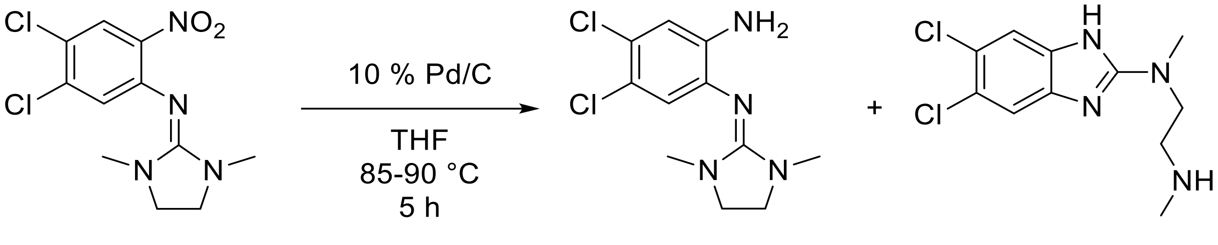

N-(4,5-dichloro-2-nitrophenyl)-1,3-dimethylimidazolidin-2-imine (250 mg, 1.00 eq., 824 µmol) and 10 %palladium on activated charcoal (87.8 mg, 10 mol%, 82.4 µmol) were suspended in 10.00 mL THF. 402 µL Hydrazine monohydrate (412 mg, 10.0 eq., 8.25 mmol) was slowly added to the yellow solution and refluxed at 85-90 °C for 5 h. The yellowish solution was separated from the palladium and the solvent was removed under fine vacuum. The product was obtained as a yellow viscous liquid in 93% yield (209 mg, 765 µmol).

4,5-dichloro-2-((1,3-dimethylimidazolidin-2-ylidene)amino)aniline:

^{1}H NMR (400 MHz, $CDCl_3$): δ = 6.75 (s, 1 H, CH_{arom}), 6.69 (s, 1 H, CH_{arom}), 3.89 (s, 2 H, NH_2) 3.29 (s, 4 H, 2xCH_2), 2.65 (s, 6 H, 2xCH_3) ppm.

^{13}C NMR (101 MHz, $CDCl_3$): δ = 157.30 ($C_{q,Guan}$), 139.55 ($C_{q,arom}$), 136.67 ($C_{q,arom}$), 123.11 ($C_{q,arom}$), 122.33 (CH_{arom}), 120.03 ($C_{q,arom}$), 114.83 (CH_{arom}), 48.62 (NCH_2), 35.09 (NCH_3) ppm.

N^1-(5,6-dichloro-1*H*-benzo[*d*]imidazol-2-yl)-*N*^{1},*N*^{2}-dimethylethane-1,2-diamine (rearranged species):

^{1}H NMR (400 MHz, $CDCl_3$): δ = 7.30 (s, 1 H, N*H*), 7.29 – 7.26 (m, 1 H, CH_{arom}), 7.00 (dd, *J* = 5.8, 3.2 Hz, 1 H, CH_{arom}), 3.46 – 3.38 (m, 2 H, CH_2), 3.19 (d, *J* = 2.7 Hz, 3 H, CH_3), 2.94 – 2.88 (m, 2 H, CH_2), 2.50 (d, *J* = 3.0 Hz, 3 H, CH_3) ppm.

Analytical data:

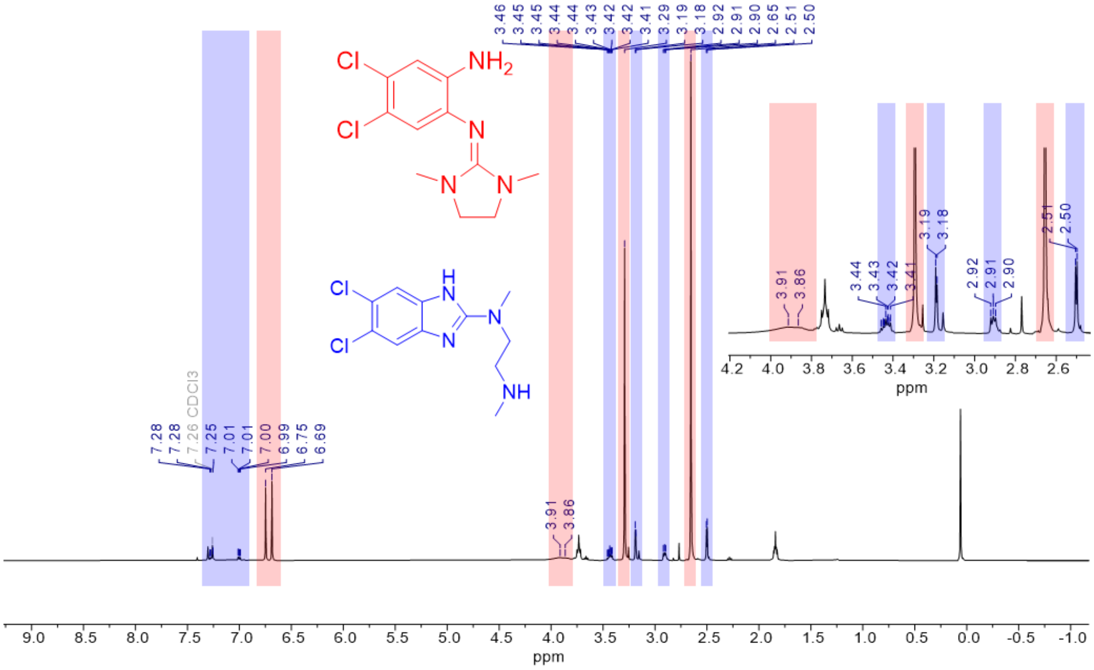

Figure S32: ^1H NMR spectrum (400 MHz, $CDCl_3$) of the two species 4,5-dichloro-2-((1,3-dimethylimidazolidin-2-ylidene)amino)aniline (red) and *N*[1]-(5,6-dichloro-1*H*-benzo[*d*]imidazol-2-yl)-*N*[1],*N*[2]-dimethylethane-1,2-diamine (blue). The impurity at 0.06 ppm is due to grease, while the impurities at 3.73 ppm and 1.85 ppm are due to tetrahydrofuran.

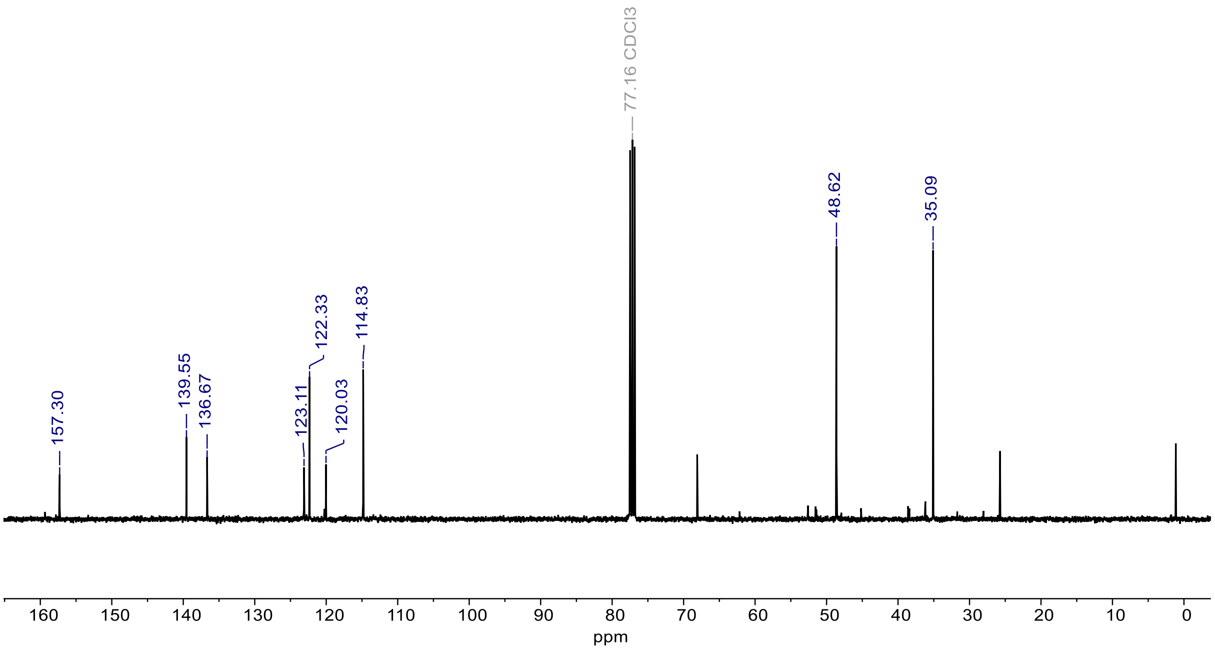

Figure S33: ^{13}C NMR spectrum (101 MHz, $CDCl_3$) of 4,5-dichloro-2-((1,3-dimethylimidazolidin-2-ylidene)amino)aniline. The impurity at 1.15 ppm is due to grease, while the impurities at 68.10 ppm and 25.73 ppm are due to tetrahydrofuran.

4.4 Reduction of *N*-(4,5-dimethyl-2-nitrophenyl)-1,3-dimethylimidazolidin-2-imine

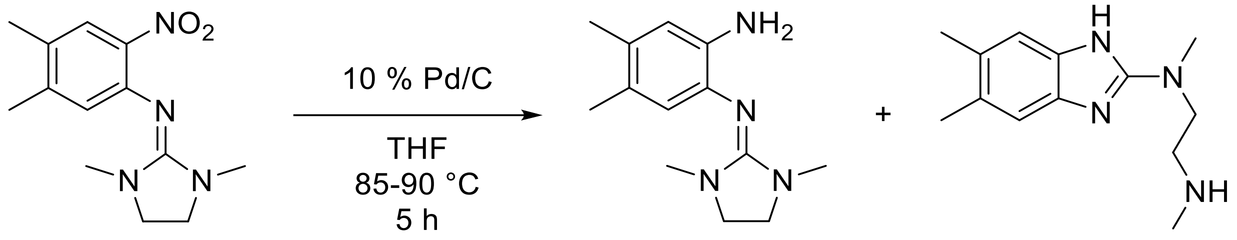

N-(4,5-dimethyl-2-nitrophenyl)-1,3-dimethylimidazolidin-2-imine (500 mg, 1.00 eq., 1.91 mmol) and 10% palladium on activated charcoal (203 mg, 10 mol%, 190 µmol) were suspended in 10.00 mL THF. 929 µL hydrazine monohydrate (954 mg, 10.0 eq., 19.1 mmol) was slowly added to the yellow solution and refluxed at 85-90 °C for 5 h. The brownish solution was separated from the palladium and the solvent was removed in a fine vacuum. The product was obtained as a slightly brownish solid in quantitative yield (443 mg, 1.91 µmol).

2-((1,3-dimethylimidazolidin-2-ylidene)amino)-4,5-dimethylaniline:

^{1}H NMR (600 MHz, $CDCl_3$): δ = 6.53 (s, 1 H, C*H$_{arom}$*), 6.47 (s, 1 H, C*H$_{arom}$*), 3.59 (s, 2 H, N*H*$_2$) 3.24 (s, 4 H, 2xC*H*$_2$), 2.65 (s, 6 H, 2xC*H*$_3$), 2.12 – 2.09 (m, 6 H, 2xC*H*$_{3,arom}$) ppm.

^{13}C NMR (151 MHz, $CDCl_3$): δ = 123.58 (*C*H$_{arom}$), 116.57 (*C*H$_{arom}$), 48.81 (N*C*H$_2$), 35.23 (N*C*H$_3$), 19.33 (*C*H$_{3,arom}$), 19.09 (*C*H$_{3,arom}$).

N^1-(5,6-dimethyl-1*H*-benzo[*d*]imidazol-2-yl)-*N*^{1},*N*^{2}-dimethylethane-1,2-diamine (rearranged species):

^{1}H NMR (600 MHz, $CDCl_3$): δ = 7.08 (s, 2 H, C*H$_{arom}$*), 3.45 – 3.40 (m, 2 H, C*H*$_2$), 3.18 (s, 3 H, C*H*$_3$), 2.90 – 2.87 (m, 2 H, C*H*$_2$), 2.48 (s, 3 H, C*H*$_3$), 2.29 (s, 6 H, 2×C*H*$_{3,arom}$).

^{13}C NMR (151 MHz, $CDCl_3$): δ = 52.93 (N*C*H$_2$), 51.58 (N*C*H$_2$), 38.37 (N*C*H$_3$), 36.54 (N*C*H$_3$), 20.28 (*C*H$_{3,arom}$) ppm.

Peaks without clear assignment:

^{13}C NMR (151 MHz, $CDCl_3$): δ : 157.62 , 156.05, 136.76, 134.17, 129.16, 126.17, 118.65 ppm.

Analytical data:

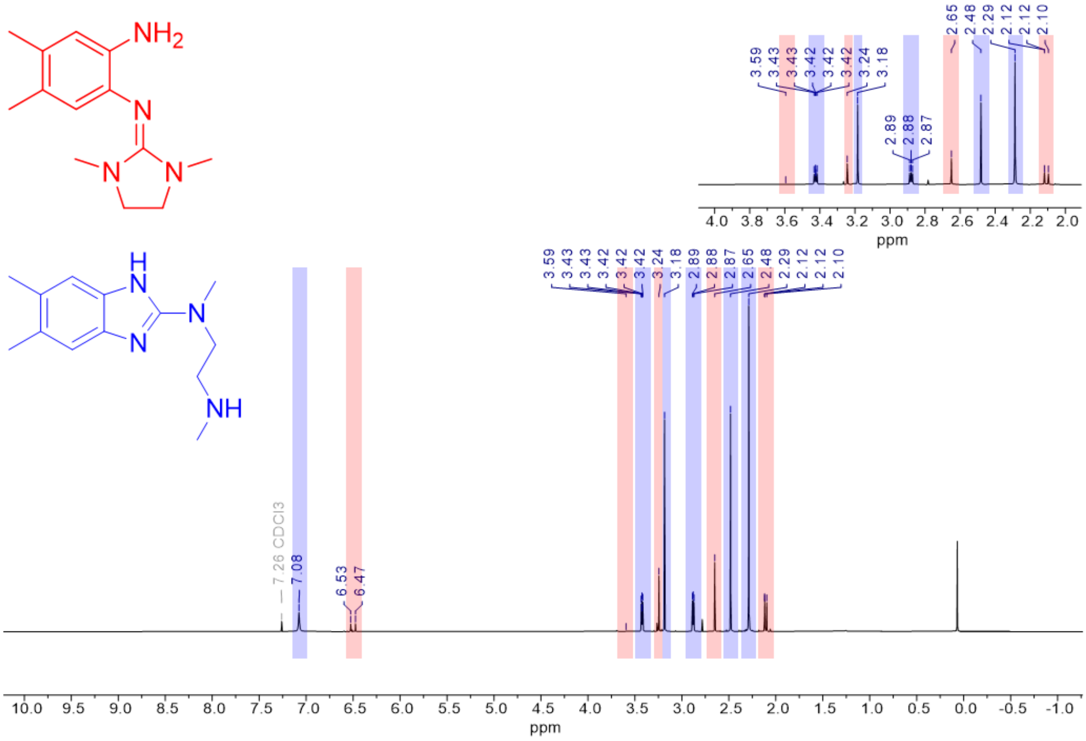

Figure S34: ^{1}H NMR spectrum (600 MHz, $CDCl_3$) of the two species 2-((1,3-dimethylimidazolidin-2-ylidene)amino)-4,5-dimethylaniline (red) and *N*[1]-(5,6-dimethyl-1*H*-benzo[*d*]imidazol-2-yl)-*N*[1],*N*[2]-dimethylethane-1,2-diamine (blue). The impurity at 0.07 ppm is due to grease.

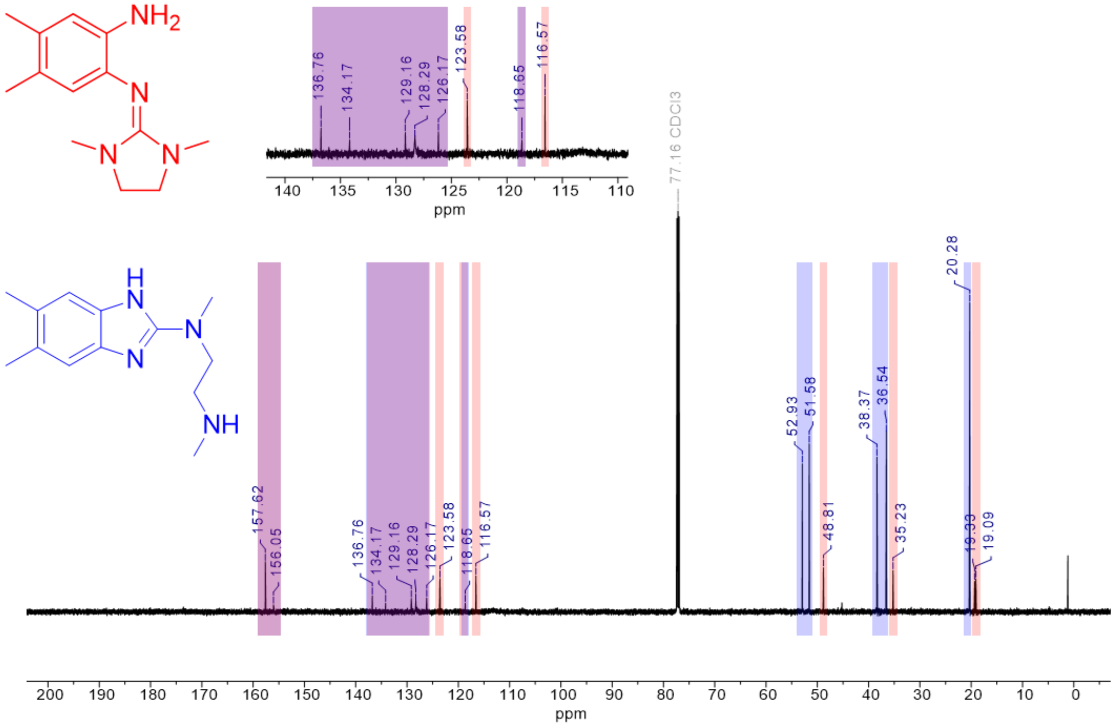

Figure S35: ^{13}C NMR spectrum (151 MHz, $CDCl_3$) of) of the two species 2-((1,3-dimethylimidazolidin-2-ylidene)amino)-4,5-dimethylaniline (red) and *N*[1]-(5,6-dimethyl-1*H*-benzo[*d*]imidazol-2-yl)-*N*[1],*N*[2]-dimethylethane-1,2-diamine (blue). Peaks without clear assignment are characterised by violet boxes. The impurity at 1.17 ppm is due to grease.

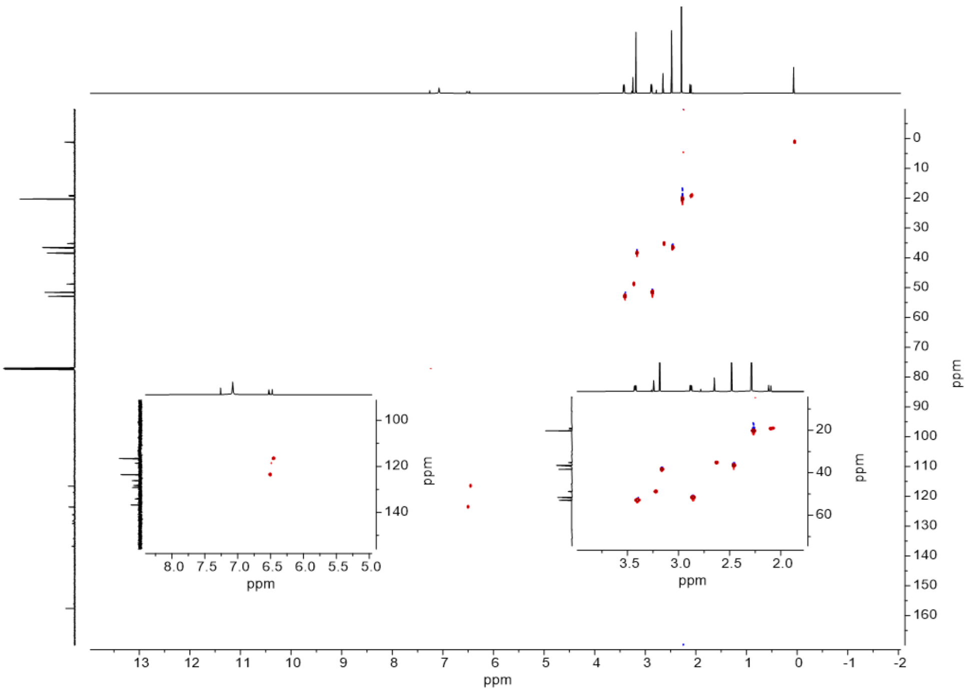

Figure S36: HSQC (^{1}H-^{13}C) spectrum of the two species 2-((1,3-dimethylimidazolidin-2-ylidene)amino)-4,5-dimethylaniline and *N*¹-(5,6-dimethyl-1*H*-benzo[*d*]imidazol-2-yl)-*N*¹,*N*²-dimethylethane-1,2-diamine.

4.5 Reduction of *N*-(4,5-dimethoxy-2-nitrophenyl)-1,3-dimethylimidazolidin-2-imine

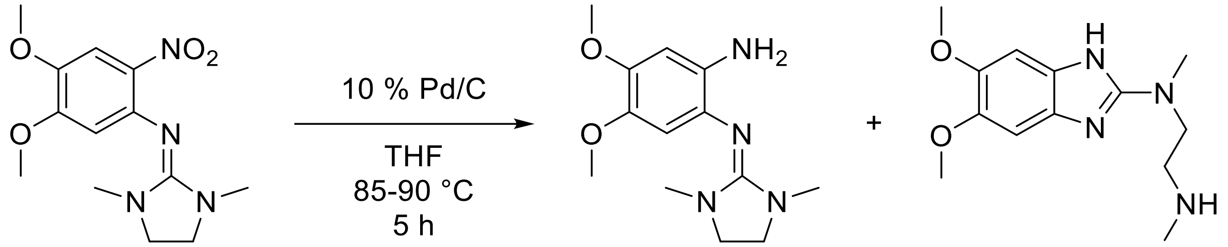

N-(4,5-dimethoxy-2-nitrophenyl)-1,3-dimethylimidazolidin-2-imine (250 mg, 1.00 eq., 849 µmol) and 10% palladium on activated charcoal (84.9 mg, 10 mol%, 84.9 µmol) were suspended in 10.00 mL THF. 414 µL hydrazine monohydrate (425 mg, 10.0 eq., 8.49 mmol) was slowly added to the yellow solution and refluxed at 85-90 °C for 5 h. The brownish solution was separated from the palladium and the solvent was removed in a fine vacuum. The product was obtained as a slightly brownish viscous solution in quantitative yield (224 mg, 849 µmol).

2-((1,3-dimethylimidazolidin-2-ylidene)amino)-4,5-dimethoxyaniline:

^{1}H NMR (400 MHz, $CDCl_3$): δ = 6.40 (s, 1 H, C*H*$_{arom}$), 6.30 (s, 1 H, C*H*$_{arom}$), 3.75 (d, *J* = 8.4 Hz, 6 H, 2xOC*H*$_3$), 3.23 (s, 4 H, 2×C*H*$_2$), 2.63 (s, 6 H, 2×C*H*$_3$).

^{13}C NMR (101 MHz, $CDCl_3$): δ = 108.12 (*C*H$_{arom}$), 100.80 (*C*H$_{arom}$), 56.21 (O*C*H$_3$), 48.72 (N*C*H$_2$), 35.05 (N*C*H$_3$).

N^1-(5,6-dimethoxy-1*H*-benzo[*d*]imidazol-2-yl)-*N*^{1},*N*^{2}-dimethylethane-1,2-diamine (rearranged species):

^{1}H NMR (400 MHz, $CDCl_3$): δ = 6.88 (s, 2 H, C*H*$_{arom}$), 3.83 (s, 6 H, 2xOC*H*$_3$), 3.43 – 3.34 (m, 2 H, C*H*$_2$), 3.14 (s, 3 H, C*H*$_3$), 2.88 – 2.80 (m, 2 H, C*H*$_2$), 2.46 (s, 3 H, C*H*$_3$).

^{13}C NMR (101 MHz, $CDCl_3$): δ = 56.69 (O*C*H$_3$), 52.79 (N*C*H$_2$), 51.52 (N*C*H$_2$), 38.36 (N*C*H$_3$), 36.45 (N*C*H$_3$).

Peaks without clear assignment:

^{13}C NMR (101 MHz, $CDCl_3$): δ = 157.49, 156.26, 144.57, 144.16, 141.79, 132.23, 129.04, 103.75 ppm.

Analytical data:

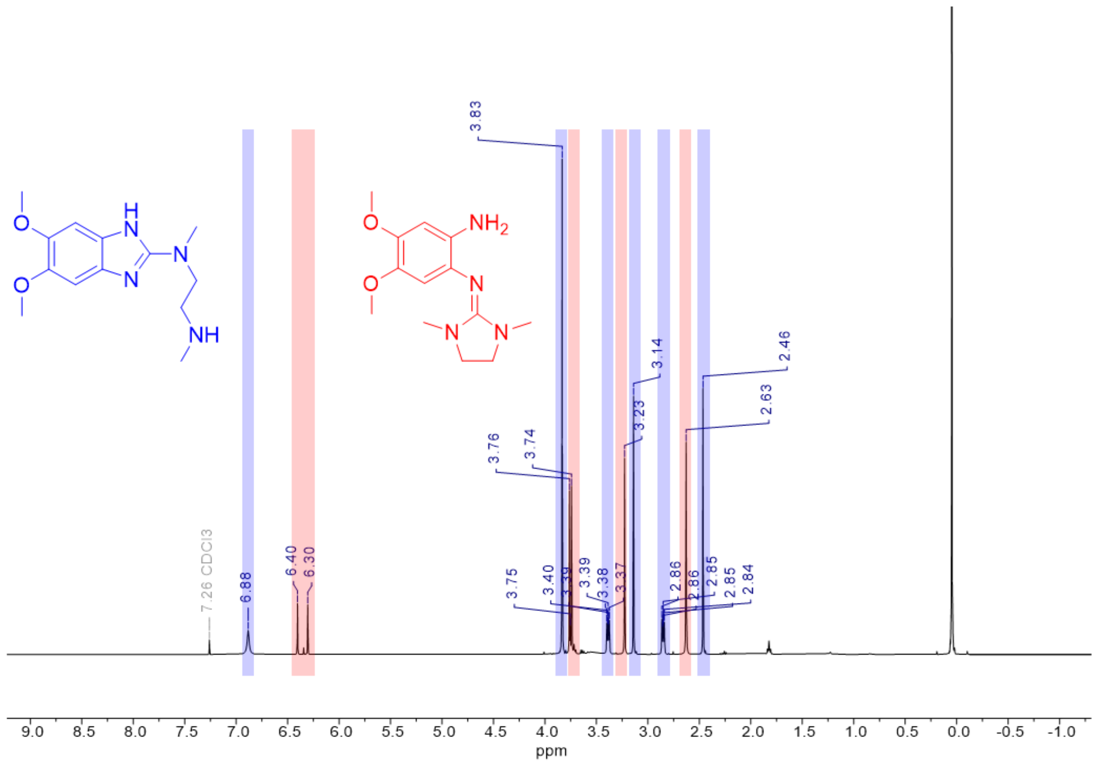

Figure S37: ^{1}H NMR spectrum (400 MHz, $CDCl_3$) of the two species 2-((1,3-dimethylimidazolidin-2-ylidene)amino)-4,5-dimethoxyaniline (red) and N^1-(5,6-dimethoxy-1*H*-benzo[*d*]imidazol-2-yl)-N^1,N^2-dimethylethane-1,2-diamine (blue). The impurity at 0.06 ppm is due to grease, while the impurities at 3.73 ppm and 1.85 ppm are due to tetrahydrofuran.

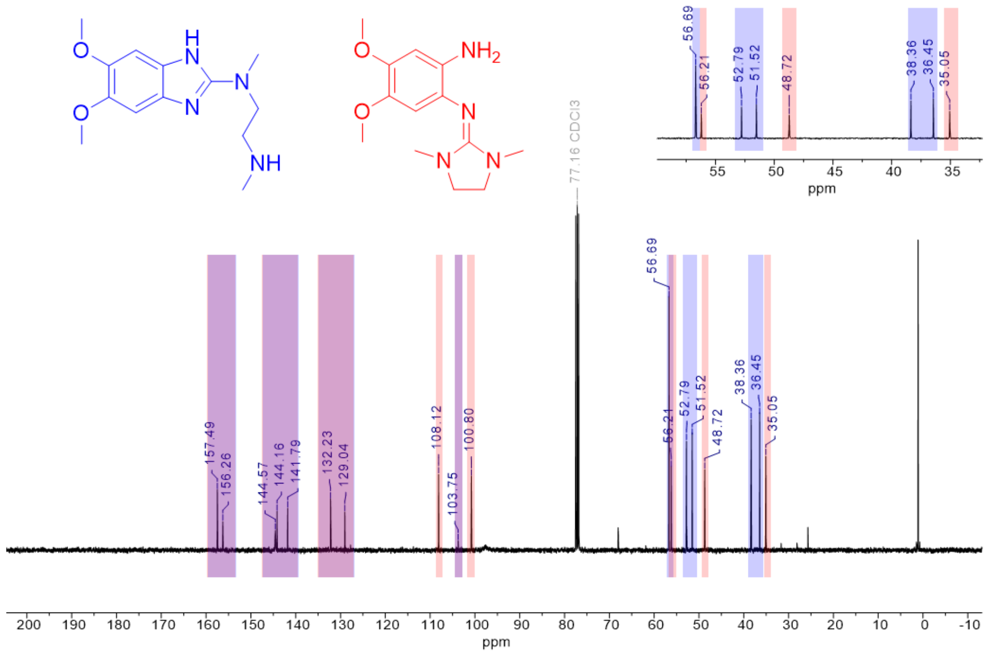

Figure S38: ^{13}C NMR spectrum (101 MHz, $CDCl_3$) of of the two species 2-((1,3-dimethylimidazolidin-2-ylidene)amino)-4,5-dimethoxyaniline (red) and N^1-(5,6-dimethoxy-1*H*-benzo[*d*]imidazol-2-yl)-N^1,N^2-dimethylethane-1,2-diamine (blue). Peaks without clear assignment are characterised by violet boxes. The impurity at 1.15 ppm is due to grease, while the impurities at 68.05 ppm and 25.68 ppm are due to tetrahydrofuran.

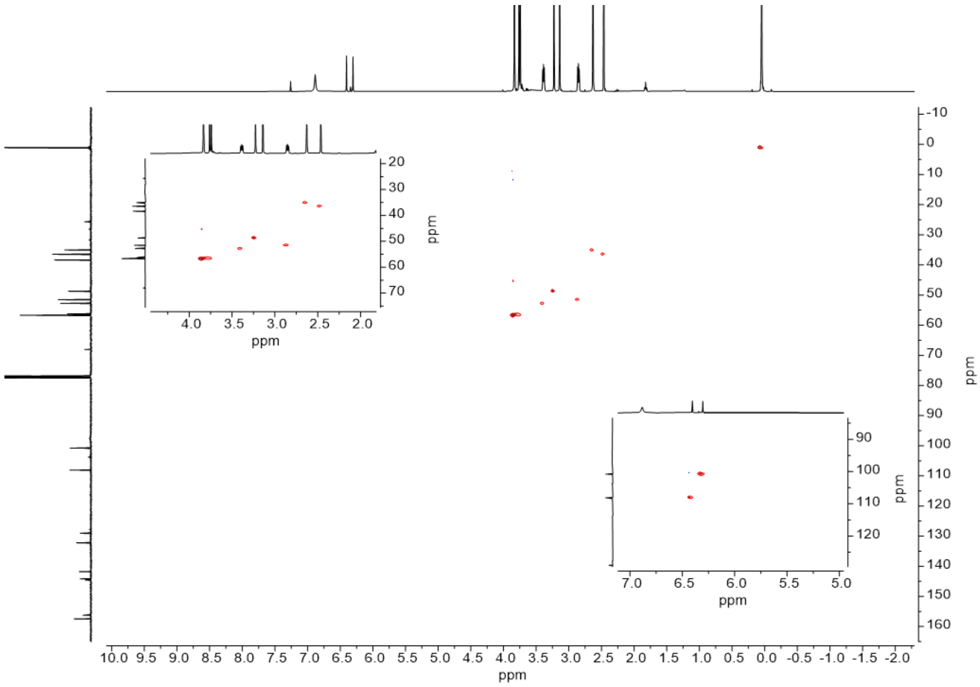

Figure S39: HSQC (^1H-^{13}C) spectrum of the two species 2-((1,3-dimethylimidazolidin-2-ylidene)amino)-4,5-dimethoxyaniline and *N*[1]-(5,6-dimethoxy-1*H*-benzo[*d*]imidazol-2-yl)-*N*[1],*N*[2]-dimethylethane-1,2-diamine.

5 Diguanidines

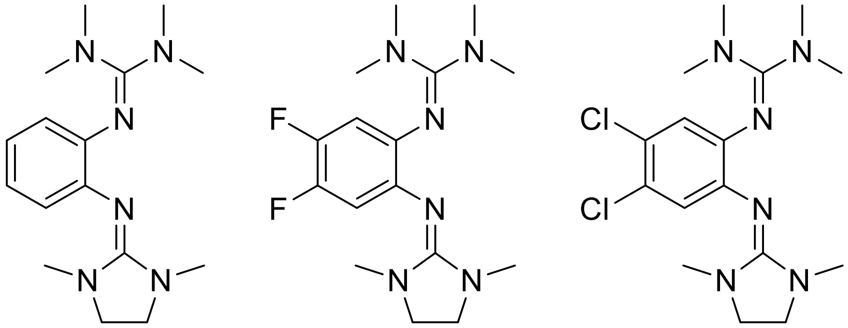

Scheme S3: Overview over the unsymmetric diguanidines 2-(2-((1,3-dimethylimidazolidin-2-ylidene)amino)phenyl)-1,1,3,3-tetramethylguanidine, 2-(2-((1,3-dimethylimidazolidin-2-ylidene)amino)-4,5-difluorophenyl)-1,1,3,3-tetramethylguanidine and 2-(4,5-dichloro-2-((1,3-dimethylimidazolidin-2-ylidene)amino)phenyl)-1,1,3,3-tetramethylguanidine.

5.1 2-(2-((1,3-Dimethylimidazolidin-2-ylidene)amino)phenyl)-1,1,3,3-tetramethylguanidine

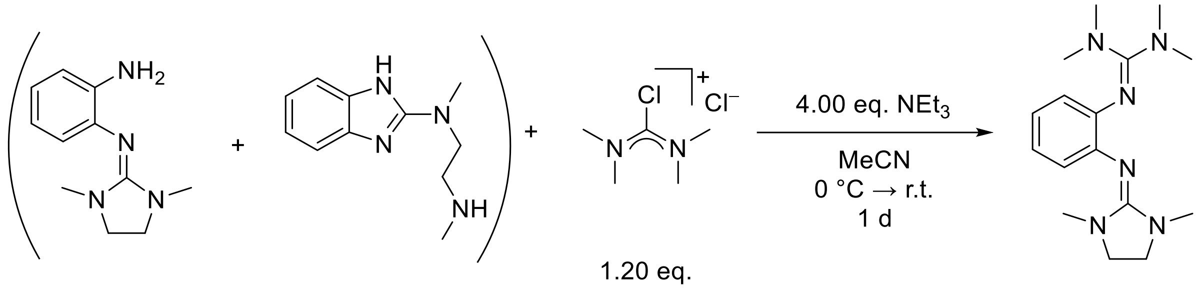

403 mg mixture of 2-((1,3-dimethylimidazolidin-2-ylidene)amino)aniline and *N*[1]-(1*H*-benzo[*d*]imidazol-2-yl)-*N*[1],*N*[2]-dimethylethane-1,2-diamine (1.97 mmol, 1.00 eq.) and 1.20 eq. activated urea (438 mg, 2.56 mmol) were dissolved in 8.00 mL acetonitrile under ice-cooling and 1.10 mL triethylamine (798 mg, 4.00 eq., 7.89 mmol) was added. The reaction mixture was stirred overnight. The solution was filtered and removed *in vacuo*. The residue was mixed with 10.00 mL of 15% degassed sodium hydroxide solution and extracted with 4 × 4.00 mL diethyl ether. The combined organic phases were dried over magnesium sulfate. The product is obtained as a viscous colourless liquid in 55% yield (330 mg, 1.09 mmol).

^{1}H NMR (600 MHz, $CDCl_3$): δ = 6.80-6.70 (m, 4 H, CH_{arom}), 3.17-3.16 (s, 4 H, 2×CH_2), 2.70 (s, 12 H, 4×CH_3), 2.59 (s, 6 H, 2×CH_3) ppm.

^{13}C NMR (151 MHz, $CDCl_3$): δ = 159.52 ($C_{q,Guan}$), 153.26 ($C_{q,arom}$), 141.97 ($C_{q,arom}$), 123.20 (CH$_{arom}$), 122.94 (CH$_{arom}$), 121.42 (CH$_{arom}$), 120.37 (CH$_{arom}$), 48.78 (NCH$_2$), 39.61 (NCH$_3$), 35.17 (NCH$_3$) ppm.

MS ESI(+): calculated: M+H$^+$ = 303.2292, found: M+H$^+$ = 303.2288.

CV:

Irreversibel oxidation at: E_{Ox1}: 0.06 V (Schulter), E_{Ox2}: 0.15 V, E_{Ox3}: 0.60-0.78 V (broad)

Irreversibel reduction at: E_{Red1}: -0.26 V, E_{Red2}: -0.46 V

UV-Vis (DCM, c =7.19801·10^{-5} mol·l^{-1}, d = 1 cm): λ_{max} (ε [l·mol^{-1}·cm^{-1}]) = 247 (4086), 284 (2871) nm.

Elemental analysis (%):

calculated:	C: 63.54	H: 8.67	N: 27.79
found:	C: 63.56	H: 9.21	N: 27.06

Analytical data:

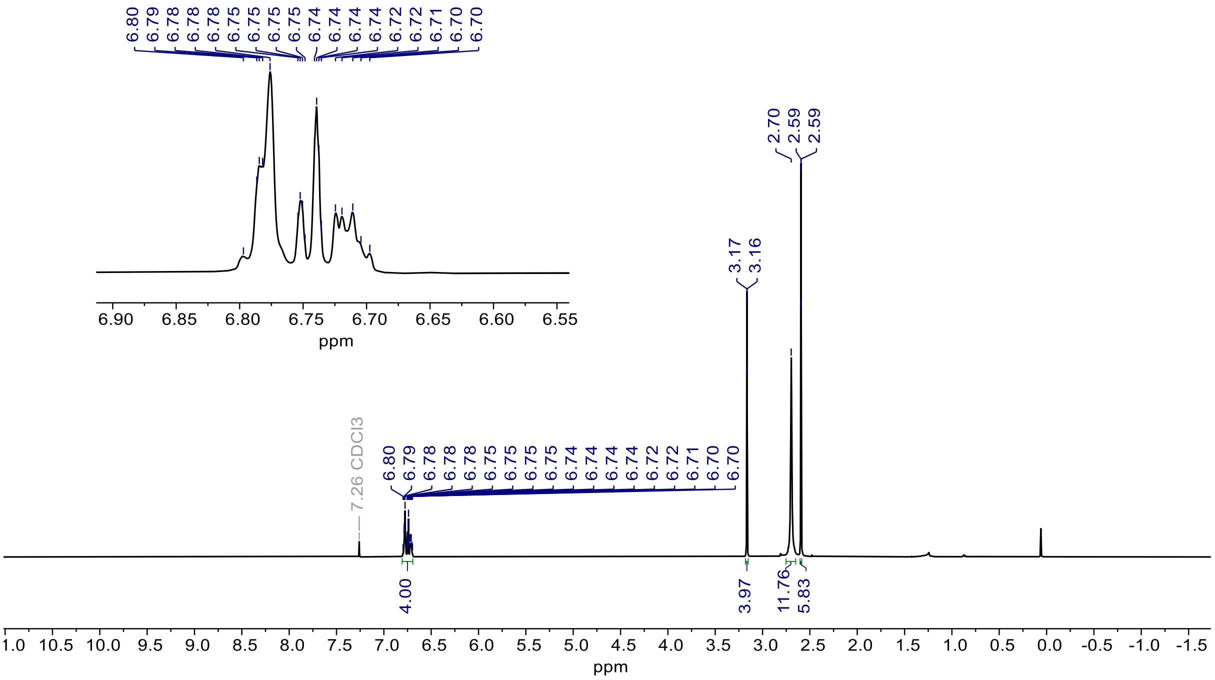

Figure S40: ^{1}H NMR spectrum (600 MHz, $CDCl_3$) of 2-(2-((1,3-dimethylimidazolidin-2-ylidene)amino)phenyl)-1,1,3,3-tetramethylguanidine. Impurity at 0.07 ppm is attributable to grease.

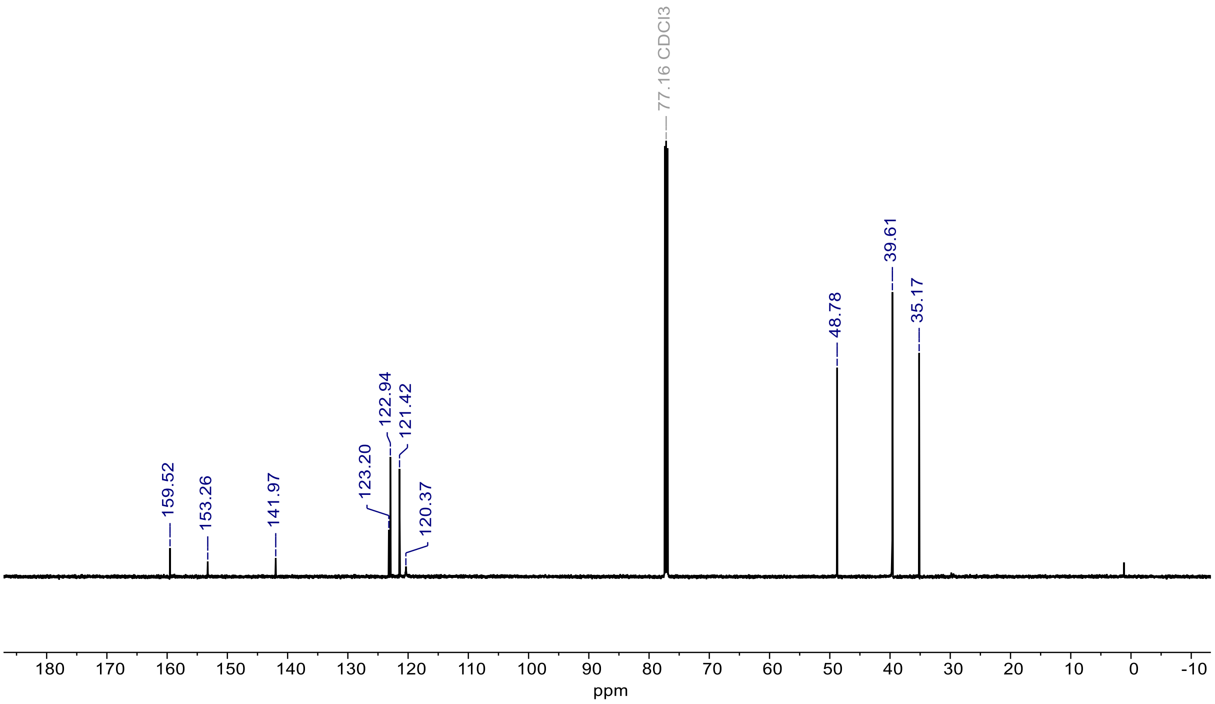

Figure S41: ^{13}C NMR spectrum (151 MHz, $CDCl_3$) of 2-(2-((1,3-dimethylimidazolidin-2-ylidene)amino)phenyl)-1,1,3,3-tetramethylguanidine.

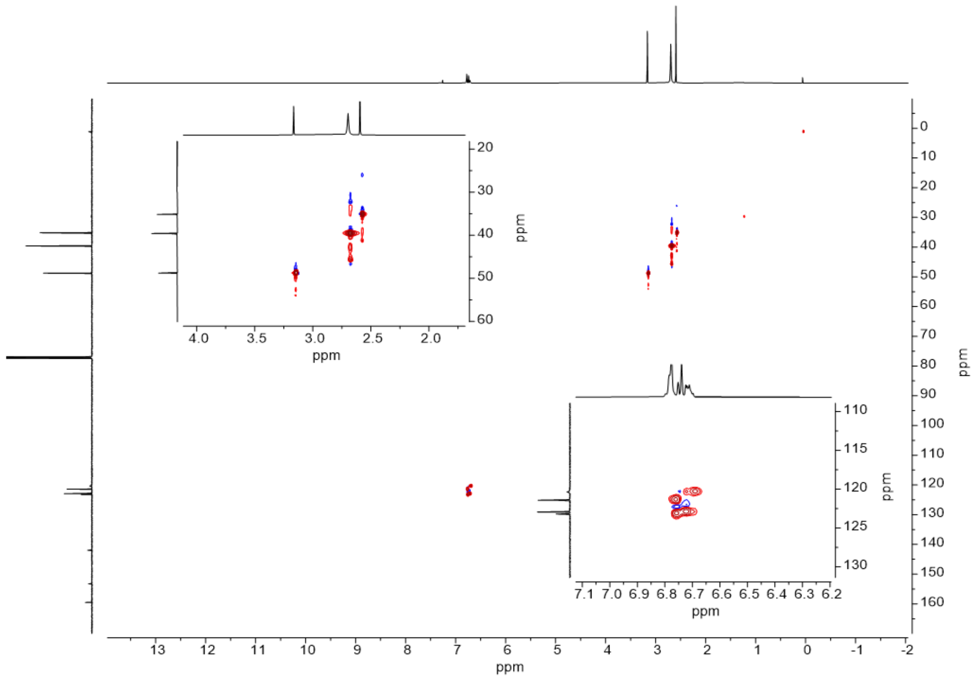

Figure S42: HSQC (^1H-^{13}C) spectrum of 2-(2-((1,3-dimethylimidazolidin-2-ylidene)amino)phenyl)-1,1,3,3-tetramethylguanidine.

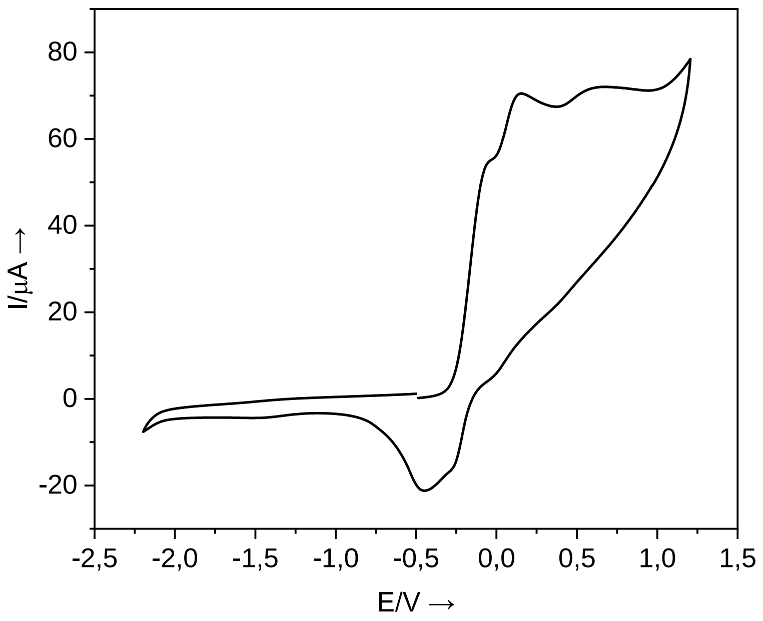

Figure S43: Cyclovoltammogram of 2-(2-((1,3-dimethylimidazolidin-2-ylidene)amino)phenyl)-1,1,3,3-tetramethylguanidine in dichloromethane ([*n*-Bu_4N][PF_6], 100 mV/s internally referenced to Fc^+/Fc).

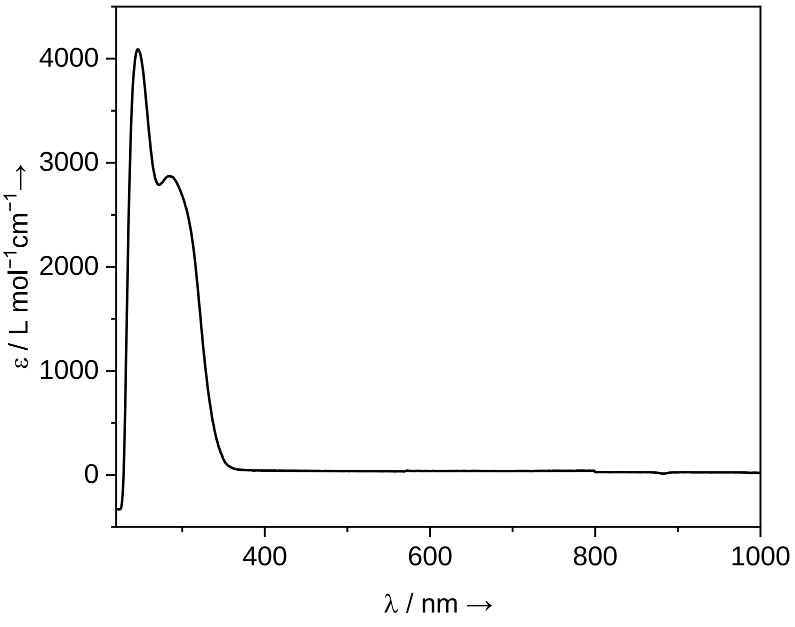

Figure S44: UV-Vis spectrum of 2-(2-((1,3-dimethylimidazolidin-2-ylidene)amino)phenyl)-1,1,3,3-tetramethylguanidine in DCM.

5.2 2-(2-((1,3-Dimethylimidazolidin-2-ylidene)amino)-4,5-difluorophenyl)-1,1,3,3-tetramethylguanidine

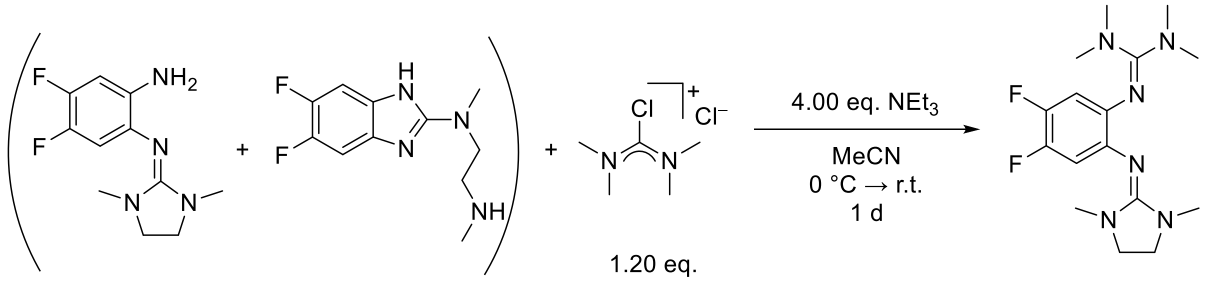

226 mg of a mixture of 2-((1,3-dimethylimidazolidin-2-ylidene)amino)-4,5-difluoroaniline and *N*[1]-(5,6-difluoro-1*H*-benzo[d]imidazol-2-yl)-*N*[1],*N*[2]-dimethylethane-1,2-diamine (925 µmol, 1.00 eq) and 1.20 eq. activated urea (189 mg, 1.11 mmol) were dissolved in 2.00 mL acetonitrile under ice-cooling and 516 µL triethylamine (374 mg, 4.00 eq., 3.70 mmol) was added. The reaction mixture was stirred overnight. The solution was filtered and removed *in vacuo*. The residue was mixed with 8.00 mL of 15% degassed sodium hydroxide solution and extracted with 4 × 2.00 mL diethyl ether. The combined organic phases were dried over magnesium sulfate. The product is obtained as a viscous orange liquid in 39% yield (123 mg, 363 µmol).

^{1}H NMR (400 MHz, $CDCl_3$): δ = 6.65 – 6.49 (m, 2 H, CH_{arom}), 3.19 (s, 4 H, 2×CH_2), 2.69 (s, 12 H, 4×CH_3), 2.61 (s, 6 H, 2×CH_3) ppm.

^{19}F NMR (376 MHz, $CDCl_3$): δ = -59.90 ppm.

MS: ESI(+): calculated: M+H$^+$ = 339.2103, found: M+H$^+$ = 339.2098.

CV:

1. Reversible step at: $E_{1/2}$: -0.145 V (E_{Ox1}: -0.07 V, E_{Red1}: -0.22 V)

2. Reversible step at: $E_{1/2}$: 0.025 V (E_{Ox2}: 0.10 V, E_{Red2}: -0.05 V)

UV-Vis (DCM, c = 8.4428095·10^{-5} mol·l^{-1}, d = 1 cm): λ_{max} (ε [l·mol^{-1}·cm^{-1}]) = 251 (6400), 280 (5870), 314 (5360) nm.

Elemental analysis (%):

calculated:	C: 56.79	H: 7.15	N: 24.83
found:	C: 56.59	H: 7.30	N: 24.29

Analytical data:

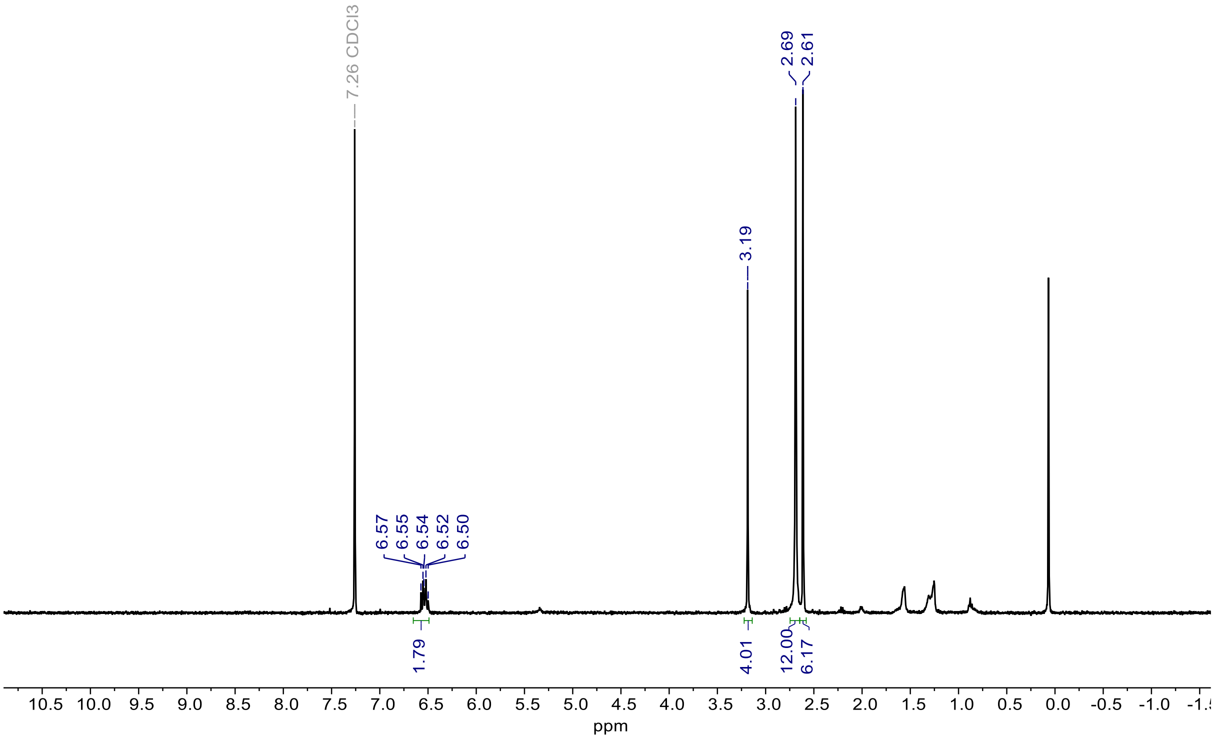

Figure S45: 1H NMR spectrum (400 MHz, $CDCl_3$) of 2-(2-((1,3-dimethylimidazolidin-2-ylidene)amino)-4,5-difluorophenyl)-1,1,3,3-tetramethylguanidine. The impurities at 0.07 ppm, 0.88 ppm and 1.21 ppm are due to grease, while the impurity at 1.57 ppm is due to water.

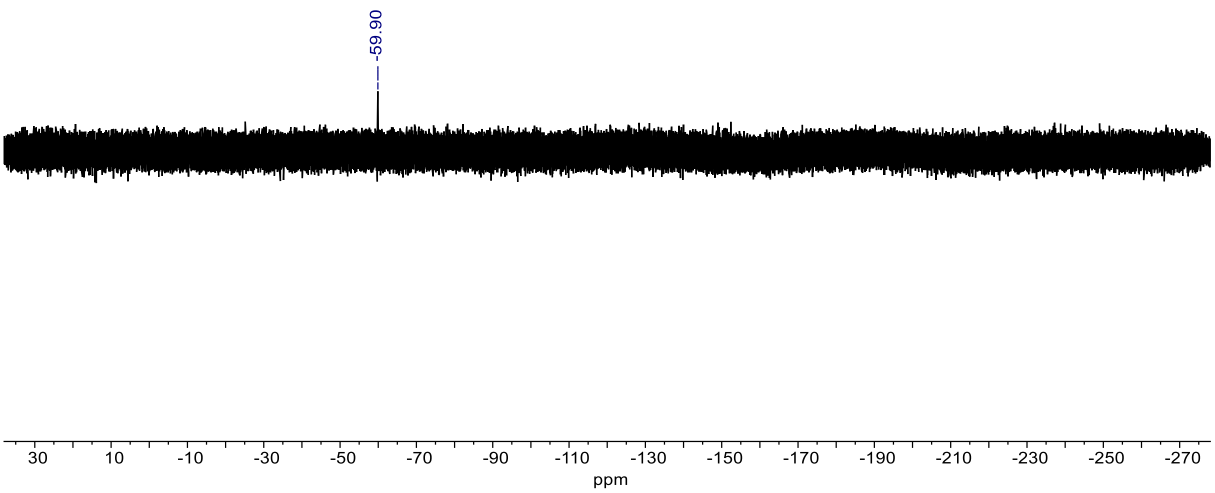

Figure S46: ^{19}F NMR spectrum (376 MHz, $CDCl_3$) of 2-(2-((1,3-dimethylimidazolidin-2-ylidene)amino)-4,5-difluorophenyl)-1,1,3,3-tetramethylguanidine.

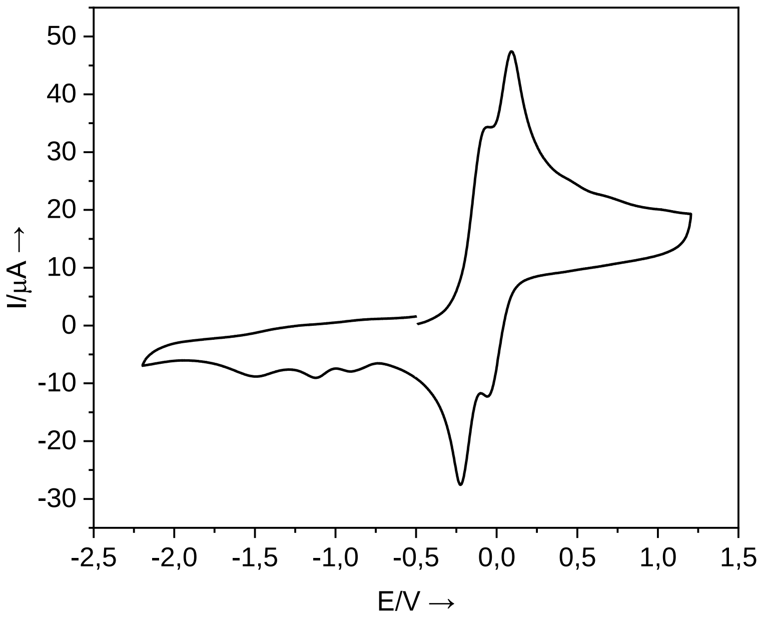

Figure S47: Cyclovoltammogram of 2-(2-((1,3-dimethylimidazolidin-2-ylidene)amino)-4,5-difluorophenyl)-1,1,3,3-tetramethylguanidine in dichloromethane ([*n*-Bu_4N][PF_6], 100 mV/s internally referenced to Fc^+/Fc).

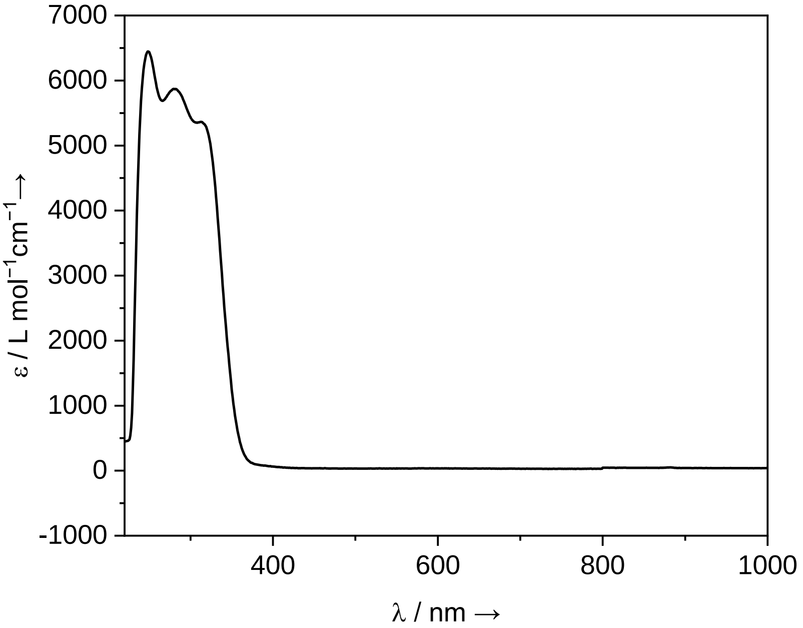

Figure S48: UV-Vis spectrum of 2-(2-((1,3-dimethylimidazolidin-2-ylidene)amino)-4,5-difluorophenyl)-1,1,3,3-tetramethylguanidine in DCM.

5.3 2-(4,5-dichloro-2-((1,3-dimethylimidazolidin-2-ylidene)amino)phenyl)-1,1,3,3-tetramethylguanidine

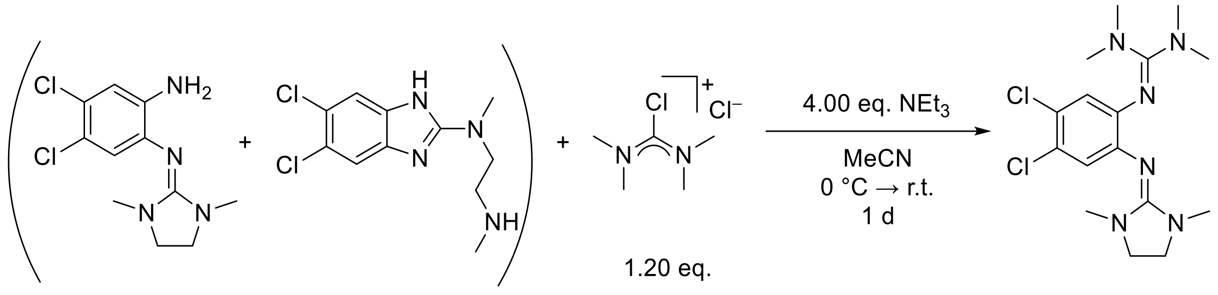

209 mg of 2-((1,3-dimethylimidazolidin-2-ylidene)amino)-4,5-dichloroaniline (765 µmol, 1.00 eq) and 1.30 eq of activated urea (170 mg, 995 µmol) were dissolved in 2.00 mL of acetonitrile under ice cooling and 426 µL of triethylamine (310 mg, 4.00 eq., 3.06 mmol) were added. The reaction mixture was stirred overnight. The solution was filtered and removed under vacuum. The residue was mixed with 4.00 mL of 15% degassed sodium hydroxide solution and extracted with 2 × 2.00 mL of diethyl ether. The combined organic phases were dried over magnesium sulfate. The product is obtained as a viscous yellowish liquid in a 35% yield (100 mg, 269 µmol).

^{1}H NMR (400 MHz, $CDCl_3$): δ = δ 6.78 (d, J = 8.7 Hz, 2 H, CH_{arom}), 3.20 (s, 4 H, 2×CH_2), 2.69 (s, 12 H, 4×CH_3), 2.63 (s, 6 H, 2×CH_3) ppm.

^{13}C NMR (101 MHz, $CDCl_3$): δ = 160.28 ($C_{q,Guan}$), 154.32 ($C_{q,arom}$), 142.14 ($C_{q,arom}$), 123.40 ($CCl_{q,arom}$), 123.25 (CH_{arom}, 2C), 123.11($CCl_{q,arom}$), 48.67 (NCH_2), 39.61 (NCH_3), 35.08 (NCH_3) ppm.

MS: ESI(+): calculated: M+H$^+$ = 371.1512, found: M+H$^+$ = 371.1506.

CV:

1. Reversible step at: $E_{1/2}$:-0.045 V (E_{Ox1}: 0.03 V, E_{Red1}: -0.12 V)

2. Reversible step at: $E_{1/2}$:0.17 V (E_{Ox2}: 0.24 V, E_{Red2}: 0.10 V)

UV-Vis (DCM, c = 5.129·10^{-5} mol·l^{-1}, d = 1 cm): λ_{max} (ε [l·mol^{-1}·cm^{-1}]) = 254 (2247), 294 (2441), 326 (1912, shoulder) nm.

Elemental analysis (%):

calculated: C: 51.76% H: 6.51 % N: 22.63 %

found: C: 51.68 % H: 6.74 % N: 21.84%

Analytical data:

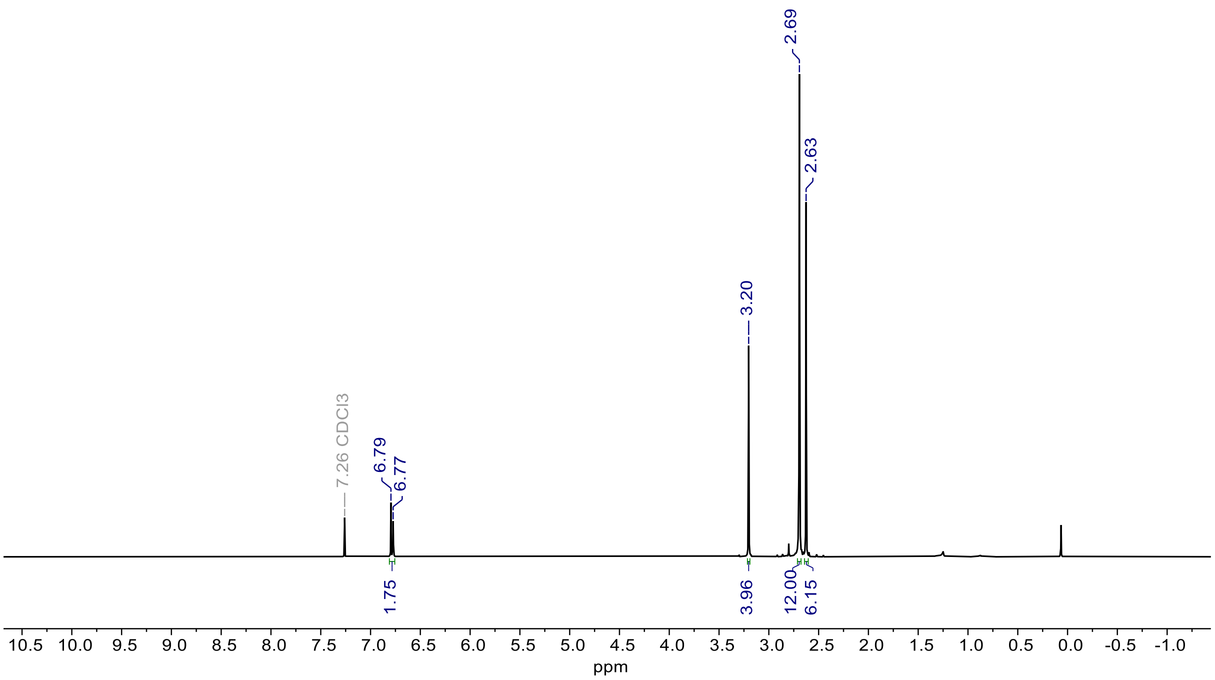

Figure S49: ^{1}H NMR spectrum (400 MHz, $CDCl_3$) of 2-(4,5-dichloro-2-((1,3-dimethylimidazolidin-2-ylidene)amino)phenyl)-1,1,3,3-tetramethylguanidine. Impurity at 0.07 ppm is attributable to grease.

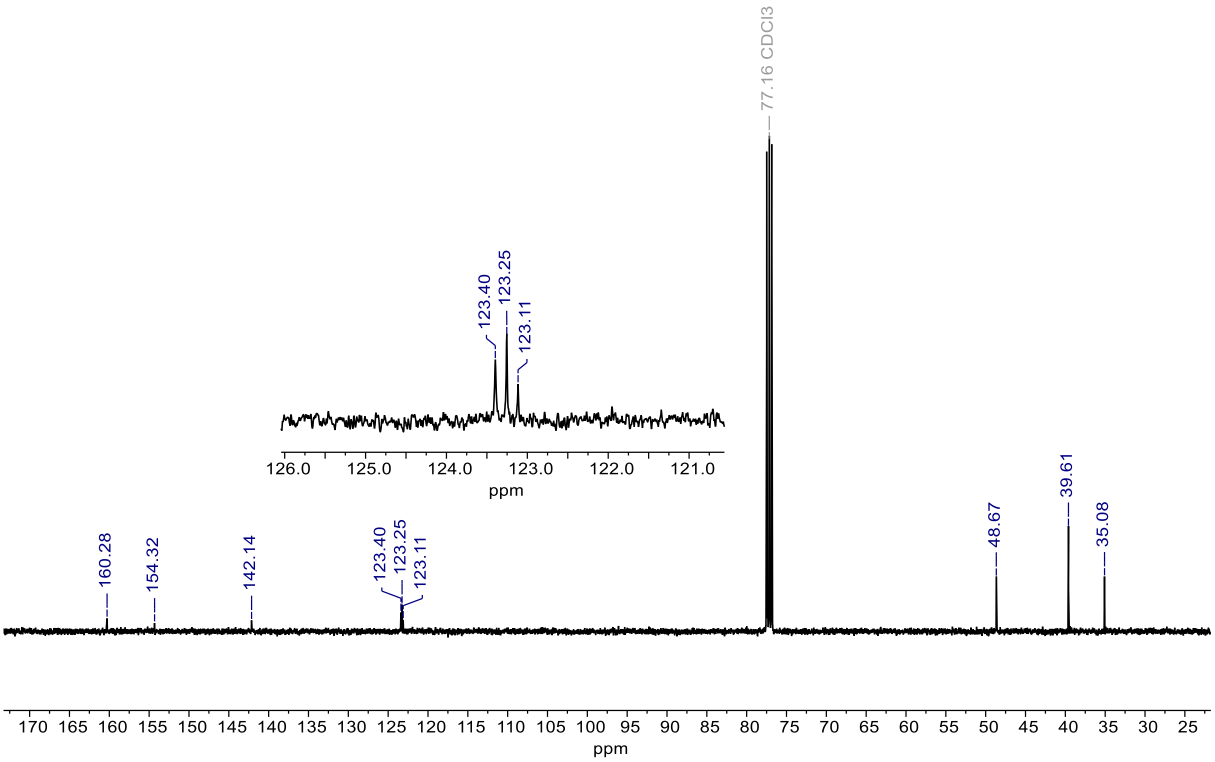

Figure S50: ^{13}C NMR spectrum (101 MHz, $CDCl_3$) of 2-(4,5-dichloro-2-((1,3-dimethylimidazolidin-2-ylidene)amino)phenyl)-1,1,3,3-tetramethylguanidine.

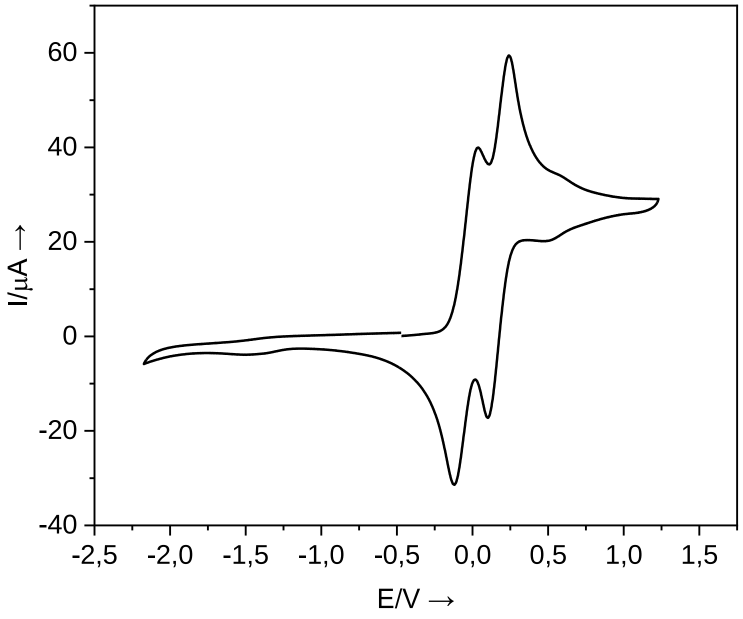

Figure S51: Cyclovoltammogram of 2-(4,5-dichloro-2-((1,3-dimethylimidazolidin-2-ylidene)amino)phenyl)-1,1,3,3-tetramethylguanidine in dichloromethane ([*n*-Bu_4N][PF_6], 100 mV/s internally referenced to Fc^+/Fc).

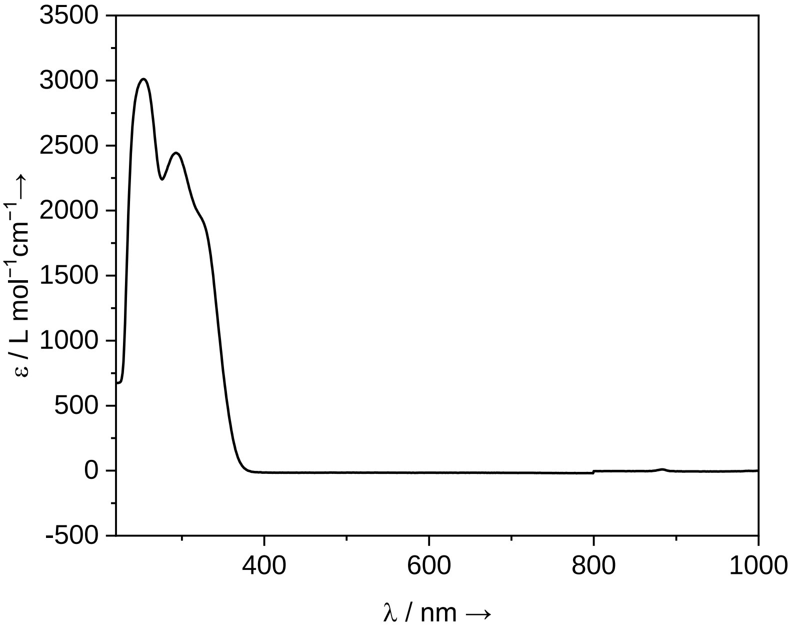

Figure S52: UV-Vis spectrum of 2-(4,5-dichloro-2-((1,3-dimethylimidazolidin-2-ylidene)amino)phenyl)-1,1,3,3-tetramethylguanidine in DCM.

6 Diguanidine complex

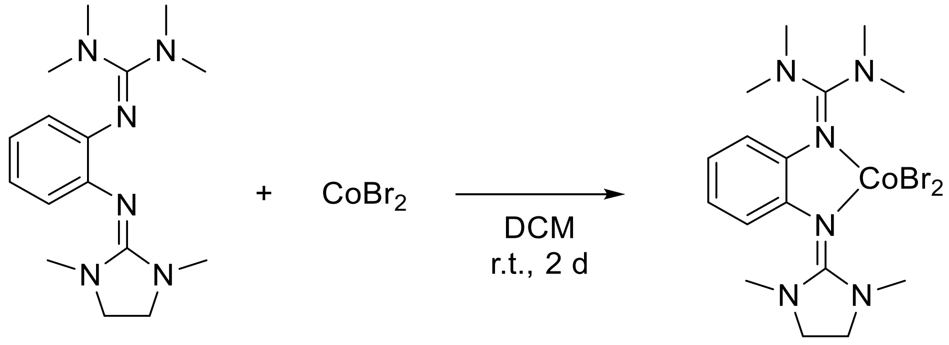

2-(2-((1,3-dimethylimidazolidin-2-ylidene)amino)phenyl)-1,1,3,3-tetramethylguanidine (13.0 mg, 1.00 eq., 42.9 µmol) and cobalt bromide (9.40 mg, 1.00 eq., 42.9 µmol) were dissolved in 2.00 mL dichloromethane and stirred for 2 days at room temperature. The solvent was removed in a fine vacuum and the light blue solid was washed with 2 × 2.00 mL diethyl ether. The product was obtained as blue powder in quantitative yield (22.4 mg, 42.9 µmol). The solid was dissolved in 2.00 mL dichloromethane and layered with 4.00 mL *n*-hexane, whereupon blue crystals were obtained.

CV:

1. Reversible step at: $E_{1/2}$:0.21 V (E_{Ox2}: 0.26 V, E_{Red2}: 0.16 V)

2. Reversible step at: $E_{1/2}$: 0.66 V (E_{Ox1}: 0.74 V, E_{Red1}: 0.58 V)

UV-Vis (DCM, c =1.1031·10^{-4} mol·l^{-1}, d = 1 cm): λ_{max} (ε [l·mol^{-1}·cm^{-1}]) = 244 (5328), 286 (4296), 571 (45, broad), 632 (78, broad), 675 (100, broad) nm.

IR (ATR): $\tilde{v}$ = 3167.08 , 2928.35, 2871.23**,** 1714.03, 1610.17, 1580.57, 1558.99, 1517.29, 1481.33, 1459.22, 1445.05, 1401.98, 1392.45, 1330.30, 1291.55, 1229.89, 1204.49, 1151.19, 1088.41, 1055.35, 1029.01, 1003.06, 979.31, 925.79, 878.73, 839.70, 815.75, 767.91, 741.56, 697.81, 660.09, 628.54, 571.08, 538.52, 513.86, 502.96, 470.75, 451.99, 433.23 cm^{-1}.

Elemental analysis (%): • 0.25 Et_2O

calculated:	C: 37.83	H: 5.32	N: 15.57
found:	C: 37.64	H: 5.79	N: 15.36

Analytical data:

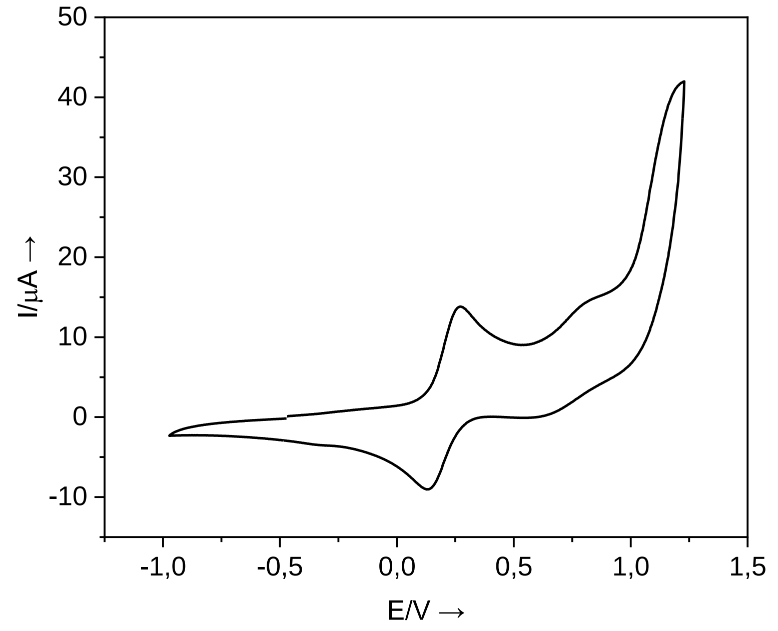

Figure S53: Cyclic voltammogram of the complex from 2-(2-((1,3-dimethylimidazolidin-2-ylidene)amino)phenyl)-1,1,3,3-tetramethylguanidine with $CoBr_2$ in dichloromethane ([*n*-Bu_4N][PF_6], 100 mV/s internally referenced to Fc^+/Fc).

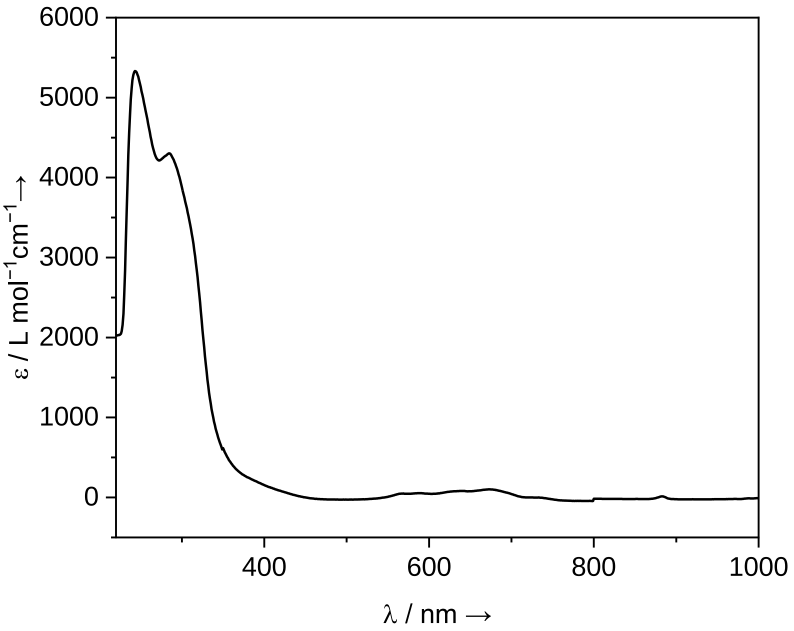

Figure S54: UV-Vis of the complex from 2-(2-((1,3-dimethylimidazolidin-2-ylidene)amino)phenyl)-1,1,3,3-tetramethylguanidine with $CoBr_2$ in DCM under Argon.

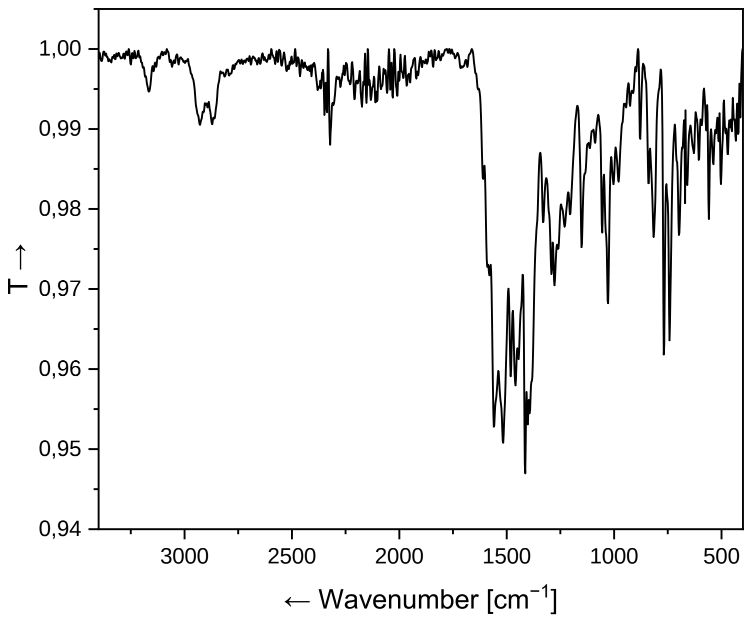

Figure S55: IR spectrum of the complex from 2-(2-((1,3-dimethylimidazolidin-2-ylidene)amino)phenyl)-1,1,3,3-tetramethylguanidine with $CoBr_2$.

Table S6: Molecular view of the complex from 2-(2-((1,3-dimethylimidazolidin-2-ylidene)amino)phenyl)-1,1,3,3-tetramethylguanidine with $CoBr_2$, as well as crystallographic data. H atoms have been omitted for clarity. Displacement ellipsoids correspond to 50% probability of residence. Colour coding: C dark-grey, N blue, Co violet, Br brown.

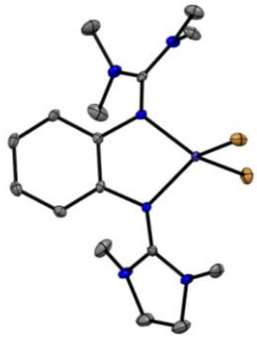

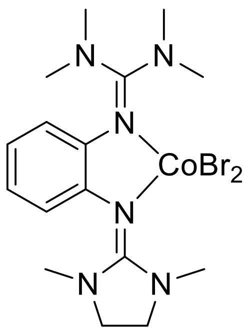

Empirical formula	$C_{16}H_{26}N_6CoBr_2$
Formula weight	521.18
Temperature/K	100.00
Crystal system	monoclinic
Space group	$P2_1/c$
a/Å	11.9513(7)
b/Å	10.6770(6)
c/Å	16.3518(9)

α/°	90
β/°	103.587(2)
γ/°	90
Volume/Å^3	2028.2(2)
Z	4
ρ_{calc}g/cm^3	1.707
μ/mm^{-1}	4.798
F(000)	1044.0
Crystal size/mm^3	0.38 × 0.27 × 0.22
Radiation	MoKα (λ = 0.71073)
2Θ range for data collection/°	4.596 to 56.712
Index ranges	-15 ≤ h ≤ 15, -14 ≤ k ≤ 14, -21 ≤ l ≤ 21
Reflections collected	65456
Independent reflections	5050 [R_{int} = 0.0821, R_{sigma} = 0.0407]
Data/restraints/parameters	5050/7/241
Goodness-of-fit on F^2	1.041
Final R indexes [I>=2σ (I)]	R_1 = 0.0324, wR_2 = 0.0824
Final R indexes [all data]	R_1 = 0.0360, wR_2 = 0.0844
Largest diff. peak/hole / e Å^{-3}	1.11/-0.52

7 Secondary amines (Phenyl residue)

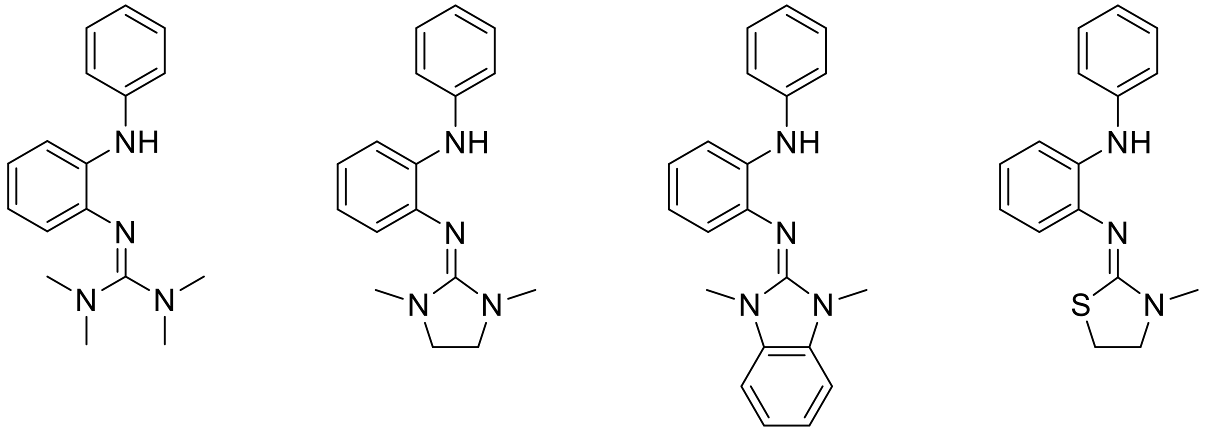

L3 L4 L5 L6

Scheme S4: Overview over the ligands L3, L4, L5 and L6 with phenyl group.

7.1 1,1,3,3-Tetramethyl-2-(2-(phenylamino)phenyl)guanidine (L3)

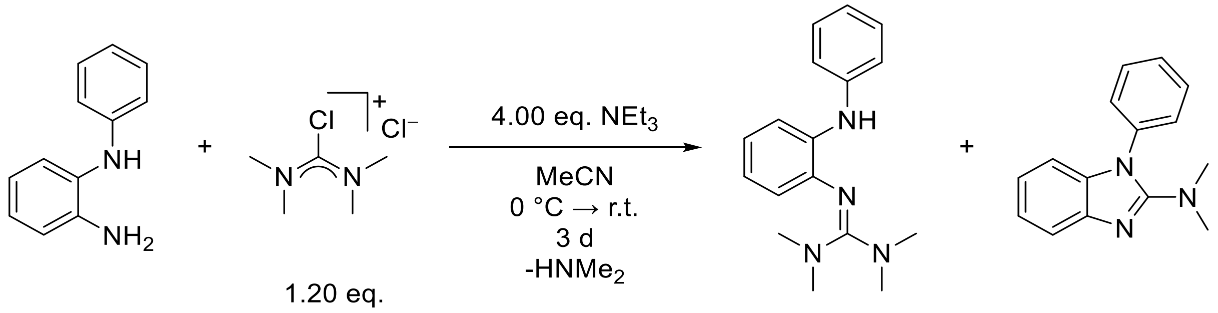

300 mg of N^1-phenylbenzene-1,2-diamine (1.00 eq., 1.63 mmol) and 1.20 eq. of activated urea (334 mg, 1.95 mmol) were dissolved in 2.00 mL of acetonitrile under ice cooling, and 907 µL of triethylamine (659 mg, 4.00 eq., 6.51 mmol) were added. The reaction mixture was stirred over three days. The solvent was filtered off and removed under vacuum. The residue was mixed with 5.00 mL of 15% degassed sodium hydroxide solution and extracted with 3 × 2.00 mL of diethyl ether. The combined organic phases were dried over magnesium sulfate. The product is obtained as a viscous liquid as a product mixture of 1,1,3,3-tetramethyl-2-(2-(phenylamino)phenyl)guanidine (L3), *N*,*N*-dimethyl-1-phenyl-1*H*-benzo[*d*]imidazol-2-amine and dimethylamine in 72% yield (333 mg, 1.18 mmol). Due to rapid rearrangement in solution, the guanidine ligand was not isolated.

1,1,3,3-tetramethyl-2-(2-(phenylamino)phenyl)guanidine (L3):

^{1}H NMR (400 MHz, $CDCl_3$): δ = 7.31 – 7.27 (m, 1 H, CH_{arom}), 7.24 (m, 2 H, CH_{arom}), 7.20 – 7.16 (m, 2 H, CH_{arom}), 6.90 – 6.71 (m, 3 H, CH_{arom}), 6.46 (dd, *J* = 7.6, 1.6 Hz, 1 H, CH_{arom}), 2.71 (s, 12 H, 4×CH_3) ppm.

^{13}C NMR (101 MHz, $CDCl_3$): δ = 160.28 ($C_{q,Guan}$), 143.70 ($C_{q,arom}$), 130.03 ($C_{q,arom}$), 129.22 (CH_{arom}), 126.70 ($C_{q,arom}$), 120.63 (CH_{arom}), 120.31 (CH_{arom}), 119.67 (CH_{arom}), 118.31 (CH_{arom}), 108.71 (CH_{arom}), 39.79 (NCH_3) ppm.

N,*N*-dimethyl-1-phenyl-1*H*-benzo[*d*]imidazol-2-amine (rearranged species):

^{1}H NMR (400 MHz, $CDCl_3$): δ = 7.60 – 7.42 (m, 6 H, CH_{arom}), 7.04 – 7.01 (m, 2 H, CH_{arom}), 2.85 (s, 6 H, 4×CH_3) ppm. Some signals are missing due to overlapping.

^{13}C NMR (101 MHz, $CDCl_3$): δ = 41.22 (N*C*H_3) ppm. Due to low concentration only the *C*H_3 signals are visible.

Analytical data:

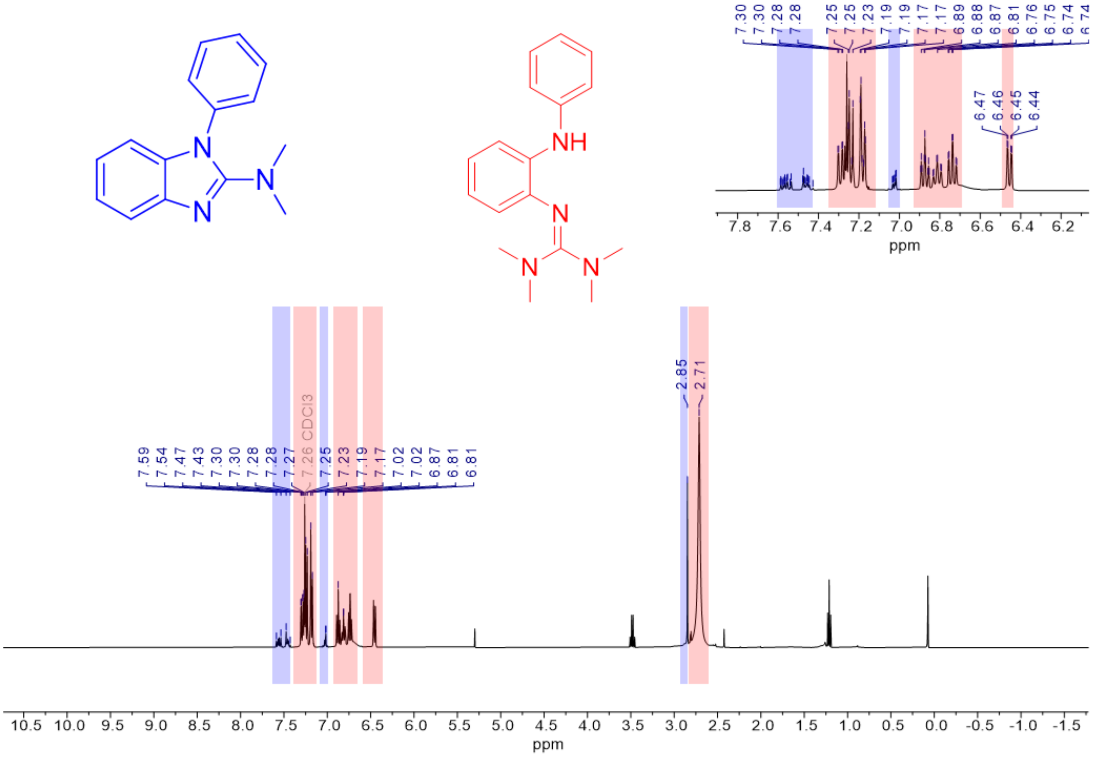

Figure S56: ^{1}H NMR spectrum (400 MHz, $CDCl_3$) of the two species 1,1,3,3-tetramethyl-2-(2-(phenylamino)phenyl)guanidine (red) and *N*,*N*-dimethyl-1-phenyl-1*H*-benzo[*d*]imidazol-2-amine (blue). The impurities at 0.07 ppm are due to grease, while the impurities at 1.21 ppm and 3.49 ppm are due to diethylether.

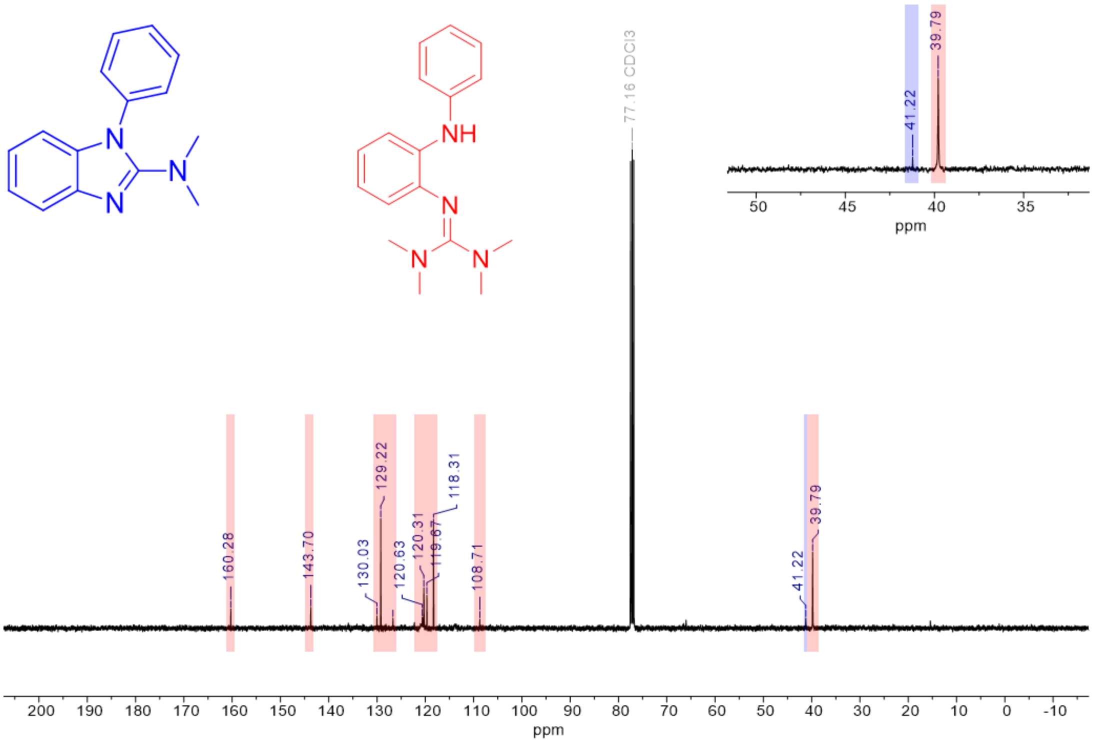

Figure S57: ^{13}C NMR spectrum (101 MHz, $CDCl_3$)) of the two species 1,1,3,3-tetramethyl-2-(2-(phenylamino)phenyl)guanidine (red) and *N*,*N*-dimethyl-1-phenyl-1*H*-benzo[*d*]imidazol-2-amine (blue).

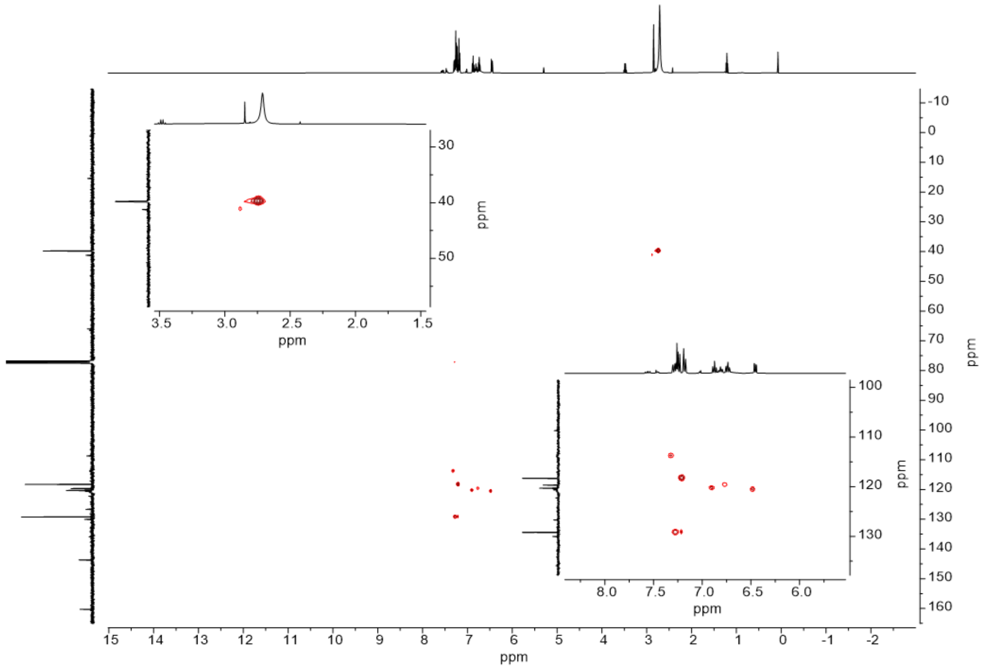

Figure S58: HSQC (^{1}H-^{13}C) spectrum of the two species 1,1,3,3-tetramethyl-2-(2-(phenylamino)phenyl)guanidine and *N*,*N*-dimethyl-1-phenyl-1*H*-benzo[*d*]imidazol-2-amine.

7.2 2-((1,3-Dimethylimidazolidin-2-ylidene)amino)-*N*-phenylaniline (L4)

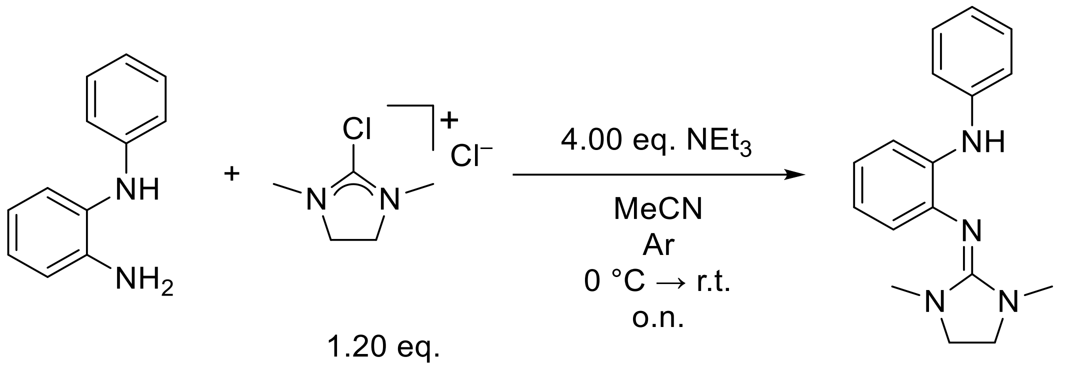

500 mg N^1-phenylbenzene-1,2-diamine (1.00 eq., 2.71 mmol) and 1.20 eq. activated urea (551 mg, 3.26 mmol) were dissolved in 10.00 ml of acetonitrile under ice cooling and 1.51 ml of triethylamine (1.10 g, 4.00 eq., 10.86 mmol) were added. The reaction mixture was stirred overnight. The solvent was filtered off and removed under vacuum. The residue was mixed with 10.00 ml of 15% degassed sodium hydroxide solution and extracted with 2 × 5.00 ml of diethyl ether. The combined organic phases were dried over magnesium sulfate. The product is obtained as a viscous liquid in 84% yield (645 mg, 2.29 mmol). Colourless crystals were obtained from dichloromethane at -31 °C.

^{1}H NMR (600 MHz, $CDCl_3$): δ = 7.28 – 7.26 (m, 1 H, CH_{arom}), 7.26 – 7.23 (m, 2 H, CH_{arom}), 7.18 – 7.14 (m, 2 H, CH_{arom}), 6.90 – 6.86 (m, 1 H, CH_{arom}), 6.84 – 6.79 (m, 2 H, CH_{arom}), 6.77 – 6.72 (m, 1 H, CH_{arom}), 6.47 (s, 1 H, N*H*), 3.29 (s, 4 H, 2×CH_2), 2.68 (d, *J* = 1.1 Hz, 6 H, 2×CH_3) ppm.

^{13}C NMR (151 MHz, $CDCl_3$): δ = 156.69 ($C_{q,Guan}$), 143.74 ($C_{q,arom}$), 138.02 ($C_{q,arom}$), 136.03 ($C_{q,arom}$), 130.13 (CH_{arom}), 129.23 (CH_{arom}), 126.93 (CH_{arom}), 122.15 (CH_{arom}), 121.00 (CH_{arom}), 120.33 (CH_{arom}), 119.69 (CH_{arom}), 118.25 (CH_{arom}), 114.11 (CH_{arom}), 48.72 (NCH_2), 35.25 (NCH_3) ppm.

CV:

Irreversibel oxidation at: E_{Ox1}: 0.03 V, E_{Ox2}: 0.77 V.

Irreversibel reduction at: E_{Red1}: 0.39 V, E_{Red2}: 0.34 V, E_{Red2}: -0.55 V.

UV-Vis (DCM, c =4.99·10^{-5} mol·l^{-1}, d = 1 cm): λ_{max} (ε [l·mol^{-1}·cm^{-1}]) = 252 (7383), 298 (8417) nm.

IR (ATR): $\tilde{\nu}$ = 3242.69, 3173.96, 3053.85, 3023.47, 2925.83, 2859.30, 2835.37, 1631.79, 1603.46, 1577.92, 1512.26, 1495.76, 1474.33, 1452.18, 1418.63, 1396.17, 1374.97, 1339.37, 1314.12, 1280.13, 1268.79, 1228.79, 1192.70, 1177.41, 1100.92, 1075.05, 1030.27,991.31, 969.05, 924.19, 891.16, 864.83, 843.77, 827.14, 776.90, 737.58, 690.55, 635.97, 599.50, 541.54, 496.56, 469.96, 450.37, 409.40 cm^{-1}.

Elemental analysis:

calculated:	C: 72.83	H: 7.19	N: 19.98
found:	C: 72.70	H: 7.10	N: 20.15

Analytical data:

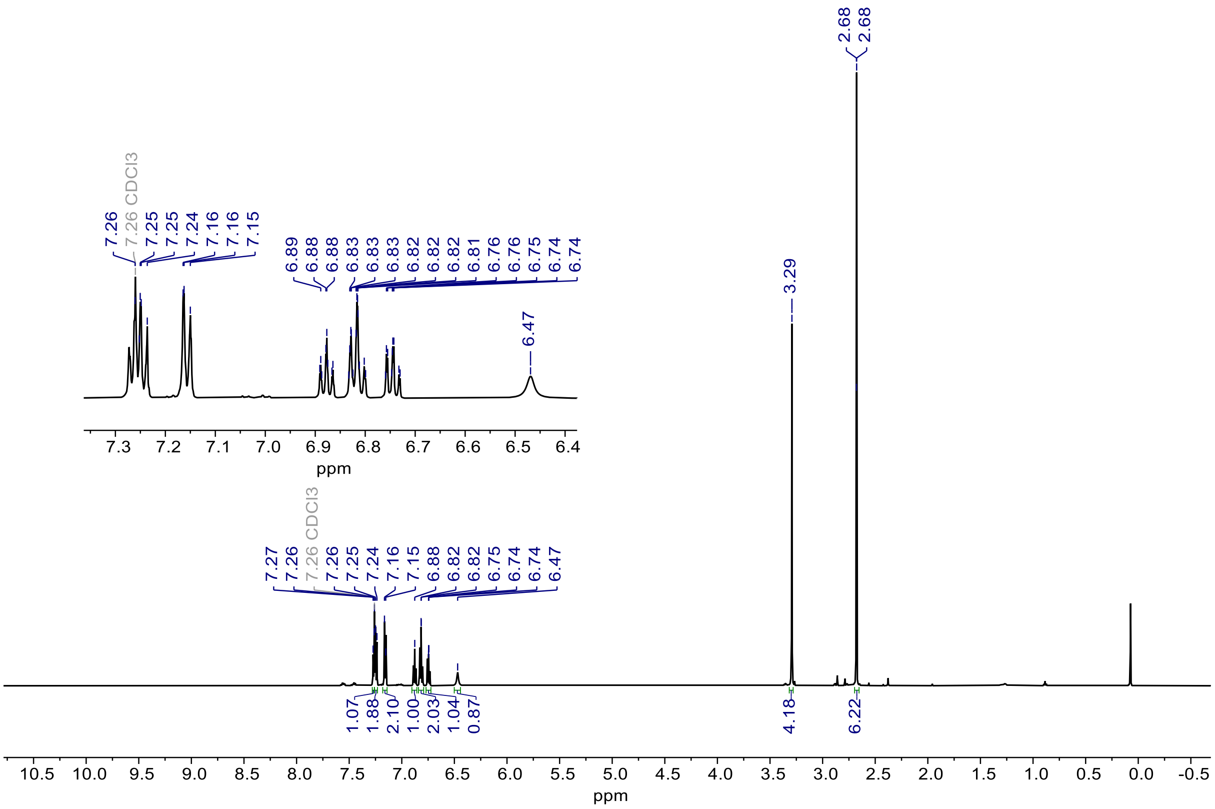

Figure S59: ^{1}H NMR spectrum (600 MHz, $CDCl_3$) of L4. The impuritie at 0.07 ppm is due to grease.

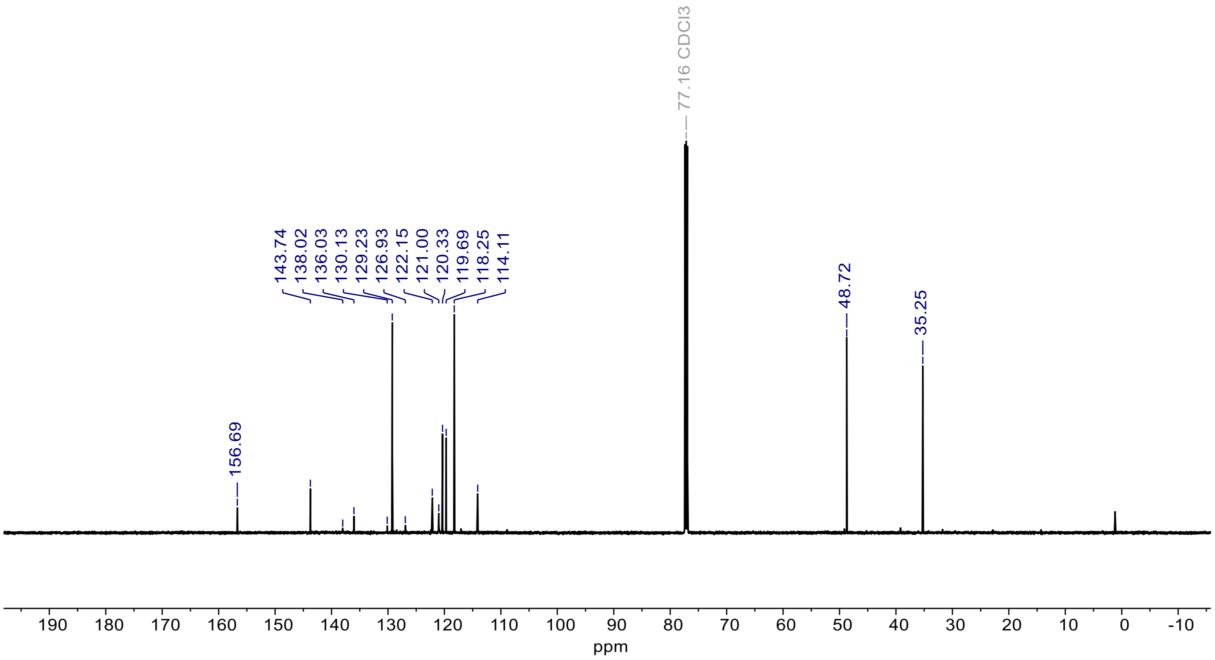

Figure S60: ^{13}C NMR spectrum (151 MHz, $CDCl_3$) of L4.

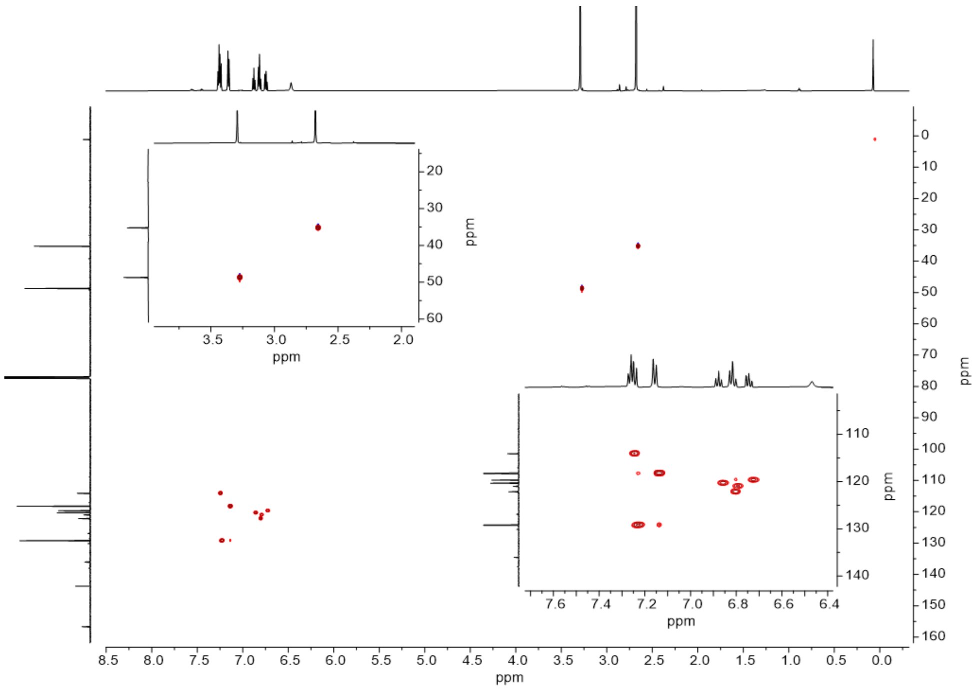

Figure S61: HSQC (^{1}H-^{13}C) of L4.

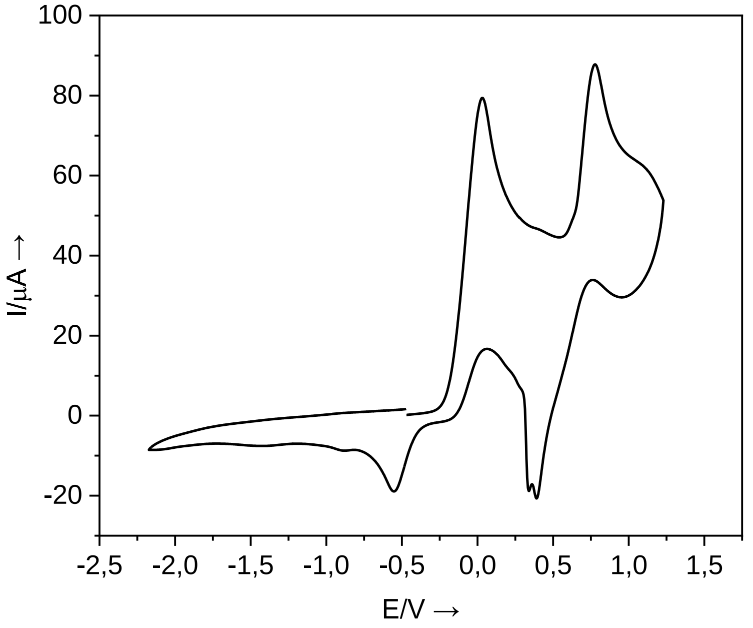

Figure S62: Cyclovoltammogram of L4 in dichloromethane ([*n*-Bu_4N][PF_6], 100 mV/s internally referenced to auf Fc/Fc+).

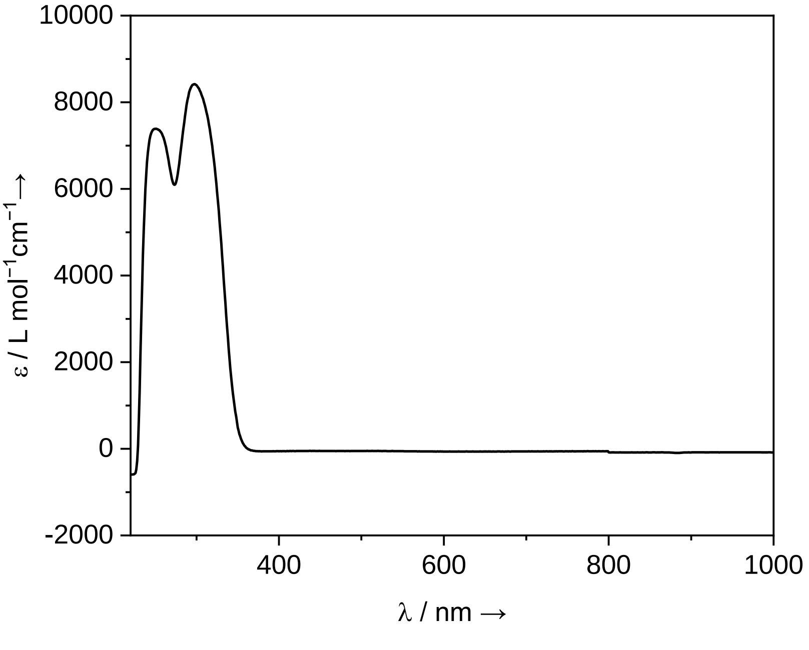

Figure S63: UV-Vis spectrum of L4 in DCM under argon.

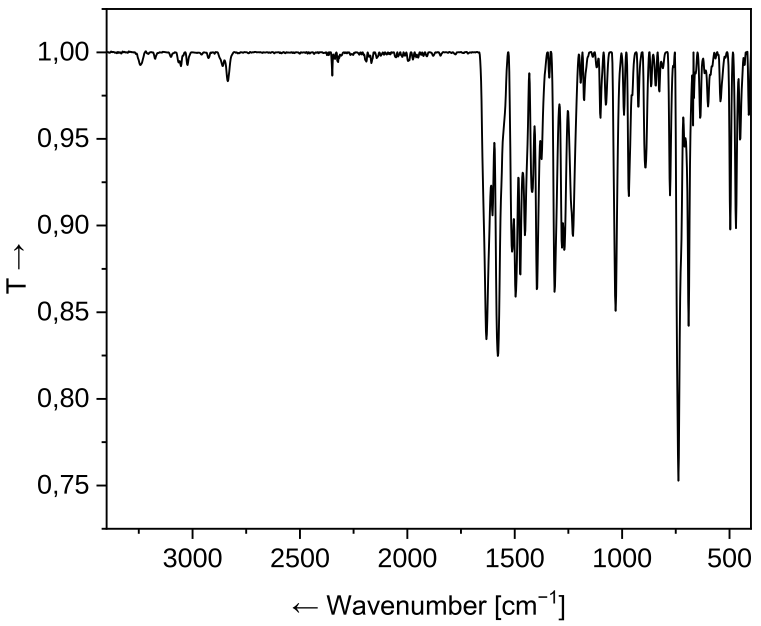

Figure S64: IR spectrum of L4.

Table S7: Molecular view of ligand L4, as well as crystallographic data. Most H atoms have been emitted for clarity. Displacement ellipsoids correspond to a 50% probability of occurrence. Colour coding: C dark-grey, N blue, H light-grey.

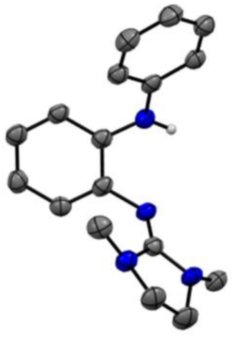

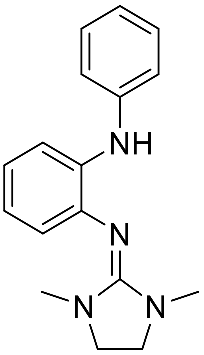

Empirical formula	$C_{17}H_{20}N_4$
Formula weight	280.37
Temperature/K	100.00
Crystal system	orthorhombic
Space group	Ibca
a/Å	16.259(4)
b/Å	18.710(4)
c/Å	20.146(4)

α/°	90
β/°	90
γ/°	90
Volume/Å^3	6128(2)
Z	16
ρ_{calc}g/cm^3	1.215
μ/mm^{-1}	0.075
F(000)	2400.0
Crystal size/mm^3	0.235 × 0.116 × 0.056
Radiation	MoKα (λ = 0.71073)
2Θ range for data collection/°	4.044 to 51.99
Index ranges	-20 ≤ h ≤ 20, -23 ≤ k ≤ 23, -24 ≤ l ≤ 24
Reflections collected	38557
Independent reflections	3015 [R_{int} = 0.2000, R_{sigma} = 0.0892]
Data/restraints/parameters	3015/0/192
Goodness-of-fit on F^2	1.057
Final R indexes [I>=2σ (I)]	R_1 = 0.0718, wR_2 = 0.1722
Final R indexes [all data]	R_1 = 0.1371, wR_2 = 0.2255
Largest diff. peak/hole / e Å^{-3}	0.25/-0.29

7.3 (*E*)-2-((3-methylthiazolidin-2-ylidene)amino)-*N*-phenylaniline (L6)

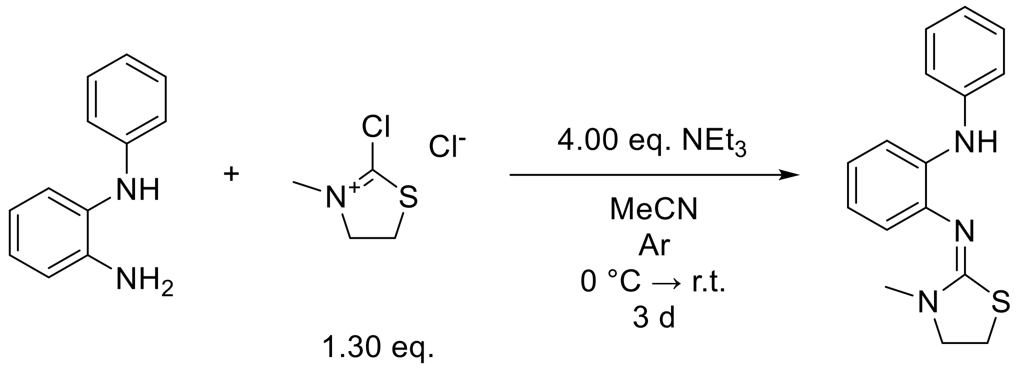

100 mg of N^1-phenylbenzene-1,2-diamine (1.00 eq., 542 µmol) and 1.20 eq. of activated urea (112 mg, 651 µmol) were dissolved in 2.00 mL of acetonitrile under ice cooling, and 302 µL of triethylamine (220 mg, 4.00 eq., 2.17 mmol) were added. The reaction mixture was stirred for 3 days. The solvent was filtered off and removed under vacuum. The residue was mixed with 4.00 mL of 15% degassed sodium hydroxide solution and extracted with 3 × 4.00 mL of diethyl ether. The combined organic phases were dried over magnesium sulfate. The product is obtained as a viscous liquid in a 44% yield (68 mg, 239 µmol).

^{1}H NMR (600 MHz, C_6D_6): δ = 7.49 (dd, *J* = 7.9, 1.5 Hz, 1 H, CH_{arom}), 7.38 (dd, *J* = 7.6, 1.6 Hz, 1 H, CH_{arom}), 7.16 – 7.08 (m, 4 H, CH_{arom}), 6.98 (td, *J* = 7.7, 1.7 Hz, 1 H, CH_{arom}), 6.93 (td, *J* = 7.5, 1.5 Hz, 1 H, CH_{arom}), 6.84 – 6.80 (m, 1 H, CH_{arom}), 6.73 (s, 1 H, N*H*), 2.60 (s, 3 H, CH_3), 2.52 (t, *J* = 6.9 Hz, 2 H, CH_2), 2.21 (t, *J* = 6.9 Hz, 2 H, CH_2) ppm.

^{13}C NMR (151 MHz, C_6D_6): δ = 159.81 ($C_{q,Guan}$), 143.88 ($C_{q,arom}$), 140.64 ($C_{q,arom}$), 137.31 ($C_{q,arom}$), 129.57 (CH_{arom}), 128.35 (CH_{arom}), 127.98 (CH_{arom}), 124.19 (CH_{arom}), 121.09 (CH_{arom}), 120.92 (CH_{arom}), 120.14 (CH_{arom}), 119.04(CH_{arom}), 114.77 (CH_{arom}), 52.20 (CH_2), 33.49 (CH_2), 26.36 (NCH_3) ppm.

MS: ESI ($M+H^+$): calculated: 284.1216, found: 284.1211.

CV:

Irreversible oxidation at: E_{Ox1}: 0.27 V

1. Reversible step at: $E_{1/2}$:0.64 V (E_{Ox1}: 0.75 V, E_{Red1}: 0.53 V)

UV-Vis (DCM, c = 4.23·10^{-5} mol·l^{-1}, d = 1 cm): λ_{max} (ε [l·mol^{-1}·cm^{-1}]) = 253 (4899), 294 (4817) nm.

IR (ATR): $\tilde{\nu}$ = 3358.57, 3041.27, 2923.99, 2852.90, 1744.22, 1671.26, 1611.23, 1580.09, 1544.11, 1494.79, 1456.69, 1418.67, 1391.67, 1309.08, 1285.65, 1237.47, 1155.52, 1103.37, 1072.73, 1041.86, 940.13, 867.27, 816.02, 615.55, 530.77, 495.87, 461.90 cm^{-1}.

Elemental analysis (%): L6 • 0.5 H_2O

calculated:	C: 65.72	H: 6.21	N: 14.37
found:	C: 65.66	H: 5.67	N: 14.12

Analytical data:

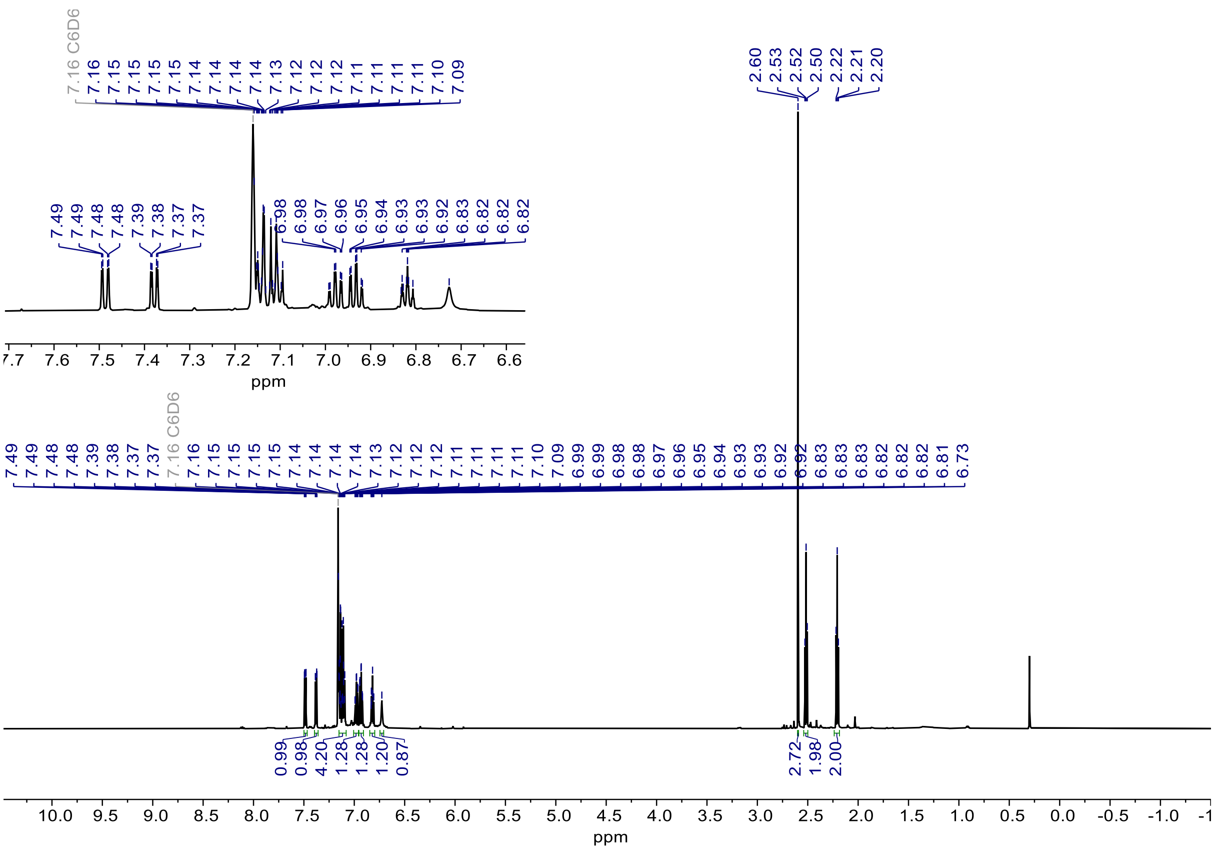

Figure S65: ^{1}H NMR spectrum (600 MHz, C_6D_6) of L6. The impurities at 0.30 ppm are due to grease.

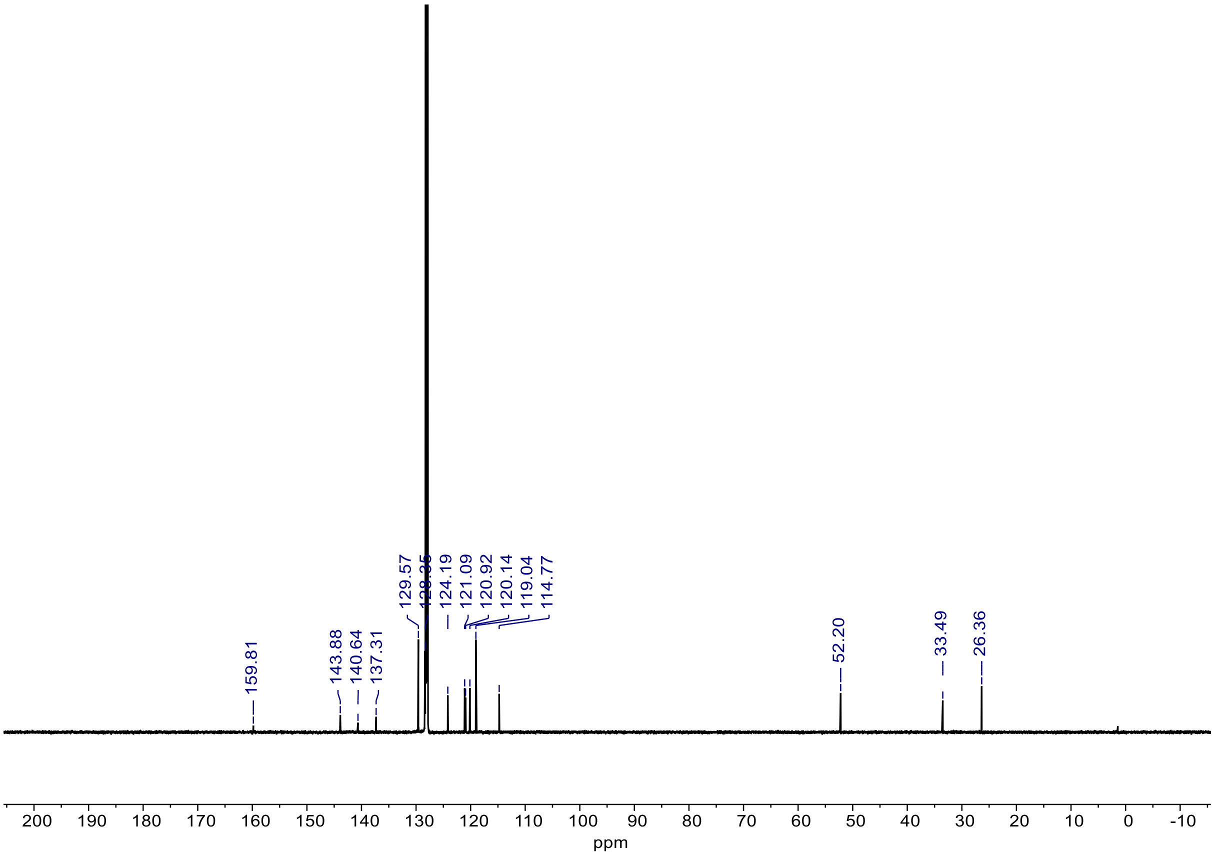

Figure S66: ^{13}C NMR spectrum (151 MHz, C_6D_6) of L6.

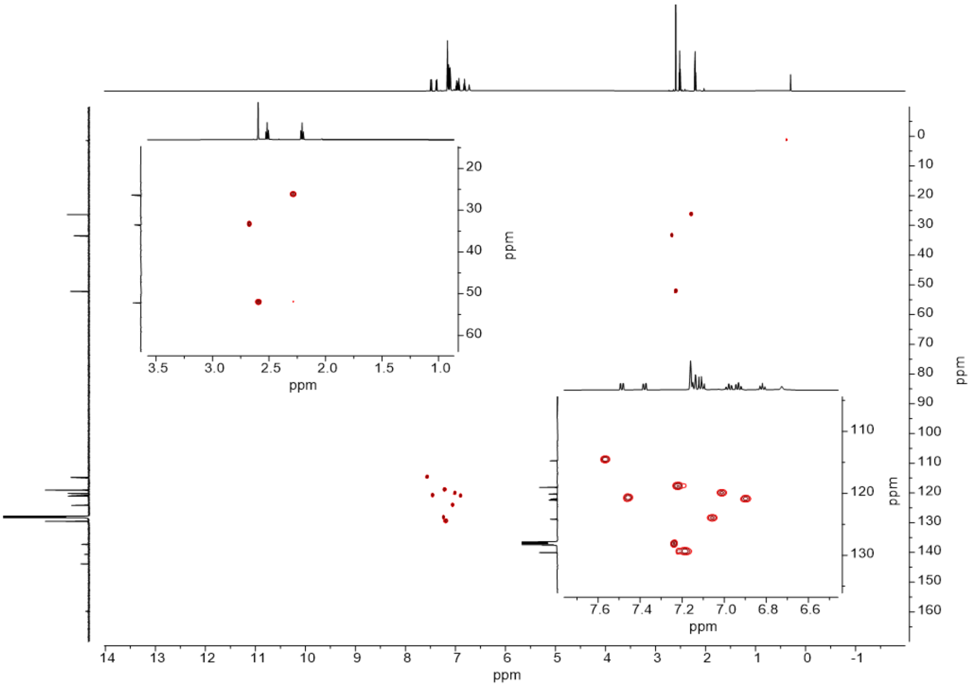

Figure S67: HSQC (^{1}H-^{13}C) spectrum of L6.

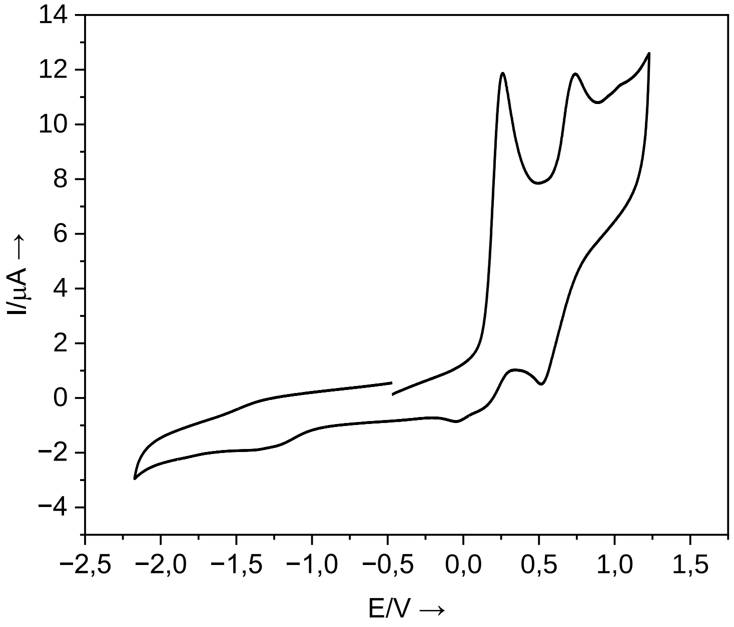

Figure S68: Cyclovoltammogram of L6 in dichloromethane ([*n*-Bu_4N][PF_6], 50 mV/s internally referenced to Fc/Fc+).

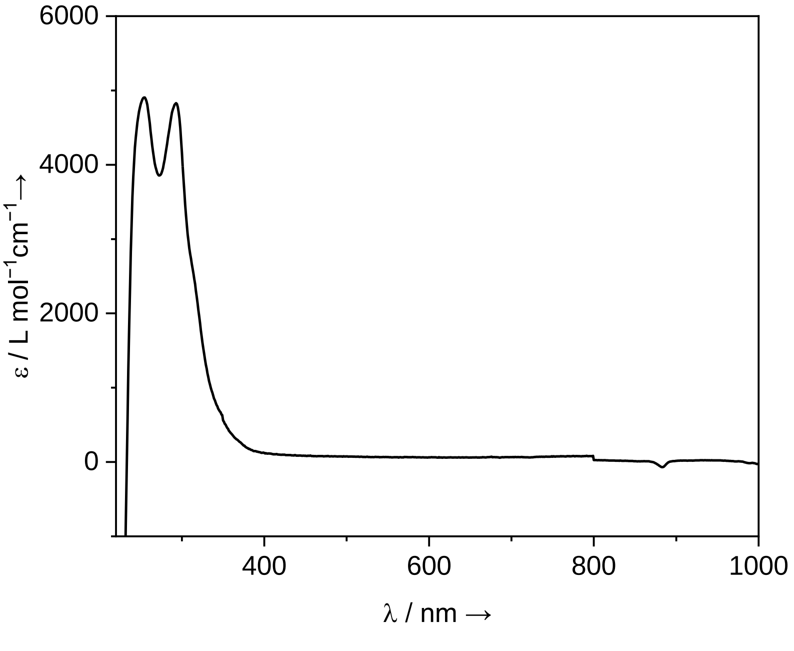

Figure S69: UV-Vis spectrum of L6 in DCM.

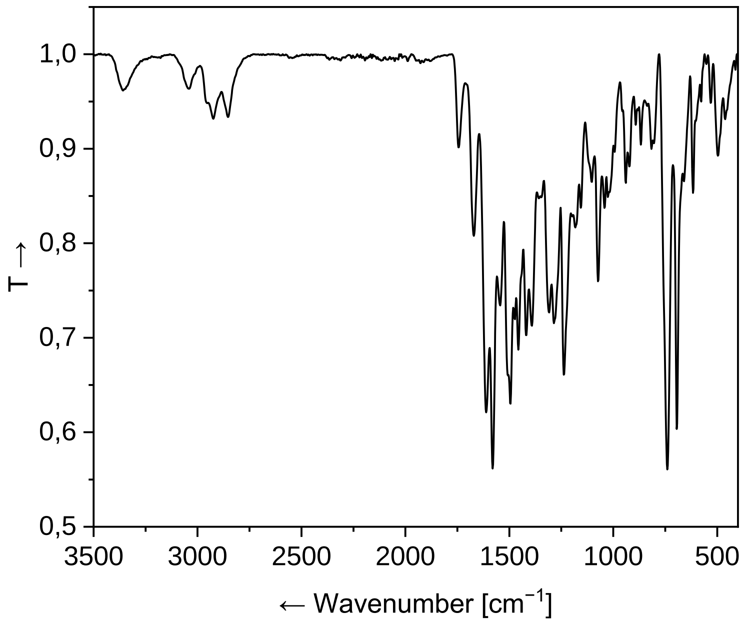

Figure S70: IR spectrum of L6.

7.4 2-((1,3-dimethyl-1,3-dihydro-2*H*-benzo[*d*]imidazol-2-ylidene)amino)-*N*-phenylaniline (L5)

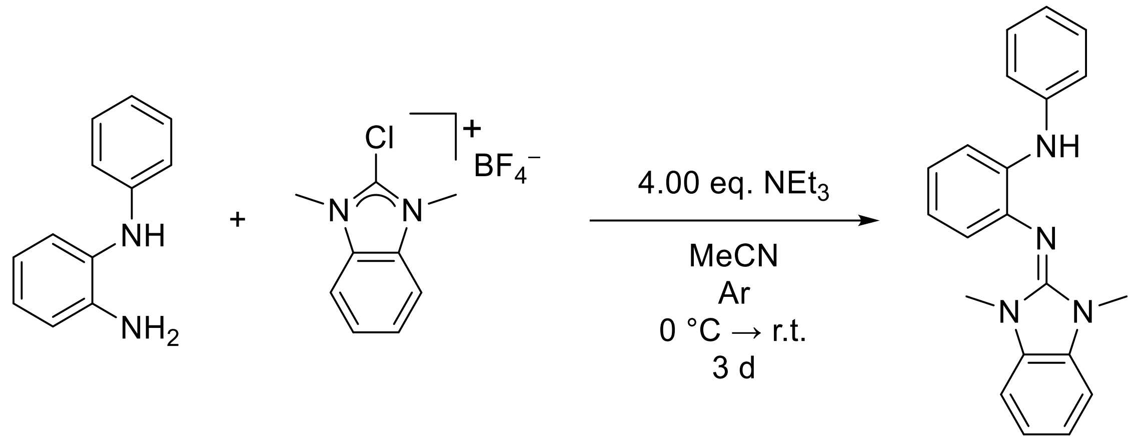

200 mg of N^1-phenylbenzene-1,2-diamine (1.00 eq., 1.09 mmol) and 1.20 eq. of activated urea (349 mg, 1.30 mmol) were dissolved in 2.00 mL of acetonitrile under ice cooling, and 605 µL of triethylamine (439 mg, 4.00 eq., 4.34 mmol) were added. The reaction mixture was stirred for 3 days. The solvent was filtered off and removed under vacuum. The residue was mixed with 5.00 mL of 15% degassed sodium hydroxide solution and extracted with 2 × 3.00 mL of diethyl ether. The combined organic phases were dried over magnesium sulfate. The product is obtained as a viscous liquid in 79% yield (284 mg, 861 µmol). Colourless crystals were obtained by layering a concentrated dichloromethane solution with *n*-hexane at -31 °C.

^{1}H NMR (600 MHz, CD_2Cl_2): δ = 7.28 (dd, *J* = 7.9, 1.4 Hz, 1 H, CH_{arom}), 7.27 – 7.22 (m, 2 H, 2×CH_{arom}), 7.18 – 7.15 (m, 2 H, 2×CH_{arom}), 7.06 – 7.02 (m, 2 H, 2×CH_{arom}), 6.90 (dd, *J* = 5.7, 3.2 Hz, 2 H, 2×CH_{arom}), 6.89 – 6.86 (m, 1 H, CH_{arom}), 6.86 – 6.81 (m, 2 H, 2×CH_{arom}), 6.77 (ddd, *J* = 7.7, 7.1, 1.3 Hz, 1 H, CH_{arom}), 6.59 (s, 1 H, N*H*), 3.27 (s, 6 H, 2×CH_3) ppm.

^{13}C NMR (151 MHz, CD_2Cl_2): δ = 148.21 ($C_{q,Guan}$), 143.81 ($C_{q,arom}$), 138.27 ($C_{q,arom}$), 136.57 ($C_{q,arom}$, 1C), 133.17 ($C_{q,arom}$), 129.52 (CH_{arom}), 121.43 (CH_{arom}), 121.19 (CH_{arom}), 121.17 (CH_{arom}), 120.72 (CH_{arom}), 119.80 (CH_{arom}), 118.36 (CH_{arom}), 114.08 (CH_{arom}), 106.90 (CH_{arom}), 30.38 (NCH_3) ppm.

CV:

Irreversible oxidation at: E_{Ox}: 0.02 V

1. Reversible step at: $E_{1/2}$:0.59 V (E_{Ox1}: 0.69 V, E_{Red1}: 0.50 V)

Irreversible reduction at: E_{Red}: 0.49 V

UV-Vis (DCM, c = 6.39·10^{-5} mol·l^{-1}, d = 1 cm): λ_{max} (ε [l·mol^{-1}·cm^{-1}]) = 259 (3310), 316 (3532), 432 (breit, 414), 524 (breit, 301) nm.

IR (ATR): ṽ = 3589.68, 3356.22, 2923.57, 1698.54, 1576.86, 1507.79, 1450.99, 1417.57, 1389.34, 1319.44, 1277.77, 1236.93, 1218.51, 1185.85, 1153.77, 1124.00, 1028.07, 1011.61, 946.99, 882.06, 830.87, 795.55, 759.51, 711.07, 694.17, 595.99, 582.54, 559.29, 521.51, 501.10, 464.86 cm^{-1}.

Elemental analysis: (%):

calculated:	C: 76.80	H: 6.14	N: 17.06
found:	C: 76.77	H: 6.08	N: 16.86

Analytical data:

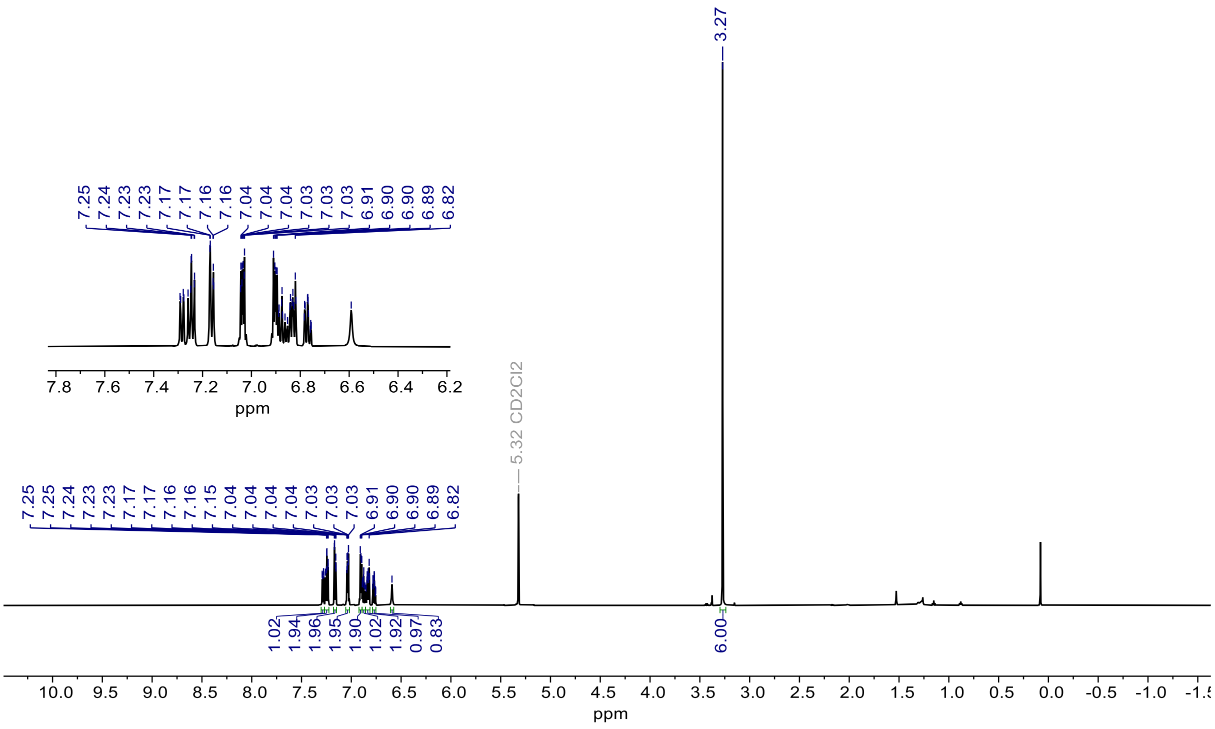

Figure S71: ^{1}H NMR spectrum (600 MHz, CD_2Cl_2) of L5. The impurities at 1.55 ppm are due to water and at 1.22 ppm, 0.88 ppm and 0.07 ppm are due to grease.

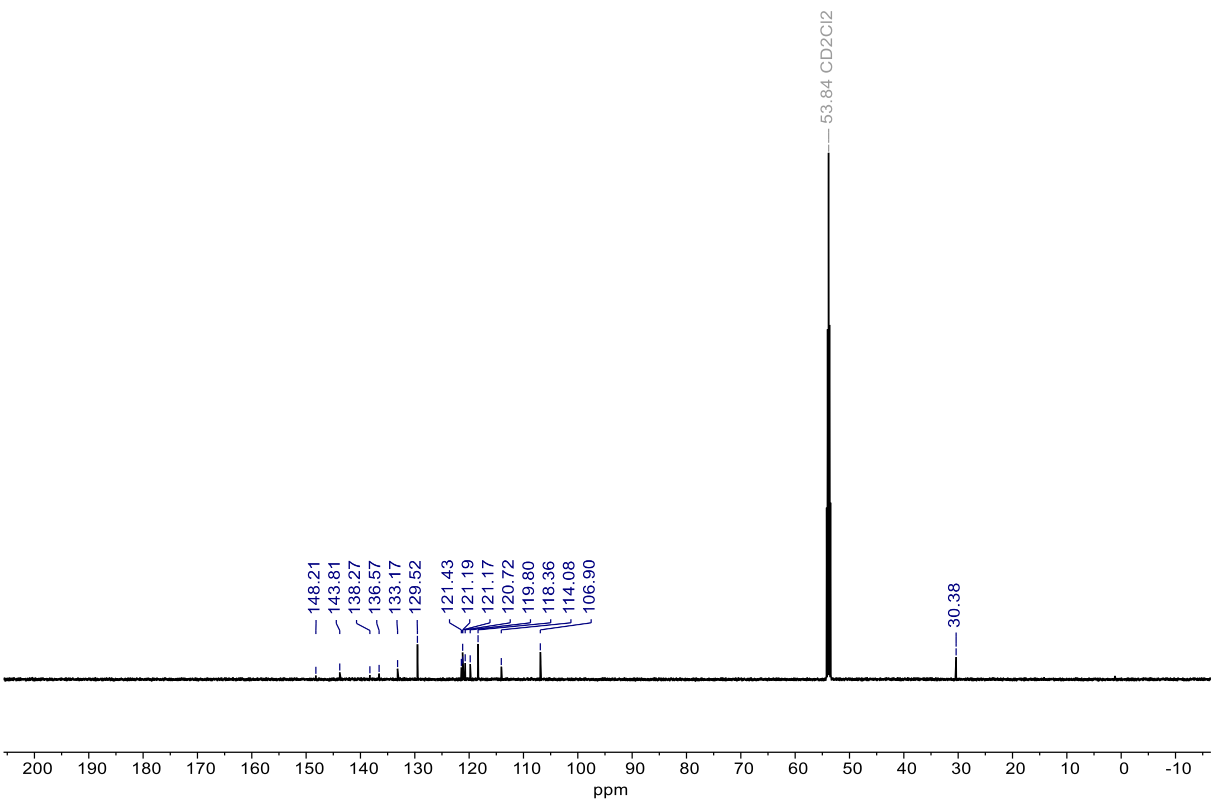

Figure S72: ^{13}C NMR spectrum (151 MHz, CD_2Cl_2) of L5.

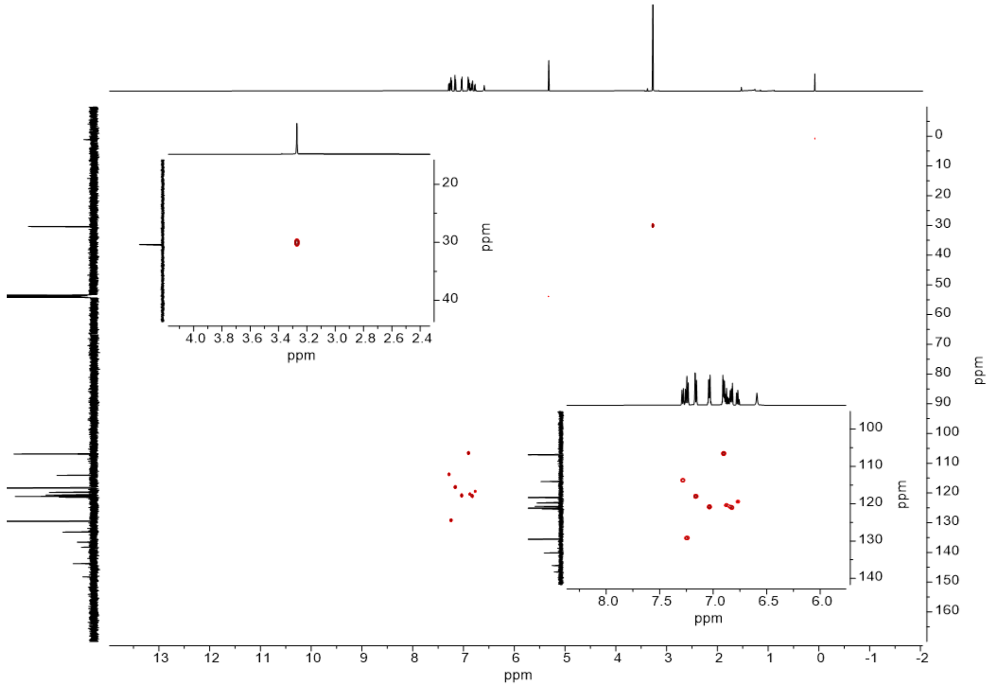

Figure S73: HSQC (^{1}H-^{13}C) spectrum of L5.

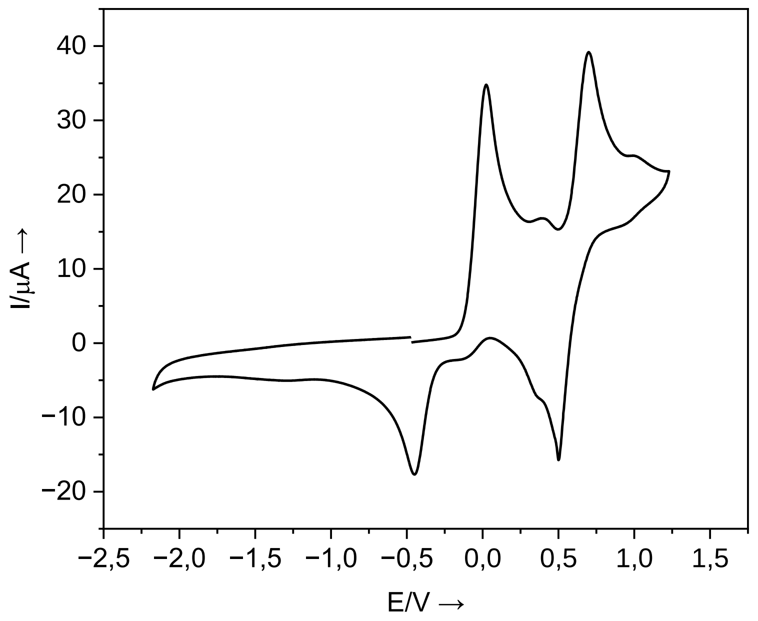

Figure S74: Cyclovoltammogram of L5 in dichloromethane ([*n*-Bu_4N][PF_6], 100 mV/s internally referenced to Fc/Fc+).

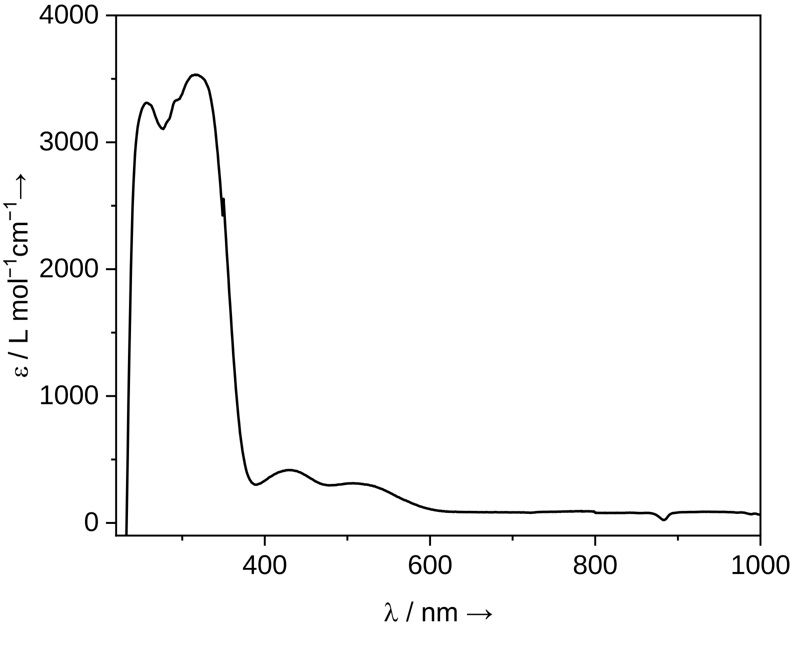

Figure S75: UV-Vis spectrum of L5 in DCM.

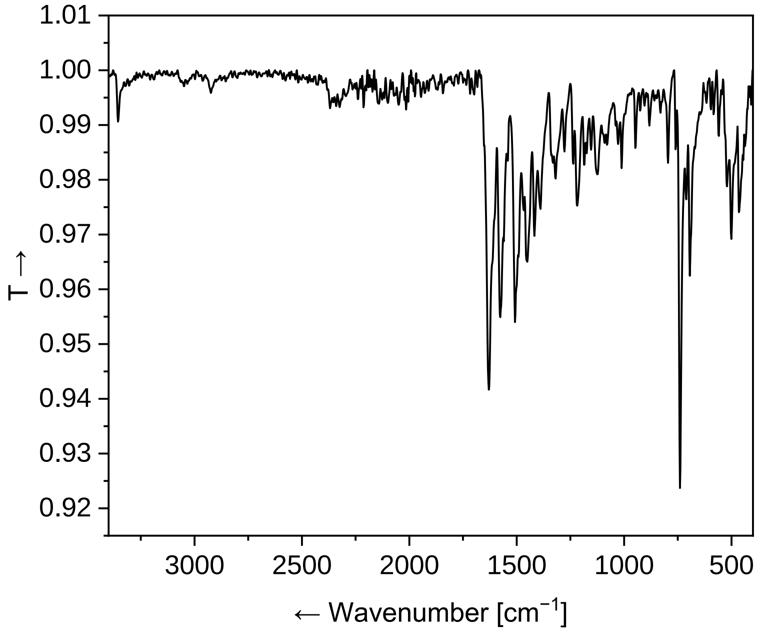

Figure S76: IR spectrum of L5.

Table S8: Molecular view of ligand L5, as well as crystallographic data. Most H atoms have been emitted for clarity. Displacement ellipsoids correspond to a 50% probability of occurrence. Colour coding: C dark-grey, N blue, H light-grey.

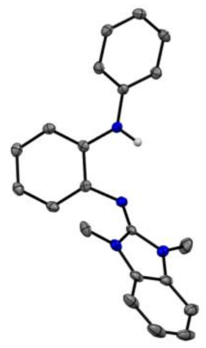

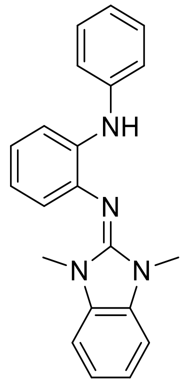

Empirical formula	$C_{21}H_{20}N_4$
Formula weight	328.41
Temperature/K	100.00
Crystal system	monoclinic
Space group	$P2_1/c$
a/Å	10.0614(5)
b/Å	14.4436(7)
c/Å	12.2939(7)
α/°	90
β/°	106.868(2)
γ/°	90
Volume/Å^3	1709.72(15)
Z	4
ρ_{calc}g/cm^3	1.276
μ/mm^{-1}	0.078
F(000)	696.0
Crystal size/mm^3	0.39 × 0.37 × 0.18
Radiation	MoKα (λ = 0.71073)
2Θ range for data collection/°	4.23 to 54.582
Index ranges	-12 ≤ h ≤ 12, -18 ≤ k ≤ 18, -15 ≤ l ≤ 15
Reflections collected	99698

Independent reflections	3802 [R_{int} = 0.0618, R_{sigma} = 0.0185]
Data/restraints/parameters	3802/0/232
Goodness-of-fit on F^2	1.046
Final R indexes [I>=2σ (I)]	R_1 = 0.0478, wR_2 = 0.1229
Final R indexes [all data]	R_1 = 0.0534, wR_2 = 0.1271
Largest diff. peak/hole / e $Å^{-3}$	0.33/-0.29

8 Secondary (Methyl residue)

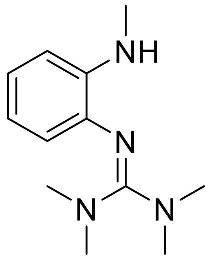

L7

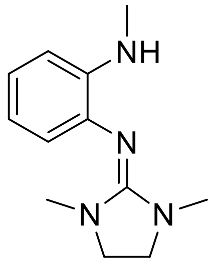

L8

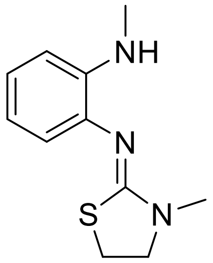

L9

Scheme S5: Overview over the ligands with L7, L8 and L9 with methylamino group.

8.1 1,1,3,3-tetramethyl-2-(2-(methylamino)phenyl)guanidine (L7)

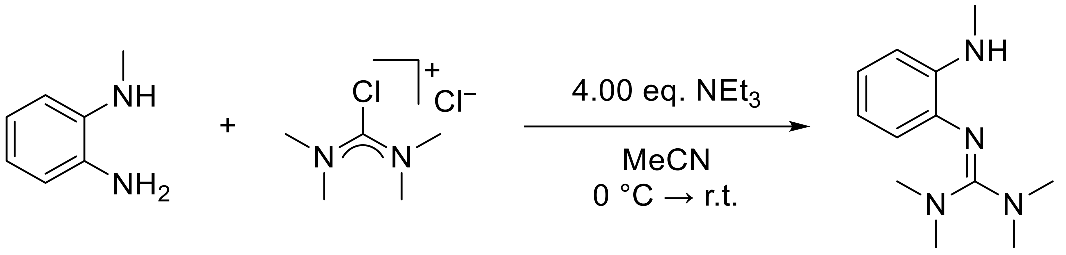

0.29 mL N^1-methylbenzene-1,2-diamine (315 mg, 2.58 mmol, 1.00 eq.) and 529 mg of 2-chloro-*N,N,N',N'*-tetramethylformamidinium chloride (3.09 mmol, 1.20 eq.) were dissolved in 2.00 mL of acetonitrile and cooled to 0 °C using an ice bath. 1.45 mL (10.31 mmol, 4.00 eq.) of triethylamine were added and the reaction mixture was stirred overnight and slowly warmed to room temperature. The solution was separated and the solvent was then removed under a fine vacuum. The residue was taken up in 8.00 mL of degassed NaOH (15%) and the aqueous phase was extracted with 4 × 3.00 mL of diethyl ether. The solvent from the collected organic phases was removed under reduced vacuum. The crude product was dissolved in 8.00 mL of DCM and the organic phase was extracted with 2 × 4.00 mL of degassed, deionized water and then washed with 1 × 4.00 mL of degassed NaOH (15%). The separated organic phase was dried over $MgSO_4$ and the solvent was again removed under reduced vacuum. 1,1,3,3-Tetramethyl-2-(2-(methylamino)phenyl)guanidine (341 mg, 1.55 mmol, 60%) was initially obtained as a viscous liquid, which tended to form colourless crystals spontaneously.

^{1}H NMR (600 MHz, CD_2Cl_2): δ = 6.77 (td, *J* = 7.6, 1.5 Hz, 1 H, $CH_{arom.}$), 6.53 – 6.47 (m, 2 H, $CH_{arom.}$), 6.28 (dd, *J* = 7.5, 1.4 Hz, 1 H, CH_{arom}), 4.51 (s, 1 H, N*H*), 2.82 (d, *J* = 5.4 Hz, 3 H, $NHCH_3$), 2.64 (s, 12 H, 4×CH_3) ppm.

^{13}C NMR (151 MHz, CD_2Cl_2): δ = 160.45 ($C_{q,Guan}$), 142.88 ($C_{q,arom}$), 138.34 ($C_{q,arom}$), 121.38 (d, *J* = 14.1 Hz, CH_{arom}), 119.24 (d, *J* = 7.6 Hz, CH_{arom}), 116.44 (CH_{arom}), 108.73 (CH_{arom}), 39.67 (NCH_3), 30.76 (p, *J* = 7.2 Hz, $NHCH_3$) ppm.

MS: ESI$^+$ (in DCM) $[M+H]^+$: m/z = calculated: 221.1761, found: 221.1754.

UV-Vis (DCM, c = 1.634·10^{-5} mol·l^{-1}, d = 1 cm): λ_{max} (ε [l·mol^{-1}·cm^{-1}]) = 272 (2694), 319 nm (2723) nm

CV:

Irreversible oxidation at: E_{Ox1}: -0.02 V, E_{Ox2}: 0.80 V.

Irreversible reduction at: E_{Red1}: -0.34 V, E_{Red2}: -0.77 V.

IR (ATR): $\tilde{\nu}$ = 3269, 2917, 2877, 2856, 2801, 1567, 1498, 1421, 1323, 1269, 1233, 1191, 1091, 1063, 1035, 1016, 925, 910, 853, 780, 740, 697, 625, 550, 511, 458 cm^{-1}.

Elemental analysis (%):

calculated:	C: 65.42	H: 9.15	N: 25.43
found:	C: 65.42	H: 9.22	N: 25.55.

Analytical data:

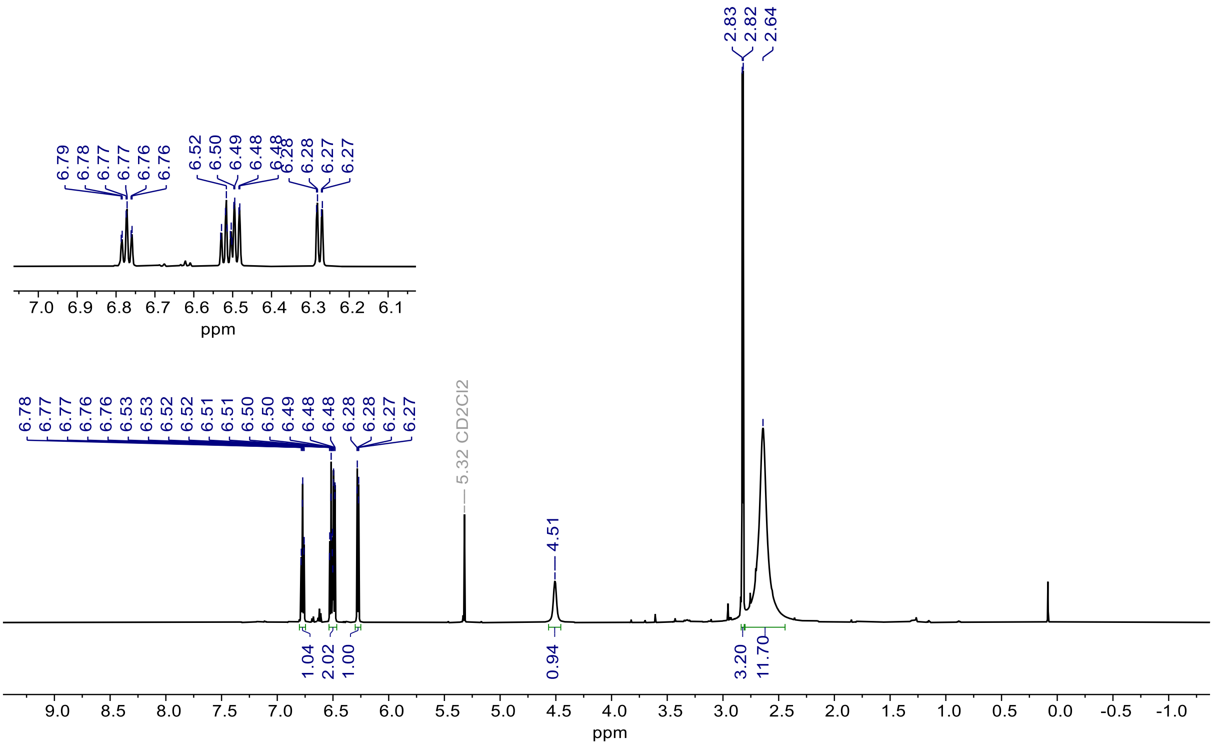

Figure S77: ^{1}H NMR spectrum (600 MHz, CD_2Cl_2) of L7. The impurities at 0.1 ppm are due to grease. At 1.15 ppm and 3.38 ppm, these are solvent residues from Et_2O. At 2.91 ppm is deactivated urea.

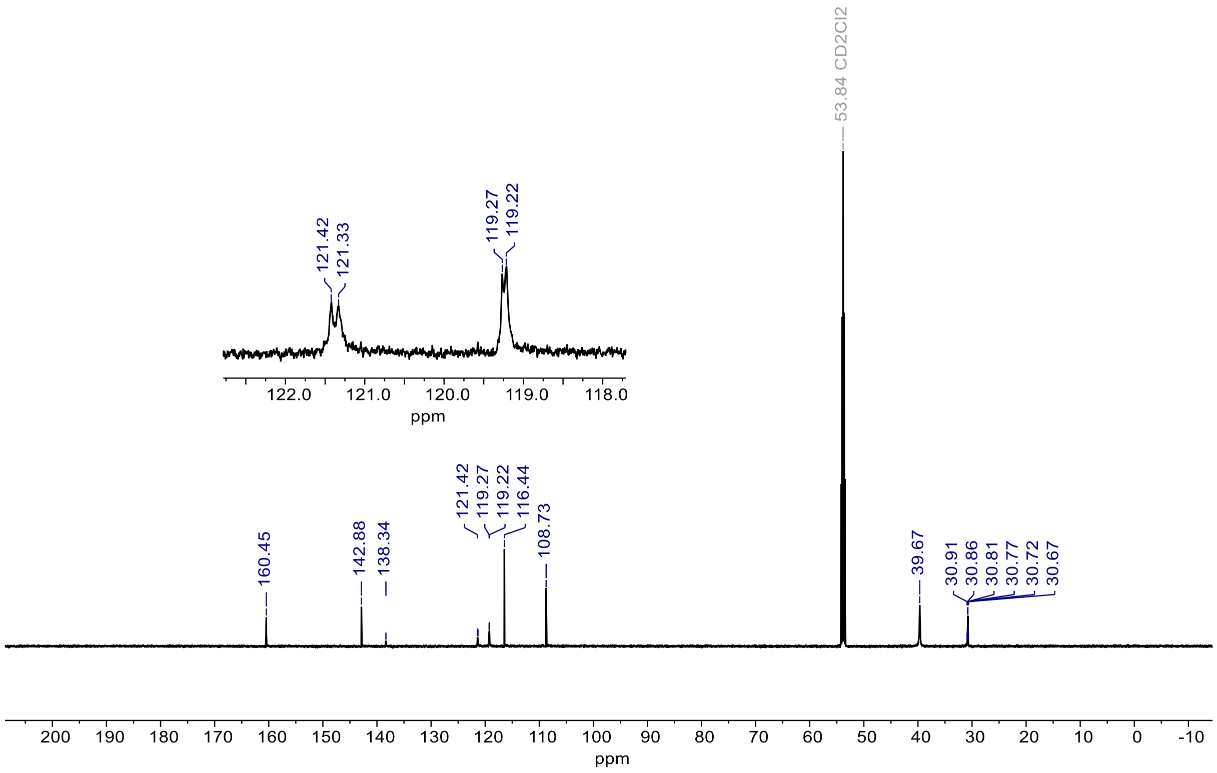

Figure S78: ^{13}C NMR spectrum (151 MHz, CD_2Cl_2) of L7.

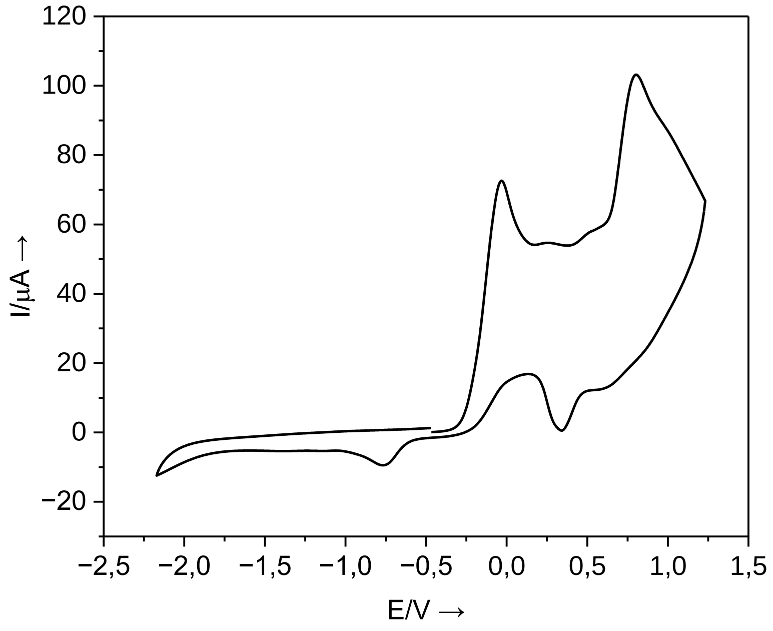

Figure S79: Cyclovoltammogram of L7 in dichloromethane ([n-Bu_4N][PF_6], 100 mV/s internally referenced to Fc^+/Fc).

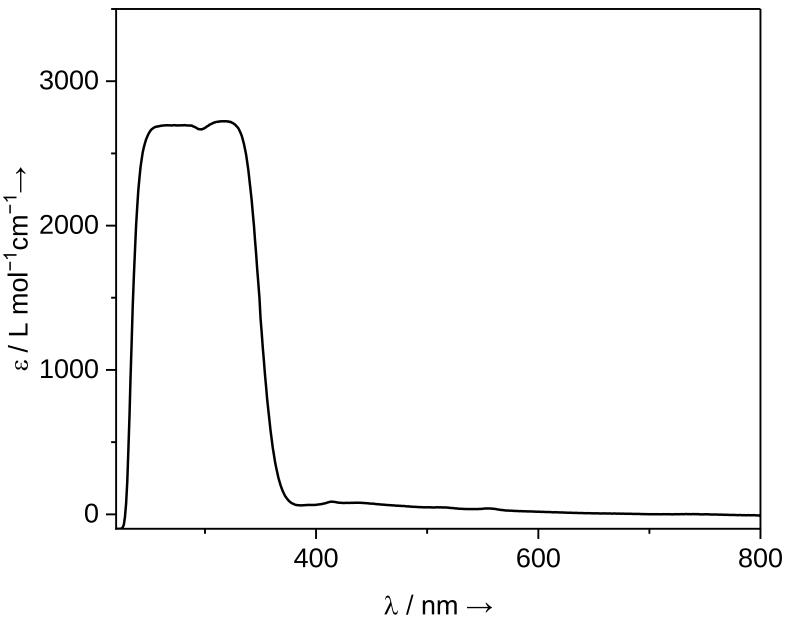

Figure S80: UV-Vis absorption spectrum of L7 measured in DCM.

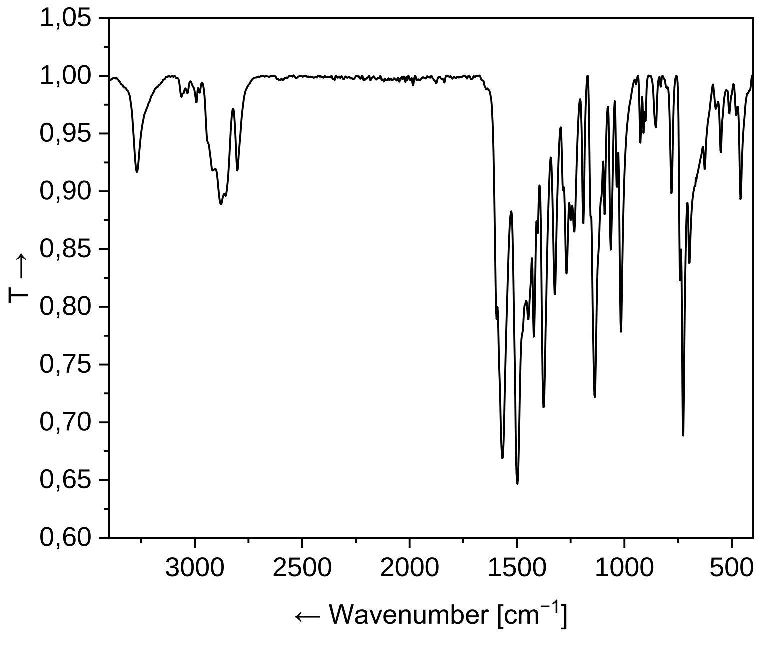

Figure S81: IR spectrum of L7.

Table S9: Molecular view of ligand L7, as well as crystallographic data. Most H atoms have been emitted for clarity. Displacement ellipsoids correspond to a 50% probability of occurrence. Colour coding: C dark-grey, N blue, H light-grey.

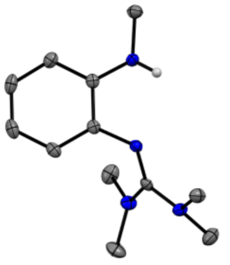

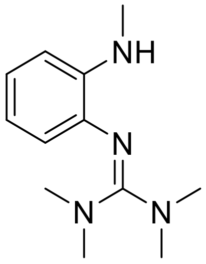

Empirical formula	$C_{12}H_{20}N_4$
Formula weight	220.32
Temperature/K	100.00
Crystal system	monoclinic
Space group	$P2_1/n$
a/Å	11.5662(14)
b/Å	18.089(2)
c/Å	12.2738(14)
α/°	90
β/°	95.176(4)
γ/°	90
Volume/Å^3	2557.5(5)
Z	8
ρ_{calc}g/cm^3	1.144
μ/mm^{-1}	0.072
F(000)	960.0
Crystal size/mm^3	0.44 × 0.37 × 0.21
Radiation	MoKα (λ = 0.71073)

2Θ range for data collection/°	4.022 to 52.99
Index ranges	-14 ≤ h ≤ 14, -22 ≤ k ≤ 22, -15 ≤ l ≤ 15
Reflections collected	89920
Independent reflections	5300 [R_{int} = 0.0922, R_{sigma} = 0.0410]
Data/restraints/parameters	5300/2/307
Goodness-of-fit on F^2	1.026
Final R indexes [I>=2σ (I)]	R_1 = 0.0408, wR_2 = 0.1027
Final R indexes [all data]	R_1 = 0.0474, wR_2 = 0.1098
Largest diff. peak/hole / e $Å^{-3}$	0.27/-0.20

8.2 2-((1,3-dimethylimidazolidin-2-ylidene)amino)-*N*-methylaniline (L8)

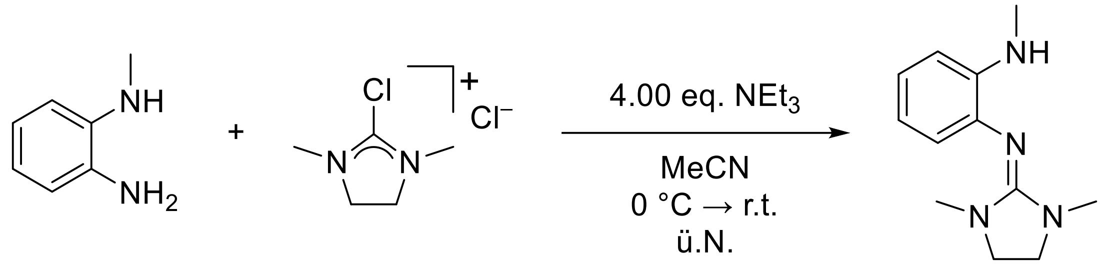

1.20 eq.

198 mg N^1-methylbenzene-1,2-diamine (184 µml, *d* = 1.07 g/ml, 1.62 mmol, 1.00 eq.) and 1.20 eq. activated urea (329 mg, 1.94 mmol) were dissolved in 2.00 mL of acetonitrile under ice cooling and 904 µL of triethylamine (656 mg, 4.00 eq., 6.48 mmol) were added. The reaction mixture was stirred overnight. The solvent was filtered off and removed *in vacuo*. The residue was mixed with 4.00 mL of 15% degassed sodium hydroxide solution and extracted with 2 × 3.00 mL of diethyl ether. The combined organic phases were dried over magnesium sulfate. The product is obtained as a viscous liquid in 70% yield (248 mg, 1.13 mmol).

^{1}H NMR (400 MHz, $CDCl_3$): δ = 6.84 (td, *J* = 7.6, 1.5 Hz, 1 H, CH_{arom}), 6.70 (dd, *J* = 7.6, 1.5 Hz, 1 H, CH_{arom}), 6.60 – 6.52 (m, 2 H, CH_{arom}), 4.34 (s, 1 H, N*H*), 3.26 (s, 4 H, 2×CH_2), 2.87 – 2.83 (m, 3 H, $NHCH_3$), 2.64 (s, 6 H, 2×CH_3) ppm.

2-((1,3-dimethylimidazolidin-2-ylidene)amino)-*N*-methylaniline (L8):

^{13}C NMR (101 MHz, $CDCl_3$): δ = 156.67 ($C_{q,Guan}$), 142.91 ($C_{q,arom}$), 135.93 ($C_{q,arom}$), 121.71 (CH_{arom}), 120.93 (CH_{arom}), 116.41 (CH_{arom}), 109.01 (CH_{arom}), 48.75 (NCH_2), 35.17 (NCH_3), 31.02 ($NHCH_3$) ppm.

N^1,N^2-dimethyl-N^1-(1-methyl-1*H*-benzo[*d*]imidazol-2-yl)ethane-1,2-diamine (rearranged species):

^{13}C NMR (101 MHz, $CDCl_3$): δ = 141.46 ($C_{q,arom}$),121.77 (CH_{arom}), 121.07 (CH_{arom}), 117.82 (CH_{arom}), 108.43 (CH_{arom}), 54.09 (NCH_2), 49.28 (NCH_2), 39.80 (NCH_3), 36.62 (NCH_3), 30.87 ($NHCH_3$) ppm. Carbon atoms are missing due to low sensitivity.

Elemental analysis (%):

calculated:	C: 66.02	H: 8.31	N: 25.67
found:	C: 65.66	H: 7.81	N: 25.26

Analytical data:

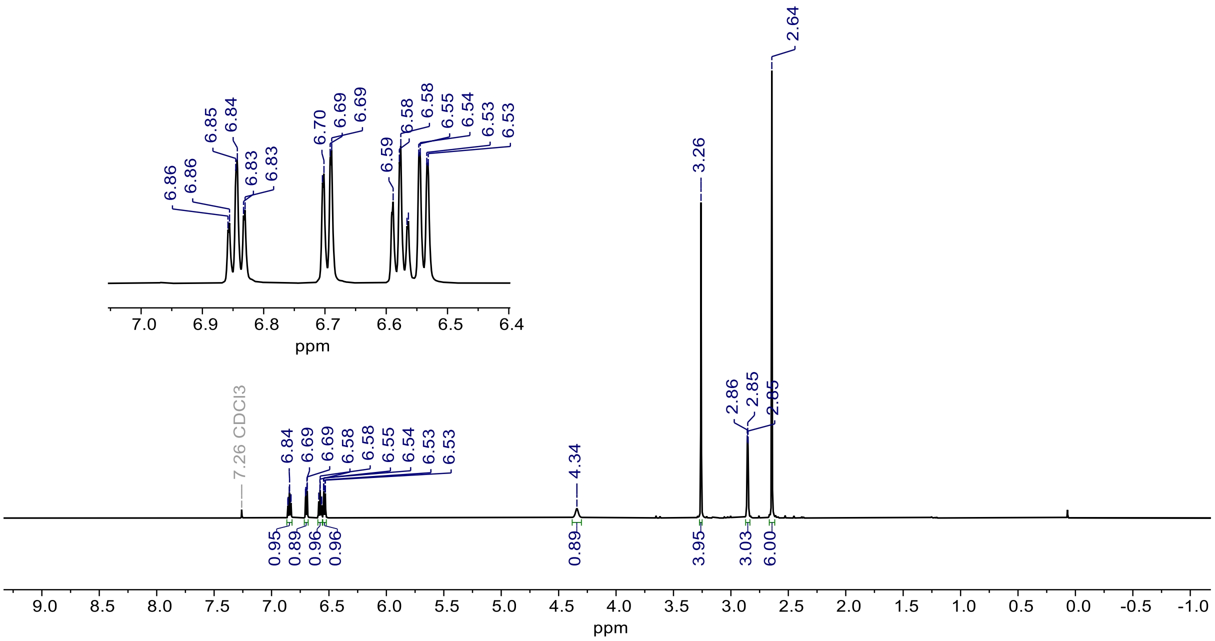

Figure S82: ^{1}H NMR spectrum (400 MHz, $CDCl_3$) of L8.

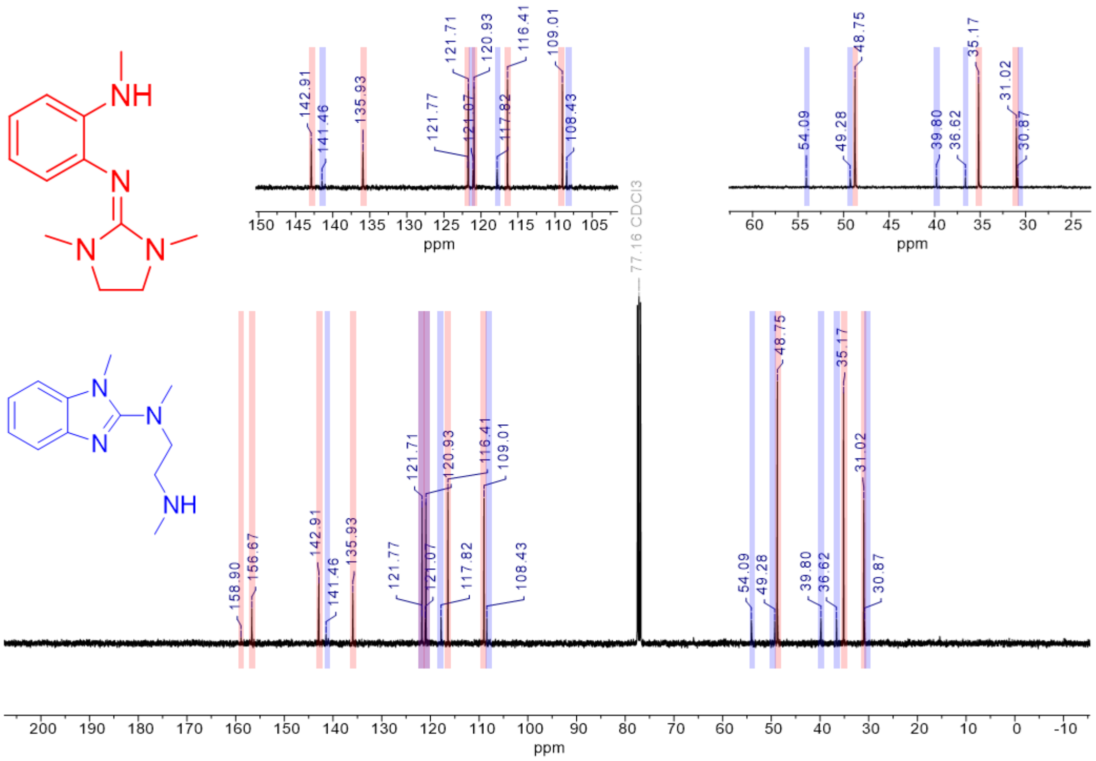

Figure S83: ^{13}C NMR spectrum (101 MHz, $CDCl_3$) of the two species 2-((1,3-dimethylimidazolidin-2-ylidene)amino)-*N*-methylaniline (red) and *N*[1],*N*[2]-dimethyl-*N*[1]-(1-methyl-1*H*-benzo[*d*]imidazol-2-yl)ethane-1,2-diamine (blue).

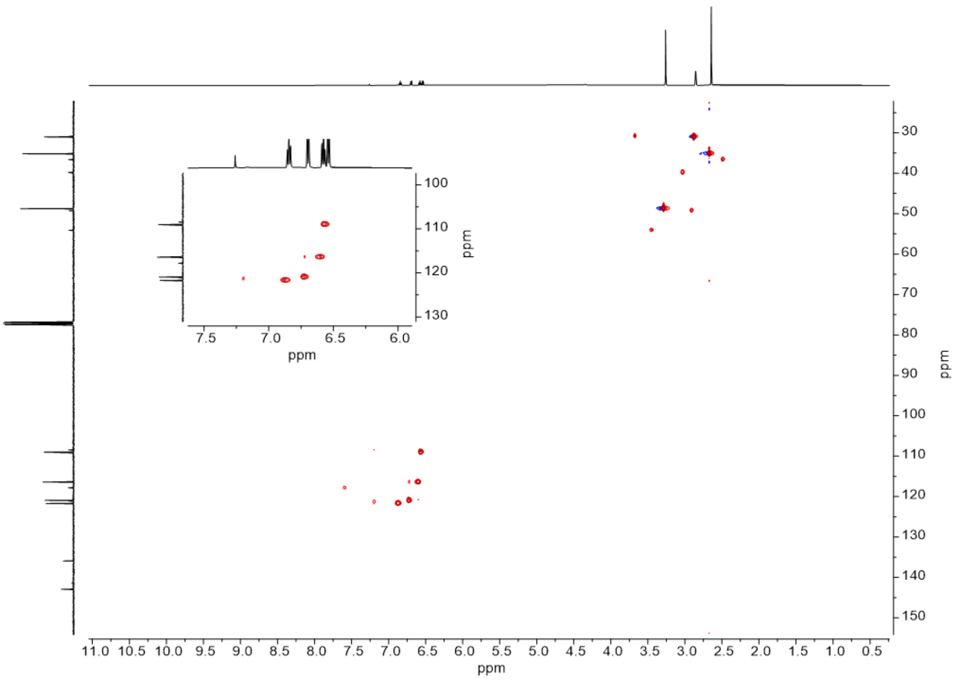

Figure S84: HSQC (^1H-^{13}C) spectrum of L8.

8.3 (*E*)-*N*-methyl-2-((3-methylthiazolidin-2-ylidene)amino)aniline (L9)

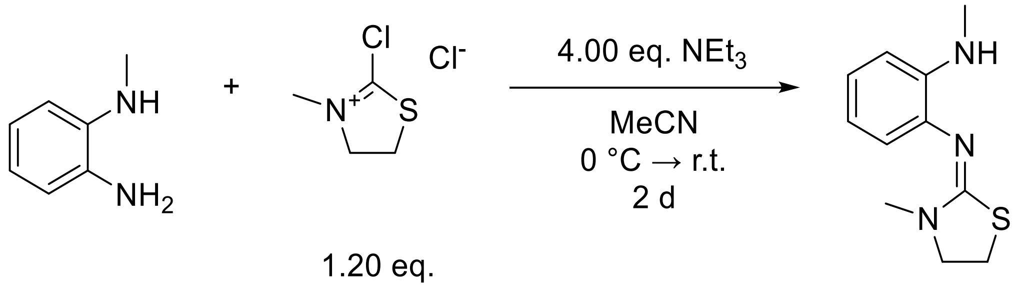

198 mg *N*[1]-methylbenzene-1,2-diamine (187 µml, *d* = 1.07 g/ml, 1.64 mmol, 1.00 eq.) and 1.20 eq. activated urea (338 mg, 1.64 mmol) were dissolved in 4.00 mL of acetonitrile under ice cooling and 913 µL of triethylamine (703 mg, 4.00 eq., 6.95 mmol) were added. The reaction mixture was stirred overnight. The solvent was filtered off and removed *in vacuo*. The residue was mixed with 4.00 mL of 15% degassed sodium hydroxide solution and extracted with 2 × 3.00 mL of diethyl ether. The combined organic phases were dried over magnesium sulfate. The product is obtained as a dark yellow viscous liquid in 25% yield (92.0 mg, 416 µmol).

^{1}H NMR (400 MHz, $CDCl_3$): δ = 7.00 (td, *J* = 7.7, 1.5 Hz, 1 H, CH_{arom}), 6.85 (dd, *J* = 7.6, 1.5 Hz, 1 H, CH_{arom}), 6.66 – 6.57 (m, 2H, CH_{arom}), 4.32 (s, 1 H, N*H*), 3.59 (t, *J* = 6.9 Hz, 2 H, CH_2), 3.14 (t, *J* = 6.9 Hz, 2 H, CH_2), 3.07 (s, 3 H, $NHCH_3$), 2.85 (s, 3 H, CH_3) ppm.

^{13}C NMR (101 MHz, $CDCl_3$): δ = 160.57 ($C_{q,Guan}$), 142.89 ($C_{q,arom}$), 138.34 ($C_{q,arom}$), 124.45 (CH_{arom}), 119.90 (CH_{arom}), 116.40 (CH_{arom}), 109.29 (CH_{arom}), 53.17 (CH_2), 34.02 ($NHCH_3$), 30.84 (NCH_3), 26.83 (CH_2) ppm.

UV-Vis (DCM, $c = 2.26\cdot10^{-5}$ mol·l^{-1}, *d* = 1 cm): λ_{max} (*ε* [l·mol^{-1}·cm^{-1}]) = 245 nm (6338), 311 nm (3083) nm

CV:

Irreversible oxidation at: E_{Ox}: 0.69 V

1. Reversible step at: $E_{1/2}$:0.065 V (E_{Ox1}: 0.13 V, E_{Red1}: 0.00 V)

Irreversible reduction at: E_{Red}: −0.84 V

IR (ATR): ṽ = 3402.24, 3056.12, 3038.67, 2862.05, 2807.47, 1609.19, 1585.25, 1503.49, 1472.95, 1439.05, 1420.47, 1384.45, 1325.54, 1307.20, 1278.23, 1236.74,

1207.65, 1183.11, 1158.67, 1120.15, 1071.87, 1035.21, 1015.43, 992.00, 938.76, 912.88, 878.85, 831.52, 736.19, 703.81, 665.59, 619.41, 604.07, 532.84, 449.52 cm^{-1}.

Elemental analysis (%): L9 • 0.5 MeCN

calculated:	C: 59.60	H: 6.88	N: 20.27
found:	C: 59.69	H: 6.65	N: 20.71

Analytical data:

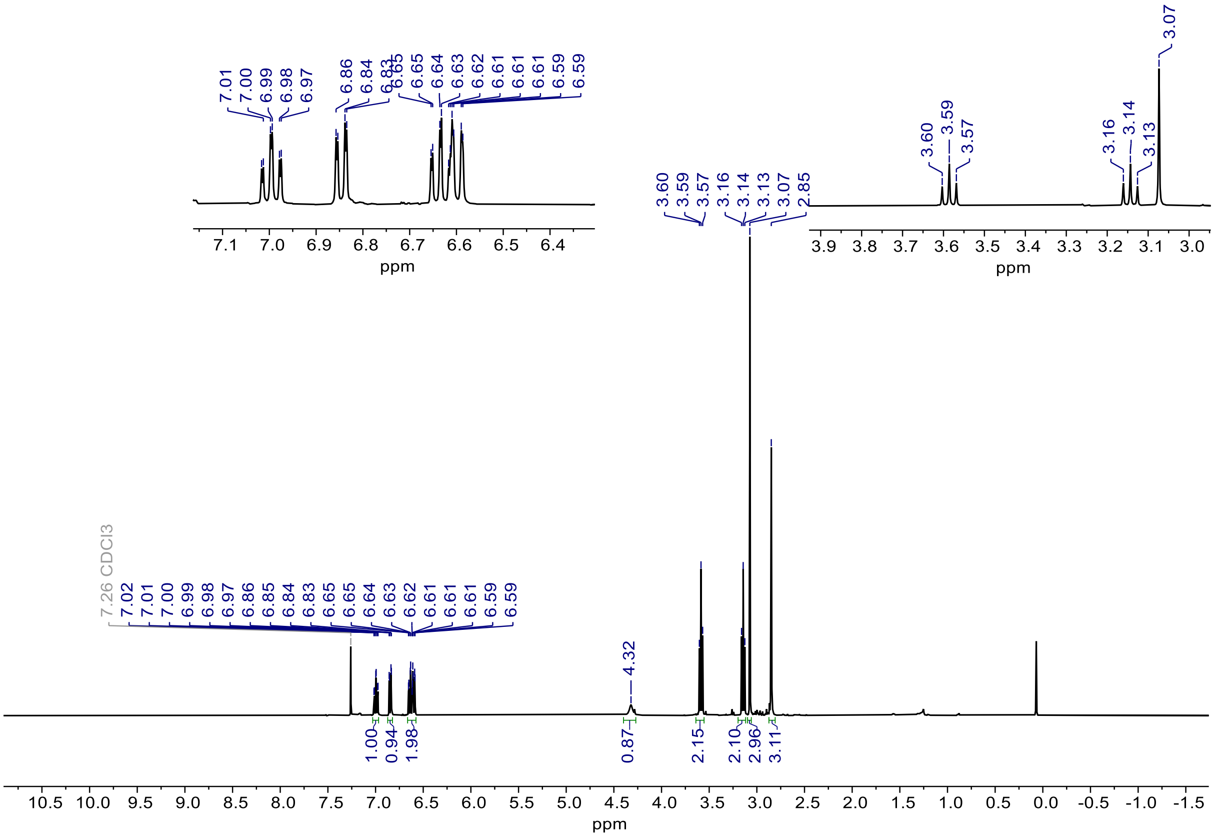

Figure S85: ^{1}H NMR spectrum (400 MHz, $CDCl_3$) of L9. The impurity at 0.07 ppm is due to grease.

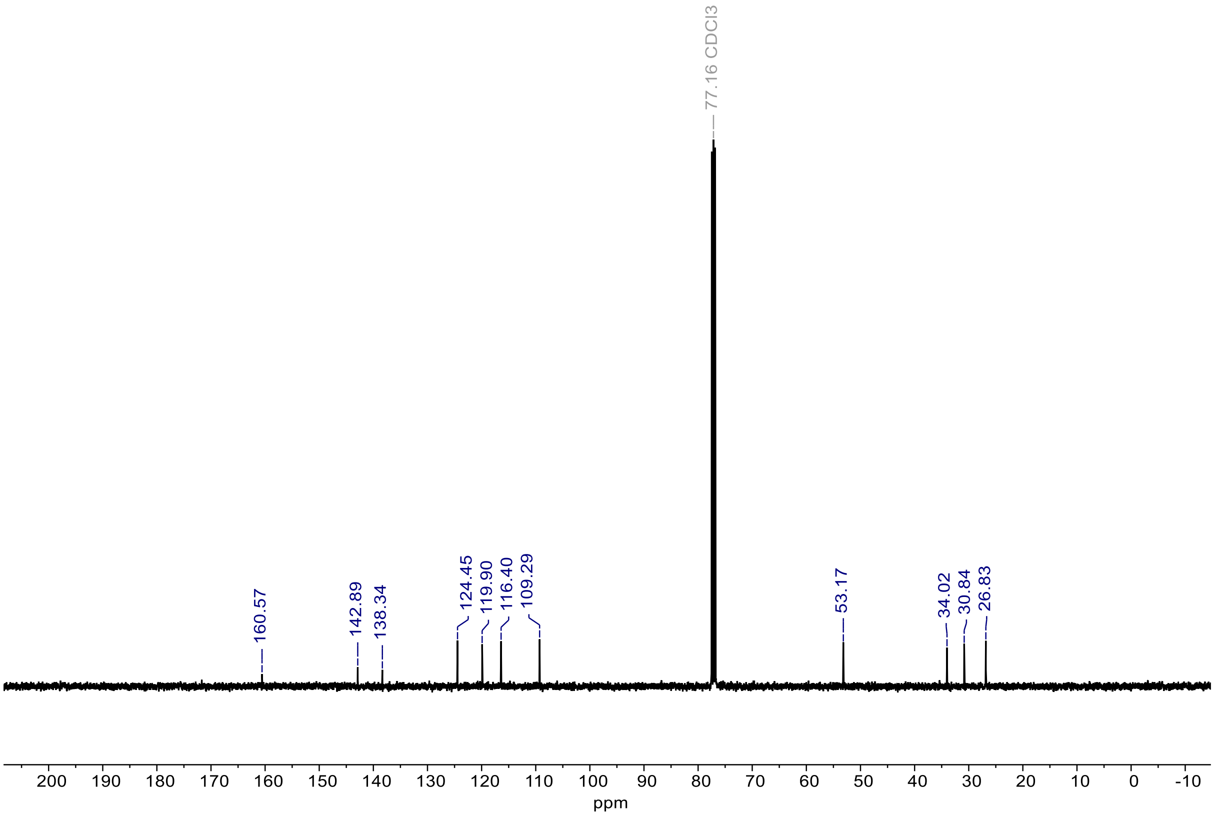

Figure S86: ^{13}C NMR spectrum (101 MHz, $CDCl_3$) of L9.

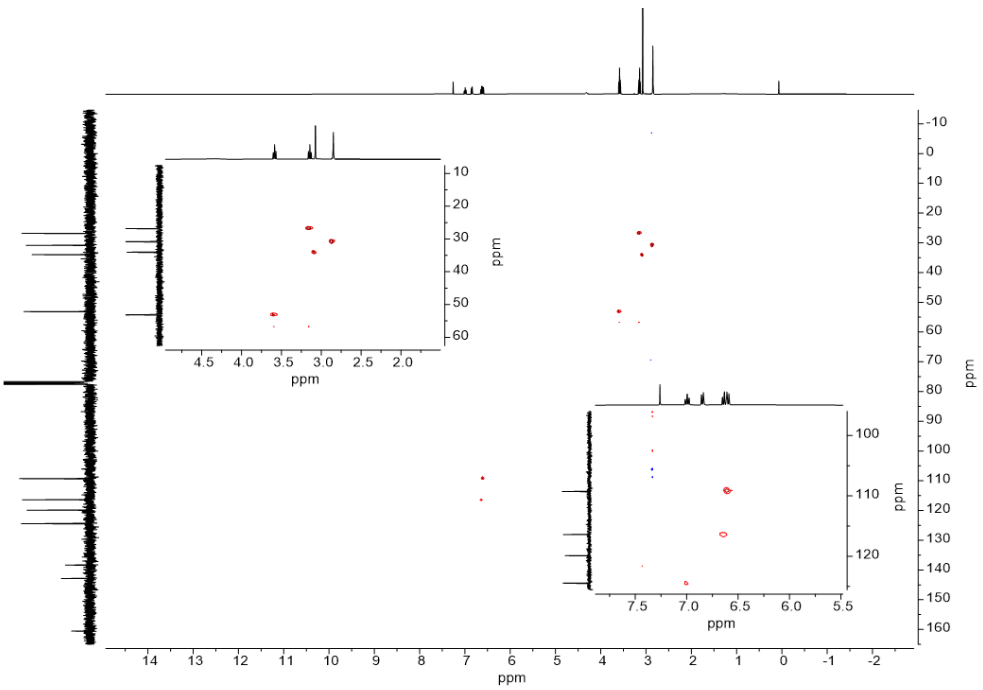

Figure S87: HSQC (^{1}H-^{13}C) spectrum of L9.

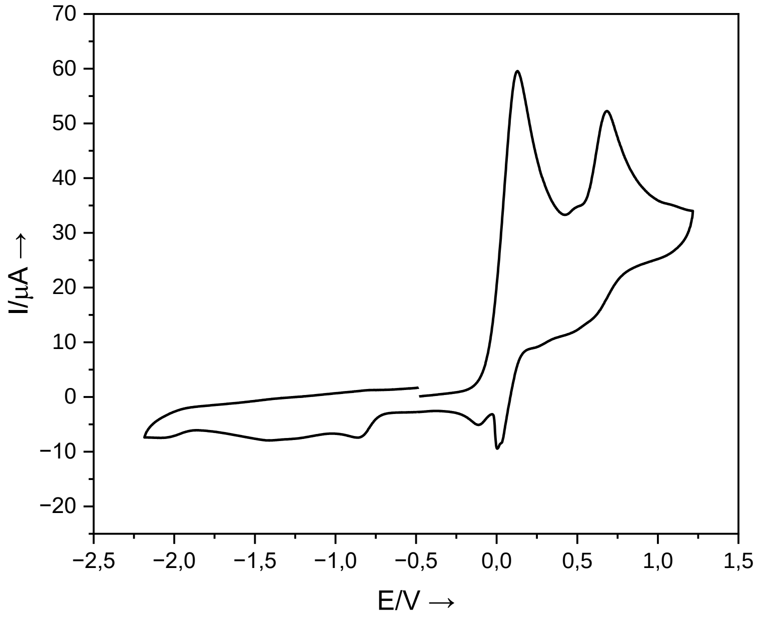

Figure S88: Cyclovoltammogram of L9 in dichloromethane ([*n*-Bu_4N][PF_6], 100 mV/s internally referenced to Fc^+/Fc).

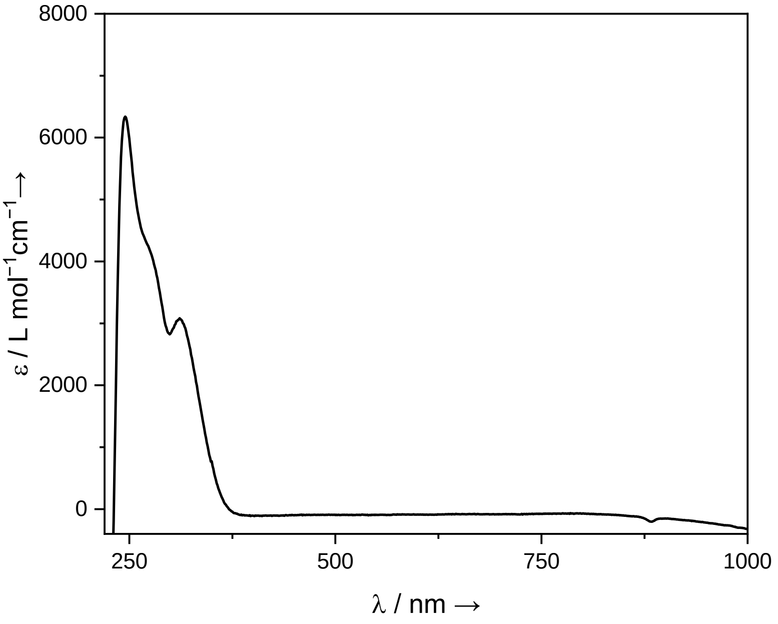

Figure S89: UV-Vis absorption spectrum of L9 in DCM.

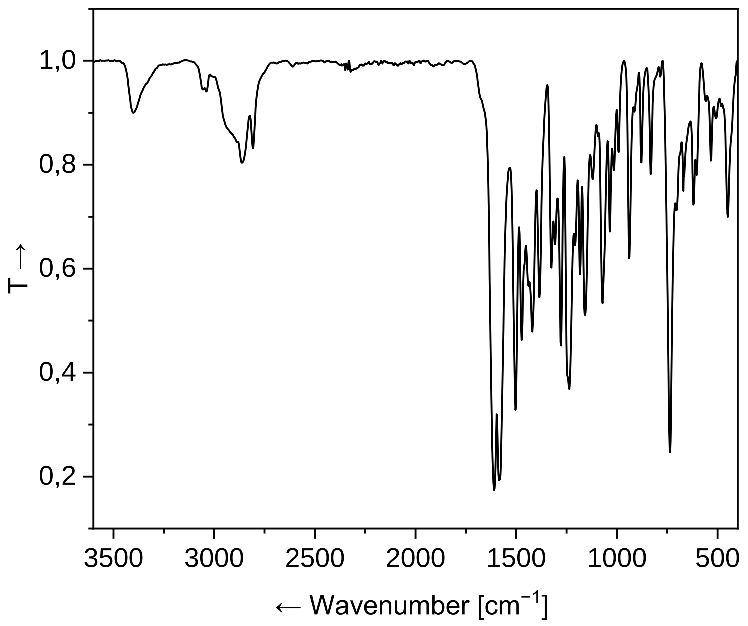

Figure S90: IR spectrum of L9.

9 Protonation and Deprotonation Experiments

9.1 Protonation of L3

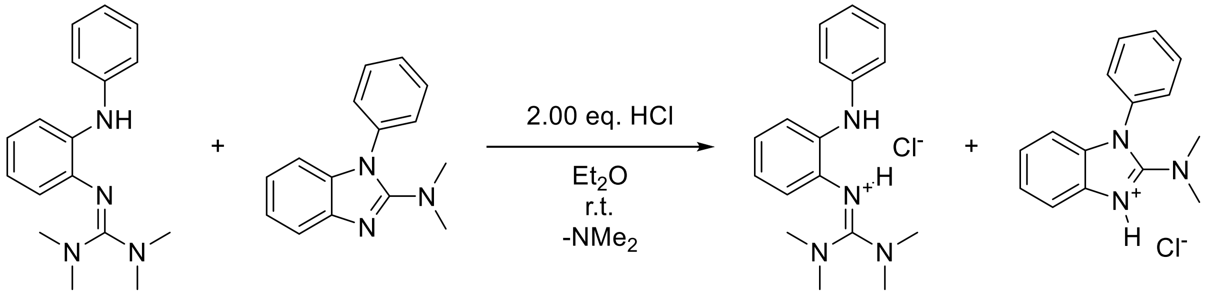

A mixture of 1,1,3,3-tetramethyl-2-(2-(phenylamino)phenyl)guanidine (L3) and *N,N*-dimethyl-1-phenyl-1*H*-benzo[*d*]imidazol-2-amine (13.0 mg, 1.00 eq. 46.0 µmol) was dissolved in 2.00 mL diethyl ether and mixed with 46.0 µL HCl in diethyl ether (3.36 mg, 2.00 eq., 92.1 µmol, 2.00 M). The purple solid was freed from the solvent and washed with 2 × 2.00 mL diethyl ether. Solvent residues were removed *in vacuo* and the product was obtained as a purple solid in quantitative yield (12.60 mg, 46.0 µmol).

1,1,3,3-tetramethyl-2-(2-(phenylamino)phenyl)guanidine-hydrochloride:

^{1}H NMR (400 MHz, MeOD): δ = 7.13 – 6.86 (m, 9 H, CH_{arom}), 2.85 (s, 12 H, 4×CH_3).

MS (ESI+): [M+H$^+$]: calculated: 283.1918, found: 283.1905.

N,N-dimethyl-1-phenyl-1*H*-benzo[*d*]imidazol-2-amine-hydrochloride:

^{1}H NMR (400 MHz, MeOD): δ = 7.82 – 7.59 (m, 5 H, CH_{arom}), 7.54 – 7.33 (m, 2 H, CH_{arom}), 7.34 – 7.18 (m, 2 H, CH_{arom}), 3.19 – 2.93 (m, 6 H, 2×CH_3) ppm.

MS (ESI+): [M+H$^+$]: calculated: 238.1339, found: 238.1327.

Analytical data:

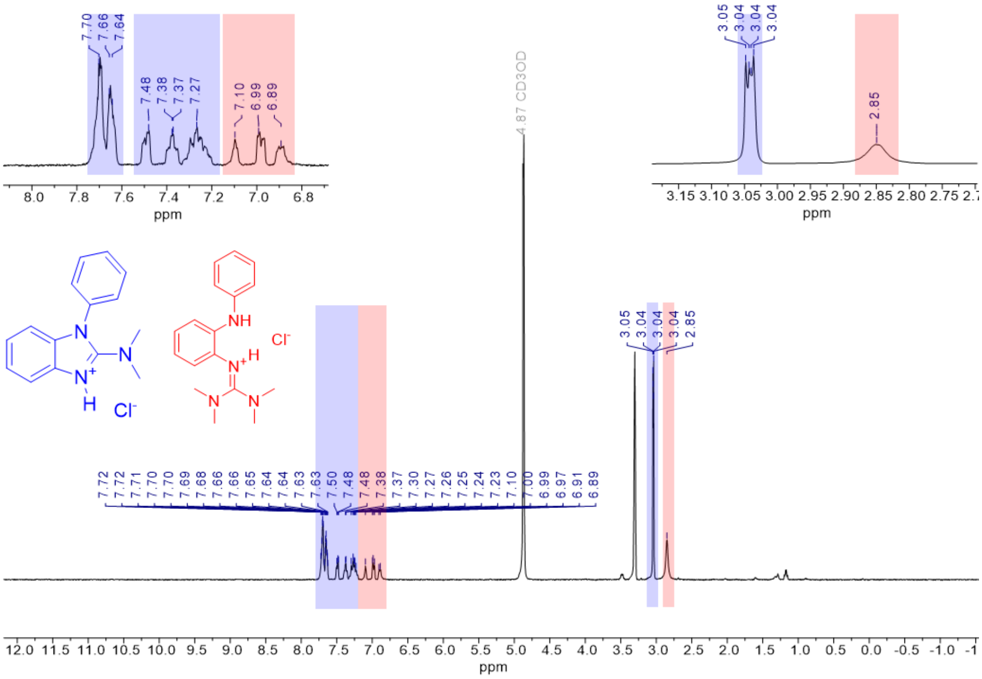

Figure S91: ^{1}H NMR spectrum (400 MHz, MeOD) of the two species 1,1,3,3-tetramethyl-2-(2-(phenylamino)phenyl)guanidine-hydrochloride (red) and *N,N*-dimethyl-1-phenyl-1*H*-benzo[*d*]imidazol-2-amine-hydrochloride (blue). The peak at 3.30 ppm is MeOD.

9.2 Protonation of L4

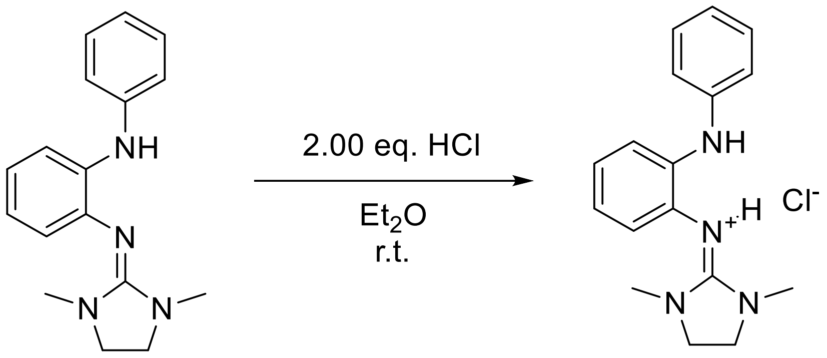

2-((1,3-dimethylimidazolidin-2-ylidene)amino)-N-phenylaniline (L4) (32.0 mg, 1.00 eq. 114 µmol) was dissolved in 2.00 mL diethyl ether and mixed with 114 µL HCl in diethyl ether (8.32 mg, 2.00 eq., 228 µmol, 2.00 M). The purple solid was freed from the solvent and washed with 2 × 2.00 mL diethyl ether. Solvent residues were removed *in vacuo* and the product was obtained as a purple solid in quantitative yield (40.3 mg, 114 µmol).

^{1}H NMR (600 MHz, MeOD): δ = 7.33 – 7.22 (m, 5 H, CH_{arom}), 7.06 – 7.02 (m, 1 H, CH_{arom}), 7.00 – 6.88 (m, 3 H, CH_{arom}), 3.61 (s, 4 H, 2×CH_2), 2.63 (s, 6 H, 2×CH_3).

Analytical data:

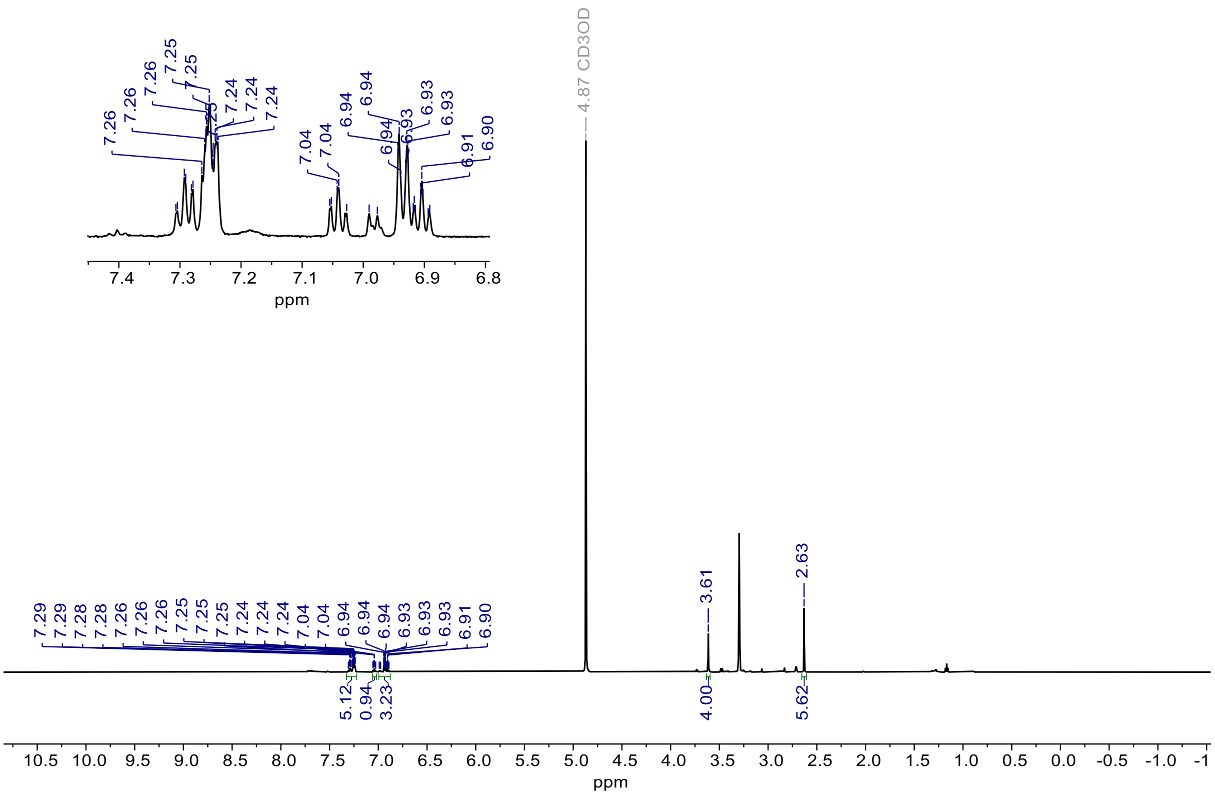

Figure S92: ^{1}H NMR spectrum (600 MHz, MeOD) of the protonation of L4. The peak at 3.30 ppm is MeOD.

9.3 Protonation of L6

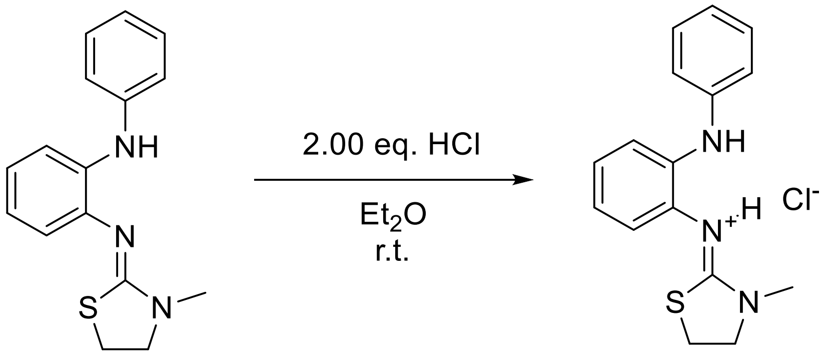

(*Z*)-2-((3-methylthiazolidin-2-ylidene)amino)-*N*-phenylaniline (L6) (12.0 mg, 1.00 eq. 42.3 µmol) was dissolved in 2.00 mL diethyl ether and 42 µL HCl in diethyl ether (3.09 mg, 2.00 eq., 84.7 µmol, 2.00 M) was added. The light rose solid was freed from the solvent and washed with 2 × 2.00 mL diethyl ether. Solvent residues were removed *in vacuo* and the product was obtained as a light rose solid in quantitative yield (13.5 mg, 42.3 µmol).

^{1}H NMR (400 MHz, CD_2Cl_2): δ = 12.90 (s, 1 H, N*H*), 8.46 (s, 1 H, N*H*), 7.45 (d, *J* = 8.2 Hz, 1 H, CH_{arom}), 7.30 – 7.19 (m, 4 H, CH_{arom}), 7.17 (d, *J* = 7.9 Hz, 2 H, CH_{arom}), 6.91 (q, *J* = 8.2 Hz, 2 H, CH_{arom}), 4.08 (t, *J* = 7.8 Hz, 2 H, CH_2), 3.69 (s, 3 H, CH_3), 3.37 (t, *J* = 7.8 Hz, 2 H, CH_2).

Analytical data:

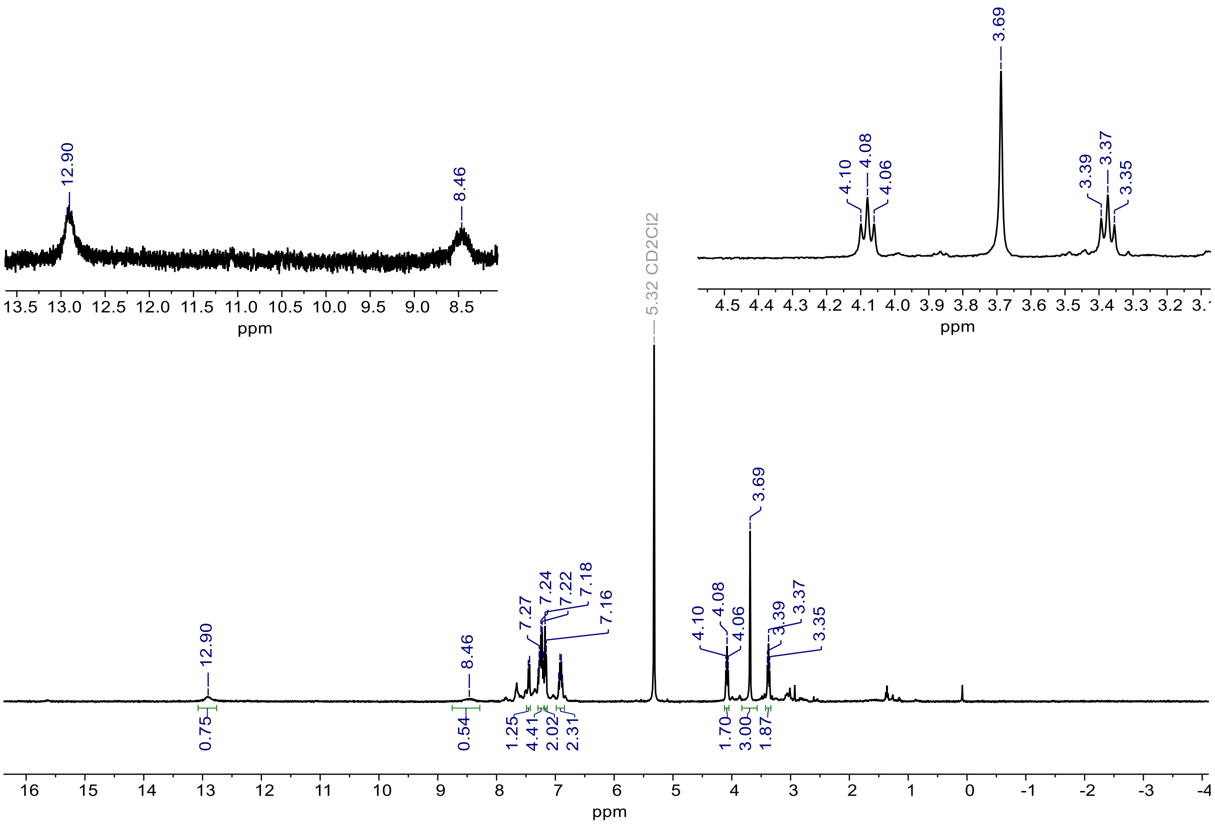

Figure S93: ^{1}H NMR spectrum (400 MHz, CD_2Cl_2) of the protonation of L6. The impurities at 0.08 ppm and 1.26 ppm are due to grease.

9.4 Protonation of L5

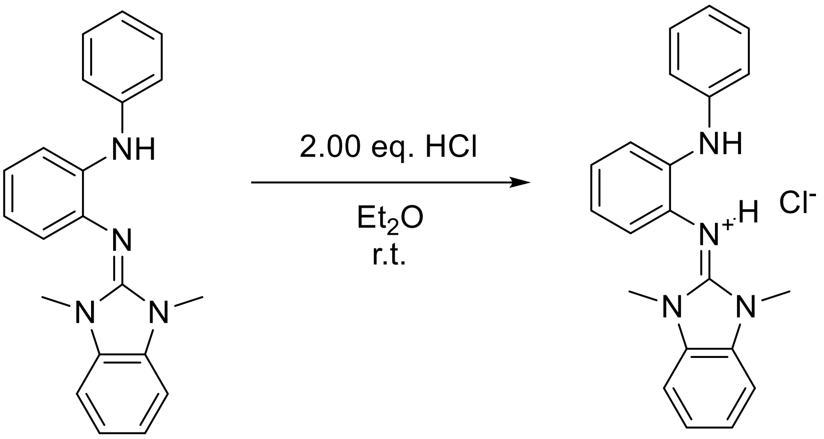

2-((1,3-dimethyl-1,3-dihydro-2*H*-benzo[*d*]imidazol-2-ylidene)amino)-*N*-phenylaniline (L5) (21.0 mg, 1.00 eq. 63.9 µmol) was dissolved in 2.00 mL diethyl ether and 51 µL HCl in diethyl ether (4.66 mg, 2.00 eq., 128 µmol, 2.00 M) was added. The light rose solid was freed from the solvent and washed with 2 × 2.00 mL diethyl ether. Solvent residues were removed *in vacuo* and the product was obtained as a light rose solid in 85% yield (20 mg, 54.3 µmol).

^{1}H NMR (400 MHz, MeOD): δ = 7.57 – 7.46 (m, 4 H, CH_{arom}), 7.45 – 7.36 (m, 2 H, CH_{arom}), 7.31 (dd, *J* = 8.0, 1.5 Hz, 1 H, CH_{arom}), 7.26 – 7.14 (m, 3 H, CH_{arom}), 6.89 (t, *J* = 7.4 Hz, 1 H, CH_{arom}), 6.67 – 6.60 (m, 2 H, CH_{arom}), 3.36 (s, 6 H, 2×CH_3) ppm.

Analytical data:

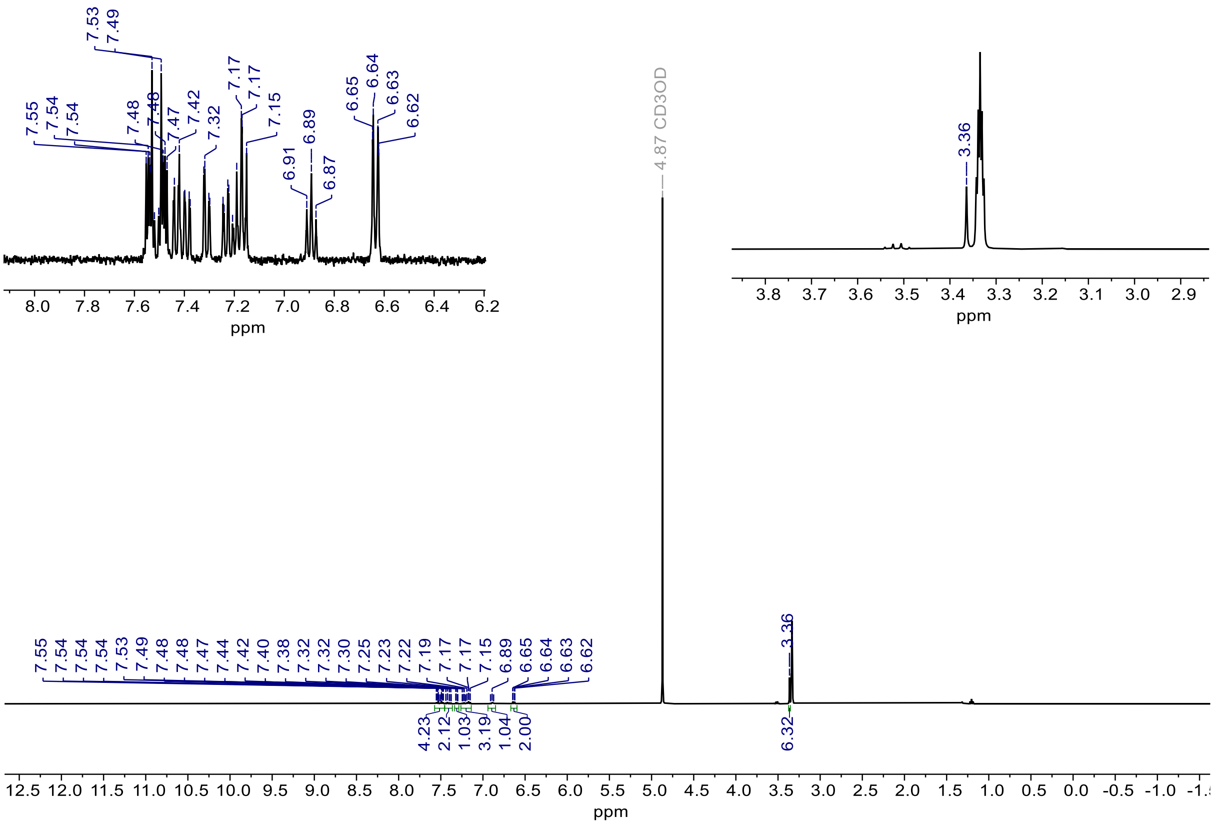

Figure S94: ^{1}H NMR spectrum (400 MHz, MeOD) of the protonation of L5. The peak at 3.30 ppm is MeOD.

9.5 Protonation of L7

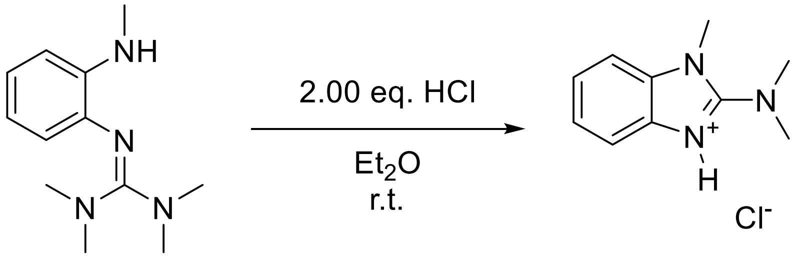

1,1,3,3-tetramethyl-2-(2-(methylamino)phenyl)guanidine (L7) (14.0 mg, 1.00 eq. 63.5 µmol) was dissolved in 2.00 mL diethyl ether and 50 µL HCl in diethyl ether (4.63 mg, 2.00 eq., 127 µmol, 2.00 M) was added. The light rose solid was freed from the solvent and washed with 2 × 2.00 mL diethyl ether. Solvent residues were removed *in vacuo* and the product was obtained as a light rose solid in 68% yield (11 mg, 43.2 µmol).

^{1}H NMR (600 MHz, MeOD): δ = 7.29 (s, 1 H, CH_{arom}), 7.13 – 6.82 (m, 3 H, CH_{arom}), 2.97 (s, 3 H, NCH_3), 2.95 (s, 6 H, 2×CH_3) ppm.

Analytical data:

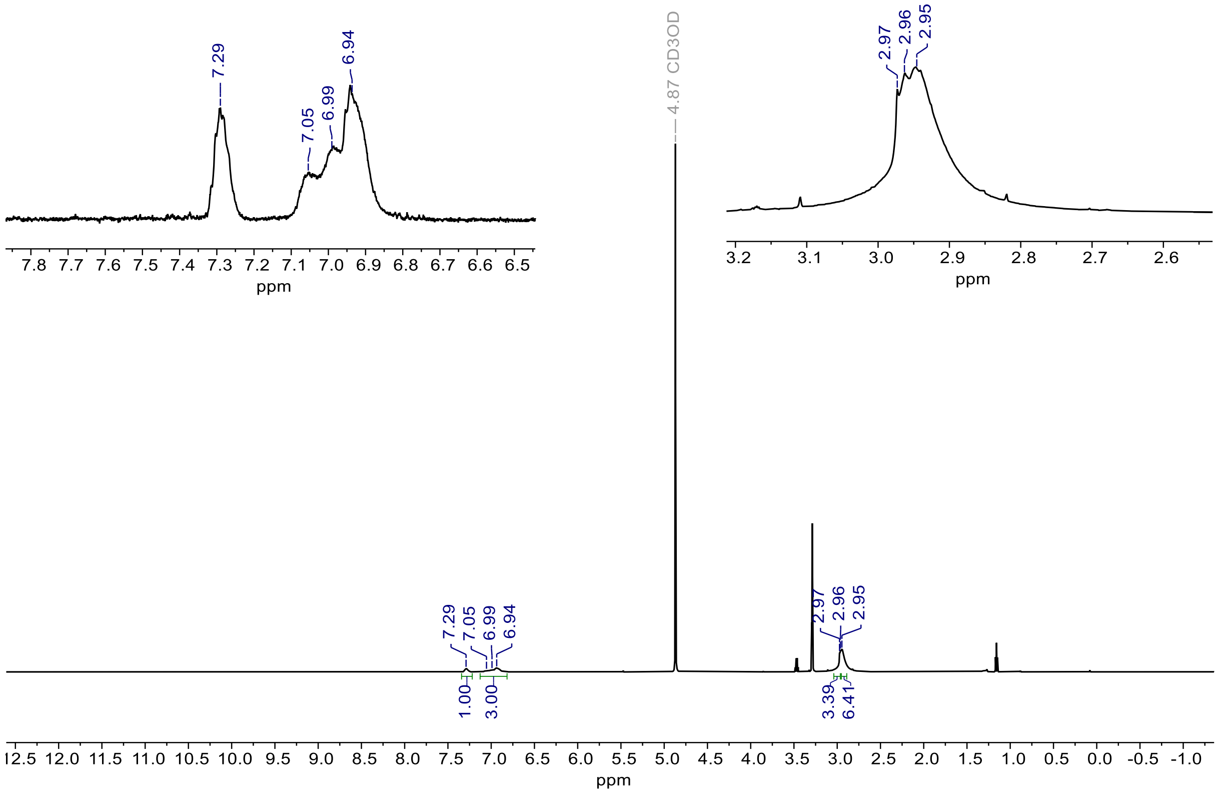

Figure S95: ^{1}H NMR spectrum (600 MHz, MeOD) of the protonation of L7. The peak at 3.30 ppm is MeOD. The spectrum contains diethylether as an impurity.

9.6 Protonation of L8

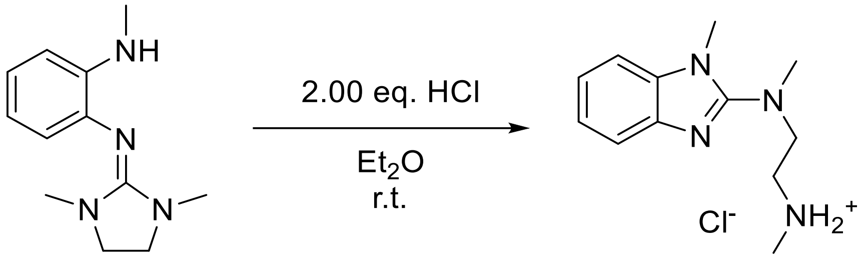

2-((1,3-dimethylimidazolidin-2-ylidene)amino)-*N*-methylaniline (L8) (7.80 mg, 1.00 eq. 35.7 µmol) was dissolved in 2.00 mL diethyl ether and 36 µL HCl in diethyl ether (2.61 mg, 2.00 eq., 71.5 µmol, 2.00 M) was added. The grey solid was freed from the solvent and washed with 2 × 2.00 mL diethyl ether. Solvent residues were removed *in vacuo* and the product was obtained as a grey solid in quantitative yield (9.10 mg, 35.7 µmol).

^{1}H NMR (400 MHz, MeOD): δ = 7.50 (dd, *J* = 5.8, 3.1 Hz, 2 H, CH_{arom}), 7.39 – 7.29 (m, 2 H, CH_{arom}), 3.84 (s, 3 H, CH_3), 3.81 (d, *J* = 6.1 Hz, 2 H, CH_2), 3.41 (t, *J* = 6.1 Hz, 2 H, CH_2), 3.26 (s, 2 H, NH_2), 2.78 (s, 3 H, CH_3).

Analytical data:

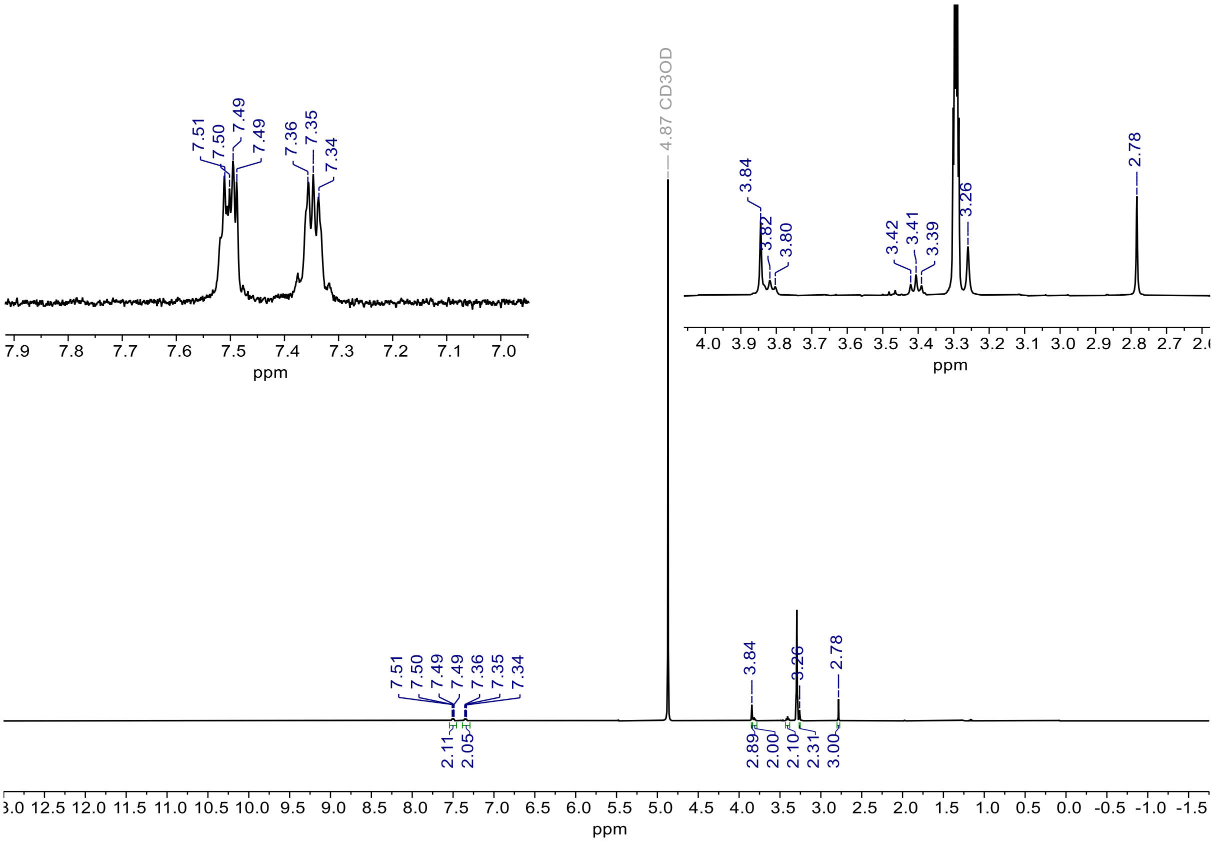

Figure S96: ^{1}H NMR spectrum (400 MHz, MeOD) of the protonation of L8. The peak at 3.30 ppm is MeOD.

9.7 Deprotonation of L7

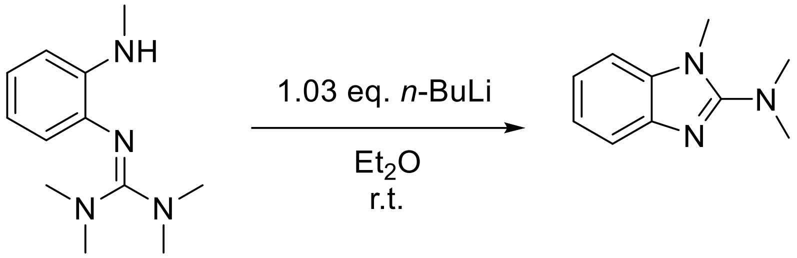

1,1,3,3-tetramethyl-2-(2-(methylamino)phenyl)guanidine (L7) (16.0 mg, 1.00 eq., 72.6 µmol) was dissolved in 2.00 mL THF in an ice bath and 30 µL *n*-BuLi (3.74 mg, 1.03 eq., 2.50 M in *n*-hexane) were slowly added dropwise. The reaction mixture was stirred overnight and the solvent was removed. The orange solid was washed with 2 × 2.00 mL *n*-hexane.

^{1}H NMR (400 MHz, C_6D_6): δ = 7.88 (d, *J* = 7.8 Hz, 1 H, CH_{arom}), 7.25 – 7.18 (m, 1 H, CH_{arom}), 6.87 (d, *J* = 7.7 Hz, 1 H, CH_{arom}), 2.79 (s, 3 H, CH_3), 2.59 (s, 6 H, 2×CH_3) ppm. One aromatic Hydrogen atom is missing due to overlapping with the NMR solvent.

Analytical data:

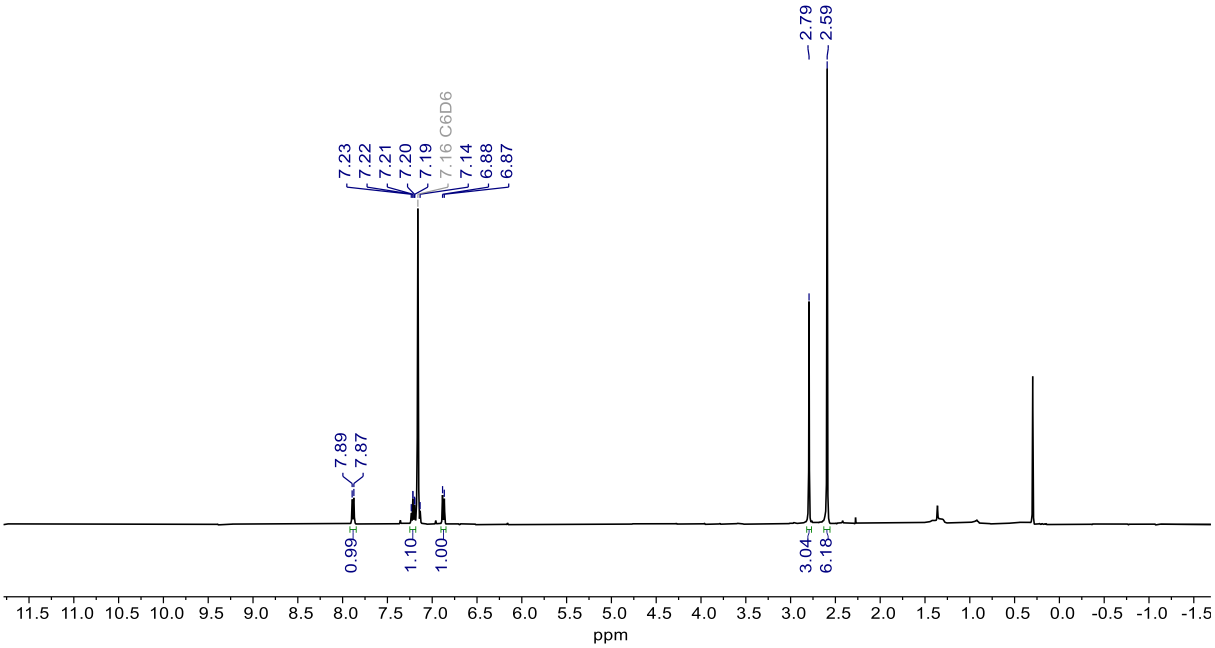

Figure S97: ^{1}H NMR spectrum (400 MHz, C_6D_6) of the deprotonation of L7 The impurities at 1.36 ppm and 0.30 ppm are due to grease.

9.8 Deprotonation of L4

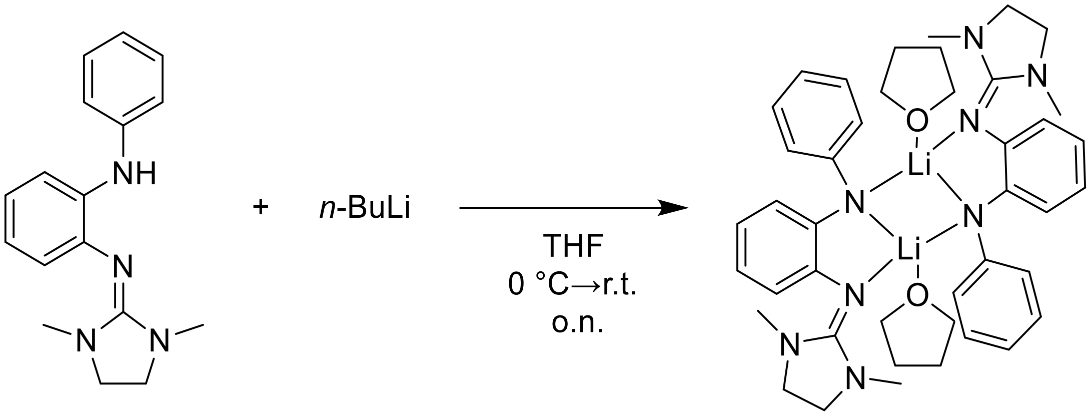

2-((1,3-dimethylimidazolidin-2-ylidene)amino)-*N*-phenylaniline (L4) (11.0 mg, 1.00 eq., 39.2 µmol) was dissolved in 2.00 mL THF in an ice bath and 18.8 µL *n*-BuLi (3.02 mg, 1.20 eq., 2.50 M in *n*-hexane) were slowly added dropwise. The reaction mixture was stirred overnight and the solvent was removed. The reddish solid was washed with 2 × 2.00 mL *n*-hexane, dissolved in THF and overlaid with *n*-hexane. Red crystals were obtained at -31 °C.

Analytical data:

Table S10: Molecular view of $(L4Li(thf))_2$, as well as crystallographic data. H atoms have been omitted for clarity. Displacement ellipsoids correspond to a 50% probability of occurrence. Colour coding: C dark-grey, N blue, H light-grey, Li violet, O red.

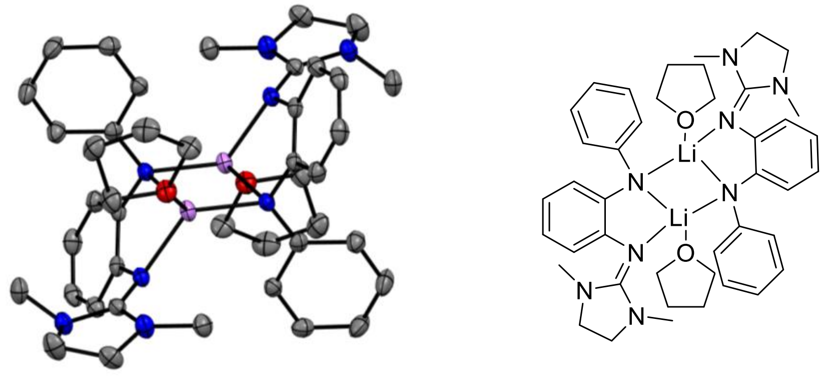

Empirical formula	$C_{42}H_{54}Li_2N_8O_2$
Formula weight	716.81
Temperature/K	100.00
Crystal system	monoclinic
Space group	$P2_1/n$
a/Å	10.9582(7)
b/Å	13.1275(9)
c/Å	13.6099(10)
α/°	90
β/°	100.105(3)
γ/°	90
Volume/Å^3	1927.5(2)
Z	2
ρ_{calc}g/cm^3	1.235
μ/mm^{-1}	0.077
F(000)	768.0
Crystal size/mm^3	0.31 × 0.2 × 0.19
Radiation	MoKα (λ = 0.71073)
2Θ range for data collection/°	4.344 to 53.982
Index ranges	$-13 \le h \le 13$, $-16 \le k \le 16$, $-17 \le l \le 17$

Reflections collected	20557
Independent reflections	4192 [R_{int} = 0.0723, R_{sigma} = 0.0801]
Data/restraints/parameters	4192/0/246
Goodness-of-fit on F^2	1.032
Final R indexes [I>=2σ (I)]	R_1 = 0.0684, wR_2 = 0.1559
Final R indexes [all data]	R_1 = 0.1036, wR_2 = 0.1857
Largest diff. peak/hole / e Å^{-3}	0.33/-0.26

10 Oxidations

10.1 Oxidation of L3

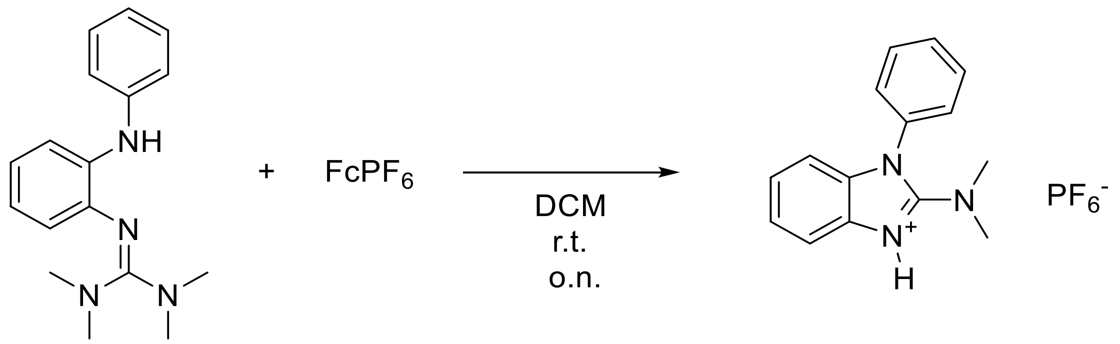

20.0 mg 1,1,3,3-tetramethyl-2-(2-(phenylamino)phenyl)guanidine (L3) (1.00 eq., 70.8 µmol) was dissolved with 23.4 mg ferrocene hexafluorophosphate (1.00 eq., 70.8 µmol) in 2.00 mL dichloromethane and stirred overnight. The solvent of the dark purple solution was removed and the residue was washed with 3 × 2.00 mL. Solvent residues were removed under *in vacuo*. Purple crystals were obtained by layering a concentrated solution of dichloromethane with *n*-hexane. No further analysis was recorded.

Analytical data:

Table S11: Molecular view of [L3'+H]PF_6, as well as crystallographic data. Most H atoms have been emitted for clarity. Displacement ellipsoids correspond to a 50% probability of occurrence. Colour coding: C dark-grey, N blue, H light-grey, F pale yellow, P orange.

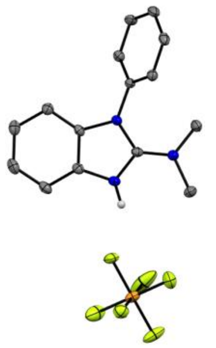

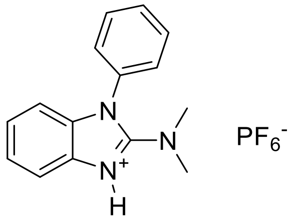

Empirical formula	$C_{15}H_{16}F_6N_3P$
Formula weight	383.28
Temperature/K	100.00
Crystal system	triclinic
Space group	P-1
a/Å	7.7773(5)
b/Å	10.3766(8)
c/Å	10.6089(9)
α/°	76.353(3)
β/°	89.050(3)
γ/°	74.863(3)
Volume/Å^3	802.20(11)
Z	2
ρ_{calc}g/cm^3	1.587
μ/mm^{-1}	0.239
F(000)	392.0

Crystal size/mm^3	0.288 × 0.182 × 0.128
Radiation	MoKα (λ = 0.71073)
2Θ range for data collection/°	3.956 to 53.994
Index ranges	$-9 \le h \le 9$, $-13 \le k \le 13$, $-13 \le l \le 13$
Reflections collected	38470
Independent reflections	3505 [R_{int} = 0.0587, R_{sigma} = 0.0292]
Data/restraints/parameters	3505/0/228
Goodness-of-fit on F^2	1.047
Final R indexes [I>=2σ (I)]	R_1 = 0.0373, wR_2 = 0.0924
Final R indexes [all data]	R_1 = 0.0410, wR_2 = 0.0958
Largest diff. peak/hole / e $Å^{-3}$	0.40/-0.43

10.2 Oxidation of L4

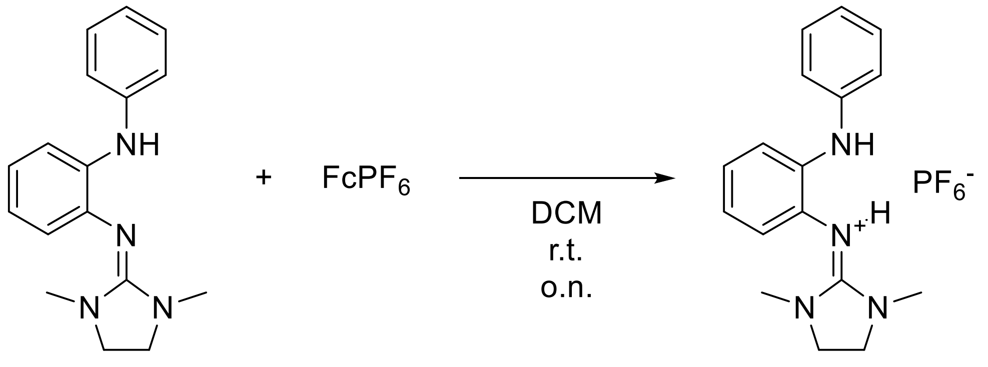

19.0 mg 2-((1,3-dimethylimidazolidin-2-ylidene)amino)-*N*-phenylalanine (1.00 eq., 67.8 µmol) was dissolved with 22.4 mg ferrocene hexafluorophosphate (1.00 eq., 67.8 µmol) in 2.00 mL dichloromethane and stirred overnight. The solvent of the dark purple solution was removed and the residue was washed with 3 × 2.00 mL. Solvent residues were removed under low vacuum. Purple crystals were obtained by layering a concentrated solution of dichloromethane with *n*-hexane. Despite appropriate ^{1}H NMR, no suitable elemental analysis could be obtained. No further analysis was recorded.

^{1}H NMR (400 MHz, CD_2Cl_2): δ = 7.40 – 7.21 (m, 5 H, CH_{arom}), 7.18 – 7.06 (m, 3 H, CH_{arom}), 6.99 (ddd, *J* = 8.6, 7.4, 1.3 Hz, 2 H, CH_{arom}), 6.74 (s, 1 H, N*H*), 6.24 (s, 1 H, N*H*), 3.73 (s, 4 H, 2×CH_2), 2.79 (s, 6 H, 3×CH_3) ppm.

Analytical data:

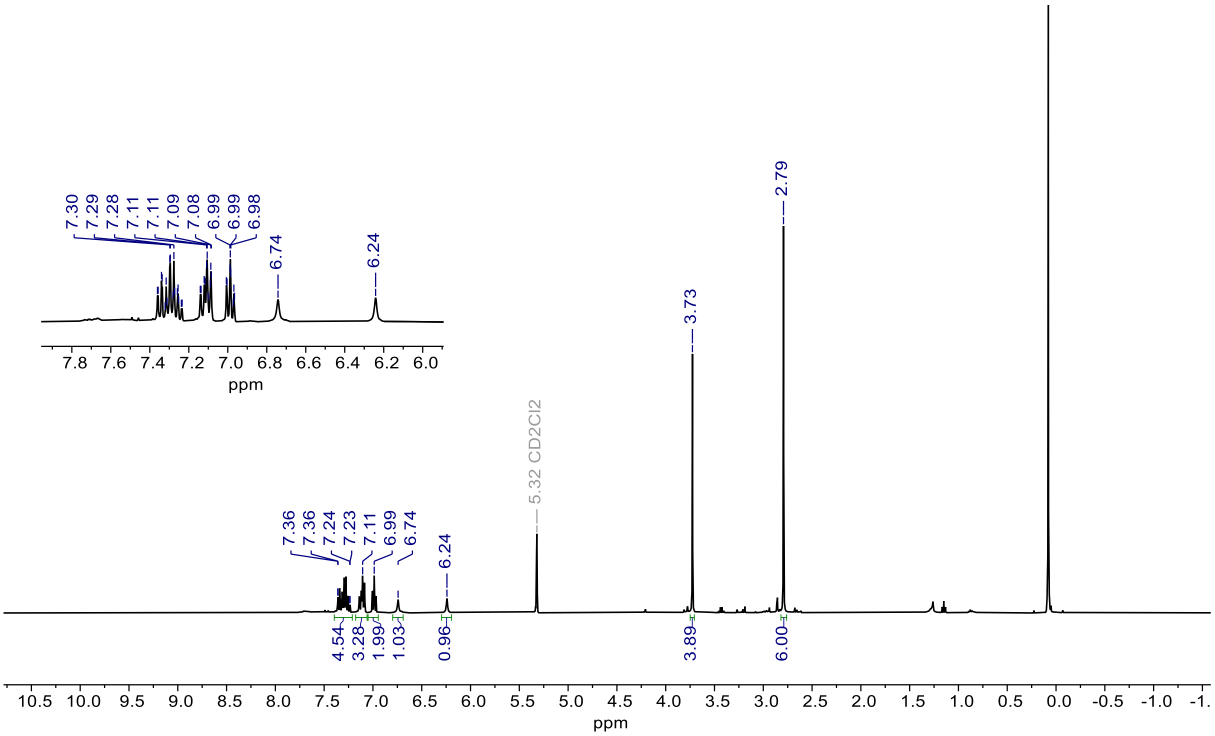

Figure S98: ^1H NMR spectrum (400 MHz, CD_2Cl_2) of $L4HPF_6$. The impurities at 0.08 ppm are due to grease. At 1.15 ppm and 3.38 ppm, these are solvent residues from Et_2O.

Table S12: Molecular view of $[L4+H]PF_6$, as well as crystallographic data. Most H atoms have been emitted for clarity. Displacement ellipsoids correspond to a 50% probability of occurrence. Colour coding: C dark-grey, N blue, H light-grey, F pale yellow, P orange.

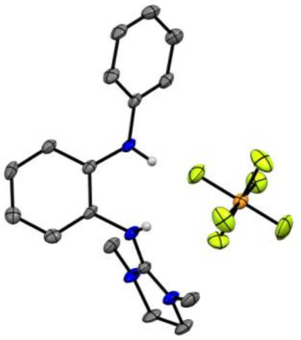

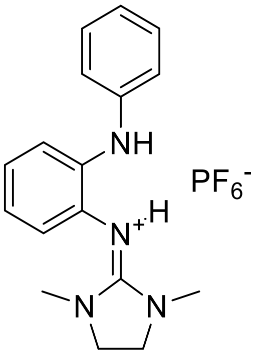

Empirical formula	$C_{17}H_{21}F_6N_4P$
Formula weight	426.35
Temperature/K	100.00
Crystal system	triclinic

Space group	P-1
a/Å	10.4466(12)
b/Å	11.3160(12)
c/Å	16.707(2)
α/°	89.736(5)
β/°	77.202(5)
γ/°	89.943(4)
Volume/Å^3	1925.9(4)
Z	4
ρ_{calc}g/cm^3	1.470
μ/mm^{-1}	0.209
F(000)	880.0
Crystal size/mm^3	0.64 × 0.54 × 0.26
Radiation	MoKα (λ = 0.71073)
2Θ range for data collection/°	2.5 to 52.996
Index ranges	-13 ≤ h ≤ 13, -14 ≤ k ≤ 14, -20 ≤ l ≤ 20
Reflections collected	78305
Independent reflections	7891 [R_{int} = 0.0791, R_{sigma} = 0.0390]
Data/restraints/parameters	7891/2/518
Goodness-of-fit on F^2	1.099
Final R indexes [I>=2σ (I)]	R_1 = 0.0741, wR_2 = 0.2066
Final R indexes [all data]	R_1 = 0.0777, wR_2 = 0.2118
Largest diff. peak/hole / e Å^{-3}	0.92/-1.02

10.3 Oxidation von L7

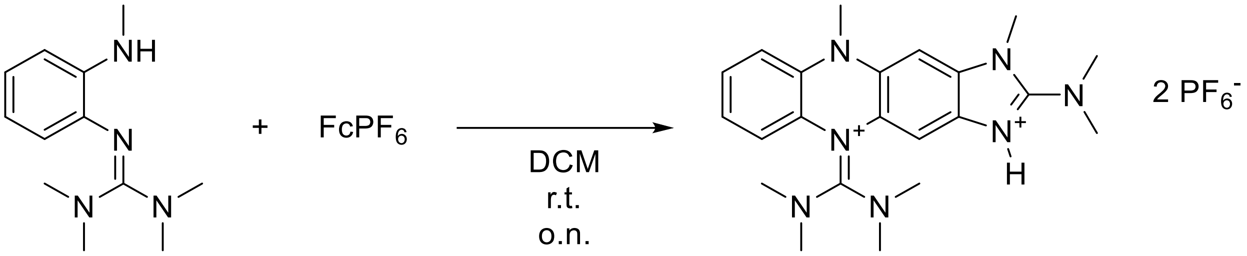

15.0 mg of L7 (68.1 µmol, 1.00 eq.) were dissolved in 2.00 mL of DCM. 22.5 mg of ferrocene hexafluorophosphate (68.1 µmol, 1.00 eq.) was added and the resulting reaction mixture was stirred at room temperature overnight, causing the solution to turn dark. The solvent was then removed *in vacuo*. The remaining residue was washed with 2 × 2.00 mL of absolute diethyl ether and further solvent residues were removed under *in vacuo*. The product was obtained in 76% yield (28 mg, 59.91 µmol). A crystallization attempt using DCM and *n*-hexane yielded crystals of the shown oxidation product. Further purification attempts were carried out, but the product could not be isolated.

^{1}H NMR (600 MHz, $CDCl_3$): δ = 7.21 – 7.19 (m, 1 H, $CH_{arom.}$), 6.72 (dd, *J* = 8.2, 1.3 Hz, 2 H, CH_{arom}), 6.64 (td, *J* = 7.5, 1.3 Hz, 2 H, $CH_{arom.}$), 6.56 (dd, *J* = 7.8, 1.5 Hz, 1 H, $CH_{arom.}$), 4.68 (s, 1 H, N*H*), 3.23 – 2.68 (m, 24 H, 8×CH_3) ppm.

MS (ESI^+ in DCM): $[M]^+$: m/z = calculated: 221.1039, found: 221.1029.

Elemental analysis (%): Oxidation product of L7 •1 DCM

calculated:	C: 35.95	H: 4.33	N: 12.75
found:	C: 36.80	H: 5.87	N: 13.86

Analytical data:

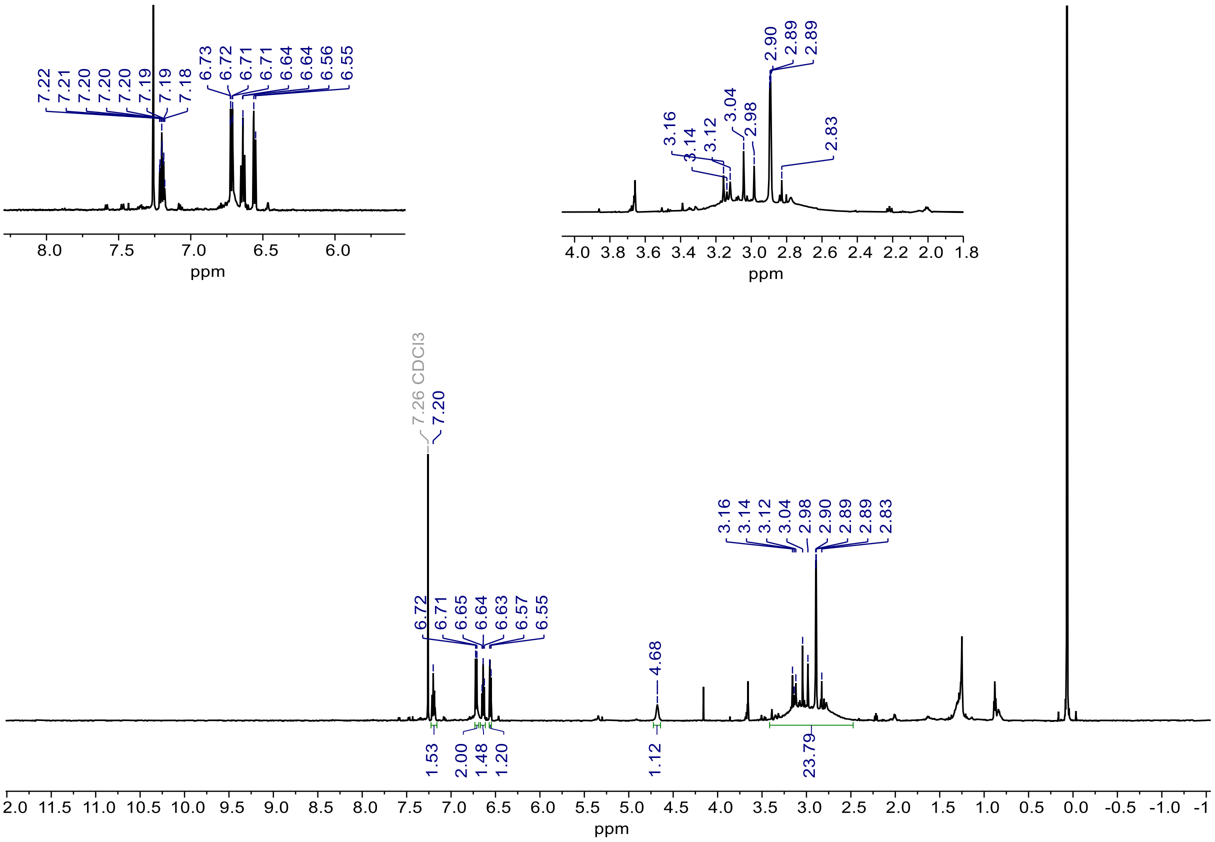

Figure S99: ^{1}H NMR spectrum (600 MHz, $CDCl_3$) of the oxidation product of L7. The impurities at 0.08 ppm are due to grease, while the signal at 4.15 ppm is due to ferrocene. At 1.15 ppm and 3.38 ppm, these are solvent residues from diethyl ether.

Table S13: Molecular view of the oxidation product of L7, as well as crystallographic data. Most H atoms have been omitted for clarity. Solvent molecules are emitted due to clarity. Displacement ellipsoids correspond to a 50% probability of occurrence. Colour coding: C dark-grey, N blue, H light-grey, F pale yellow, P orange.

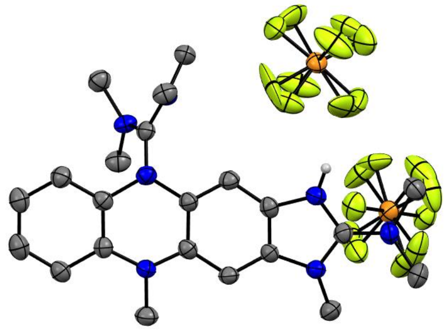

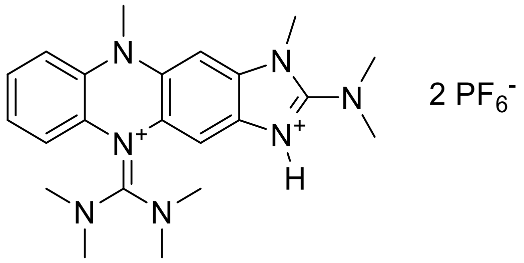

Empirical formula	$C_{23}H_{33}Cl_2F_{12}N_7P_2$
Formula weight	768.40
Temperature/K	100.00
Crystal system	monoclinic

Space group	$P2_1/n$
a/Å	11.0849(17)
b/Å	21.455(3)
c/Å	13.428(2)
α/°	90
β/°	102.218(6)
γ/°	90
Volume/Å^3	3121.1(8)
Z	4
ρ_{calc}g/cm^3	1.635
μ/mm^{-1}	0.412
F(000)	1568.0
Crystal size/mm^3	0.42 × 0.09 × 0.05
Radiation	MoKα (λ = 0.71073)
2Θ range for data collection/°	4.212 to 52.994
Index ranges	-13 ≤ h ≤ 13, 0 ≤ k ≤ 26, 0 ≤ l ≤ 16
Reflections collected	6455
Independent reflections	6455 [R_{int} = ?, R_{sigma} = 0.0893]
Data/restraints/parameters	6455/633/515
Goodness-of-fit on F^2	1.019
Final R indexes [I>=2σ (I)]	R_1 = 0.0729, wR_2 = 0.1740
Final R indexes [all data]	R_1 = 0.1184, wR_2 = 0.2040
Largest diff. peak/hole / e Å^{-3}	0.77/-0.55

10.4 Oxidation of L8

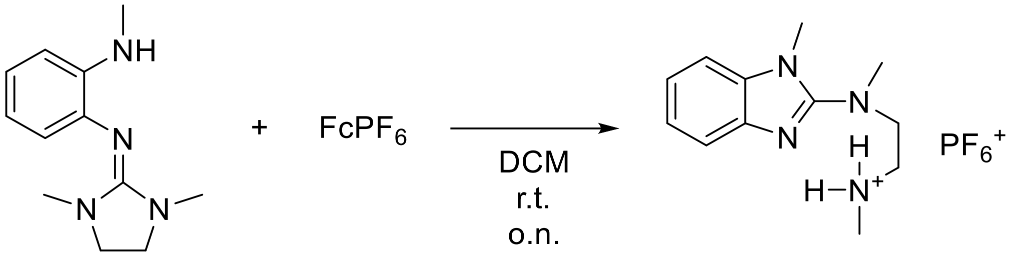

11.0 mg 2-((1,3-dimethylimidazolidin-2-ylidene)amino)-*N*-methylaniline (1.00 eq., 50.4 µmol) was dissolved with 16.7 mg ferrocene hexafluorophosphate (1.00 eq., 50.4 µmol) in 2.00 mL dichloromethane and stirred overnight. The solvent from the dark solution was removed and the residue was washed with 3 × 2.00 mL. Solvent residues were removed *in vacuo*. Colourless crystals were obtained by layering a concentrated solution of dichloromethane with *n*-hexane. Further attempts at purification were unsuccessful.

Analytical data:

Table S14: Molecular view of the oxidation product of L8, as well as crystallographic data. Solvent molecules and most H atoms are emitted due to clarity. Displacement ellipsoids correspond to a 50% probability of occurrence. Colour coding: C dark-grey, N blue, H light-grey, F pale yellow, P orange.

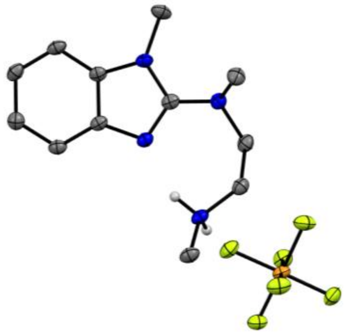

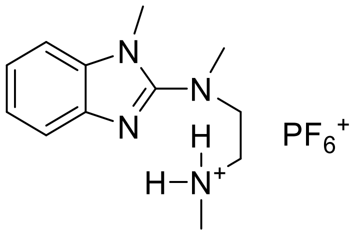

Empirical formula	$C_{12}H_{19}F_6N_4P$
Formula weight	364.28
Temperature/K	100.00
Crystal system	triclinic
Space group	P-1
a/Å	8.4334(6)
b/Å	9.5356(8)
c/Å	10.7743(9)
α/°	91.187(3)
β/°	111.438(3)
γ/°	102.281(3)
Volume/Å^3	783.41(11)
Z	2
ρ_{calc}g/cm^3	1.544
μ/mm^{-1}	0.242
F(000)	376.0

Crystal size/mm^3	0.22 × 0.17 × 0.13
Radiation	MoKα (λ = 0.71073)
2Θ range for data collection/°	4.086 to 53.998
Index ranges	$-10 \le h \le 10$, $-12 \le k \le 12$, $-13 \le l \le 13$
Reflections collected	39425
Independent reflections	3432 [R_{int} = 0.0930, R_{sigma} = 0.0381]
Data/restraints/parameters	3432/2/219
Goodness-of-fit on F^2	1.037
Final R indexes [I>=2σ (I)]	R_1 = 0.0388, wR_2 = 0.0914
Final R indexes [all data]	R_1 = 0.0477, wR_2 = 0.0982
Largest diff. peak/hole / e $Å^{-3}$	0.23/-0.39

11 Complexes of secondary and primary amines

11.1 [$CoCl_2$(L4)]

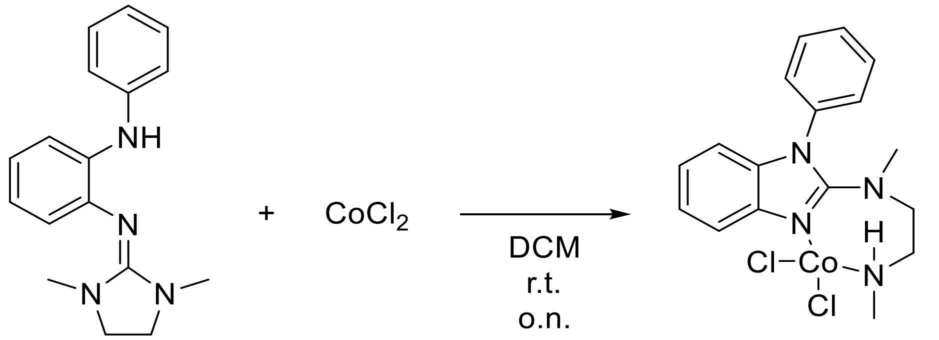

2-((1,3-dimethylimidazolidin-2-ylidene)amino)-*N*-phenylaniline (L4) (20.5 mg, 1.00 eq., 73.1 µmol) and cobalt chloride (9.49 mg, 1.00 eq., 73.1 µmol) were dissolved in 2.00 mL of dichloromethane and stirred overnight at room temperature. The solvent was removed under reduced vacuum and the dark blue solid was washed with 2 × 2.00 mL of diethyl ether. The blue solid was isolated in quantitative yield (29.9 mg, 73.1 µmol). Crystals were obtained by dissolving the blue solid in dichloromethane and overlaying with *n*-hexane.

UV-Vis (DCM, c = 1.0726·10^{-4} mol·l^{-1}, d = 1 cm): λ_{max} (ε [l·mol^{-1}·cm^{-1}]) = 250 (4794), 284 (4725), 291 (4749), 564 (246), 613 (315), 639 (316) nm.

IR (ATR): $\tilde{\nu}$ = 3165.84, 3056.33, 3001.80, 2919.52, 2871.54, 2814.54, 2648.28, 1685.56, 1611.07, 1594.74, 1573.86, 1356.90,1305.87, 1259.17, 1208.07, 1187.19, 1110.37, 1053.75, 1034.86, 1003.99, 909.96, 878.62, 838.40, 667.09, 659.54, 616.57, 570.62, 538.21, 513.60, 480.40, 459.22, 449.21, 419.12 cm^{-1}.

Elemental analysis (%): $CoCl_2L4$ • 1.5 H_2O

calculated:	C: 46.70	H: 5.30	N: 12.81
found:	C: 46.92	H: 4.84	N: 13.23

Analytical data:

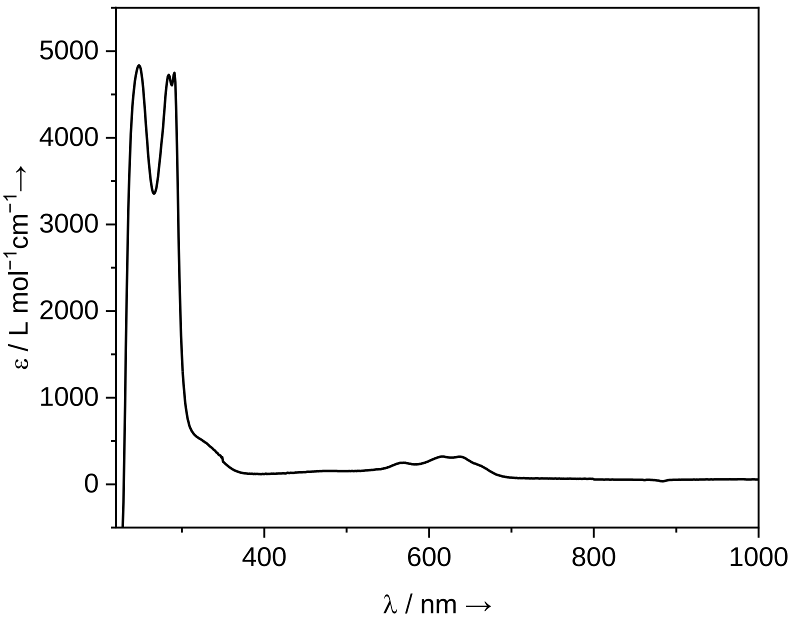

Figure S100: UV-Vis spectrum of [$CoCl_2$(L4)] in DCM.

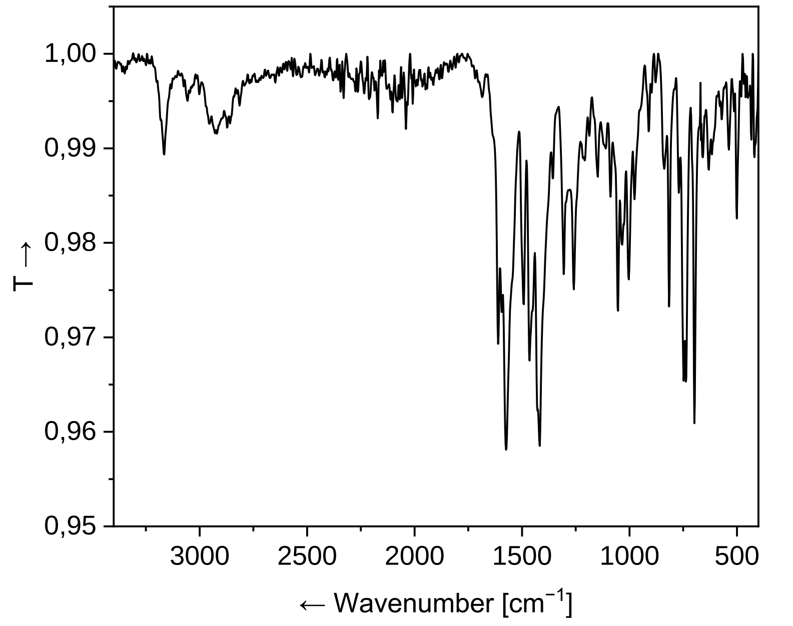

Figure S101: IR spectrum of [$CoCl_2$(L4)].

Table S15: Molecular view of [$CoCl_2$(L4)], as well as crystallographic data. Most H atoms have been omitted for clarity. Displacement ellipsoids drawn at the 50% probability level. Colour coding: C dark-grey, N blue, H light-grey, Co violet, Cl green

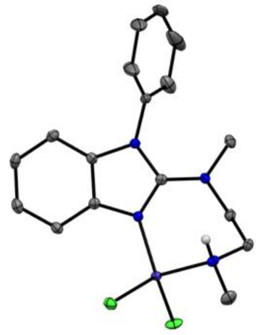

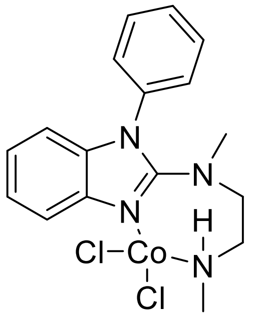

Empirical formula	$C_{17}H_{20}N_4Cl_2Co$
Formula weight	410.20
Temperature/K	100.00
Crystal system	monoclinic
Space group	$P2_1/c$
a/Å	11.2896(6)
b/Å	15.2974(8)
c/Å	10.7906(5)
α/°	90
β/°	97.326(2)
γ/°	90
Volume/Å^3	1848.34(16)
Z	4
ρ_{calc}g/cm^3	1.474
μ/mm^{-1}	1.223
F(000)	844.0
Crystal size/mm^3	0.31 × 0.24 × 0.09
Radiation	MoKα (λ = 0.71073)
2Θ range for data collection/°	4.508 to 54
Index ranges	-14 ≤ h ≤ 14, -19 ≤ k ≤ 19, -13 ≤ l ≤ 13
Reflections collected	115653
Independent reflections	4013 [R_{int} = 0.0755, R_{sigma} = 0.0221]

Data/restraints/parameters	4013/0/223
Goodness-of-fit on F^2	1.086
Final R indexes [I>=2σ (I)]	R_1 = 0.0311, wR_2 = 0.0779
Final R indexes [all data]	R_1 = 0.0330, wR_2 = 0.0799
Largest diff. peak/hole / e $Å^{-3}$	0.57/-0.43

11.2 [CoBr2(L4)]

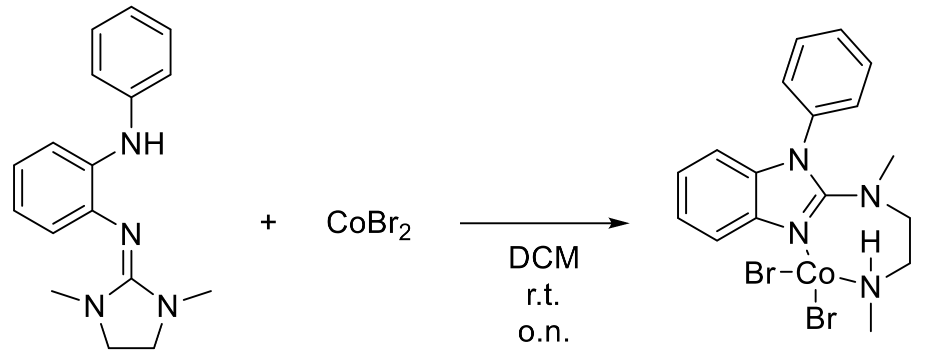

2-((1,3-dimethylimidazolidin-2-ylidene)amino)-*N*-phenylaniline (L4) (38.0 mg, 1.00 eq., 136 µmol) and cobalt bromide (29.7 mg, 1.00 eq., 136 µmol) were dissolved in 2.00 mL of dichloromethane and stirred overnight at room temperature. The solvent was removed under reduced vacuum and the dark green solid was washed with 2 × 2.00 mL of diethyl ether. The green solid was obtained in quantitative yield (67.6 mg, 136 µmol).

^{1}H NMR$_{para}$ (600 MHz, CD_2Cl_2): δ = 212.39 (s), 145.54 (s), 63.62 (s), 26.99 (s), 21.55 (s), 12.53 (s), 11.82 (s), 11.03 (s), 9.96 (s), 8.42 (s), 7.23 (s), 3.67 (s), 2.93 (s), 2.75 (s), 1.27 (d, *J* = 15.9 Hz), -37.26 (s), -112.55 (s).

^{13}C NMR$_{para}$ (151 MHz, CD_2Cl_2): δ = 389.29, 291.88, 219.70, 167.57, 166.50, 156.54, 155.49, 135.63, 134.54, 77.53, 75.05, 73.96, -153.19, -210.51 ppm.

UV-Vis (DCM, $c = 1.12195 \cdot 10^{-4}$ mol·l^{-1}, d = 1 cm): λ_{max} (ε [l·mol^{-1}·cm^{-1}]) = 251 (3072), 284 (3194), 583 (255), 631 (341), 650 (342) nm.

IR (ATR): $\tilde{\nu}$ = 3163.44, 3054.26, 2933.82, 1596.45, 1575.66, 1494.21, 1466.11, 1423.00, 1356.14, 1306.26, 1259.39, 1203.74, 1187.49, 1146.66, 1109.10, 1086.31, 1035.99, 976.77, 910.99, 838.85, 769.41, 669.93, 659.67, 624.59, 565.90, 501.24, 447.30, 406.98 cm^{-1}

Elemental analysis (%):

calculated:	C: 40.91	H: 4.04	N: 11.23
found:	C: 40.63	H: 4.51	N: 11.49

Analytical data:

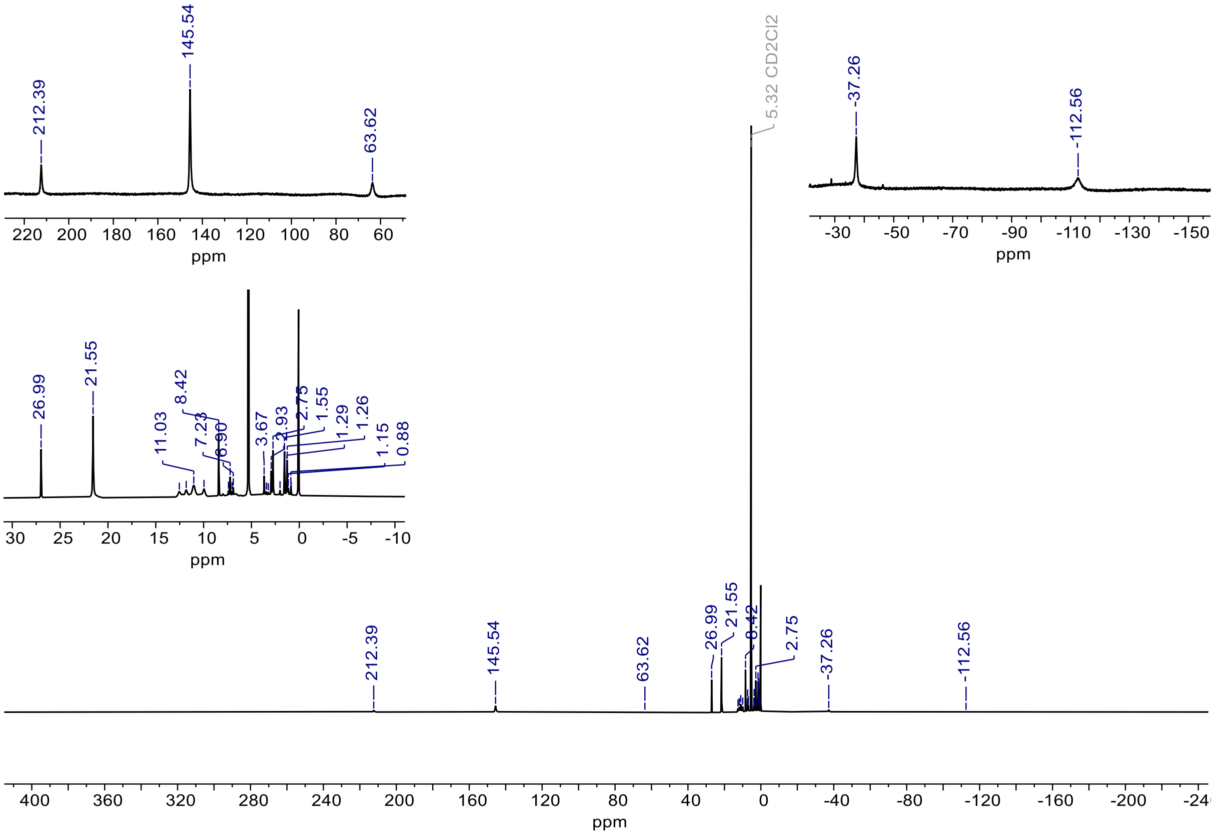

Figure S102: Paramagnetic ^{1}H NMR spectrum (600 MHz, CD_2Cl_2) of $[CoBr_2(L4)]$.

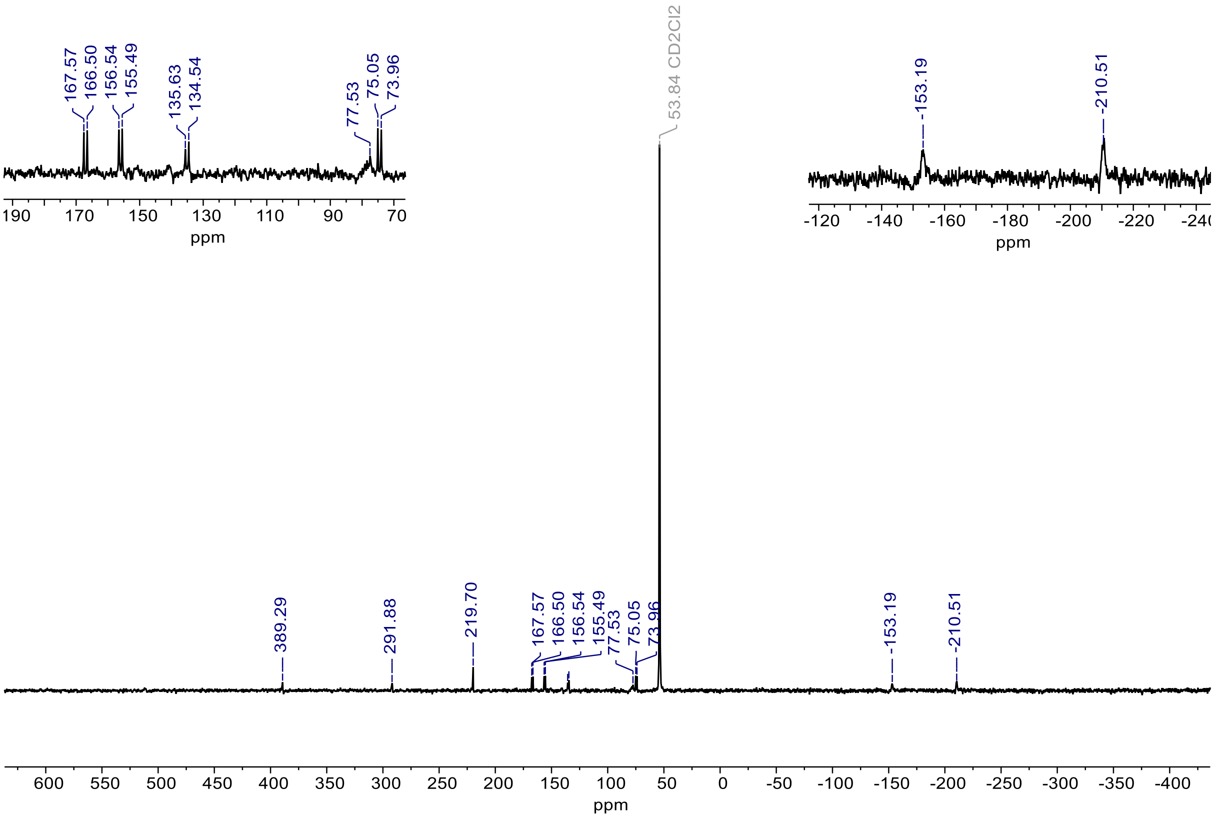

Figure S103: Paramagnetic ^{13}C NMR spectrum (151 MHz, CD_2Cl_2) of [$CoBr_2$(L4)].

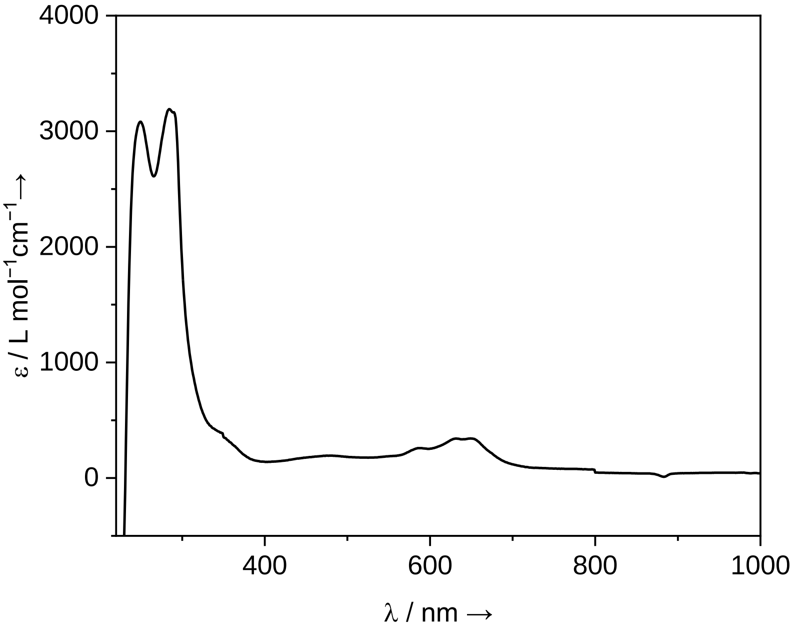

Figure S104: UV-Vis spectrum of [$CoBr_2$(L4)] in DCM.

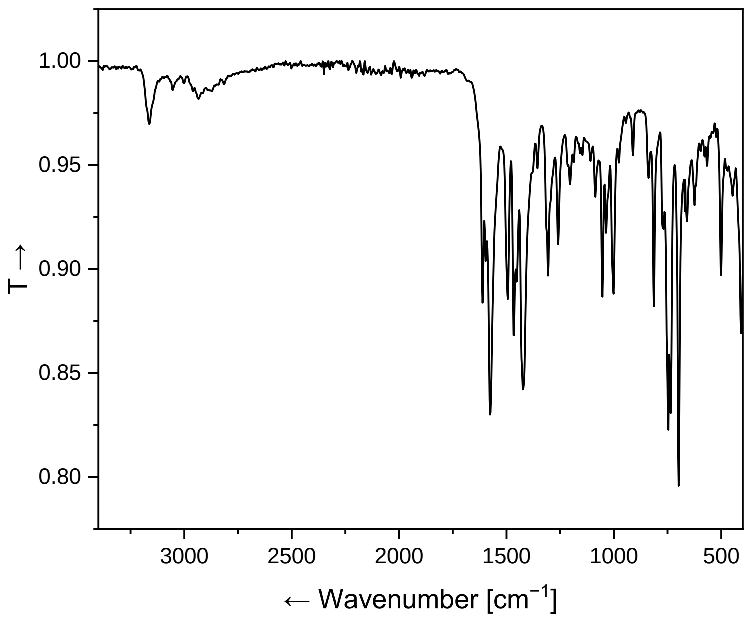

Figure S105: IR spectrum of [$CoBr_2$(L4)].

Table S16: Molecular view of the complex [$CoBr_2$(L4)], as well as crystallographic data. Most H atoms have been omitted for clarity. Displacement ellipsoids correspond to a 50% probability of occurrence. Colour coding: C dark-grey, N blue, H light-grey, Co violet, Br brown.

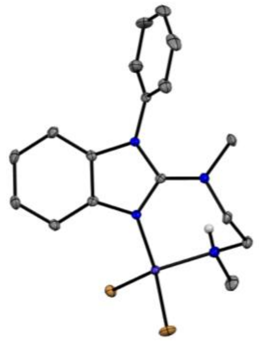

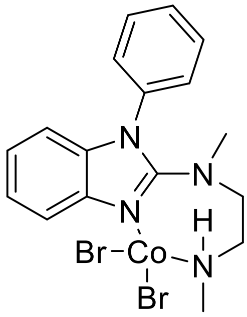

Empirical formula	$C_{17}H_{20}N_4CoBr_2$
Formula weight	499.12
Temperature/K	100.00
Crystal system	monoclinic
Space group	$P2_1/c$
a/Å	11.2276(6)
b/Å	15.4348(9)
c/Å	11.0579(6)
α/°	90

β/°	95.234(2)
γ/°	90
Volume/Å^3	1908.30(18)
Z	4
ρ_{calc}g/cm^3	1.737
μ/mm^{-1}	5.093
F(000)	988.0
Crystal size/mm^3	0.21 × 0.203 × 0.108
Radiation	MoKα (λ = 0.71073)
2Θ range for data collection/°	4.498 to 57.498
Index ranges	-15 ≤ h ≤ 15, -20 ≤ k ≤ 20, -14 ≤ l ≤ 14
Reflections collected	74041
Independent reflections	4967 [R_{int} = 0.0679, R_{sigma} = 0.0319]
Data/restraints/parameters	4967/0/223
Goodness-of-fit on F^2	1.039
Final R indexes [I>=2σ (I)]	R_1 = 0.0269, wR_2 = 0.0688
Final R indexes [all data]	R_1 = 0.0311, wR_2 = 0.0714
Largest diff. peak/hole / e Å^{-3}	0.85/-0.50

11.3 [$ZnCl_2$(L4)]

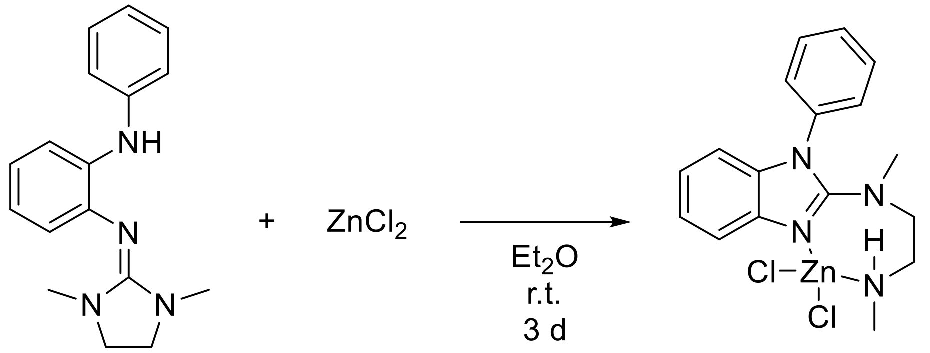

2-((1,3-dimethylimidazolidin-2-ylidene)amino)-*N*-phenylaniline (23.0 mg, 1.00 eq., 82.0 µmol) and zinc chloride (11.2 mg, 1.00 eq., 82.0 µmol) were dissolved in 2.00 mL of diethyl ether and stirred at room temperature for three days. The solvent was removed by syringe filtration and the pink solid was washed with 2 × 2.00 mL of diethyl ether. The solid was dried in a fine vacuum and the product was obtained as a pink solid in quantitative yield (34.0 mg, 83.0 µmol). Crystals were obtained by evaporating a concentrated acetone solution in air.

UV-Vis (DCM, c = 1.12195·10^{-4} mol·l^{-1}, d = 1 cm): λ_{max} (ε [l·mol^{-1}·cm^{-1}]) = 247 (4482), 284 (4730), 291 (4888) nm.

IR (ATR): $\tilde{v}$ = 3195.78, 3155.35, 3142.83, 3058.04, 3046.53, 2963.26, 2941.15, 2349.23, 1614.61, 1597.91, 1580.36, 1541.87, 1498.65, 1486.03, 1469.85, 1420.07, 1383.83, 1357.80, 1306.75, 1296.19, 1262.88, 1218.85, 1206.94, 1154.02, 1109.88, 1091.99, 1058.43, 1038.33, 1004.20, 978.11, 944.14, 914.21, 881.89, 831.71, 815.25, 799.89, 767.13, 752.15, 734.28, 697.25, 670.13, 664.79, 624.07, 616.97, 578.61, 566.03, 520.31, 502.65, 482.85, 464.57, 444.26, 421.84, 406.83 cm^{-1}.

Analytical data:

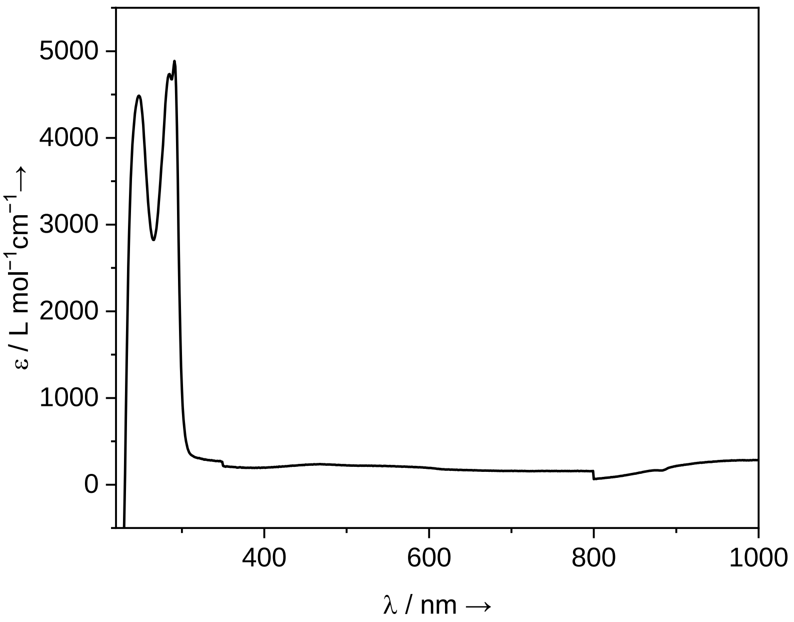

Figure S106: UV-Vis spectrum of [$ZnCl_2$(L4)] in DCM.

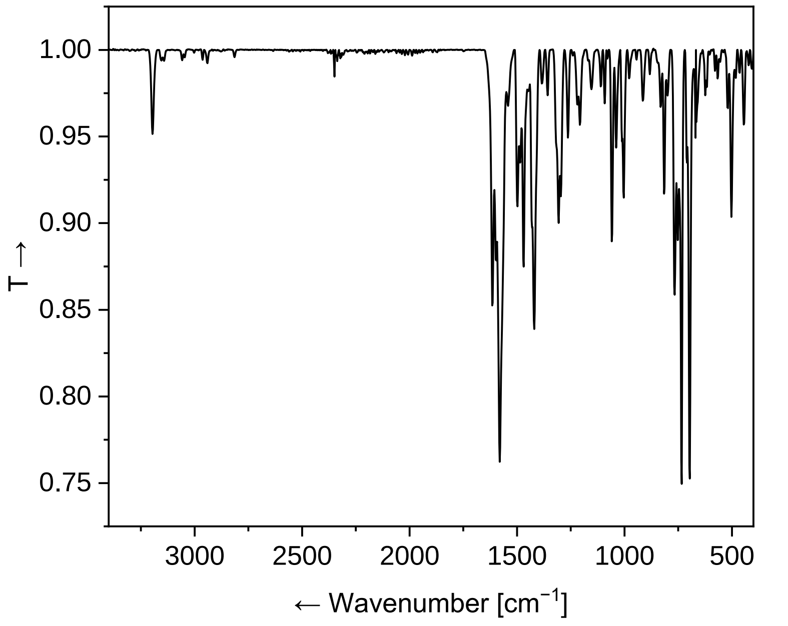

Figure S107: IR spectrum of [$ZnCl_2$(L4)].

Table S17: Molecular view of the complex [$ZnCl_2$(L4)], as well as crystallographic data. Most H atoms have been omitted for clarity. Displacement ellipsoids correspond to a 50% probability of occurrence. Colour coding: C dark-grey, N blue, H light-grey, Zn violet, Cl green.

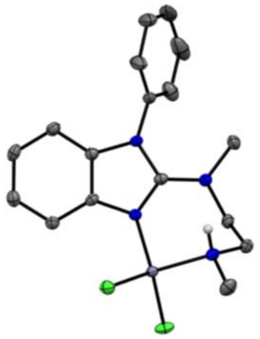

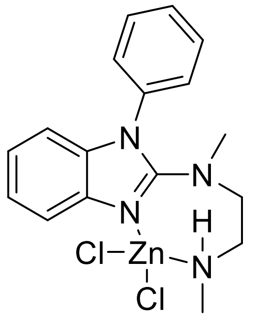

Empirical formula	$C_{17}H_{20}Cl_2N_4Zn$
Formula weight	416.64
Temperature/K	100.00
Crystal system	monoclinic
Space group	$P2_1/c$
a/Å	11.2741(6)
b/Å	15.2999(8)
c/Å	10.7962(6)
α/°	90
β/°	97.291(2)
γ/°	90
Volume/Å^3	1847.21(17)
Z	4
ρ_{calc}g/cm^3	1.498
μ/mm^{-1}	1.625
F(000)	856.0
Crystal size/mm^3	0.14 × 0.12 × 0.04

Radiation	MoKα (λ = 0.71073)
2Θ range for data collection/°	4.512 to 55.112
Index ranges	$-14 \le h \le 14$, $-19 \le k \le 19$, $-14 \le l \le 14$
Reflections collected	135005
Independent reflections	4263 [R_{int} = 0.0920, R_{sigma} = 0.0278]
Data/restraints/parameters	4263/0/223
Goodness-of-fit on F^2	1.063
Final R indexes [I>=2σ (I)]	R_1 = 0.0294, wR_2 = 0.0691
Final R indexes [all data]	R_1 = 0.0379, wR_2 = 0.0741
Largest diff. peak/hole / e Å^{-3}	0.44/-0.39

11.4 [$ZnCl_2$(L7)]

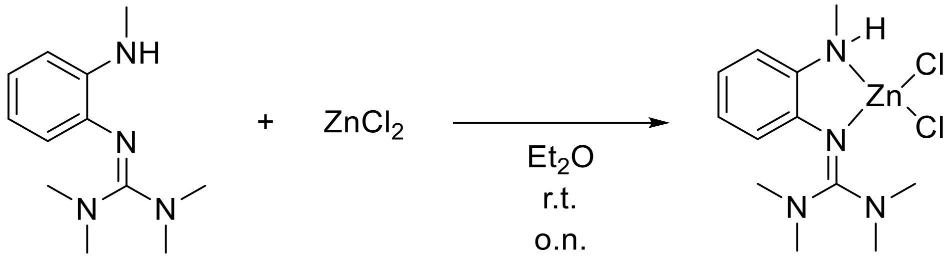

1,1,3,3-tetramethyl-2-(2-(methylamino)phenyl)guanidine (L7) (25.0 mg, 1.00 eq., 113 µmol) and zinc chloride (15.6 mg, 1.00 eq., 113 µmol) were dissolved in 2.00 mL of diethyl ether and stirred overnight at room temperature. The grey solid was washed with 3 × 2.00 mL of diethyl ether and then dried under a fine vacuum. The product was obtained in quantitative yield (40.5 mg, 113 µmol) as a pink powder. Crystals were obtained by evaporating a concentrated acetone solution in air.

^{1}H NMR (400 MHz, $CDCl_3$): δ [ppm] = 7.28 (d, *J* = 7.7 Hz, 1 H, CH_{arom}), 7.18 (td, *J* = 7.7, 1.5 Hz, 1 H, CH_{arom}), 6.98 (td, *J* = 7.7, 1.4 Hz, 1 H, CH_{arom}), 6.56 (dd, *J* = 8.0, 1.4 Hz, 1 H, CH_{arom}), 3.93 (d, *J* = 5.8 Hz, 1 H, N*H*), 3.05 – 2.63 (m, 15 H, 1 × NCH_3 4 × CH_3).

CV:

Irreversible oxidation at: E_{Ox1}: 0.91 V.

Irreversible reduction at: E_{Red1}: 0.47 V, E_{Red2}: 0.18 V, E_{Red2}: -0.08 V.

UV-Vis (DCM, c = $2.24 \cdot 10^{-5}$ mol·l^{-1}, d = 1 cm): λ_{max} (ε [l·mol^{-1}·cm^{-1}]) =269 nm (9395) nm.

IR (ATR): $\tilde{\nu}$ = 3243, 3007, 2937, 2880, 2345, 1627, 1545, 1489, 1445, 1422, 1394, 1344, 1219, 1159, 1100, 1062, 1033, 971, 860, 816, 775, 757, 745, 710, 667, 633, 530, 508, 417 cm^{-1}.

Elemental analysis (%): [$ZnCl_2$(L7)] • 1 H_2O

calculated:	C: 38.47	H: 5.92	N: 14.96
found:	C: 38.61	H: 5.95	N: 14.35

Analytical data:

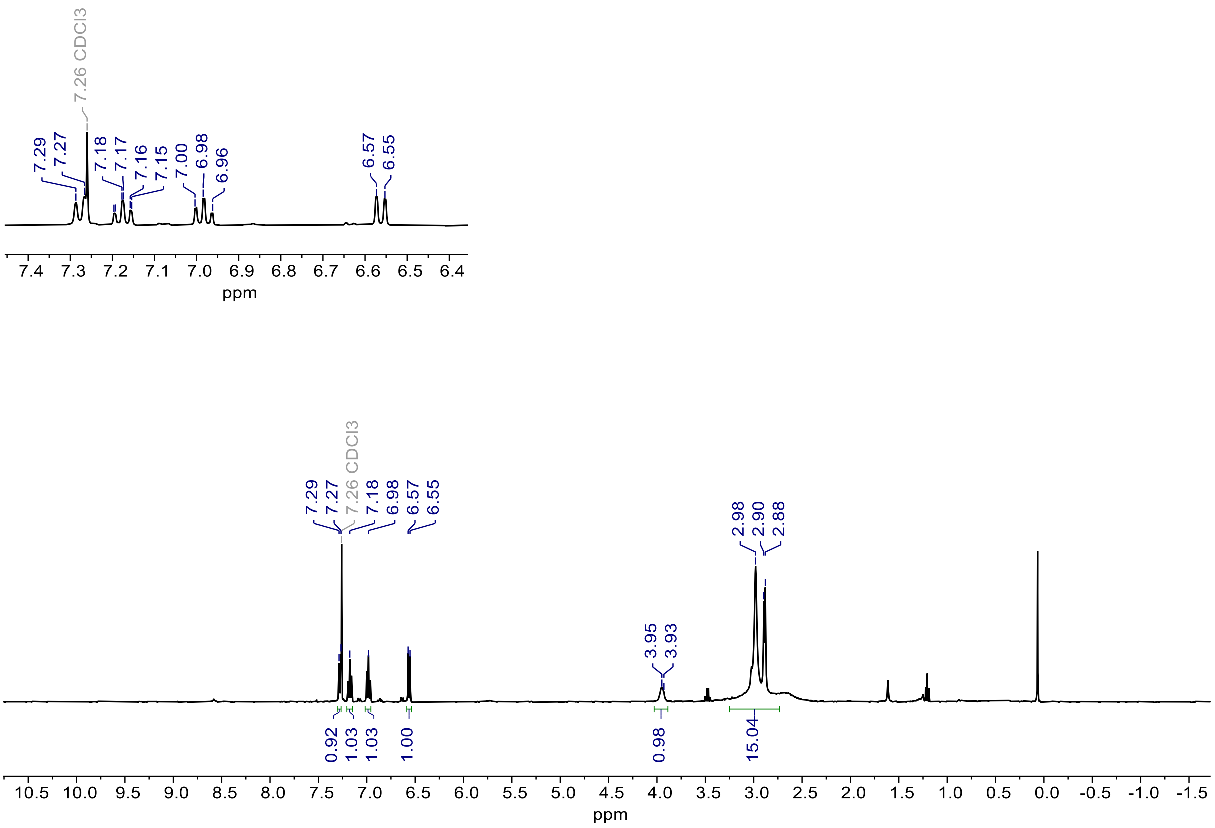

Figure S108: ^{1}H NMR spectrum (400 MHz, $CDCl_3$) of [$ZnCl_2$(L7)]. The impurities at 1.55 ppm are due to water, at 3.49 ppm and at 1.22 ppm due to diethylether. Additionally the impurities at 1.22 ppm, 0.88 ppm and 0.07 ppm are due to grease.

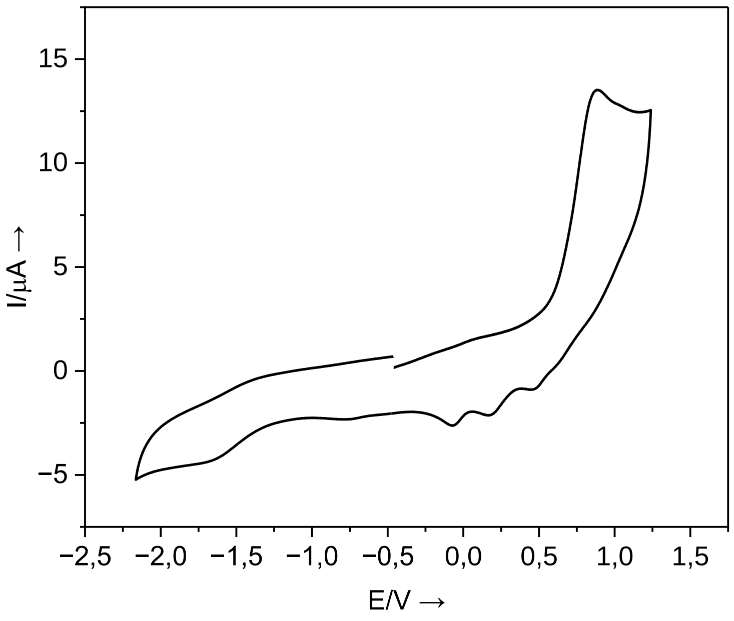

Figure S109: Cyclovoltammogram of [$ZnCl_2$(L7)] in dichloromethane ([*n*-Bu_4N][PF_6], 100 mV/s internally referenced to Fc^+/Fc).

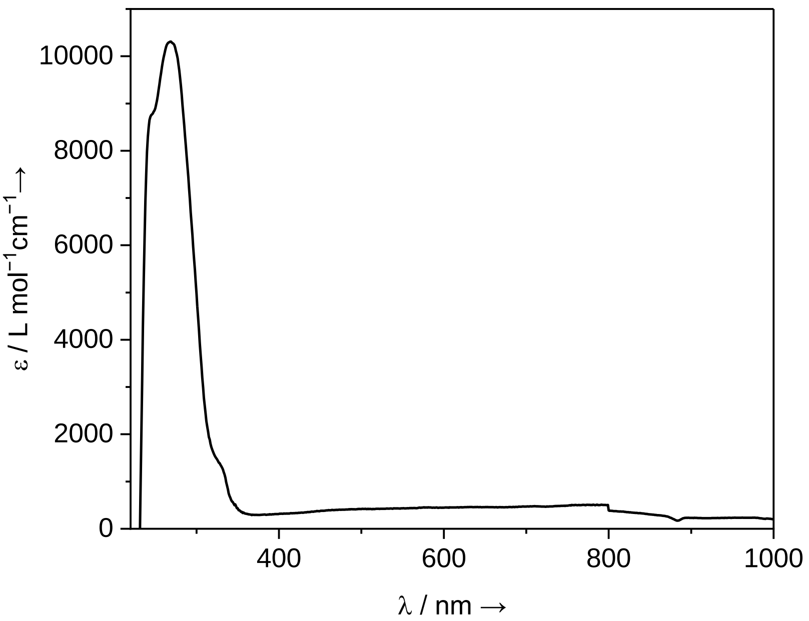

Figure S110: UV-Vis spectrum of [$ZnCl_2$(L7)] in DCM.

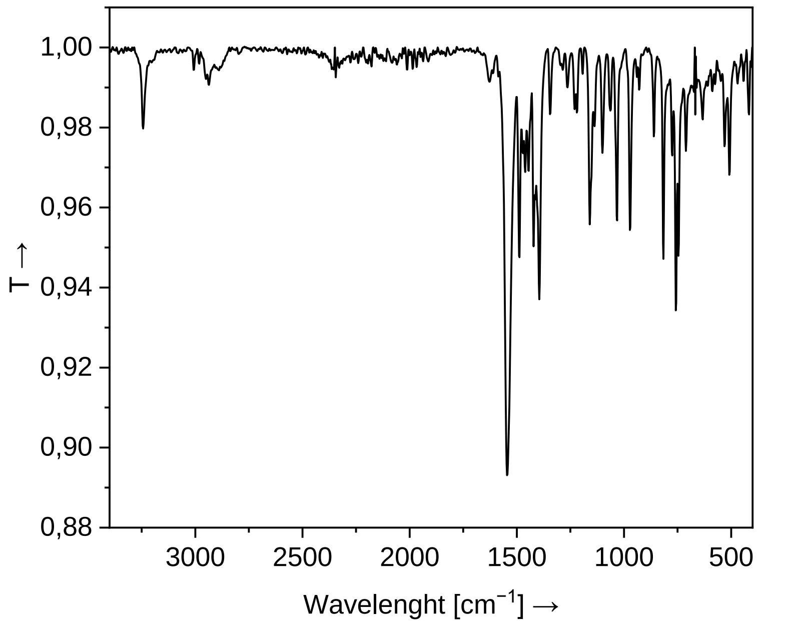

Figure S111: IR spectrum of [$ZnCl_2$(L7)].

Table S18: Molecular view of the complex [$ZnCl_2$(L7)], as well as crystallographic data. Most H atoms have been omitted for clarity. Displacement ellipsoids correspond to a 50% probability of occurrence. Colour coding: C dark-grey, N blue, H light-grey, Zn violet, Cl green.

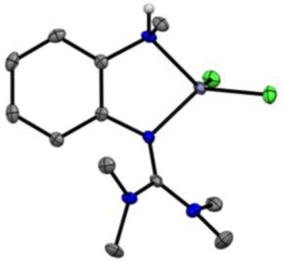

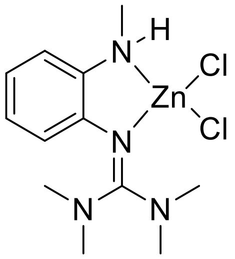

Empirical formula	$C_{12}H_{20}Cl_2N_4Zn$
Formula weight	356.59
Temperature/K	100.00
Crystal system	orthorhombic
Space group	Pbcn
a/Å	14.5474(6)
b/Å	11.0221(5)
c/Å	19.7469(9)
α/°	90
β/°	90
γ/°	90
Volume/Å^3	3166.3(2)
Z	8
ρ_{calc}g/cm^3	1.496
μ/mm^{-1}	1.881
F(000)	1472.0
Crystal size/mm^3	0.18 × 0.14 × 0.11
Radiation	MoKα (λ = 0.71073)
2Θ range for data collection/°	4.126 to 54.374
Index ranges	-18 ≤ h ≤ 18, -14 ≤ k ≤ 14, -25 ≤ l ≤ 25
Reflections collected	145061
Independent reflections	3518 [R_{int} = 0.0923, R_{sigma} = 0.0257]

Data/restraints/parameters	3518/0/181
Goodness-of-fit on F^2	1.066
Final R indexes [I>=2σ (I)]	R_1 = 0.0269, wR_2 = 0.0666
Final R indexes [all data]	R_1 = 0.0318, wR_2 = 0.0702
Largest diff. peak/hole / e $Å^{-3}$	0.39/-0.49

11.5 [$CoBr_2$(L8)]

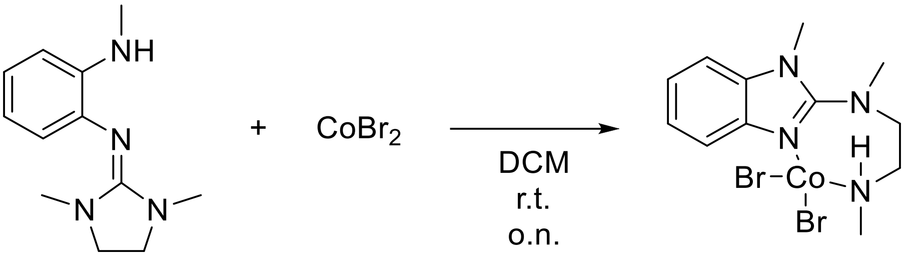

2-((1,3-dimethylimidazolidin-2-ylidene)amino)-*N*-methylaniline (L8) (33.1 mg, 1.00 eq., 152 µmol) and cobalt bromide (33.1 mg, 1.00 eq., 152 µmol) were dissolved in 2.00 mL of dichloromethane and stirred overnight at room temperature. The solvent was removed under reduced vacuum and the dark blue solid was washed with 2 × 2.00 mL of diethyl ether. The solid was dissolved in 2.00 mL of dichloromethane and overlaid with 6.00 mL of *n*-hexane, yielding dark blue crystals in 58% yield (37.4 mg, 88.12 µmol).

^{1}H NMR$_{para}$ (600 MHz, CD_2Cl_2): δ = 220.65 (s), 146.49 (s), 47.35 (s), 32.08 (s), 20.58 (s), 17.39 (s), 7.52 (s), 3.32 (s), 2.01 (s), 1.28 – 1.24 (m), 1.09 (s), -32.99 (s), -114.57 (s) ppm.

UV-Vis (DCM, c = $1.8075 \cdot 10^{-4}$ mol·l^{-1}, d = 1 cm): λ_{max} (ε [l·mol^{-1}·cm^{-1}]) =251 (1668), 284 (1785), 584 (156), 527 (broad, 229), 653 (broad, 223) nm.

IR (ATR): $\tilde{\nu}$ = 3159.38, 2965.19, 2863.37, 1610.89, 1577.85, 1480.42, 1455.44, 1416.50, 1401.48, 1319.27, 1302.07, 1237.73, 1210.36, 1155.87, 1121.75, 1103.12, 1084.35, 1050.76, 986.33, 912.96, 810.74, 743.34, 664.88, 654.69, 621.65, 554.13, 420.29 cm^{-1}.

Elemental analysis (%):

calculated:	C: 32.98	H: 4.15	N: 12.85
found:	C: 32.46	H: 4.50	N: 12.79

Analytical data:

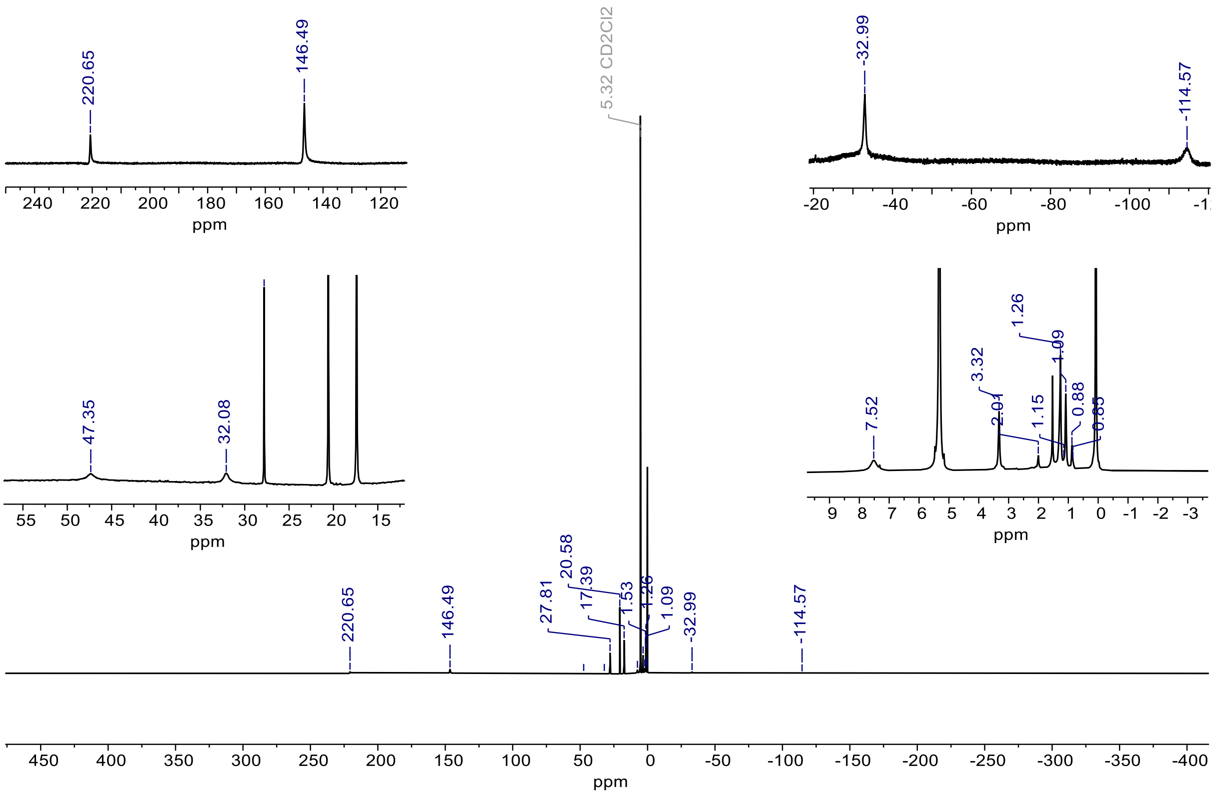

Figure S112: Paramagnetic ^{1}H NMR spectrum (600 MHz, CD_2Cl_2) of [$CoBr_2$(L8)].

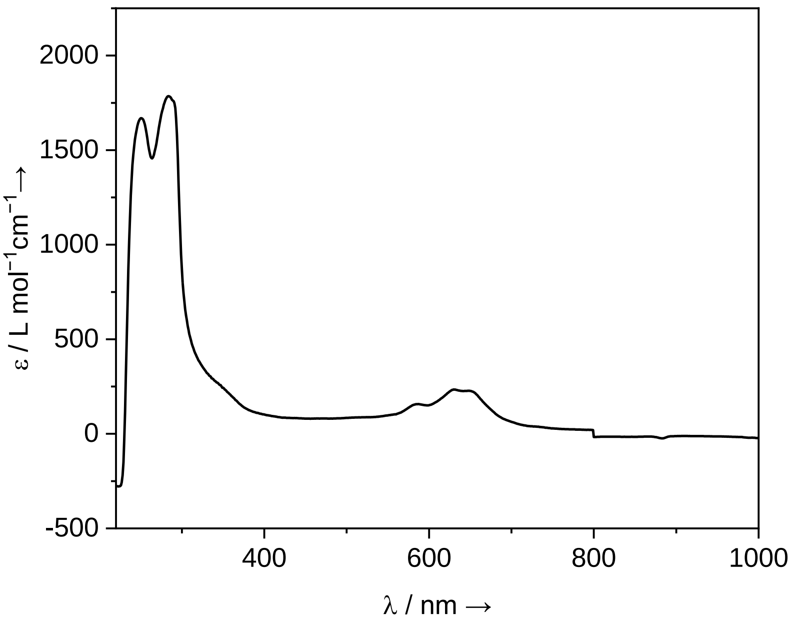

Figure S113: UV-Vis spectrum of [$CoBr_2$(L8)] in DCM.

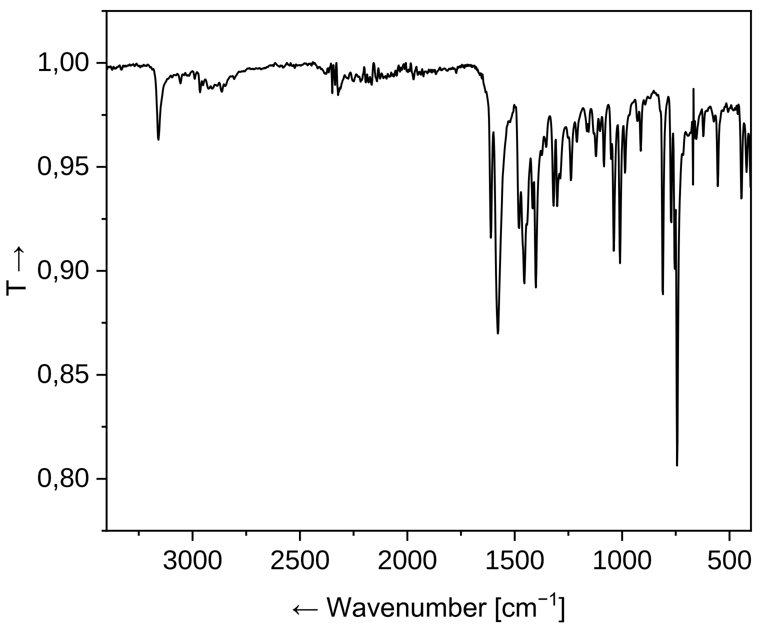

Figure S114: IR spectrum of [$CoBr_2$(L8)].

Table S19: Molecular view of [$CoBr_2$(L8)], as well as crystallographic data. Most H atoms have been omitted for clarity. Displacement ellipsoids correspond to a 50% probability of occurrence. Colour coding: C dark-grey, N blue, H light-grey, Co violet, Br brown.

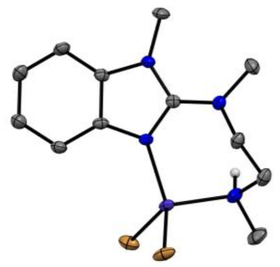

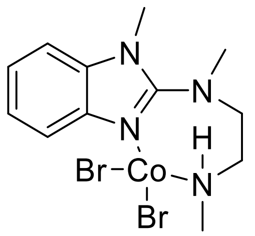

Empirical formula	$C_{12}H_{18}Br_2CoN_4$
Formula weight	437.05
Temperature/K	100.00
Crystal system	monoclinic
Space group	$P2_1/c$
a/Å	9.2779(6)
b/Å	11.7825(9)
c/Å	15.2425(11)
α/°	90
β/°	107.134(3)

γ/°	90
Volume/Å^3	1592.3(2)
Z	4
ρ_{calc}g/cm^3	1.823
μ/mm^{-1}	6.089
F(000)	860.0
Crystal size/mm^3	0.29 × 0.24 × 0.22
Radiation	MoKα (λ = 0.71073)
2Θ range for data collection/°	4.446 to 54.998
Index ranges	-12 ≤ h ≤ 12, -15 ≤ k ≤ 15, -19 ≤ l ≤ 19
Reflections collected	66046
Independent reflections	3639 [R_{int} = 0.1042, R_{sigma} = 0.0500]
Data/restraints/parameters	3639/0/179
Goodness-of-fit on F^2	1.040
Final R indexes [I>=2σ (I)]	R_1 = 0.0304, wR_2 = 0.0748
Final R indexes [all data]	R_1 = 0.0388, wR_2 = 0.0796
Largest diff. peak/hole / e Å^{-3}	0.65/-0.58

11.6 [$ZnCl_2$(L8)]

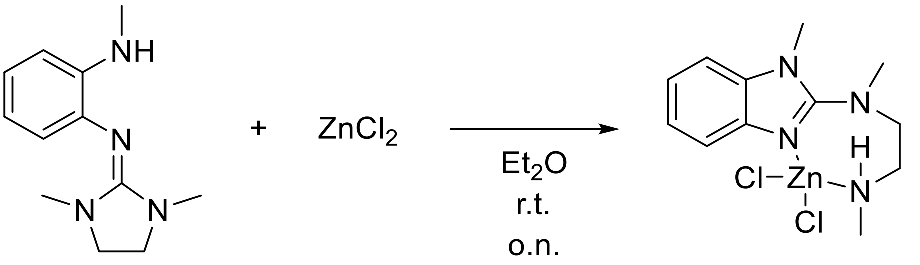

2-((1,3-dimethylimidazolidin-2-ylidene)amino)-N-methylaniline (L8) (20.0 mg, 1.00 equiv., 91.6 µmol) and zinc chloride (12.5 mg, 1.00 equiv., 91.6 µmol) were dissolved in 2.00 ml of diethyl ether and stirred overnight at room temperature. The solvent was filtered off and the light purple solid was washed with 2×2.00 ml diethyl ether. The light purple solid was obtained in quantitative yield (32.5 mg, 91.6 µmol). Crystals were obtained by controlled evaporation of a suspension in acetone in air. Due to its low solubility, only a UV-Vis is available as a liquid analysis.

UV-Vis (DCM, $c = 7.85 \cdot 10^{-5}$ mol·l^{-1}, d = 1 cm): λ_{max} (ε [l·mol^{-1}·cm^{-1}]) =246 (1034), 285 (1536), 291 (1563) nm.

IR (ATR): $\tilde{\nu}$ = 3187.36, 3058.10, 2968.79, 2921.20, 2866.86, 1640.54, 1611.26, 1581.00, 1511.40, 1478.98, 1459.17, 1418.95, 1404.21, 1355.82, 1320.80, 1304.94, 1293.71, 1239.06, 1213.82, 1165.48, 1156.58, 1124.79, 1102.12, 1087.60, 1053.67, 1041.68, 1011.48, 988.62, 928.64, 913.72, 897.47, 830.26, 813.03, 770.67, 756.47, 744.74, 719.46, 678.96, 670.19, 655.95, 621.64, 556.53, 481.10, 446.91, 422.99 cm^{-1}.

Elemental analysis (%): [$ZnCl_2$(L8)] • 0.5 H_2O

calculated:	C: 39.64	H: 5.27	N: 15.41
found:	C: 39.13	H: 4.95	N: 14.85

Analytical data:

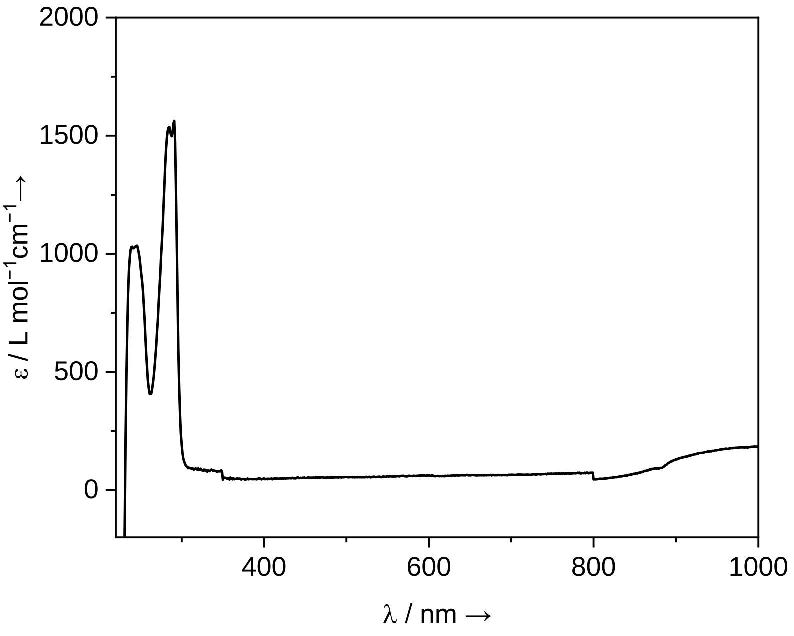

Figure S115: UV-Vis spectrum of [$ZnCl_2$(L8)] in DCM.

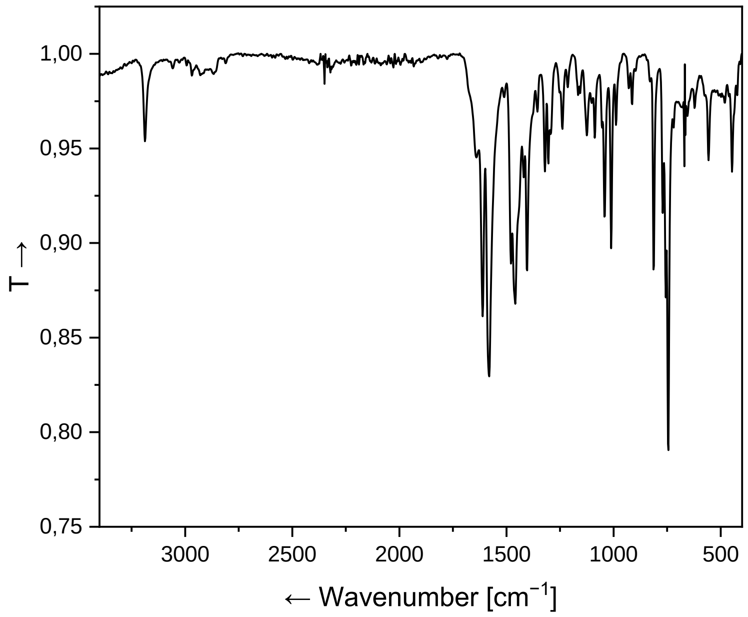

Figure S116: IR spectrum of [$ZnCl_2$(L8)].

Table S20: Molecular view of [$ZnCl_2$(L8)], as well as crystallographic data. Most H atoms have been omitted for clarity. Displacement ellipsoids correspond to 50% probability of residence. Colour coding: C dark-grey, N blue, H light-grey, Zn violet, Cl green.

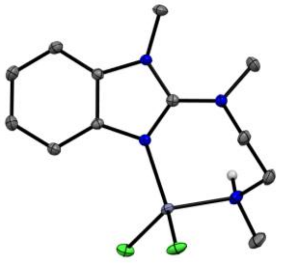

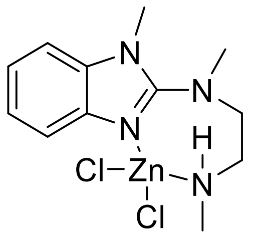

Empirical formula	$C_{12}H_{18}N_4Cl_2Zn$
Formula weight	354.57
Temperature/K	100.00
Crystal system	monoclinic
Space group	$P2_1/n$
a/Å	9.3045(4)
b/Å	11.2344(4)
c/Å	15.0653(6)
α/°	90
β/°	106.105(2)
γ/°	90
Volume/Å^3	1512.98(10)
Z	4
ρ_{calc}g/cm^3	1.557
μ/mm^{-1}	1.968
F(000)	728.0
Crystal size/mm^3	0.34 × 0.27 × 0.22
Radiation	MoKα (λ = 0.71073)

2Θ range for data collection/°	4.59 to 57.55
Index ranges	-12 ≤ h ≤ 12, -15 ≤ k ≤ 15, -20 ≤ l ≤ 20
Reflections collected	85165
Independent reflections	3935 [R_{int} = 0.0545, R_{sigma} = 0.0191]
Data/restraints/parameters	3935/0/179
Goodness-of-fit on F^2	1.046
Final R indexes [I>=2σ (I)]	R_1 = 0.0188, wR_2 = 0.0490
Final R indexes [all data]	R_1 = 0.0195, wR_2 = 0.0494
Largest diff. peak/hole / e $Å^{-3}$	0.48/-0.28

11.7 [$ZnCl_2$(L9)]

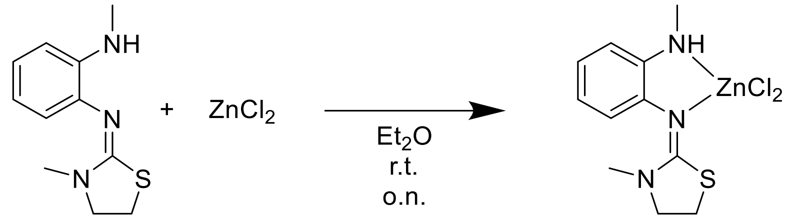

Zinc chloride (14.8 mg, 109 µmol, 1.00 eq.) and L9 (24.1 mg, 109 µmol, 1.00 eq.) were dissolved in 2.00 mL diethyl ether. The suspension was stirred overnight and the solid was then filtered off. This was washed with 3 × 1.00 mL with diethyl ether and solvent residues were removed under vacuum to obtain the product as a pink solid in quantitative yield (38.7 mg, 109 µmol). Crystals were obtained by partial removal of acetone.

^{1}H NMR (600 MHz, CD_3CN): δ = 7.42 – 7.27 (m, 1 H, C*H*$_{arom}$), 7.23 (t, *J* = 9.1 Hz, 3 H, 3 × C*H*$_{arom}$), 7.16 (s, 1 H, N*H*), 3.94 (t, *J* = 7.7 Hz, 2 H, C*H*$_2$), 3.37 (dd, *J* = 8.7, 6.5 Hz, 2 H, C*H*$_2$), 3.32 (s, 3 H, C*H*$_3$), 2.71 (s, 3 H, C*H*$_3$) ppm.

CV:

Irreversible oxidation at: E_{Ox1}: -1.01 V, E_{Ox2}: 0.18 V, E_{Ox3}: 0.51 V, E_{Ox4}: 0.77 V, E_{Ox5}: 1.43 V.

Irreversible reduction at: E_{Red1}: 1.31 V, E_{Red2}: 0.67 V, E_{Red2}: -2.19 V.

UV-Vis (DCM, $c = 9.30 \cdot 10^{-5}$ mol·l^{-1}, d = 1 cm): λ_{max} (ε [l·mol^{-1}·cm^{-1}]) =215 nm (1496), 286 nm (1368) nm.

IR (ATR): $\tilde{v}$ = 3478.53, 3398.50, 3195.27, 2996.42, 2876.68, 2820.86, 1628.18, 1603.74, 1574.55, 1501.91, 1467.28, 1439.61, 1415.06,1319.82, 1298.89, 1256.53, 1163.64, 1143.69, 1093.49, 1039.53, 1013.85, 999.92, 955.66, 897.62, 796.87, 758.71, 675.95, 637.78, 618.74, 578.50, 567.24, 537.94, 511.49, 501.62, 478.12, 467.51, 442.43, 426.77, 408.09 cm^{-1}.

Elemental analysis (%):

calculated:	C: 36.95	H: 4.23	N: 11.75
found:	C: 35.98	H: 4.21	N: 11.28

Analytical data:

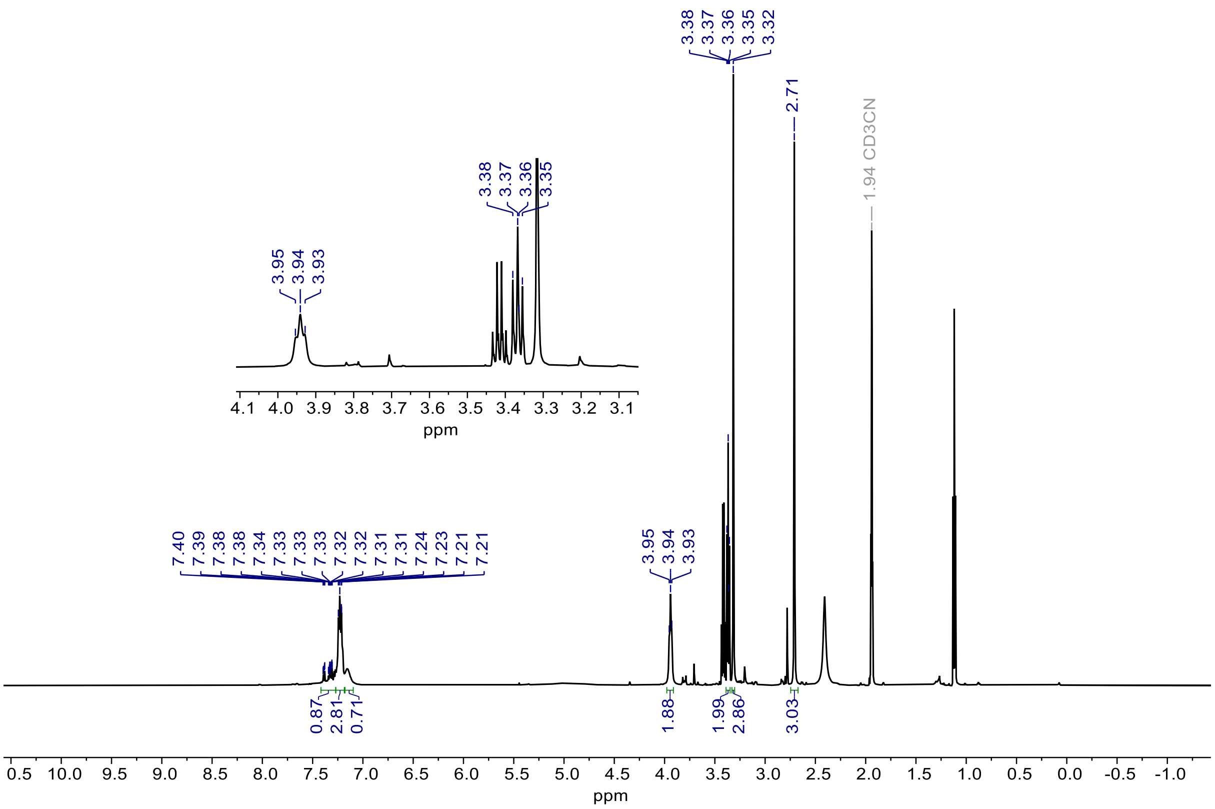

Figure S117: ^{1}H NMR spectrum (600 MHz, CDCN) of [$ZnCl_2$(L9)]. The impurities at 1.12 ppm and 3.42 ppm are due to diethylether

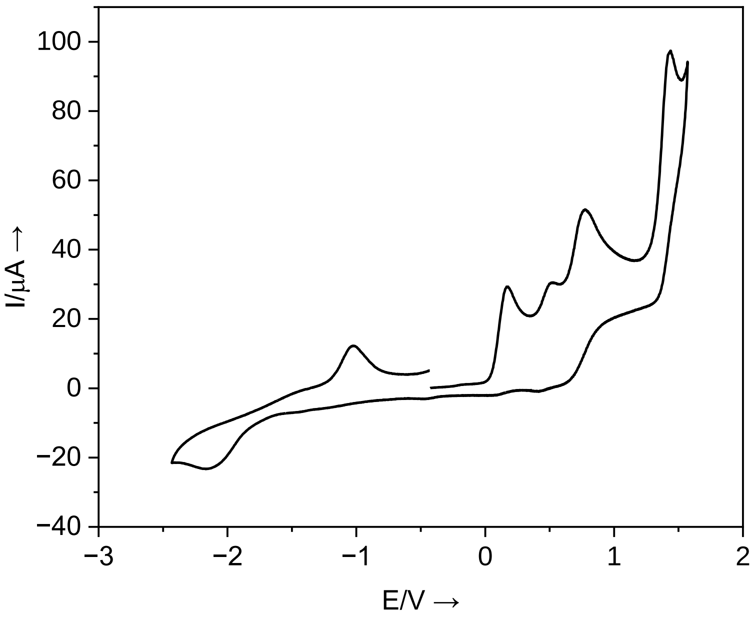

Figure S 118: Cyclovoltammogram of [$ZnCl_2$(L9)] in acetonitrile ([*n*-Bu_4N][PF_6], 100 mV/s internally referenced to Fc^+/Fc).

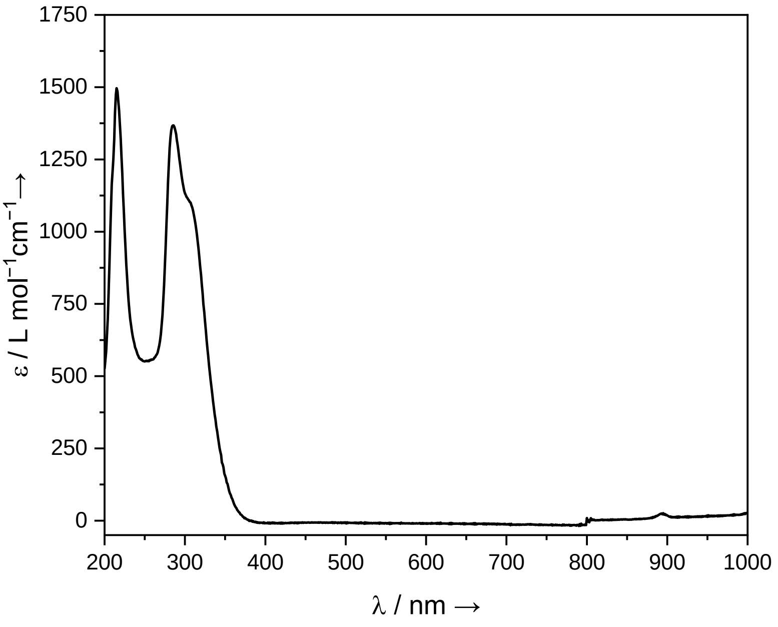

Figure S119: UV-Vis spectrum of [$ZnCl_2$(L9)] in DCM.

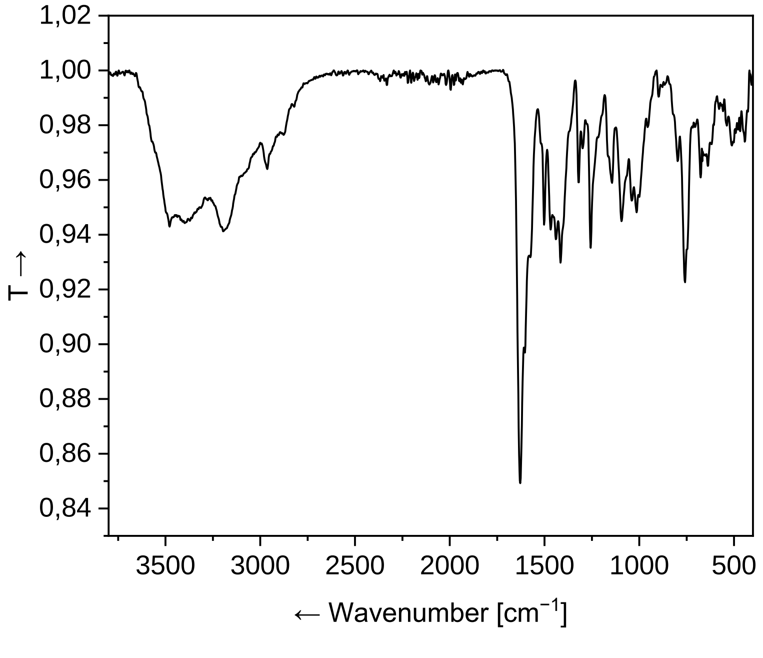

Figure S120: IR spectrum of [$ZnCl_2$(L9)].

Table S21: Molecular view of [$ZnCl_2$(L9)], as well as crystallographic data. Most H atoms have been omitted for clarity. Displacement ellipsoids correspond to 50% probability of residence. Colour coding: C dark-grey, N blue, H light-grey, Zn violet, Cl green.

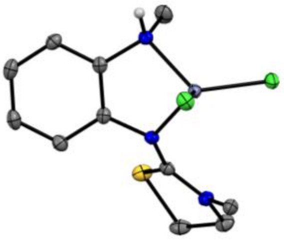

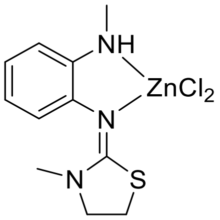

Empirical formula	$C_{11}H_{15}N_3SCl_2Zn$
Formula weight	357.59
Temperature/K	100.00
Crystal system	monoclinic
Space group	$P2_1/n$
a/Å	11.8638(10)
b/Å	8.1139(7)
c/Å	15.3049(12)
α/°	90
β/°	98.671(3)
γ/°	90
Volume/Å^3	1456.4(2)
Z	4
ρ_{calc}g/cm^3	1.631
μ/mm^{-1}	2.181
F(000)	728.0
Crystal size/mm^3	0.29 × 0.18 × 0.16
Radiation	MoKα (λ = 0.71073)

2Θ range for data collection/°	4.06 to 55.222
Index ranges	-15 ≤ h ≤ 15, -10 ≤ k ≤ 10, -19 ≤ l ≤ 19
Reflections collected	80503
Independent reflections	3381 [R_{int} = 0.0630, R_{sigma} = 0.0225]
Data/restraints/parameters	3381/1/169
Goodness-of-fit on F^2	1.097
Final R indexes [I>=2σ (I)]	R_1 = 0.0242, wR_2 = 0.0563
Final R indexes [all data]	R_1 = 0.0277, wR_2 = 0.0584
Largest diff. peak/hole / e $Å^{-3}$	0.52/-0.28

11.8 [$CoBr_2$(L2)]

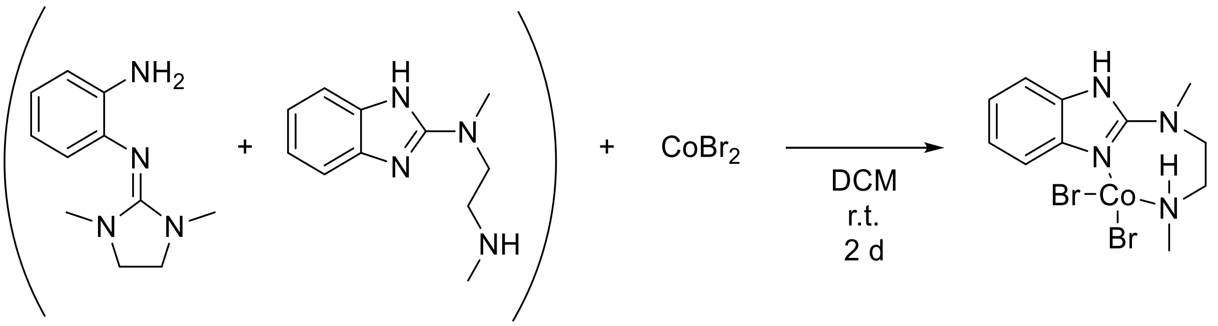

A mixture of 2-((1,3-dimethylimidazolidin-2-ylidene)amino)aniline (L2) and N^1-(1*H*-benzo[*d*]imidazol-2-yl)-N^1,N^2-dimethylethane-1,2-diamine (32.0 mg, 1.00 eq., 156 µmol) and cobalt bromide (34.3 mg, 1.00 eq., 156 µmol) were dissolved in 2.00 mL of dichloromethane and stirred for 2 days at room temperature. The solvent was removed from the blue suspension under reduced vacuum and the light blue solid was washed with 2 × 2.00 mL of diethyl ether. The product was obtained as blue powder in quantitative yield (66.3 mg, 156 µmol). Light blue crystals were obtained by layering a suspension in acetonitrile with diethyl ether at -31°C. Due to poor solubility, no liquid analysis was performed.

IR: (ATR): $\tilde{\nu}$ = 3276.50, 3170.10, 2957.06, 2924.73, 1628.13, 1610.04, 1593.35, 1471.61, 1446.23, 1438.21, 1431.12, 1383.63, 1355.99, 1307.67, 1273.90, 1261.45, 1222.98, 1200.65, 1087.11, 1058.68, 1031.27, 1011.42, 997.90, 898.18, 806.37, 759.07, 735.18, 647.99, 592.85, 578.56, 542.45, 456.74, 436.34, 407.87 cm^{-1}.

Elemental analysis (%): [$CoBr_2$(L2)] • 1 H_2O

calculated:	C: 29.96	H: 4.41	N: 12.70
found:	C: 29.40	H: 3.83	N: 12.34

Analytical data:

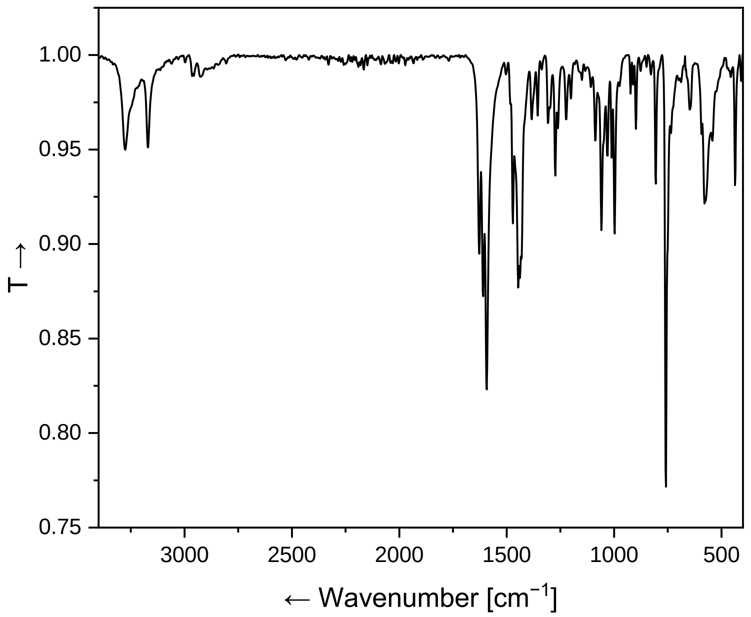

Figure S121: IR spectrum of $[CoBr_2(L2)]$.

Table S22: Molecular view of $[CoBr_2(L2)]$, as well as crystallographic data. Most H atoms have been omitted for clarity. Displacement ellipsoids correspond to a 50% probability of occurrence. Colour coding: C dark-grey, N blue, H light-grey, Co violet, Br brown.

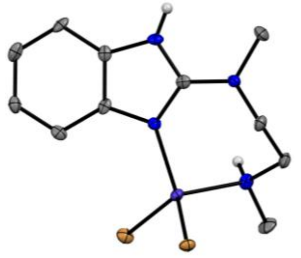

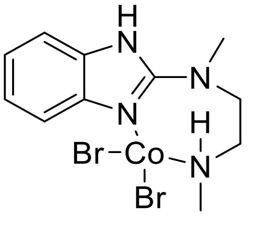

Empirical formula	$C_{11}H_{16}N_4CoBr_2$
Formula weight	423.03
Temperature/K	100.00
Crystal system	triclinic
Space group	P-1
a/Å	8.5727(9)
b/Å	9.0033(10)

c/Å	11.0190(11)
α/°	90.826(4)
β/°	110.447(4)
γ/°	113.736(4)
Volume/$Å^3$	717.47(13)
Z	2
ρ_{calc}g/cm^3	1.958
μ/mm^{-1}	6.753
F(000)	414.0
Crystal size/mm^3	0.17 × 0.09 × 0.05
Radiation	MoKα (λ = 0.71073)
2Θ range for data collection/°	4.01 to 52.996
Index ranges	-10 ≤ h ≤ 10, -11 ≤ k ≤ 11, -13 ≤ l ≤ 13
Reflections collected	30632
Independent reflections	2972 [R_{int} = 0.0990, R_{sigma} = 0.0550]
Data/restraints/parameters	2972/0/169
Goodness-of-fit on F^2	1.068
Final R indexes [I>=2σ (I)]	R_1 = 0.0377, wR_2 = 0.0831
Final R indexes [all data]	R_1 = 0.0479, wR_2 = 0.0880
Largest diff. peak/hole / e $Å^{-3}$	0.63/-1.06

11.9 [$ZnCl_2$(L5)]

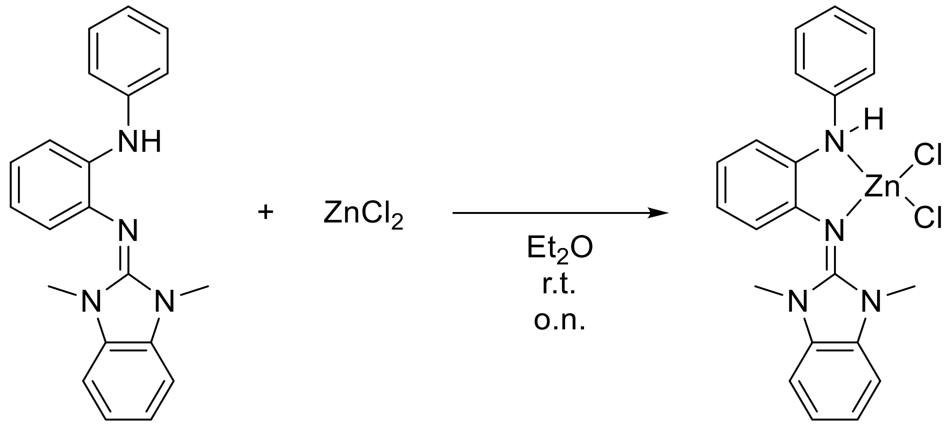

2-((1,3-dimethyl-1,3-dihydro-2*H*-benzo[*d*]imidazol-2-ylidene)amino)-*N*-phenylaniline (L5) (21.0 mg, 1.00 eq., 63.9 µmol) and zinc chloride (8.71 mg, 1.00 eq., 63.9 µmol) were dissolved in 2.00 mL of diethyl ether and stirred overnight at room temperature. The pink solid was washed with 3 × 2.00 mL of diethyl ether and then dried under a fine vacuum. The product was obtained in quantitative yield (29.7 mg, 63.9 µmol) as a pink powder. Crystals were obtained by dissolving in dichloromethane and layering with *n*-hexane.

^{1}H NMR (600 MHz, CD_2Cl_2): δ = 7.41 – 7.30 (m, 6 H, CH_{arom}), 7.30 – 7.24 (m, 1 H, CH_{arom}), 7.22 – 7.18 (m, 1 H, CH_{arom}), 7.17 – 7.13 (m, 1 H, CH_{arom}), 7.13 – 7.07 (m, 2 H, CH_{arom}), 6.97 (td, *J* = 7.8, 1.4 Hz, 1 H, CH_{arom}), 6.85 (dd, *J* = 8.1, 1.4 Hz, 1 H, CH_{arom}), 6.17 (s, 1 H, N*H*), 3.65 (s, 6 H, 2×CH_3) ppm.

^{13}C NMR (151 MHz, CD_2Cl_2): δ = 153.67 ($C_{q,Guan}$), 144.38 (d, *J* = 14.3 Hz, $C_{q,arom}$), 133.69 ($C_{q,arom}$), 131.72 ($C_{q,arom}$), 129.84 (CH$_{arom}$), 129.04 (CH$_{arom}$), 127.57 (CH$_{arom}$), 125.58 (CH$_{arom}$), 124.38 (CH$_{arom}$), 122.41 (CH$_{arom}$), 121.23 (CH$_{arom}$), 119.55 (CH$_{arom}$), 110.33 (CH$_{arom}$), 32.54 (NCH$_3$) ppm.

CV:

Irreversible oxidation at: E_{Ox1}: 0.91 V.

Irreversible reduction at: E_{Red1}: 0.12 V, E_{Red2}: 0.34 V, E_{Red2}: -0.76 V.

UV-Vis (DCM, *c* = 6.67098·10^{-5} mol·l^{-1}, *d* = 1 cm): λ_{max} (*ε* [l·mol^{-1}·cm^{-1}]) =242 (2972), 264 (2824), 313 (4663) nm.

IR (ATR): ṽ = 3131.47, 3071.58, 1600.61, 1564.90, 1494.76, 1482.47, 1431.22, 1376.95, 1337.48, 1299.66, 1256.55, 1229.13, 1182.54, 1161.81, 1140.50, 1109.78, 1029.12, 885.56, 855.74, 804.49, 769.47, 749.57, 723.79, 694.19, 630.86, 608.30, 561.64, 521.88, 475.54.

Elemental analysis (%): $ZnCl_2L5 \cdot 0.5\ H_2O$

calculated:	C: 53.25	H: 4.47	N: 11.83
found:	C: 53.35	H: 4.13	N: 12.01

Analytical data:

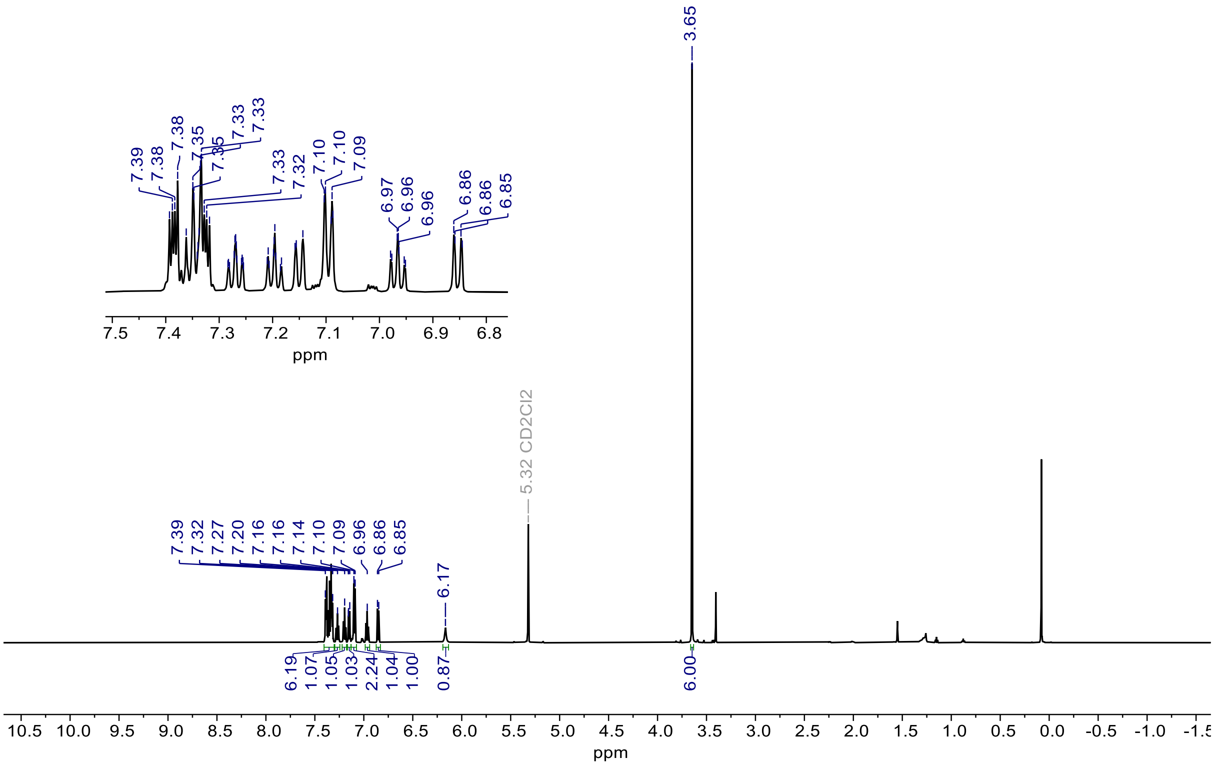

Figure S122: ^{1}H NMR spectrum (600 MHz, CD_2Cl_2) of $[ZnCl_2(L5)]$. The impurities at 1.55 ppm are due to water and at 3.40 ppm to deactivated urea derivative. The impurities at 1.22 ppm, 0.88 ppm and 0.07 ppm are due to grease.

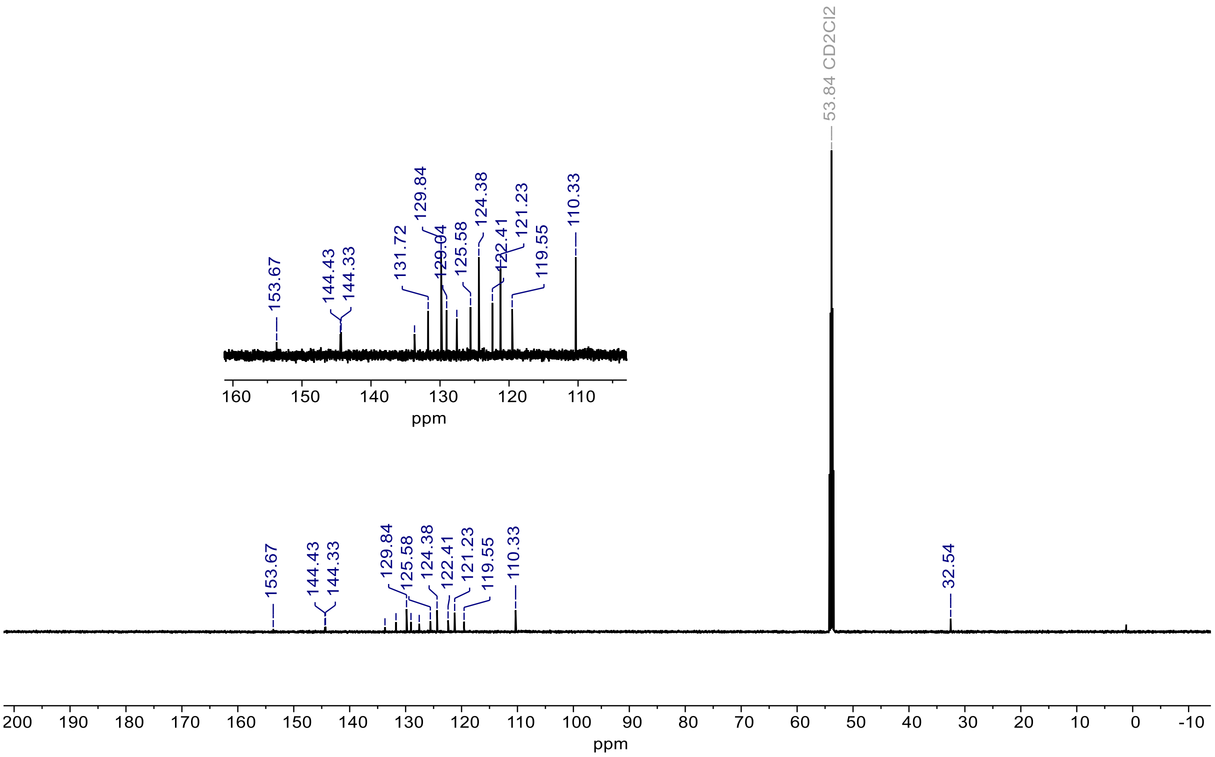

Figure S123: ^{13}C NMR spectrum (151 MHz, CD_2Cl_2) of [$ZnCl_2$(L5)].

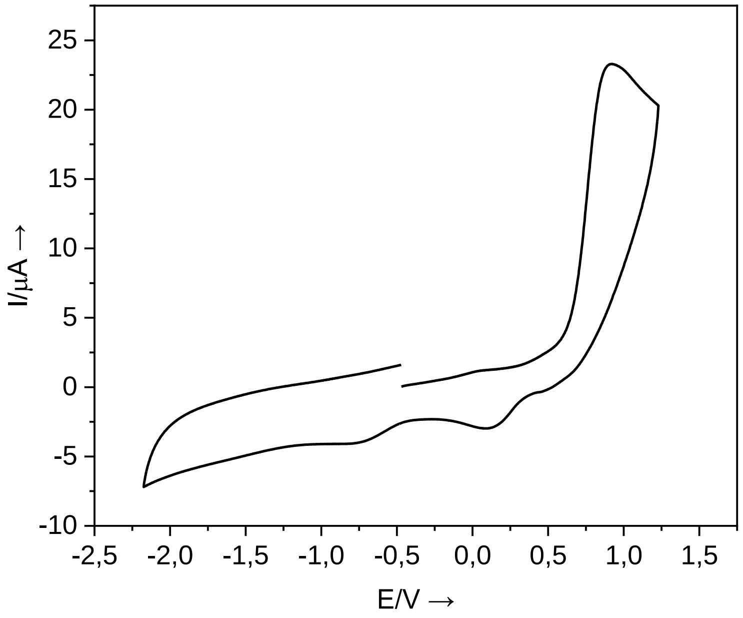

Figure S124: Cyclovoltammogram of [$ZnCl_2$(L5)] in dichloromethane ([*n*-Bu_4N][PF_6], 50 mV/s internally referenced to Fc^+/Fc).

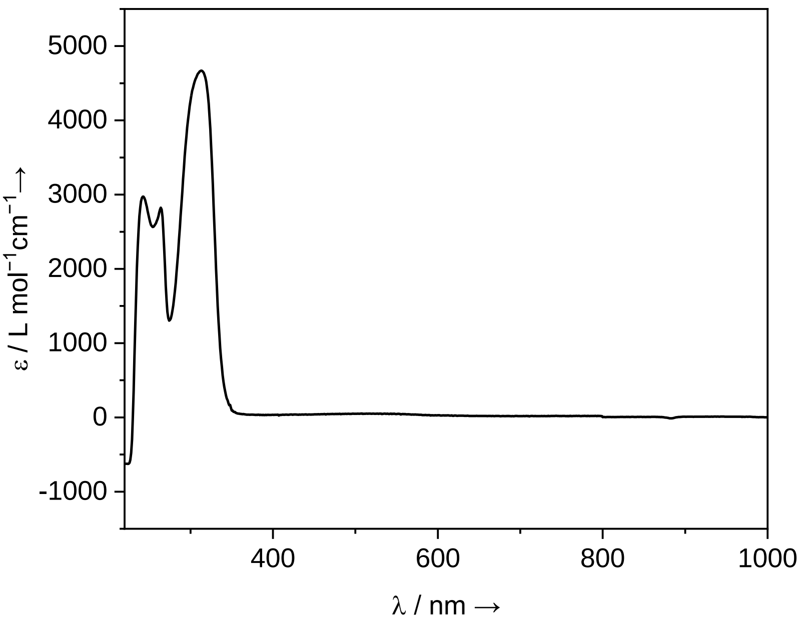

Figure S125: UV-Vis spectrum of [$ZnCl_2$(L5)] in DCM.

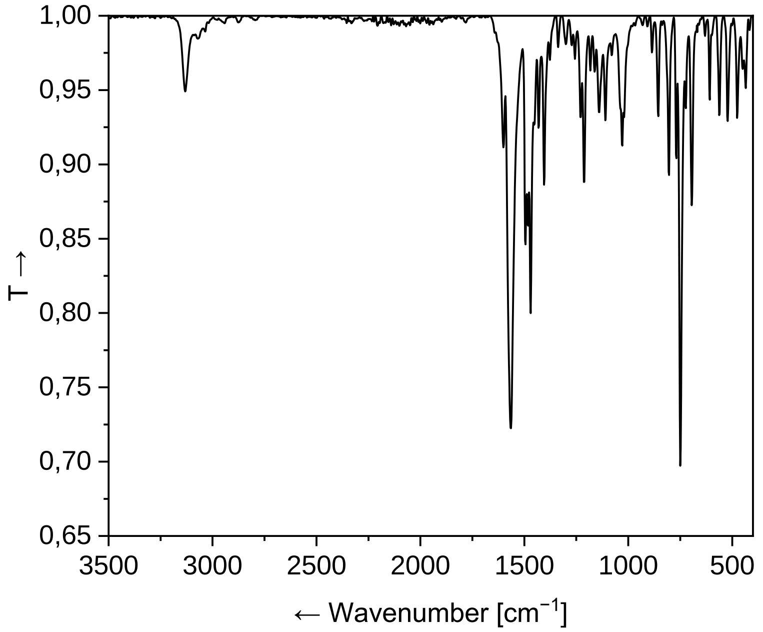

Figure S126: IR spectrum of [$ZnCl_2$(L5)].

Table S23: Molecular view of [$ZnCl_2$(L5)], as well as crystallographic data. Most H atoms have been omitted for clarity. Displacement ellipsoids correspond to a 50% probability of occurrence. Colour coding: C dark-grey, N blue, H light-grey, Zn violet, Cl green.

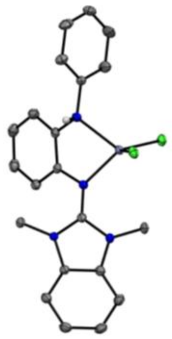

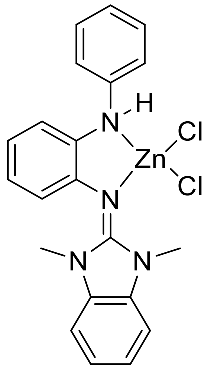

Empirical formula	$C_{21.5}H_{21}Cl_3N_4Zn$
Formula weight	507.14
Temperature/K	100.00
Crystal system	triclinic
Space group	P-1
a/Å	8.9431(4)
b/Å	11.6801(6)
c/Å	12.4641(6)
α/°	117.123(2)
β/°	102.638(2)
γ/°	95.989(2)
Volume/Å^3	1099.04(9)
Z	2
ρ_{calc}g/cm^3	1.532
μ/mm^{-1}	1.498
F(000)	518.0

Crystal size/mm^3	0.413 × 0.109 × 0.096
Radiation	MoKα (λ = 0.71073)
2Θ range for data collection/°	3.85 to 55.964
Index ranges	-11 ≤ h ≤ 11, -15 ≤ k ≤ 15, -16 ≤ l ≤ 16
Reflections collected	61013
Independent reflections	5289 [R_{int} = 0.0798, R_{sigma} = 0.0334]
Data/restraints/parameters	5289/0/259
Goodness-of-fit on F^2	1.053
Final R indexes [I>=2σ (I)]	R_1 = 0.0333, wR_2 = 0.0765
Final R indexes [all data]	R_1 = 0.0384, wR_2 = 0.0797
Largest diff. peak/hole / e Å^{-3}	0.44/-0.40

12 NMR Experiments

To comprehend whether this was an irreversible rearrangement or an equilibrium, a mixture of 2-((1,3-dimethylimidazolidin-2-ylidene)amino)aniline (marked with red blocks in the ^{1}H NMR) and *N*^{1}-(1*H*-benzo[*d*]imidazol-2-yl)-*N*^{1},*N*^{2}-dimethylethane-1,2-diamine (marked with blue blocks in the ^{1}H NMR) was dissolved in the protic solvent D_2O. As can be seen in **Figure S127** above, only signals for the rearranged species are visible. The solvent was then removed and $CDCl_3$ was used as the solvent. As can be seen in **Figure S127** below, the same slightly shifted signals occur.

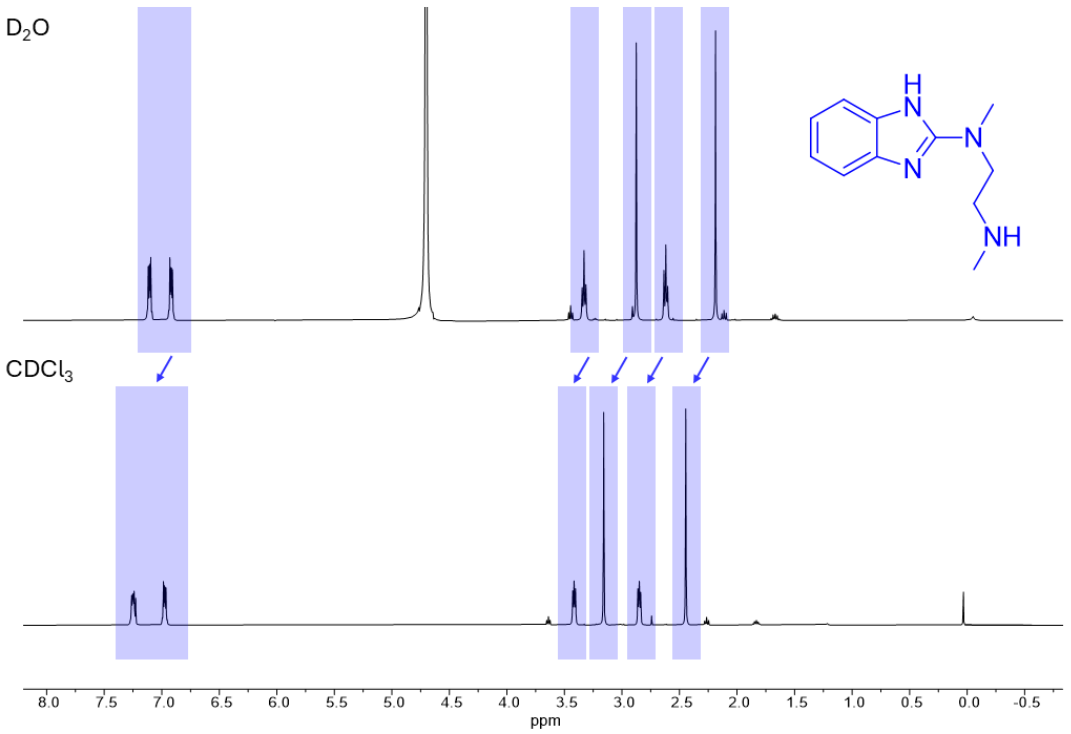

Figure S127: NMR experiment: Two ^{1}H NMR spectra are shown. D_2O was chosen as the solvent at the top, while $CDCl_3$ was used at the bottom. The signals of species *N*^{1}-(1*H*-benzo[*d*]imidazol-2-yl)-*N*^{1},*N*^{2}-dimethylethane-1,2-diamine are marked with blue boxes. Impurities include THF and grease.

For comparison, **Figure S128** shows a comparison of two ^{1}H NMRs. The upper spectrum was recorded in $CDCl_3$ without first dissolving it in D_2O. Here, the rearranged species *N*^{1}-(1*H*-benzo[*d*]imidazol-2-yl)-*N*^{1},*N*^{2}-dimethylethane-1,2-diamine can be found (marked with blue boxes), as well as the non-rearranged species 2-((1,3-dimethylimidazolidin-2-ylidene)amino)aniline (marked with red boxes). The lower spectrum, in which the mixture of substances was first dissolved in D_2O, shows only one species (*N*^{1}-(1*H*-benzo[*d*]imidazol-2-yl)-*N*^{1},*N*^{2}-dimethylethane-1,2-diamine, marked with blue boxes).

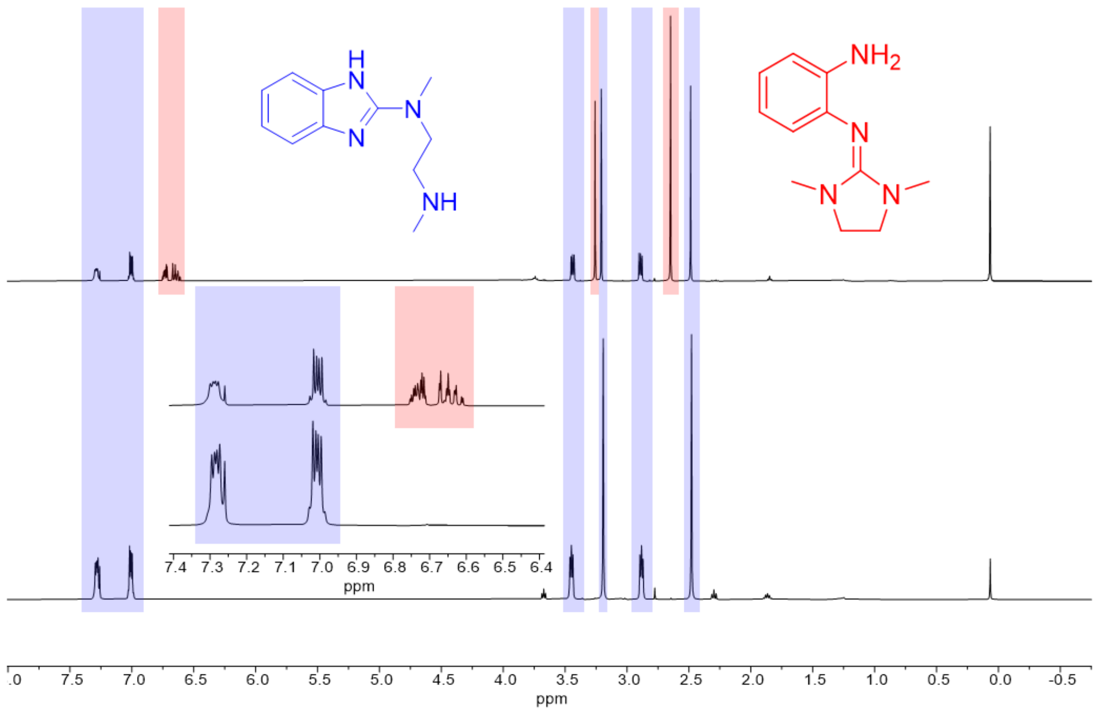

Figure S128: NMR experiment: Two ^{1}H NMR spectra are shown. $CDCl_3$ was chosen as the solvent for both, but the bottom was first dissolved in D_2O. The signals of species *N*[1]-(1*H*-benzo[*d*]imidazol-2-yl)-*N*[1],*N*[2]-dimethylethane-1,2-diamine are marked with blue boxes., while the signals for 2-((1,3-dimethylimidazolidin-2-ylidene)amino)aniline are marked with red boxes. Impurities include THF and grease.

In order to further investigate the influence of small amounts of water on the rearrangement, a sample was measured in $CDCl_3$ (see **Figure S129**, bottom spectrum) and then 10 µl of D_2O was added and measured again (see **Figure S129**, top spectrum). However, no change in the ratios of the two species was observed.

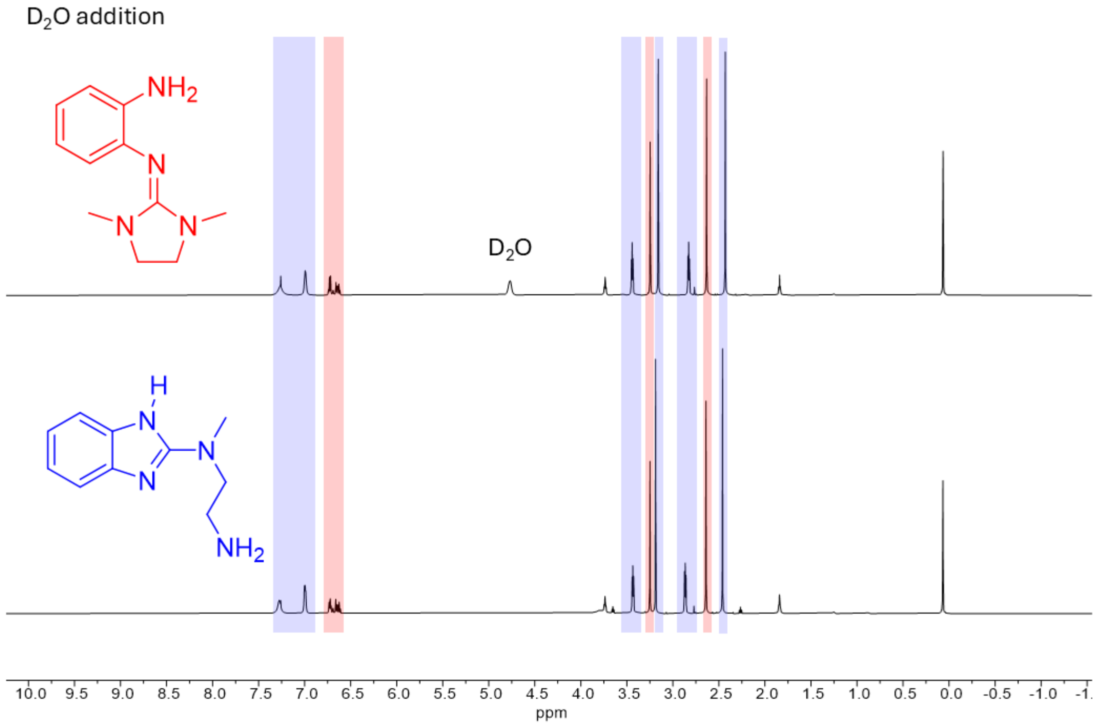

Figure S129: Two ^{1}H NMR (600 MHz) spectra are shown. $CDCl_3$ was chosen as the solvent for both, but 10 µl D_2O was added and measured again (shown as the top spectrum). The signals of species *N*[1]-(1*H*-benzo[*d*]imidazol-2-yl)-*N*[1],*N*[2]-dimethylethane-1,2-diamine are marked with blue boxes., while the signals for 2-((1,3-dimethylimidazolidin-2-ylidene)amino)aniline are marked with red boxes. Impurities include THF and grease.

13 Cartesian coordinates of optimized structures

13.1 Details on Quantum Chemical Calculations

DFT calculations were performed with ORCA 5.0.4.[1,2] All structures depicted in this section are calculated with the functional basis set combination B3LYP[3,4]/def2-TZVP[5] in combination with dispersion correction D3[6], without the influence of a polarizable environment. As starting point for the structure optimizations existing crystal structure coordinates were used. For some calculations, the crystal structures were adapted using Avogadro 1.2.0. The calculated structures were visualized with the Mercury 4.2.0 software. The electronic energy E_{el} is given in Hartree.

---

[1] F. Neese, *Wiley Interdiscip. Rev.: Comput. Mol. Sci.*, **2012**, *2*, *1*, 73–78.

[2] F. Neese, *Wiley Interdiscip. Rev.: Comput. Mol. Sci.*, **2022**, *12*, *1*, e1606.

[3] A. D. Becke, *J. Chem. Phys.* **1993**, *98*, 5648–5652.

[4] P. J. Stephens, F. J. Devlin, C. F. Chabalowski, M. J. Frisch, *J. Phys. Chem.* **1994**, *98*, 11623–11627.

[5] F. Weigend, R. Ahlrichs, *Phys. Chem. Chem. Phys*. **2005**, *7*, 3297-3305.

[6] S. Grimme, J. Antony, S. Ehrlich, H. Krieg, *J. Chem. Phys.* **2010**, *132*, 154104.

13.2 Tables with calculated thermodynamic properties

Table S24: Calculated changes in Gibbs free energy, enthalpy, and entropy (at 298 K, 1 bar) upon cyclization of the primary amines to give the 2-amino-benzimidazole isomers.

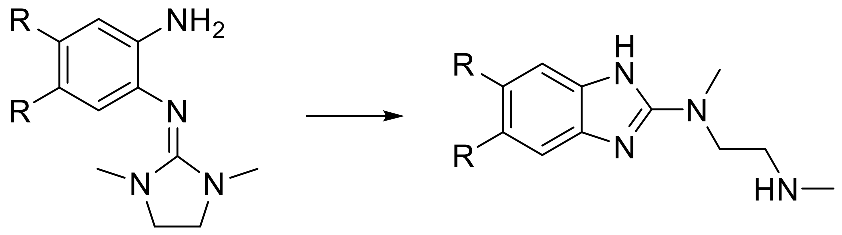

R	Δ*G* [kJ/mol]	Δ*H* [kJ/mol)]	Δ*S* [J/mol•K]
H	−5.7	−8.0	−7.6
F	−13.7	−13.9	−0.6
Cl	−5.7	−7.6	−6.2
Me	−6.9	−9.5	−8.9
OMe	−8.6	−10.4	−6.1

Table S25: Calculated changes in Gibbs free energy, enthalpy, and entropy (at 298 K, 1 bar) upon conversion of the tetramethylguanidine molecules L1, L3 and L7 with different groups R on the amine (R = H, Me, or Ph) into the benzimidazole species and dimethylamine.

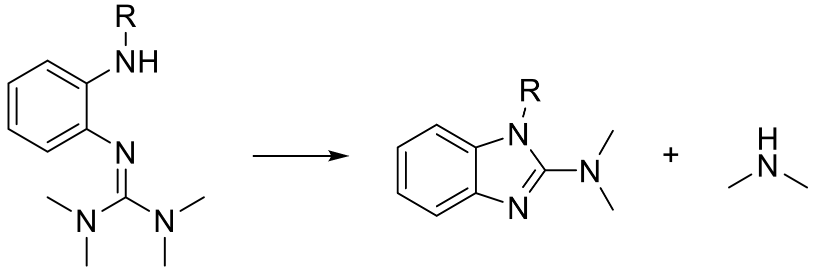

R	Δ*G* [kJ/mol]	Δ*H* [kJ/mol)]	Δ*S* [J/mol•K]
H	−49.9	2.4	175
Me	−52.5	−3.1	165
Ph	−45.2	3.5	163

Table S26: Calculated changes in Gibbs free energy, enthalpy, and entropy (at 298 K, 1 bar) of the ligands L4, L6, L8 and L9 with different residues on the amine (-Ph, -Me), as well as the rearranged benzimidazole species.

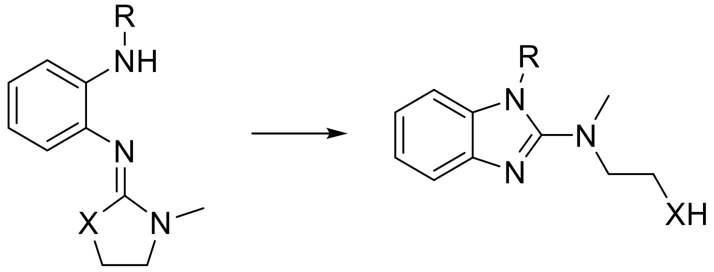

R, X	ΔG [KJ/mol]	ΔH [KJ/mol)]	ΔS[J/mol•K]
Ph, NMe	8.7	7.9	−2.9
Ph, S	7.3	4.5	−9.3
Me, NMe	1.1	−2.1	−10.7
Me, S	−6.7	−8.7	−6.5

Table S27: Calculated changes in Gibbs free energy, enthalpy, and entropy (at 298 K, 1 bar) for conversion of L5 and L10 into the benzimidazole isomers.

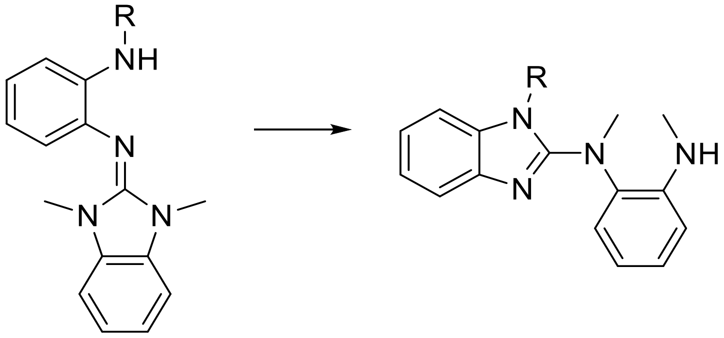

	ΔG [KJ/mol]	ΔH [KJ/mol)]	ΔS[J/mol•K]
L5 (R = Ph)	30.9	27.4	−9.9
L10 (R = Me)	23.2	20.6	−8.7

13.3 Primary amines

13.3.1 2-((1,3-dimethylimidazolidin-2-ylidene)amino)aniline

Illustration of the molecular structure. C-H hydrogen atoms are omitted. Colour code: C dark-grey, H light-grey, N blue.

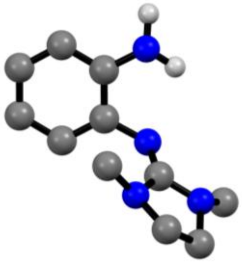

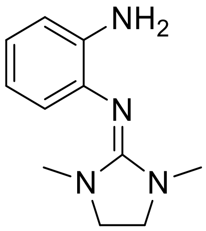

E_{el} = −647.70980753

Cartesian coordinates (in Å)

N	7.33713136027610	5.24762253103606	2.12494785644294
N	5.10264426104536	4.70119396960677	1.77206283465590
N	5.78047225205653	6.66647132505726	0.91771165812668
C	8.50930809929813	5.97713382108589	1.90327382926119
C	9.15041624393555	6.56376896250701	3.01436038137801
C	10.35987442087838	7.23276131582668	2.83027982672643
H	10.84174537036745	7.69362028623724	3.68549405577580
C	10.94155511799699	7.32225536641777	1.57123765049273
H	11.88174296388871	7.84527201342109	1.45128193597647
C	10.31994615598526	6.73479344254639	0.47565862653994
H	10.77101028809845	6.79056154483054	-0.50660516551884
C	9.11517477123480	6.06247924199881	0.65046415463856
H	8.62188264115469	5.59165584332042	-0.19100913123006
C	6.19453637967317	5.54698387669180	1.63423726843034
C	4.03952491592141	5.12534893644087	0.88013322323903
H	3.05542255434296	4.91885751036652	1.30282161678133
H	4.11166082233576	4.61813354649484	-0.09351769171186
C	4.33095830163919	6.61377998244679	0.74904808745990
H	4.03210800053917	7.02760667575546	-0.21463776065236
H	3.82771781750910	7.18186804193657	1.54372714055692

C	5.29787062509472	3.29110043361168	2.01666033815593
H	5.50759192865797	2.74042532223441	1.08921560907864
H	4.40054995754129	2.87332208257798	2.47566311979512
H	6.14182145069533	3.16655503340069	2.68968537501975
C	6.34266399434101	7.98478781536567	1.16402069642252
H	6.09360277454064	8.34930019191921	2.16847099950229
H	5.92805376584436	8.67489487079670	0.42873008637390
H	7.42151829876302	7.97305109935415	1.05418334351994
N	8.50990278111587	6.51993422788538	4.25206207931924
H	7.83612298058539	5.76833444209795	4.31186211220031
H	9.12356870464290	6.56589624673110	5.05063584324325

13.3.2 2-(2-aminophenyl)-1,1,3,3-tetramethylguanidine

Illustration of the molecular structure. C-H hydrogen atoms are omitted. Colour code: C dark-grey, H light-grey, N blue.

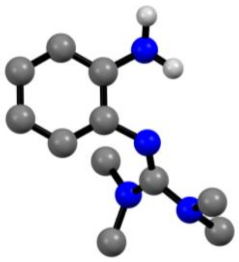

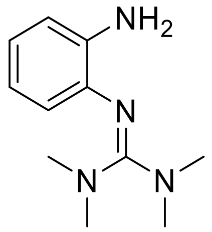

E_{el} = −648.90173550

Cartesian coordinates (in Å)

N	8.40040834092248	12.84497904881994	1.95949851469219
N	9.20545137646470	15.00487561241697	1.94292621859577
N	7.99394527934227	14.27028598622616	0.09121876404038
N	6.33440693762347	11.26633757722353	2.78342979712312
C	8.17548143853354	11.64411168545174	1.28016288343926
C	7.15848059372287	10.79386802272745	1.76324328455265
C	6.96006254446293	9.55302222802709	1.16034176510527
H	6.17155821385315	8.90727860644475	1.53073456160980
C	7.75117648965043	9.14392530111611	0.09276383901826
H	7.58244224569043	8.17444252757206	-0.35824462553761
C	8.75744926533283	9.97473450848944	-0.38480373102123
H	9.38544872775358	9.65934676337044	-1.20787455021037
C	8.96446949749978	11.21303831884446	0.21377301737321
H	9.76161736617261	11.85971978006664	-0.13318687051830
C	8.51970231911139	13.96884668539253	1.33770743356633
C	9.99375581624877	14.70425596190967	3.12246032921658
H	9.38173925392051	14.68623599117313	4.03266326454642
H	10.76400959918046	15.47025922068464	3.23694723031796
H	10.45964772091575	13.72921626879424	3.01338326715143
C	8.69769074115245	16.36572149155061	1.89328006711754
H	9.51645965665496	17.07528954490676	1.74987157686527

H	8.18514377519126	16.62315778391558	2.82907929122438
H	7.99114473879150	16.47438057851033	1.07652191803456
C	6.80335852238929	13.59837882080172	-0.39193656023672
H	7.03226424086145	12.74790140014392	-1.04198428693647
H	6.20187145640473	14.31708363294030	-0.95624066027925
H	6.21444165813119	13.23661309711913	0.44757197592770
C	8.78956737741952	14.93159296330079	-0.92456812107644
H	9.70868122965265	15.31291978028777	-0.48775986654661
H	8.24211415672260	15.76547863117058	-1.37605825156102
H	9.05106947220578	14.22721526522376	-1.72434089836769
H	5.91685420265296	10.55264460929490	3.36022159569693
H	6.77249574536749	12.00581230608240	3.31677782707666

13.3.3 2-((1,3-dimethylimidazolidin-2-ylidene)amino)-4,5-difluoroaniline

Illustration of the molecular structure. C-H hydrogen atoms are omitted. Colour code: C dark-grey, H light-grey, N blue, F pale green.

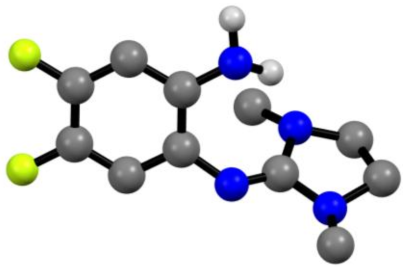

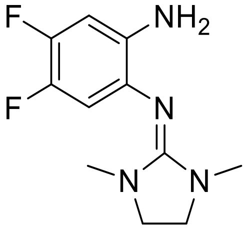

E_{el} = −846.20182819

Cartesian coordinates (in Å)

F	2.38937925088772	3.36529417362136	3.70709156553434
F	2.45312570233735	0.94499831711603	4.96972074519978
N	3.90787004269820	2.99888081055540	9.01318638260046
N	6.12961246620997	3.98288526407081	9.12351048091546
N	5.03915907840109	3.38978637421474	11.00448183675144
N	3.90012849384960	5.55465527727827	7.68417040922386
C	3.62117267802382	3.12616255571008	7.65720045438659
C	3.58510905497773	4.37885061046855	6.99676064819150
C	3.16759821995633	4.43218167921642	5.66534005884233
H	3.13629133504758	5.37633528919495	5.13576271083479
C	2.78702871408449	3.28724656345145	4.99286551740883
C	2.81965164599229	2.06128004984476	5.63427884099959
C	3.22178056719710	1.98669296757631	6.95243125522089
H	3.22256508603120	1.02941231016875	7.45487273573280
C	4.94457261435995	3.41888266460286	9.62419174884855
C	7.07359136390351	4.14070967113054	10.23185853818756
H	7.68604745913791	5.03292632652656	10.10232756629558
H	7.73590809636621	3.26745053812375	10.29543611568712
C	6.15261572630238	4.21000821813629	11.44198301766153
H	6.61165906940705	3.81329375982282	12.34810491175969

H	5.83141438066471	5.24314699521226	11.64097712958143
C	6.71038992154214	3.48797730265518	7.88281402832767
H	7.11087807618970	2.47398189672670	8.00525596908532
H	7.52482329699551	4.15134748042704	7.59119552064024
H	5.97288710303145	3.47629643329688	7.08707459831691
C	3.84602875903373	3.31560650007601	11.81703904679714
H	3.37678056272694	4.30071866714866	11.94014278308353
H	4.10139774056915	2.92114601356135	12.80130123187840
H	3.13445407638221	2.65287714424066	11.33247808835576
H	3.98765745195008	6.36232604402344	7.08699573491634
H	4.68484196574270	5.47591210180096	8.31665032873423

13.3.4 4,5-dichloro-2-((1,3-dimethylimidazolidin-2-ylidene)amino)aniline

Illustration of the molecular structure. C-H hydrogen atoms are omitted. Colour code: C dark-grey, H light-grey, N blue, Cl green.

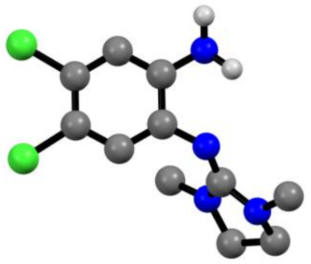

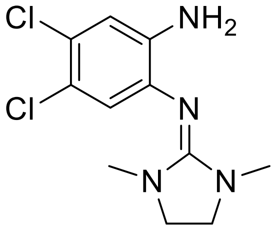

E_{el} = −1566.84118593

Cartesian coordinates (in Å)

Cl	4.11644224563922	5.10618687980815	3.96626942668206
Cl	2.88226491053042	7.41417480070705	5.83845959142298
N	3.15368614617036	4.05505666591586	9.72334009872690
N	0.75690870660636	4.31085359824746	9.97077627554322
N	2.06755755725715	4.33943314415347	11.76507687059731
N	4.09626236017934	2.06692664300696	8.09613759528584
C	3.30486566245963	4.32544939481176	8.36767757544566
C	3.86508161357118	3.32107513198451	7.54437490936745
C	4.10039589060322	3.59307642140003	6.20149482604131
H	4.51218580571583	2.82691602332585	5.55784042699321
C	3.80175151384781	4.83821472244087	5.65622113931619
C	3.26819052638342	5.83508028163626	6.46393911920634
C	3.03778556467684	5.57279530629340	7.81031838105761
H	2.65170703723200	6.36177231659594	8.44095466332955
C	2.06570522509971	4.23322630229015	10.38469617139296
C	-0.11623527424580	4.64336595697287	11.08443410446374
H	-1.06566160242532	4.11275919167970	11.00483218613831
H	-0.32281573091649	5.72073653723623	11.12605711623576
C	0.71941132754768	4.19003392641268	12.28129870669782
H	0.56384443796235	4.80661005998726	13.16685551604117

H	0.50424167220234	3.14344273616385	12.54304547487692
C	0.26503067405814	4.27905361776158	8.61408123874861
H	0.22768877410687	5.27098350061111	8.15134787410015
H	-0.74255839280938	3.85966889498705	8.62381338190315
H	0.89581344099163	3.64245657960095	8.00050026177298
C	3.20212745257180	3.91497332755662	12.54680843965104
H	3.18554632027325	2.83388568402179	12.74274723432758
H	3.20523601185365	4.44159421609405	13.50233079813184
H	4.11023733499955	4.15368746399665	11.99895388036621
H	4.82555993603129	1.52640600286453	7.65870699062286
H	4.16099285182584	2.09514467143517	9.10463972551286

13.3.5 2-((1,3-dimethylimidazolidin-2-ylidene)amino)-4,5-dimethylaniline

Illustration of the molecular structure. C-H hydrogen atoms are omitted. Colour code: C dark-grey, H light-grey, N blue.

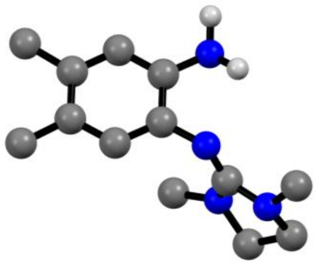

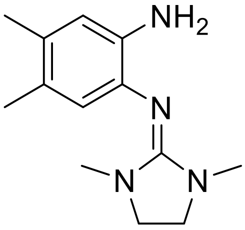

E_{el} = −726.32161231

Cartesian coordinates (in Å)

N	3.52879513100475	4.57297070845146	9.93999551377273
N	2.37548939497388	4.69674206750785	11.95556542253170
N	1.10751398432761	4.44996300516676	10.13623111166449
N	4.00069019265282	2.25660390951271	8.49201494140646
C	3.66157532006535	4.64742480260215	8.54661571848419
C	3.97541126212603	3.48411084985216	7.82353250323997
C	4.17847260242762	3.57983681829214	6.44815564690984
H	4.40438292618222	2.67634487923036	5.89093361054456
C	4.09372190277069	4.79164278460263	5.76748772544070
C	3.79985949914216	5.95838987921997	6.48863354914644
C	3.59866098298629	5.85975456813514	7.86290895494458
H	3.38594538623415	6.75322503626532	8.43865937231376
C	2.41580204102395	4.57959370208919	10.56995374646318
C	1.05516193034825	4.35011972707651	12.44442380158187
H	0.79673104064496	4.92371261557934	13.33505079423107
H	0.98373158531983	3.27898136197160	12.68672407721843
C	0.19025662804254	4.69567996311638	11.23763352316519
H	-0.69712707097536	4.06747333450835	11.15291152427173
H	-0.12912300428613	5.74709396768932	11.26631046733629
C	3.53945742301811	4.39059923684722	12.74968274803740

H	3.65567967382884	3.30965418374015	12.91305994211741
H	3.45724752241048	4.88147835708313	13.72050308914333
H	4.42120782413562	4.75704497871176	12.23060982457629
C	0.63822044222688	4.62783800905452	8.77977368506126
H	0.55567271412454	5.68451713283659	8.49864304842006
H	-0.34856892316145	4.16918272222781	8.69922486441417
H	1.30339440258482	4.13745224775609	8.07757542182930
C	4.32600356611394	4.84018602879596	4.28132032533882
H	3.45560770274220	5.23389751489944	3.74863599312445
H	4.53814578645617	3.84713556713517	3.88517121556780
H	5.16859521617576	5.48922974383841	4.02604255104695
C	3.71201005960699	7.29484095890526	5.80156042746424
H	2.93103302214721	7.30834894586490	5.03535102336336
H	4.64787910945394	7.55522622946547	5.29842338660504
H	3.48921367572237	8.08587930860407	6.51760773991347
H	4.60247095861068	1.56423801126936	8.07332126359915
H	4.16722808879115	2.36594684209518	9.48325144570993

13.3.6 2-((1,3-dimethylimidazolidin-2-ylidene)amino)-4,5-dimethoxyaniline

Illustration of the molecular structure. C-H hydrogen atoms are omitted. Colour code: C dark-grey, H light-grey, N blue, O red.

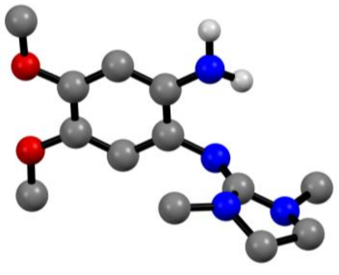

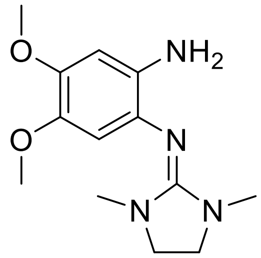

$E_{el} = -876.73246779$

Cartesian coordinates (in Å)

O	5.16713838884391	2.10416448212338	1.21455979173720
O	4.98932707084431	4.41675713980561	0.00935057391656
N	4.63654021522162	2.23067429422761	-4.33326724155741
N	2.24320655589979	1.87083882640692	-4.59353160296278
N	3.53867112726936	2.22806996839935	-6.38234326298386
N	4.74948357175504	-0.25334381828451	-3.03073382235202
C	4.72348676954262	2.16774451707457	-2.93244129491381
C	4.84796993116377	0.93506168306950	-2.28775070876839
C	4.99139901602252	0.90153599687742	-0.89584749978078
H	5.06689614041420	-0.06041070676265	-0.40806048991844
C	5.02991039726465	2.06601082015446	-0.14245166792946
C	4.93274907651829	3.31098017997576	-0.79213271977546
C	4.79113449518225	3.34025386273043	-2.17058568640203
H	4.72388040132400	4.27979679474730	-2.69850014294640
C	3.54731558177257	2.11985539635733	-4.99485983723266
C	1.34323151687779	2.02022307645440	-5.72844323925691
H	0.92977452686554	3.03782991473027	-5.77427105649934
H	0.51542736206406	1.31283888759973	-5.66611215079272
C	2.27218494249312	1.75261913564304	-6.90525293534649
H	2.31260323995659	0.67784875081925	-7.13870189201227

H	1.98560339648590	2.29173645161262	-7.80899360794459
C	1.71468365440979	2.16109446504459	-3.27605601350300
H	2.36769753542981	1.77139531855784	-2.50330456102174
H	0.74318626971281	1.67320656600440	-3.18576757440273
H	1.57987124826800	3.23769137578153	-3.11243791142835
C	4.74761861719218	2.00308094434162	-7.13689939697819
H	5.58269778834981	2.44187157730742	-6.59737089431073
H	4.65782265782683	2.47388916555398	-8.11703266800254
H	4.94957477528213	0.93166736535482	-7.28100891797167
C	5.23931869311072	0.87595194195918	1.90695069823021
H	6.11519573493357	0.29213605456528	1.60358454971111
H	4.33819625226152	0.27297801204424	1.75134268194986
H	5.32500343635714	1.12922524398719	2.96158679431528
C	4.92254646188226	5.68098204702991	-0.61510487241259
H	5.75138202135433	5.82901057924254	-1.31566702167691
H	4.99027050035466	6.41637905185437	0.18397526240073
H	3.97702136315790	5.81631279125356	-1.15190958850462
H	4.99650340763603	-0.10672032545057	-4.00078797910305
H	5.27100585869831	-1.02229782819384	-2.63575209356879

13.4 Reorganized structures of primary amines

13.5 *N*[1]-(1*H*-benzo[*d*]imidazol-2-yl)-*N*[1],*N*[2]-dimethylethane-1,2-diamine

Illustration of the molecular structure. C-H hydrogen atoms are omitted. Colour code: C dark-grey, H light-grey, N blue.

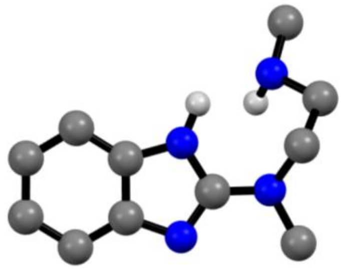

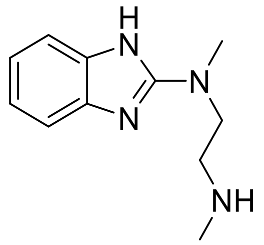

E_{el} = −647.71367370

Cartesian coordinates (in Å)

C	-1.93873572640138	0.89920413649933	-1.34093046113843
C	-1.99359367799481	-0.11529193203962	-0.35874807550401
C	-3.18821622182049	-0.58839921635155	0.15858410815865
C	-4.36035191083583	-0.01589734302547	-0.33077773953445
C	-4.32419753762177	0.98414658258589	-1.30819765692610
C	-3.12009669534212	1.45192881529733	-1.82486845061423
H	-3.21641207443057	-1.36792868195299	0.90939591353538
H	-5.31514903626411	-0.35414597372671	0.05027207104223
H	-5.25532474732194	1.40233417453262	-1.66885294009917
H	-3.09275331610873	2.22530596523580	-2.58115811785136
N	-0.61777480913199	1.17320626347026	-1.67411096427310
C	0.09698167465150	0.37315973526088	-0.91880895657776
N	1.46580565351408	0.34232408713315	-0.86017365143793
C	2.19152605555166	1.25390924565741	-1.73005143812202
H	2.28826616822126	0.85833422430467	-2.74812296260438
H	3.18712076796248	1.42221515375308	-1.31827444362428
H	1.65767402348135	2.19855863197215	-1.78378871857658

C	2.13550115489841	-0.90275867384247	-0.49012259794983
H	2.97621876181772	-1.05752161549556	-1.17064383252050
H	1.44740689594230	-1.73817153329865	-0.63500553112950
C	2.64148374291988	-0.91330209930634	0.94805862556206
H	3.05357703473644	-1.90365752431644	1.16153915154704
H	3.46507705561124	-0.19106583129981	1.06483770505943
N	1.55139395071177	-0.65885191725232	1.88566441290804
C	1.92658408672512	-0.86974860789110	3.27967956472234
H	2.81685476983246	-0.29970407449947	3.58408036516692
H	2.13390937313125	-1.92868070862081	3.44481490201333
H	1.09726747760517	-0.58423300315533	3.92723863574640
N	-0.66728238786365	-0.45604719989499	-0.12129374764661
H	1.25293946094884	0.30360984341702	1.76446562326219
H	-0.29569996712555	-0.94283092314993	0.68529920740624

13.5.1 *N,N*-dimethyl-1*H*-benzo[*d*]imidazol-2-amine

Illustration of the molecular structure. C-H hydrogen atoms are omitted. Colour code: C dark-grey, H light-grey, N blue.

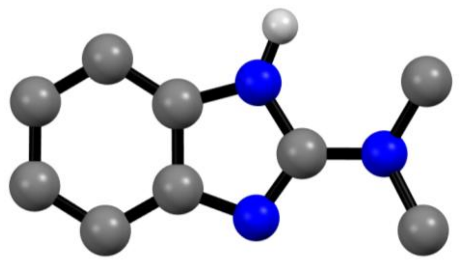

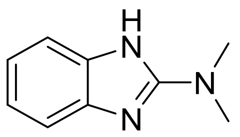

E_{el} = −513.76299039

Cartesian coordinates (in Å)

N	4.55379440122637	4.22590431962880	5.28504669689467
N	4.22990369206599	5.49913488943078	7.10361152708084
N	5.46539263807507	3.45139428406585	7.34277928089169
C	3.86164342882194	5.37163659915750	4.92164873850932
C	3.64388439147774	6.19128586832095	6.05030311681305
C	2.96957792060656	7.39865114190441	5.98198556515897
H	2.80905258515506	8.01628718152868	6.85685110755570
C	2.50402147085262	7.79054744637788	4.72873603743642
H	1.97245442701976	8.72787065298905	4.62949952330107
C	2.71234739838090	6.99310940577123	3.59838925880664
H	2.33732764099949	7.32947152285187	2.64026615383712
C	3.38875940000655	5.78034532306744	3.67830454082637
H	3.54920187796873	5.16477954407201	2.80310837177711
C	4.76627569847779	4.33989706815043	6.57554956264585
C	5.73952422132227	2.14745264100057	6.75973308000949
H	5.93395458353910	2.26930025009176	5.69834005250548
H	4.89396424369413	1.45822000514847	6.87817875261606
H	6.61614803432838	1.71813063364267	7.24565437499118
C	5.35355620963545	3.51429920990680	8.78501048743485
H	5.59542269672615	4.51389029943712	9.15167187605950
H	6.07986266850250	2.83011640690927	9.22034852156930

H	4.35583317943058	3.23517127802588	9.15155201805771
H	4.27242719168675	5.80117402852044	8.05885135522144

13.6 *N*[1]-(5,6-difluoro-1*H*-benzo[*d*]imidazol-2-yl)-*N*[1],*N*[2]-dimethylethane-1,2-diamine

Illustration of the molecular structure. C-H hydrogen atoms are omitted. Colour code: C dark-grey, H light-grey, N blue, F pale green.

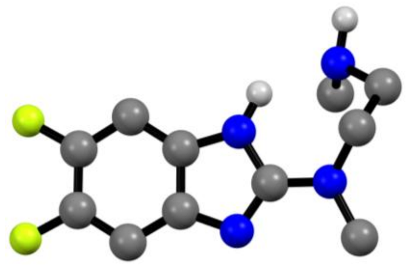

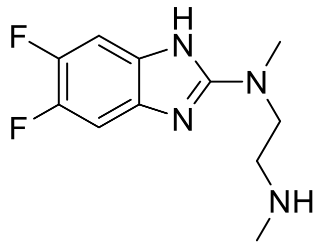

E_{el} = −846.20762760

Cartesian coordinates (in Å)

N	12.15478496709536	-0.43960342823237	10.25276685105452
H	12.22096825547566	-1.44760777557280	10.28092553432478
N	11.97681702901273	2.37769188287305	9.09935340236352
N	9.93879483005589	1.41735895296054	9.88562405223048
H	10.34922546941299	0.49168838697334	9.99629024112194
N	10.10588947565024	3.66432431347548	9.83572271652949
C	13.00620883954617	0.08374331224319	9.17513241031638
H	13.25632137905654	-0.73397205954989	8.49396894949804
H	13.95078645626554	0.47053155294675	9.57877004230560
C	12.32006062443004	1.17201308601096	8.34997743010367
H	12.98492673224040	1.46124950044499	7.53258806084365
H	11.41587941333392	0.75751775770112	7.89576978620509
C	12.45689765754928	0.10468402233813	11.57822834731846
H	13.50713312646661	-0.03469670894539	11.86831673582601
H	11.82270074878448	-0.37968229247734	12.32124030902870
H	12.24169217951649	1.17233772331412	11.59505996342372
C	10.70629924546823	2.52320178026894	9.58199351397868
C	8.75992428567333	1.90095402003346	10.43208941823974
C	7.63553271707286	1.26597461473221	10.93375340552059
H	7.52518088501465	0.19145819566920	10.98213196072213

C	6.61603592800485	2.08644287447645	11.38911043130905
F	5.49816751548425	1.53213855763095	11.90156928766569
C	6.71278302981145	3.47652697380160	11.33022911833424
F	5.67320109439912	4.21073867699752	11.77811011944727
C	7.83562384937658	4.11044081276434	10.82895839173235
H	7.88431477955642	5.18980119484707	10.79638720645292
C	8.88034278633205	3.30855604961182	10.37861944153842
C	12.72540451173614	3.58874112849356	8.80450779099318
H	12.47069621925885	4.35167123379127	9.53479602275102
H	12.49449166559511	3.98075932543910	7.80684557023111
H	13.79272430332323	3.37287633493853	8.86315348858902

13.6.1 N^1-(5,6-dichloro-1*H*-benzo[*d*]imidazol-2-yl)-N^1,N^2-dimethylethane-1,2-diamine

Illustration of the molecular structure. C-H hydrogen atoms are omitted. Colour code: C dark-grey, H light-grey, N blue, Cl green.

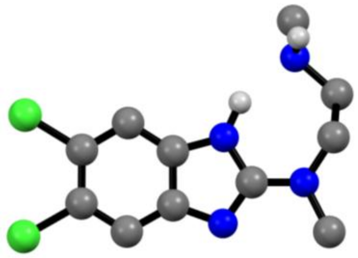

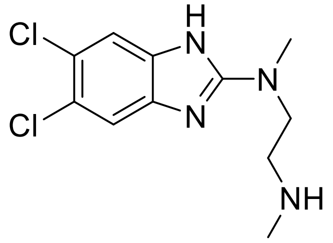

E_{el} = −1566.84460301

Cartesian coordinates (in Å)

N	12.07039919285350	-0.40639118862495	10.32405016414063
H	11.78631353741312	-1.09499628141223	9.63491186685053
N	11.98787547082877	2.63329003224485	9.00266847633834
N	10.12367570622560	1.65735312096103	10.14699967045238
H	10.61600614609876	0.78748163832951	10.37940068495277
N	9.96610613230706	3.80185504197472	9.47797049382642
C	13.08645655398409	0.46648277080193	9.72948480472797
H	13.93409316413796	-0.11900786680177	9.34427240390060
H	13.47665169301701	1.11769948435551	10.51404271877855
C	12.53094033766243	1.33947038106803	8.59613792704909
H	13.34734153363310	1.55439481405000	7.90871394042903
H	11.78600925397143	0.76731390136839	8.02617915596466
C	12.52629835785032	-1.08883447864511	11.53233638680476
H	13.45524397266099	-1.65684714567719	11.38482377103404
H	11.75177109858587	-1.77335759942271	11.87813508300863
H	12.70132198901086	-0.35192373602200	12.31780631744687
C	10.71979375335252	2.72205310867564	9.51075357264031
C	8.89091387155028	2.10448152953935	10.58667778756777
C	7.86898853703378	1.48881605470061	11.28391489516297
H	7.92403097290976	0.46208559047002	11.61677293600629

C	6.73307521753862	2.24831021790904	11.55680888074760
Cl	5.43587221039072	1.49420569395187	12.43895675700459
C	6.63585528214182	3.58056653943262	11.12979593148371
Cl	5.20581764585191	4.51443772096412	11.47064629168505
C	7.67000995789708	4.19079782099641	10.42723838770285
H	7.57894689257759	5.21805212246459	10.10534516240287
C	8.81124116340552	3.44861912622925	10.15392385163479
C	12.47931717777931	3.80265115299281	8.28404904712231
H	12.13415169232969	4.69996294427567	8.78896832749194
H	12.11610363411284	3.83529689798438	7.25005328994013
H	13.56918785088698	3.78354059086552	8.28015101570089

13.6.2 *N*[1]-(5,6-dimethyl-1*H*-benzo[*d*]imidazol-2-yl)-*N*[1],*N*[2]-dimethylethane-1,2-diamine

Illustration of the molecular structure. C-H hydrogen atoms are omitted. Colour code: C dark-grey, H light-grey, N blue.

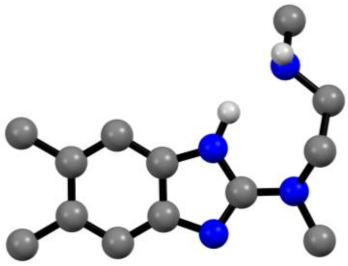

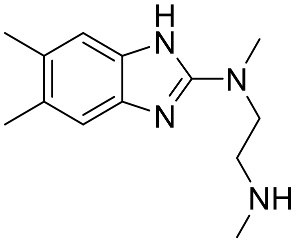

E_{el} = −726.32590878

Cartesian coordinates (in Å)

N	12.05916696016521	-0.44256172739074	10.25348237408887
H	11.70964444903747	-1.07838862390287	9.54395609326893
N	12.07024923219341	2.63684458764181	9.07343131769398
N	10.13660794784401	1.68899004361908	10.12702731505121
H	10.60057554930263	0.80950496569566	10.36238792487255
N	10.06549881164239	3.85358665341910	9.51851310599307
C	13.10069533997077	0.39934696831449	9.66060141875146
H	13.90334390758167	-0.21241167729559	9.22156739207105
H	13.54894186682046	0.99643208029783	10.45717773904049
C	12.55647063182668	1.34928919694832	8.58608853724135
H	13.36764403547588	1.56735025425025	7.89256383001503
H	11.77916020499098	0.83423673246793	8.00510007395072
C	12.51931494341238	-1.20892395611001	11.40741965167555
H	13.40702816615375	-1.82223241497796	11.19685005541371
H	11.71931173618676	-1.86380482481145	11.75306577839510
H	12.76797821531924	-0.52283965136134	12.21873026726257
C	10.78304914121852	2.75195426860222	9.54180140188408
C	8.90254337829500	2.16361129385620	10.54670082399865
C	7.83572417429784	1.56685573324380	11.19802070274329
H	7.86630108433714	0.52536134645314	11.49608118054490

C	6.70729651761354	2.34045045017778	11.47101619089951
C	5.53658883775562	1.71839682785504	12.18548008842788
C	6.66349180999278	3.69844995451313	11.07903488281219
C	5.44050685048429	4.52761932840187	11.37342679924771
C	7.74585506087243	4.27958299998666	10.42176072276670
H	7.71106516986604	5.31934731439997	10.12048296566869
C	8.87670058605073	3.51605698238126	10.15338697217297
C	12.62712829226266	3.82371014540538	8.43930310191379
H	12.32054442186024	4.70183162070350	9.00023933320587
H	12.27852258042850	3.94727685697397	7.40647276062481
H	13.71498422546160	3.75123828430100	8.44313053726586
H	5.73404579173193	0.67182388661551	12.41893468443310
H	4.62436446682653	1.75978254117769	11.58306218411821
H	5.31465593247125	2.23334550639692	13.12480034823485
H	5.56610412076952	5.54640490041893	11.00717382809307
H	5.23512927909885	4.58080938544659	12.44668118523424
H	4.54497628038023	4.11118176588470	10.90296643092281

13.6.3 *N*^{1}-(5,6-dimethoxy-1*H*-benzo[*d*]imidazol-2-yl)-*N*^{1},*N*^{2}-dimethylethane-1,2-diamine

Illustration of the molecular structure. C-H hydrogen atoms are omitted. Colour code: C dark-grey, H light-grey, N blue, O red.

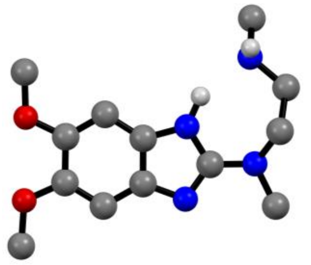

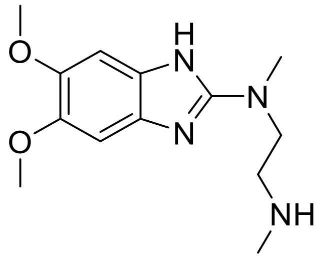

E_{el} = −876.73693803

Cartesian coordinates (in Å)

N	12.00256940522516	-0.49380084129494	10.11815506402067
H	11.63043923342413	-1.06722263961333	9.36800962526335
N	12.11859037960464	2.65527475839867	9.18678115569571
N	10.12375525671720	1.69321871806127	10.10582127155890
H	10.55916807730529	0.78908734717246	10.29442568477782
N	10.13661214196799	3.89960859348703	9.67530875604025
C	13.07489606381719	0.34704380094211	9.58015641235386
H	13.84759063054382	-0.26299036156834	9.08779585294502
H	13.55436613719554	0.86077482628195	10.41577430298519
C	12.56453703301189	1.40003852824655	8.58918868737744
H	13.38311721401272	1.64809429655798	7.91417592164089
H	11.77085049529465	0.96149745105954	7.96920650131313
C	12.43635534598546	-1.35329130141343	11.21516431559289
H	13.30258128803165	-1.98086119547112	10.96112580071378
H	11.61484700759070	-2.00340275278144	11.51694215958139
H	12.70854879177625	-0.73400569343631	12.07140316260035
C	10.81975686701603	2.77737321564476	9.62663070392167
C	8.89454065830708	2.17669609284193	10.53330304892497
C	7.79123689072633	1.55356286881055	11.10782231603826
H	7.78992330365315	0.49376445059676	11.31378790271675

C	6.69054780625986	2.34702965143061	11.41048871540688
O	5.54282916500276	1.87649418873845	11.98260223853981
C	6.70088186888769	3.73908267742317	11.12984564589198
O	5.55701608618350	4.40335778007401	11.47164703993321
C	7.80980248684630	4.34027808562910	10.55142241531345
H	7.82993387162147	5.39670525320516	10.33115713610761
C	8.92170791243089	3.54952929724649	10.25243260217021
C	12.73581191908683	3.87127284006165	8.67739080174025
H	12.43680690506657	4.70805322980293	9.30214581489842
H	12.42993040332344	4.09531047967373	7.64768652644780
H	13.82014776902097	3.76170018094835	8.71265033991240
C	5.46662383190941	0.49715044098734	12.26672140808160
C	5.50786311920721	5.79331155395595	11.22787725377681
H	4.48469569278983	0.33235585185202	12.70550253553175
H	6.23792799756051	0.18922427526369	12.98168706158886
H	5.56276578187435	-0.10756670294625	11.35790770073254
H	4.52971710006825	6.12458396382927	11.57059092209386
H	5.61388899066710	6.01959714552898	10.16147248042381
H	6.28679907098520	6.32717964477245	11.78271271534553

13.7 Secondary Amines and reorganized side products

13.7.1 1,1,3,3-tetramethyl-2-(2-(phenylamino)phenyl)guanidine

Illustration of the molecular structure. C-H hydrogen atoms are omitted. Colour code: C dark-grey, H light-grey, N blue.

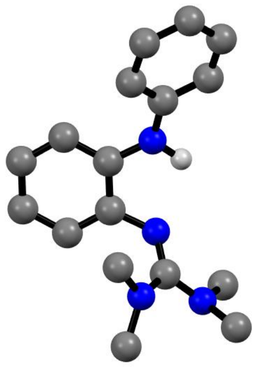

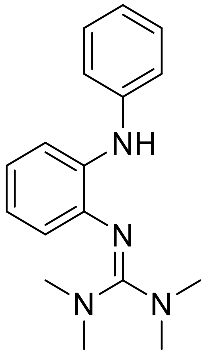

E_{el} = −879.90995204

Cartesian coordinates (in Å)

N	4.44392846421268	10.89271167722821	14.08220037875239
N	6.54591085011331	11.11281561218747	15.00136094614261
N	6.30631438309644	11.45921182357507	12.70930431177057
N	2.66404562880350	9.12398524079087	13.14595343465132
H	3.29000018115382	9.04086695722553	13.93458247588124
C	3.46315738709893	11.36277467437816	13.20582107123336
C	2.49711519058697	10.44019212087177	12.73649029220197
C	1.44175960990233	10.89805982234068	11.94764911832361
H	0.67641281418609	10.20797453766163	11.62526699497846
C	1.35107570737559	12.24091326952211	11.59672138158239
H	0.52383613209733	12.57457123481057	10.98354147680041
C	2.30106785447301	13.14851602217807	12.04580709517164
H	2.22686884424172	14.19598497969764	11.78366851172252
C	3.34108931189742	12.70593513908852	12.85653984620610
H	4.07289210398151	13.40713842434946	13.23927879033833
C	5.69738460070428	11.15475094195592	13.91410455556414
C	5.95992202852346	11.07171784402536	16.32804629242539
C	7.25506366779299	12.54908134622051	12.59063137427725

C	7.85552977153581	10.48960310556752	14.90398526021700
H	7.83474004583579	9.47780134646029	15.32831351128891
H	8.59954468904224	11.07185835722934	15.45316967634963
H	8.16247872211035	10.41641105501667	13.86549684942486
C	5.76021299578495	10.97293096146077	11.45739853879918
H	5.17014602232784	10.07715293178087	11.63376657907435
H	6.58876591840991	10.71762826608304	10.79066536415123
H	5.12462099335784	11.71158377298851	10.95907988453037
C	2.15921914766448	7.94600433077994	12.61787306737828
C	1.73375804963001	7.81653188803387	11.28870434877952
H	1.78703808229548	8.66253970711956	10.62034434520651
C	1.28162344677995	6.59236189226789	10.81474635799837
H	0.96297088701774	6.51550090867596	9.78236079689666
C	1.24665934947480	5.47148516282239	11.63592887239631
H	0.89103149074017	4.52251593433911	11.25751007236038
C	1.68200086716362	5.59154802964393	12.95245671684023
H	1.66695398481616	4.73099994516279	13.60987786189932
C	2.12590201601053	6.80942908719443	13.44041838079137
H	2.44713552820687	6.89823362480794	14.47170204424382
H	6.81173277588907	13.38354782244125	12.03298806006924
H	8.15804217421463	12.23160239060310	12.05915996806709
H	5.06195271107215	11.68206904439387	16.35151800705177
H	7.53803927019148	12.90628882060723	13.57707705680925
H	6.68796440306947	11.45878367706318	17.04424405190340
H	5.68559189711624	10.05245626934641	16.62556597944577

13.7.2 *N,N*-dimethyl-1-phenyl-1*H*-benzo[*d*]imidazol-2-amine

Illustration of the molecular structure. C-H hydrogen atoms are omitted. Colour code: C dark-grey, H light-grey, N blue.

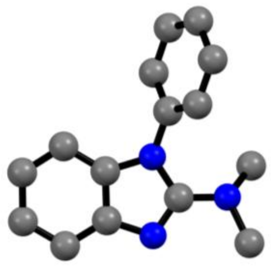

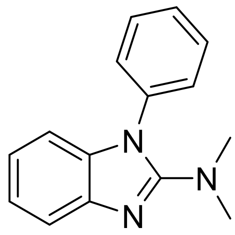

E_{el} = −744.77081236

Cartesian coordinates (in Å)

N	4.53816889415821	4.23652469537951	5.39746158882288
N	3.83795518627754	5.41698898573345	7.18566035354232
N	4.88187560853661	3.25770936611779	7.54454930944439
C	3.94537541779288	5.41064235822377	4.95802308505124
C	3.50942123425410	6.17545989182743	6.05848862586126
C	2.86567866505408	7.39415552723293	5.91065552144892
H	2.51650949503755	7.96265033889050	6.76175044615811
C	2.67591664992507	7.85210090289536	4.60962674395434
H	2.17655389080302	8.79909122482155	4.45087841520574
C	3.11638410336697	7.11242198643437	3.50630980372402
H	2.95459620072873	7.50353915718563	2.50998607880359
C	3.75111344443008	5.88608028693078	3.66488435223641
H	4.08518359154834	5.30660749238464	2.81437507783219
C	4.45463734505405	4.26154132366018	6.70306271178464
C	5.27046669845201	2.02326596012491	6.87383989818279
H	4.51333521280264	1.74918960502801	6.14400128859938
H	5.35408353755985	1.23795197291712	7.62597884181355
H	6.22835202038870	2.12019539965216	6.34897488131741
C	5.77544098397814	3.62027918093714	8.64231883761865
H	5.44030108927719	4.52797338754295	9.13390242461463
H	6.80391779944514	3.77076089412371	8.28705099512473

H	5.77567730742718	2.81608122299135	9.37854823107044
C	3.38831813625683	5.68536375209347	8.50303071639167
C	2.60198510679461	4.75606237727447	9.17959412056483
H	2.34721412181139	3.82433881855659	8.69478964031729
C	2.17146840506042	5.03004809070563	10.47050340530469
H	1.56416323078712	4.30424658858903	10.99590543123788
C	2.50372247361069	6.23438712533873	11.08257671708618
H	2.16065735647171	6.44602918829227	12.08689973647188
C	3.27809557848990	7.16470775833251	10.39895219277719
H	3.54344418553926	8.10258469572045	10.86966818188641
C	3.72679904740444	6.89062998996477	9.11306145778974
H	4.34875798147540	7.59763045409663	8.58072088796034

13.7.3 dimethylamine

Illustration of the molecular structure. C-H hydrogen atoms are omitted. Colour code: C dark-grey, H light-grey, N blue.

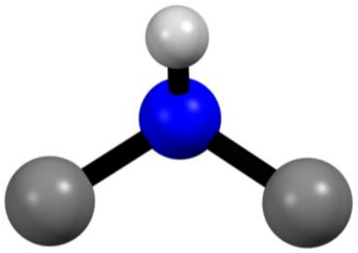

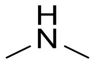

E_{el} = −135.13551897

Cartesian coordinates (in Å)

C	-9.57675404090447	2.97050598391433	-0.01461195012786
N	-8.33397857570937	3.70850526931440	-0.16871737562141
H	-10.42521197652614	3.63957289298734	-0.16480139550324
H	-9.68785889811128	2.48342868221446	0.96885922054699
H	-9.63098957122400	2.18810448084860	-0.77590413016040
C	-7.15522439297855	2.90039320617503	0.09725025451022
H	-7.16224967674822	2.41209395830959	1.08635798012761
H	-6.25909865550645	3.51832350486870	0.02483226676722
H	-7.07785532388760	2.11259350725043	-0.65652679061981
H	-8.33830888840379	4.52031851411709	0.43609192008067

13.7.4 2-((1,3-dimethylimidazolidin-2-ylidene)amino)-*N*-phenylaniline

Illustration of the molecular structure. C-H hydrogen atoms are omitted. Colour code: C dark-grey, H light-grey, N blue.

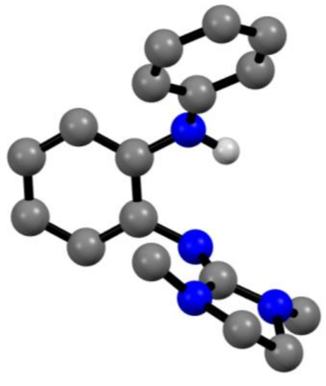

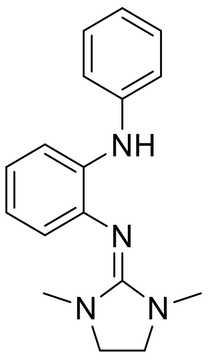

E_{el} = −878.71831343

Cartesian coordinates (in Å)

N	3.93320354189711	11.24583066274343	14.45536326131620
N	6.13059508902051	10.79596653643048	15.05986080582034
N	5.79194703675926	11.24581276870180	12.89074856138365
N	2.50822888518671	9.20727224226486	13.31134283291627
H	3.07658936355666	9.14836643356039	14.14349796358436
C	2.88925235888542	11.56054141770104	13.58206714837885
C	2.12528229282903	10.51780483968621	13.01311307018256
C	1.01783813663625	10.82598457724127	12.22557584388999
H	0.41278743377538	10.02393913218921	11.82594306561823
C	0.68106523930741	12.14916184096467	11.96563957589262
H	-0.17872849370273	12.37042348926247	11.34657730205395
C	1.43148343107335	13.17895792318975	12.51996342408706
H	1.16844792299521	14.21142683732306	12.32890406650386
C	2.51699210500182	12.88070284701336	13.33580581398183
H	3.10343455762250	13.67064187500027	13.78775247375972
C	5.16113360238990	11.11228109056652	14.11931287966470
C	7.45118400811851	11.00412655589909	14.49485972030941
H	8.17420698126990	10.29297937609332	14.89575736318353

H	7.81421070408748	12.02230298894187	14.69836810679941
C	7.17775260306217	10.80392074843328	13.01069325230980
H	7.83320490049678	11.39347361978194	12.36906685857460
H	7.27400294917231	9.74572937570832	12.73128692563638
C	5.90843371902684	11.06374476047309	16.46171383129191
H	6.12541006707304	12.10990943801410	16.71758519396563
H	6.55071426609364	10.41721763053730	17.06156591351984
H	4.86726244590783	10.86055894555235	16.69730118213743
C	5.13613052036830	10.99499948679079	11.61878066513265
H	4.90457329578773	9.93297496052100	11.47607961887372
H	5.81019795495431	11.31316975124759	10.82290261132678
H	4.21870167227024	11.56754350366339	11.53812457307019
C	2.62157393387294	8.13010256981749	12.44226249323873
C	2.41171857184198	8.22582386576150	11.06040192422150
H	2.14335788238318	9.17400204094194	10.61969791120205
C	2.57061086358266	7.10939738803468	10.24923645762158
H	2.40597414510410	7.20971728220686	9.18324023614230
C	2.94163090963964	5.88025702046501	10.78005576941831
H	3.05816868902014	5.01551735763204	10.14067846859959
C	3.15861225951830	5.78270597360974	12.15202011699626
H	3.44962888968757	4.83550974952547	12.58935821732696
C	2.99864669343732	6.88624758269687	12.97275420911155
H	3.15810757098913	6.79715851381178	14.04126329095511

13.7.5 N^1,N^2-dimethyl-N^1-(1-phenyl-1*H*-benzo[*d*]imidazol-2-yl)ethane-1,2-diamine

Illustration of the molecular structure. C-H hydrogen atoms are omitted. Colour code: C dark-grey, H light-grey, N blue.

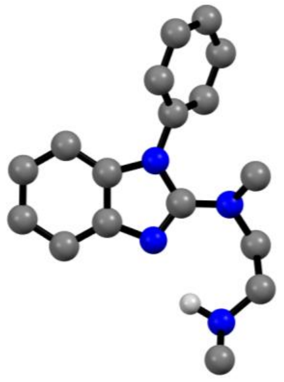

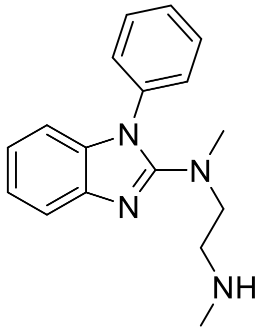

E_{el} = −878.71534667

Cartesian coordinates (in Å)

N	6.75309398389607	5.58533891356553	7.06892026143696
N	4.10927775586653	3.81359440146970	6.16663706637506
N	3.75606933585044	5.91614271354520	7.22023474593542
N	1.97809871323527	4.94940140388695	6.23052247345841
C	6.59092856213923	4.32575283260476	6.37228775790731
H	7.48459822324369	3.71373172128473	6.54267333647806
H	6.51486856873898	4.45038414368954	5.27569693414662
C	5.36571153809121	3.55193689217286	6.86996149875036
H	5.54813772422272	2.47930659990492	6.76773466141219
H	5.22033767267748	3.76715736713527	7.92853577784051
C	7.74082657099856	6.45565022523998	6.46457325394119
H	7.77444640351898	7.40320368126615	7.00403441807729
H	7.55089120733924	6.67382813661130	5.39893985474298
H	8.73397919228644	6.00055433331715	6.53071640061829
C	3.97257158786631	3.18731235377650	4.86040652890573
H	3.79112749056440	2.11344513494519	4.95732778631290
H	4.88385180859940	3.33426563382898	4.27349393462640
H	3.14672004371939	3.62345214348633	4.30779487316916

C	3.33951897929453	4.87663960593167	6.53440644946296
C	2.64403857804065	6.72093475908846	7.41523512635391
C	2.52002494603124	7.93163278140064	8.08853890882890
H	3.37801885184301	8.37925441832969	8.57235633791985
C	1.26743690639452	8.53501431391170	8.12507995293945
H	1.14634805306221	9.47769842234266	8.64295171929999
C	0.15749417008094	7.94601891875286	7.51098488262847
H	-0.80542365245787	8.43650470019556	7.56698978170295
C	0.26588030563981	6.73602879938381	6.82899593871097
H	-0.59490959528748	6.27591460855587	6.36350357804090
C	1.51944297267529	6.14884666724996	6.79002216301673
C	1.13030536084594	3.90461395226115	5.78060049735561
C	1.13137301313965	2.67182411970556	6.42801473957378
H	1.79203397888408	2.51519317297446	7.26942997139067
C	0.29126174091723	1.66010033819343	5.98318178908689
H	0.29534477067625	0.70218912673658	6.48705172112512
C	-0.56278240414134	1.87825663362853	4.90696198121305
H	-1.22077393955399	1.08917998230067	4.56745412951739
C	-0.56971563870121	3.11468401743674	4.27133536703646
H	-1.23060997881780	3.29110809901987	3.43266100049013
C	0.28027463302444	4.12598382489380	4.70093473672675
H	0.29795301230724	5.08506901170885	4.20089061584663
H	5.84894855324815	6.04210109426615	7.15598704759732

13.7.6 (Z)-2-((3-methylthiazolidin-2-ylidene)amino)-N-phenylaniline

Illustration of the molecular structure. C-H hydrogen atoms are omitted. Colour code: C dark-grey, H light-grey, N blue, S yellow.

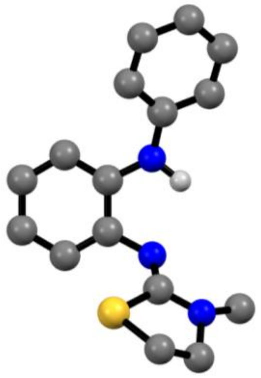

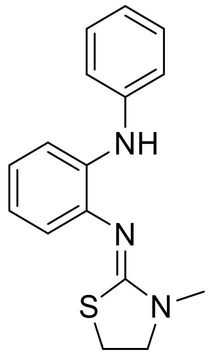

E_{el} = −1182.23598608

Cartesian coordinates (in Å)

N	4.14488396832528	10.97048704218253	14.23718946862275
N	6.35966816709571	11.00432201053057	14.95189315232798
S	5.95349549365307	12.52165047580820	12.86643932914285
N	2.75056094709666	9.04443990227155	12.99132511048117
H	3.37544622595167	8.96759688016321	13.77980079893007
C	3.11748720888075	11.36953725332901	13.37116204924302
C	2.36764061976110	10.35118719118556	12.73310849389615
C	1.27500214980584	10.71311904996300	11.94322174435590
H	0.66459491993810	9.94652049738382	11.49061844417692
C	0.94462349918563	12.05115948090169	11.76144955886866
H	0.09295770723925	12.30555886116189	11.14339576321213
C	1.68197572625064	13.05053944605258	12.38039815179133
H	1.41833877260398	14.09165885971476	12.24902679601341
C	2.75411188866614	12.70130620984765	13.19490462095265
H	3.31318695475122	13.46575127171724	13.71863372605701
C	5.33947514864937	11.39084644779009	14.11764658851674
C	7.56034056459445	11.81341663395669	14.85967930462288
H	8.42470836680399	11.22480456001827	15.17332458915785

H	7.49099894844130	12.69516202154030	15.51293194584956
C	7.67517776427687	12.23137894931155	13.40185559756529
H	8.25160563815921	13.14489922807876	13.27644751285597
H	8.11208480768297	11.43863126795479	12.79533173637581
C	6.05885475866199	10.35417418930692	16.21096218995106
H	5.83560414560501	11.08382419070534	16.99941442509867
H	6.91353198534646	9.75147232177989	16.51957982983106
H	5.19001655475156	9.71571026885594	16.07991883100658
C	2.46654378412741	7.87040182590941	12.30811862856790
C	2.10355764011654	7.83244424709868	10.95567404991323
H	2.03968767084939	8.74798809948277	10.38774508898869
C	1.86727020713531	6.61628782266416	10.32877243108063
H	1.59089462686410	6.61156042179613	9.28148775049432
C	1.99501036036326	5.41600826465284	11.01747087924056
H	1.80690591139877	4.47342544598713	10.52095265737421
C	2.37604754990119	5.44769182935838	12.35561094296801
H	2.48766958127116	4.52422837926732	12.91038484200026
C	2.60230238618010	6.65490059073410	12.99566320899421
H	2.87933734961440	6.67182856153689	14.04341976147394

13.7.7 2-(methyl(1-phenyl-1H-benzo[d]imidazol-2-yl)amino)ethane-1-thiol

Illustration of the molecular structure. C-H hydrogen atoms are omitted. Colour code: C dark-grey, H light-grey, N blue, S yellow.

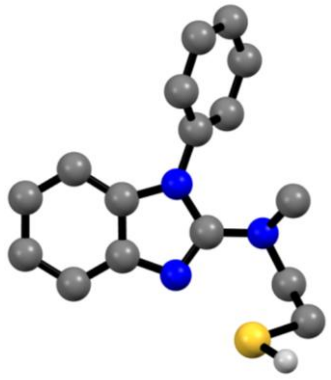

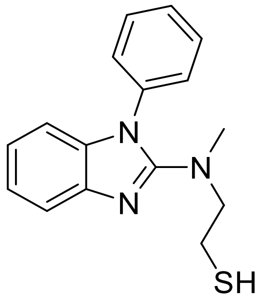

E_{el} = −1182.23252538

Cartesian coordinates (in Å)

S	6.44642545346712	5.95742837544027	6.06436322768780
N	4.08967334362558	3.78692662410385	6.49074049671962
N	3.62656372041239	5.81459929920617	7.64024158161501
N	1.96619409437680	4.96042378361302	6.37839377661565
C	6.59319779484500	4.18758056129905	6.53196418228028
H	7.43628041598863	4.05741627413741	7.21108870733506
H	6.79629770942732	3.61203606541679	5.62956728623016
C	5.34059944279338	3.65837742431505	7.23201401716865
H	5.50723158531657	2.59222583611314	7.42286630308687
H	5.20757524430571	4.15932608520884	8.18674841889962
C	4.12132158146899	3.28396225650470	5.12277935743605
H	4.54970683477149	2.28049096836509	5.13062504198966
H	4.72489839649996	3.92449090205067	4.47101494483289
H	3.12236918313492	3.21300136178175	4.70765001681329
C	3.28009722364901	4.84129772814044	6.83511326318459
C	2.51385398180066	6.63198735678909	7.75441690972183
C	2.32952379844431	7.80063758353764	8.48627044355794
H	3.13387035149866	8.19969931628277	9.08981054281007

C	1.08999371531857	8.42760015449586	8.41750602837404
H	0.92348880789729	9.33917790959414	8.97703868997436
C	0.04943495512082	7.90270806180987	7.64385446267922
H	-0.90587408519957	8.41061703889632	7.62041336304505
C	0.21912507814173	6.73635993074222	6.90078285229842
H	-0.58748375676814	6.32556238777043	6.30886753669542
C	1.46111648104898	6.12791567555394	6.96664373145321
C	1.14792336915842	3.96413284728600	5.78595594171801
C	1.02325753996057	2.71369361956265	6.38584839979817
H	1.56814491285781	2.50626686096971	7.29651659128778
C	0.21469342308518	1.74757073352154	5.80301196646164
H	0.12376910744725	0.77451820268813	6.26823101773055
C	-0.48870346133914	2.03168212253906	4.63678867499695
H	-1.12377395316559	1.27864735677925	4.18886012240989
C	-0.37451296050179	3.28682125371443	4.04999779988072
H	-0.91891190139519	3.51375026719776	3.14251066900849
C	0.44778108619781	4.25208424425406	4.61799961231486
H	0.56168076395759	5.22484354662351	4.15859730045894
H	7.54192072235079	5.95683998369506	5.28331672142888

13.7.8 2-((1,3-dimethyl-1,3-dihydro-2*H*-benzo[*d*]imidazol-2-ylidene)amino)-*N*-phenylaniline

Illustration of the molecular structure. C-H hydrogen atoms are omitted. Colour code: C dark-grey, H light-grey, N blue.

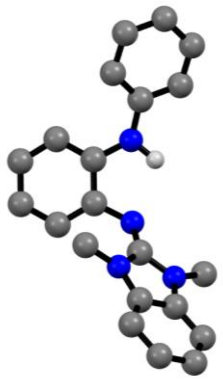

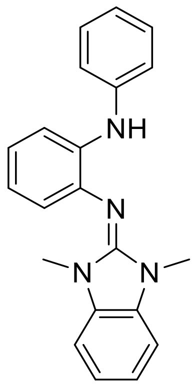

E_{el} = −1031.14178666

Cartesian coordinates (in Å)

N	3.23821231592752	3.89107688958805	4.07576861691629
C	2.35792096879139	2.82584006688488	4.27558622332066
N	3.17319736742239	4.94396583688022	6.27565547210832
C	1.26623524843979	2.70072738862834	3.37879623217900
N	4.77908179991401	5.48187234662430	4.84025803516874
C	0.43536718085217	1.58365888797701	3.47127719709036
H	-0.36977932463523	1.45406224144068	2.76423781272114
N	1.14337284045832	3.70548874433977	2.43045909948875
H	1.98017073623460	4.26551164671218	2.35306055500820
C	0.65447982598409	0.61517127913815	4.44567927881120
H	-0.00366705167385	-0.24251837101602	4.49755420794261
C	1.71921970541530	0.73513359088639	5.32807684583919
H	1.90144558117850	-0.02419605324548	6.07729552081829
C	2.57153980955110	1.82972677924429	5.22640814596620
H	3.42884251677198	1.91759093164741	5.88298503103363
C	3.66250154418808	4.67178921003236	5.00058830007196
C	3.98061673576948	5.89524698452324	6.89266632259786
C	3.91689945564676	6.48382794433516	8.14161377148914

H	3.14708604033753	6.21889818626015	8.85329014758162
C	4.89350134068719	7.43273991579777	8.46126270174656
H	4.86841583219208	7.90880404063950	9.43232105479762
C	5.89361231383421	7.77099540405431	7.55582082930873
H	6.63668683186699	8.50751364317126	7.83096519062423
C	5.95662890078271	7.17659505985959	6.29196880287161
H	6.73056123094615	7.44460358525249	5.58579873036243
C	4.98993982809869	6.23885792201280	5.97887894777252
C	1.87902469356856	4.53197133532827	6.78357627117130
H	1.19900412040166	4.35586325321394	5.95530251100694
H	1.47953518041100	5.33394950620343	7.40329308656557
H	1.94632589393803	3.61619832707345	7.37245314721388
C	5.61440186784249	5.47713542654699	3.66391496115495
H	6.66014969199196	5.34015754576008	3.94582277000331
H	5.51600710298105	6.41357149166496	3.10917821049160
H	5.29516706519423	4.65174453685401	3.03287441960155
C	0.08777565747051	4.04402909501674	1.59748685507583
C	-1.25128315464288	3.74728065108217	1.88442125111561
H	-1.50728978927628	3.22918205707882	2.79588455962809
C	-2.25896955082751	4.15650603647324	1.02097824052440
H	-3.28696475634106	3.91831230937267	1.26519295665311
C	-1.96948984782652	4.87346555797094	-0.13368918812844
H	-2.76158383693426	5.18694450149213	-0.80041873941647
C	-0.64246663541264	5.18575586774281	-0.41429752741030
H	-0.39356617159684	5.74694394668719	-1.30647060802396
C	0.37252788176706	4.77302313997516	0.43259679413512
H	1.40502001230942	5.00394131279404	0.19730995500093

13.7.9 *N*[1],*N*[2]-dimethyl-*N*[1]-(1-phenyl-1*H*-benzo[*d*]imidazol-2-yl)benzene-1,2-diamine

Illustration of the molecular structure. C-H hydrogen atoms are omitted. Colour code: C dark-grey, H light-grey, N blue.

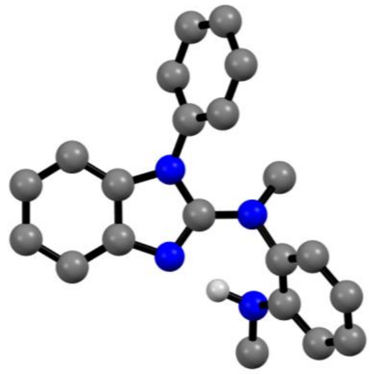

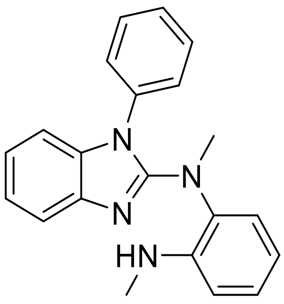

E_{el} = −1031.13131063

Cartesian coordinates (in Å)

N	3.62695757547767	3.99282802342417	3.51547658909419
C	2.90798502843533	2.78904049558319	3.82855219436184
N	2.80891562019969	5.27277906274580	5.35297953972718
C	1.49577210187033	2.75343081055197	3.84123439110697
N	4.73696232786301	5.94231230840158	4.40326371994870
C	0.87747800248463	1.50486756383676	3.99997238290675
H	-0.20089894963334	1.44106419940726	4.01304201765670
N	0.78518804930432	3.92674437336609	3.66792447518679
H	1.22972936351646	4.69901695816896	4.15454614578856
C	1.62852658524316	0.34730587744030	4.15412532758440
H	1.11777120674545	-0.59955962213703	4.27854073771650
C	3.01563746204371	0.39497403293808	4.16403025650802
H	3.60118467709332	-0.50481977590874	4.29501241727387
C	3.64162194519640	1.62521637007187	3.99476024895196
H	4.72216011235900	1.69500633314940	3.97360159829634
C	3.68797415773854	5.02391251039553	4.41071666416282
C	3.27829526543787	6.38737578910908	6.03160539180771
C	2.74267243284305	7.06504019570183	7.12185851668398

H	1.82334230097120	6.72466866942549	7.57981835703093
C	3.42613644790117	8.17708063369826	7.60082899301923
H	3.02913668135325	8.72158569421740	8.44788773513480
C	4.62323644235757	8.60401202147007	7.01570351718470
H	5.13643368827169	9.46630516738803	7.42086787924996
C	5.17142719630801	7.93931816429296	5.92168639514191
H	6.10182733555011	8.26344418643496	5.47609749720996
C	4.47689331724669	6.83935851157590	5.44451187255146
C	5.95747735806180	5.85514774334207	3.68646866832117
C	-0.65772045489302	3.92916102049566	3.78390509116526
H	-1.01123139928241	4.95190466163953	3.66049269222356
H	-1.01847660661275	3.55289935482610	4.75113540334031
H	-1.10825496438043	3.32259732896950	2.99539626295727
C	3.83392350776611	4.23008356839209	2.08449596151048
H	2.88510755070622	4.11499266963952	1.55432230740069
H	4.55934496458217	3.52366367248393	1.67851638731342
H	4.20319707286229	5.23809277313540	1.91708684174245
C	6.71036753205951	4.68452094636088	3.72809508300529
C	6.40540469710172	6.94842894806282	2.95042111747119
C	7.60625173277415	6.86864962681389	2.25636469829341
C	8.35350693923448	5.69679613751479	2.28326024710726
C	7.90088206744555	4.60626015942772	3.01873799970369
H	6.35500515428661	3.84687302823720	4.31140761597874
H	5.80606631224115	7.84837609815574	2.91699888889464
H	7.95216857173607	7.72080400254212	1.68570398103661
H	9.28494305437628	5.63384910666012	1.73606087048158
H	8.48238053575580	3.69392059855116	3.05027502076631

13.7.10 1,1,3,3-tetramethyl-2-(2-(methylamino)phenyl)guanidine

Illustration of the molecular structure. C-H hydrogen atoms are omitted. Colour code: C dark-grey, H light-grey, N blue.

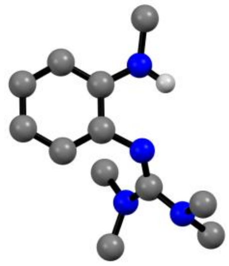

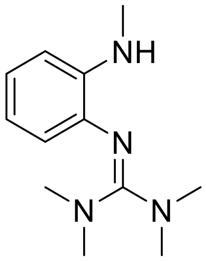

E_{el} = −688.19709031

Cartesian coordinates (in Å)

N	8.41058886244895	12.82657904680361	1.95221880932965
N	9.20667611765751	14.99000010893176	1.95117035947692
N	7.99449105377733	14.26538730650286	0.09603845559714
N	6.36934382222973	11.25068108288997	2.77383115777599
H	6.81396588818016	12.01298438356526	3.26580145414868
C	8.18701481247001	11.62999002313422	1.26428830353159
C	7.16525951464946	10.77556723723795	1.74576356926659
C	6.97579054892949	9.53242594175440	1.13989418998750
H	6.20135596812357	8.87051590774282	1.50276899098682
C	7.76794063420330	9.13776502100774	0.06417619103355
H	7.60232539557710	8.16967459184684	-0.39126512452633
C	8.76931644242652	9.97193985383741	-0.41037161063965
H	9.39723888671356	9.66350747471743	-1.23611161026666
C	8.97503544084131	11.20843909655519	0.19777564405282
H	9.77162946693515	11.85830412370719	-0.14460554646018
C	8.52363833775067	13.95582907399851	1.33912167015698
C	9.99625719395867	14.68349926655621	3.12833245514954
H	9.38389527655189	14.65204841984835	4.03792753332905
H	10.76080222457042	15.45384278055820	3.25058818577506
H	10.46931435557523	13.71301087088027	3.01018636199110
C	8.69318326836313	16.34909403283583	1.91236931165461

H	9.50789975336529	17.06331363358737	1.76924127753802
H	8.18409089897271	16.59850609146103	2.85223635790467
H	7.98240968231273	16.45925396688516	1.09961485520398
C	6.80773833857996	13.59014571081404	-0.39144946863296
H	7.04143788273981	12.74854953196904	-1.05109043480691
H	6.19915370079965	14.31048564780733	-0.94601835060273
H	6.22425581159053	13.21466441097825	0.44579303686754
C	8.78709088343352	14.93616531768866	-0.91598053482945
H	9.70408175058734	15.31972691469465	-0.47692492894718
H	8.23552158538223	15.76970421402385	-1.36288219109105
H	9.05227186384059	14.23744925908040	-1.71949229394283
C	5.56412361232873	10.35476035303430	3.57065185438296
H	4.74478090601293	9.93829698990492	2.97910996668366
H	5.12383692343289	10.91567302538723	4.39416370078546
H	6.13709589468773	9.51445828777139	3.98703640213605

13.7.11 *N,N*,1-trimethyl-1*H*-benzo[*d*]imidazol-2-amine

Illustration of the molecular structure. Hydrogen atoms are omitted. Colour code: C dark-grey, N blue.

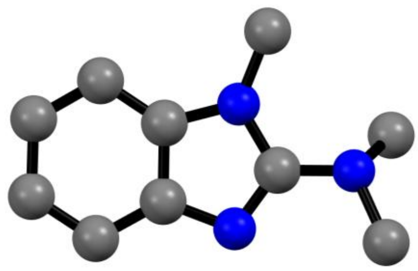

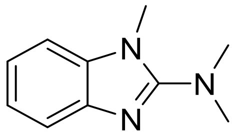

E_{el} = −553.06051137

Cartesian coordinates (in Å)

N	4.59325879266202	4.26126361625604	5.41867545573762
N	3.78514383989334	5.37962975688434	7.19825979536888
N	4.87027173308816	3.26386436523343	7.58313861532655
C	3.98451834364932	5.42823183826009	4.98252924725132
C	3.48255863022653	6.15088557714410	6.08633941966109
C	2.82675216171461	7.36553066423960	5.94776148074788
H	2.45003846177752	7.91509895298219	6.80085237141181
C	2.67662379649734	7.85562970613454	4.65383724724171
H	2.17004118824169	8.79960745233065	4.50046657833481
C	3.17220629516141	7.15324207464274	3.54840317743572
H	3.03978007672767	7.56922417154287	2.55792342737901
C	3.82893749925337	5.93722778219243	3.69681553955954
H	4.21116252714371	5.39304309822863	2.84324591393988
C	4.45531813606524	4.26405597795560	6.72147821174793
C	5.19559163881202	2.00125093156376	6.92847103348649
H	4.39511392131317	1.72737445260574	6.24612816250156
H	5.29662204560003	1.23281287947513	7.69589287998855
H	6.12763384023844	2.05975952929974	6.35427543641514
C	5.86505366226322	3.64706933593606	8.58498721188464
H	5.60466116381913	4.59524036352616	9.04787107946471
H	6.86551938305955	3.74501244824400	8.14178236551845

H	5.90199955487602	2.88379809200045	9.36294751056112
C	3.25509147823393	5.58102935871624	8.52733930062990
H	2.24391160266920	5.98304739480572	8.45471107173352
H	3.86422903174871	6.27581602972041	9.11163715143266
H	3.21391119526452	4.62212415007917	9.04023031523932

13.7.12 2-((1,3-dimethylimidazolidin-2-ylidene)amino)-*N*-methylaniline

Illustration of the molecular structure. C-H hydrogen atoms are omitted. Colour code: C dark-grey, H light-grey, N blue.

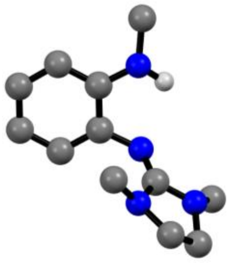

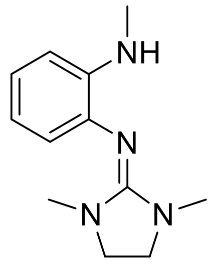

E_{el} = −687.00508294

Cartesian coordinates (in Å)

N	4.16766842403886	10.89876323050980	14.27291221970481
N	6.37180588777507	10.93135450404923	15.01750607651782
N	6.01351076593708	11.78925659900076	12.97187305915513
N	2.81946091579843	8.92698083009632	12.98010375718158
H	3.43585138939050	8.84107973156484	13.77518093731577
C	3.11206730831977	11.26135348981237	13.43002188686157
C	2.36705150321626	10.22489707511879	12.81646364701404
C	1.25082779816039	10.55614028397067	12.04513737901172
H	0.67267201749814	9.77318735605544	11.57382316331474
C	0.87928812088606	11.88644358862538	11.86718038068079
H	0.01285166856303	12.12016815578957	11.26151813018868
C	1.60487700441729	12.90270060418947	12.47153012066279
H	1.31234864458830	13.93714389526533	12.34627897607566
C	2.71096438078510	12.58149232054471	13.25554550136479
H	3.28100194158818	13.36047785679851	13.74686041403939
C	5.39494842823987	11.19488986214303	14.06861351346373
C	7.59594265531143	11.62799540238615	14.66930203856440
H	8.47857177921451	11.06659741724917	14.97847314454662
H	7.63468175140275	12.62129955811011	15.14036587972774
C	7.46111353103144	11.73890063650593	13.15680957849718

H	7.94121268338383	12.62804774117282	12.74668457387822
H	7.88417463091405	10.85421847296854	12.66098427831251
C	6.02035081050367	10.77082435344986	16.40893664214069
H	5.93282509380201	11.73834704018428	16.92214959041264
H	6.78459010371564	10.17701916546929	16.91280848027967
H	5.06357861357475	10.25943720678610	16.47047224674434
C	5.54072508512688	11.56604317454432	11.61526499006900
H	5.67580214124669	10.52142058253609	11.30746564599422
H	6.11522959261523	12.20493899725339	10.94408430628993
H	4.49157285355728	11.82476769112434	11.52249593716591
C	1.96402338196441	7.79634605094162	12.70608880297234
H	1.76916446009230	7.70739234508604	11.63409392615411
H	0.99387675097196	7.85422885027106	13.21918399041296
H	2.47093788236869	6.88642593042637	13.02509678528412

13.7.13 N^1,N^2-dimethyl-N^1-(1-methyl-1*H*-benzo[*d*]imidazol-2-yl)ethane-1,2-diamine

Illustration of the molecular structure. C-H hydrogen atoms are omitted. Colour code: C dark-grey, H light-grey, N blue.

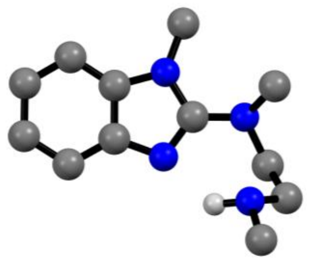

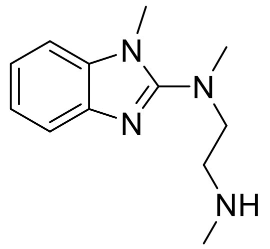

E_{el} = −687.00625821

Cartesian coordinates (in Å)

N	0.33919942435123	4.20528002650565	6.71174955318650
N	-0.90065262099333	5.08983060581347	5.05647468432701
N	0.26805913756427	6.59447722034004	6.52892557005867
N	0.28084292001660	5.75570584191251	9.31890576535561
C	-0.21019901174337	3.17689870118119	5.96421281283803
C	-1.00700470438438	3.71076446929987	4.93003991706604
C	-1.69384593654325	2.91063064725475	4.02963438450991
H	-2.30791193867064	3.32871610921614	3.24234317137126
C	-1.56420078384196	1.53267521771663	4.18378733515095
H	-2.08239217076731	0.87215000324880	3.50083258354526
C	-0.78075232153704	0.98749728337364	5.20736461542116
H	-0.70731014796979	-0.08861957379712	5.29874934627970
C	-0.09606776276418	1.79769860346736	6.10653983470447
H	0.51156313996139	1.37647759653672	6.89677147771045
C	-0.08315600788536	5.31858847412309	6.15324953146023
C	-1.29726894782870	6.04318178107125	4.04444945593230
H	-2.32791115507476	6.38186181486650	4.17790966940958
H	-1.21009123886855	5.57670975529817	3.06285133829761
H	-0.63067213377445	6.90277365322812	4.08164995032365
C	1.46118855624783	6.72155998806708	7.36774383348350

H	1.97905828166915	7.64087267088229	7.07633398359973
H	2.10802225879842	5.88165754878907	7.12457539650385
C	1.21760158596186	6.76761686915004	8.87967248985905
H	2.20823531463415	6.68114717363496	9.36226730925333
H	0.82903249005906	7.75138148457282	9.17021562469809
C	-0.78952407114564	7.55994543221544	6.82076863313898
H	-1.61194778528030	7.45705847258889	6.11924464339877
H	-0.38666743183807	8.57003418870704	6.72672090337494
H	-1.18026755574245	7.41828906796309	7.83280386010122
C	0.24085505803482	5.57500698935182	10.75555835897661
H	-0.11835529331659	6.48995677170251	11.23635796326444
H	1.22030886147494	5.33249797899285	11.20168985884271
H	-0.45583274424517	4.77378425934056	11.00540646972937
H	0.43997473544159	4.88486287338459	8.82261967482678

13.7.14 (Z)-N-methyl-2-((3-methylthiazolidin-2-ylidene)amino)aniline

Illustration of the molecular structure. C-H hydrogen atoms are omitted. Colour code: C dark-grey, H light-grey, N blue, S yellow.

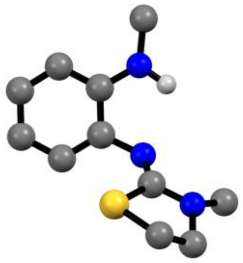

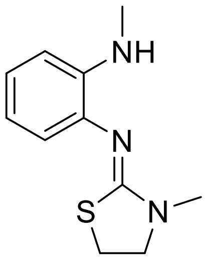

E_{el} = −990.52333272

Cartesian coordinates (in Å)

N	4.17056286794433	10.94893223335395	14.26165079879435
N	6.40443701246785	11.00935687080328	14.91681629418366
S	5.95077527272711	12.40008292755536	12.75134677960660
N	2.77664125759214	8.95873674688726	13.04985194968005
H	3.40233072379283	8.88208883682966	13.83691166191192
C	3.12554351521745	11.30721933022750	13.39666537784912
C	2.37149495440380	10.25723899896812	12.81678695341371
C	1.27558852471123	10.58153504068459	12.01294812434078
H	0.68695579644693	9.79320140498679	11.56464555167296
C	0.93630151936954	11.91059822682561	11.77617703270032
H	0.08379473887839	12.13535175529945	11.14763001914835
C	1.67092698837060	12.93795430150261	12.34833192211282
H	1.39979315578448	13.97148052077026	12.17851093973259
C	2.75817122515117	12.62698784373799	13.16205490406325
H	3.32033131295807	13.41567559555120	13.64541112391183
C	5.36277916394633	11.35143662349521	14.08508670218025
C	7.59931998179329	11.81449149779834	14.74978017987518
H	8.47249584567538	11.25021501326262	15.08339644996226
H	7.53966809343143	12.73708888015550	15.34547361044674
C	7.68258619489920	12.13875694193563	13.26622339327586

H	8.25880674526340	13.04019348337806	13.07107014521599
H	8.10623035580370	11.30752435182468	12.70311874024462
C	6.12480327442398	10.45763027163723	16.22647576990925
H	5.90404923029982	11.24396755004171	16.95965219075367
H	6.98918354110382	9.88835993167392	16.56997904289496
H	5.26056178137016	9.80311512838178	16.15788138162640
C	1.90321644318158	7.83723402241477	12.80052003084155
H	1.73279444611540	7.70666273011244	11.72857223537694
H	0.92268269379603	7.93824903552939	13.28623569884878
H	2.38078334308042	6.93087390437465	13.17052499542473

13.7.15 2-(Methyl(1-methyl-1*H*-benzo[*d*]imidazol-2-yl)amino)ethane-1-thiol

Illustration of the molecular structure. C-H hydrogen atoms are omitted. Colour code: C dark-grey, H light-grey, N blue, S yellow.

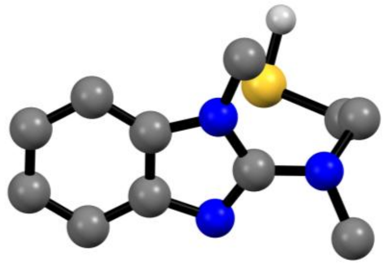

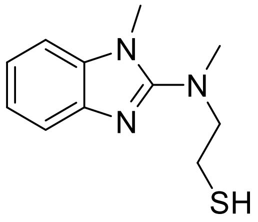

E_{el} = −990.52491627

Cartesian coordinates (in Å)

S	11.93973335916278	0.77961780973396	11.62094669263354
N	11.52744641893185	2.10981857088450	8.43607262973240
N	9.37128764240170	1.46732575369301	9.31653867635574
C	9.21922351842248	0.21355571585108	8.62303674844115
N	10.20611818322063	3.47180951444872	9.89913199834577
C	13.04372841988027	1.10044729226776	10.19688378105458
H	13.89905573990354	0.42797190721033	10.25996125498339
H	13.41843676296937	2.11208450348367	10.35088424999522
C	12.37506438469109	0.97768989577697	8.82371070057735
H	13.15200079521186	0.89722877966450	8.05917933532147
H	11.79942270342093	0.05365892019791	8.78557528984831
C	10.41297578532238	2.36884911169568	9.22111697519324
C	8.44471347736703	2.03546442584875	10.17469747503585
C	7.22702935501812	1.58345571310799	10.66309198908449
H	6.83189883272929	0.60859779888865	10.40775853133418
C	6.53047903298111	2.44150543544157	11.50837315677363
H	5.57671616324938	2.12809819462600	11.91270067207131
C	7.04184362354044	3.70005497141571	11.84965902026554
H	6.47230884577635	4.33741465562764	12.51384481699799
C	8.26291371727040	4.14438295270828	11.35713049712432

H	8.66019784333902	5.11546901436259	11.62185735299074
C	8.97488232709192	3.30043145908901	10.50975659760383
C	12.27830927269384	3.30724144381388	8.06452130933936
H	11.59535455219963	4.04197690430018	7.64531736192822
H	13.01475460446150	3.02882176406770	7.31001804461084
H	12.78797641768709	3.77808027175689	8.91120485974455
H	9.78981363525792	0.24396387851534	7.69641654686264
H	8.16765675704330	0.06480076403004	8.37786417454190
H	9.55944799568304	-0.62991880464868	9.23096860257815
H	11.95023983307102	-0.56026861785974	11.51597065862952

13.7.16 2-((1,3-dimethyl-1,3-dihydro-2*H*-benzo[*d*]imidazol-2-ylidene)amino)-*N*-methylaniline

Illustration of the molecular structure. C-H hydrogen atoms are omitted. Colour code: C dark-grey, H light-grey, N blue.

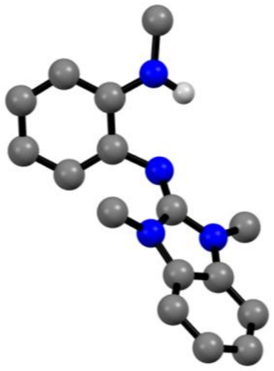

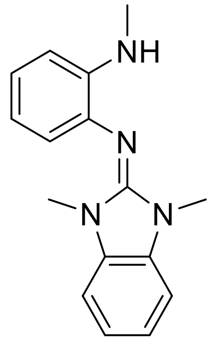

E_{el} = −839.42917979

Cartesian coordinates (in Å)

N	3.24732645426223	3.99225487290377	4.06766951211718
C	2.34497905878779	2.93950331313526	4.25412369783982
N	3.21243241282367	4.96692920066831	6.30358655764266
C	1.19305909217421	2.90664273654474	3.42916799918322
N	4.81563840545473	5.53593393804461	4.87573868356284
C	0.32135870604629	1.82040427786764	3.52487958015960
H	-0.56013794727387	1.78334451517839	2.89940126561600
N	0.96978805809480	3.98896719277567	2.59690889244694
H	1.80848734673932	4.53232657220253	2.45289147567681
C	0.56812503720560	0.78647714809530	4.42510125022272
H	-0.12357849582284	-0.04439936700305	4.48262250845695
C	1.69723029823988	0.81357562307186	5.23002173480354
H	1.90250356594062	0.00461845856003	5.91901360164598
C	2.58144077670166	1.88587245817049	5.13180295359864
H	3.48304346189172	1.90798749900870	5.73266602742076
C	3.68783276785327	4.73456785995402	5.01406959735681
C	4.03949651097876	5.88011732238259	6.94975040127807
C	3.99636469958548	6.42088693106892	8.22096350151955

H	3.22847452258400	6.14073434339290	8.92899769552375
C	4.99097158682257	7.34116410792238	8.56928378275291
H	4.98136174195140	7.77865611047214	9.55871706114184
C	5.98889659848874	7.69858774818342	7.66939617964348
H	6.74672883287897	8.41134184036203	7.96644358576440
C	6.03139362310690	7.15296624361825	6.38243813734604
H	6.80383894180772	7.43607591433255	5.68037931498259
C	5.04730918120545	6.24383574455451	6.04087579255758
C	1.91759413278429	4.54976951415042	6.80542320416222
H	1.22019913373111	4.44352335594262	5.97912583380608
H	1.54609107930602	5.31862186990919	7.48204537054363
H	1.97245076080715	3.59631160245840	7.33248049268482
C	5.64463924527188	5.55676590738811	3.69582134948220
H	6.69003633249910	5.39617424311717	3.96720299269693
H	5.55760256616842	6.51111428633518	3.16994372628092
H	5.30917750917498	4.75625773803326	3.04118604067840
C	0.05956745921555	3.90055497790348	1.47939254269691
H	0.13494184647807	4.81447931850603	0.89143742748248
H	-0.97400678477578	3.81500426759560	1.82497487731204
H	0.26541148081000	3.04402031319240	0.82245535191247

13.7.17 N^1,N^2-dimethyl-N^1-(1-methyl-1*H*-benzo[*d*]imidazol-2-yl)benzene-1,2-diamine

Illustration of the molecular structure. C-H hydrogen atoms are omitted. Colour code: C dark-grey, H light-grey, N blue.

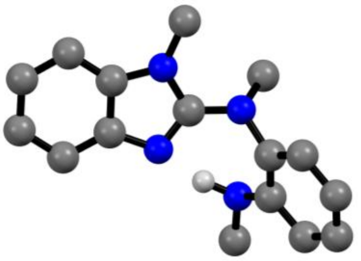

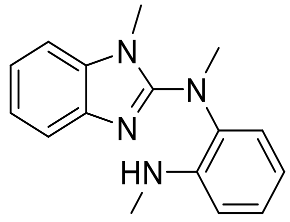

E_{el} = −839.42132368

Cartesian coordinates (in Å)

N	3.80497989412123	3.63804992372850	3.73620377175002
C	2.78594556656251	2.63936720212988	3.93139632004490
N	2.95751899609089	5.25878421751097	5.30159932931324
C	1.41834519833712	2.90474619463821	3.69075927702956
N	5.16754733821974	5.22994450328440	4.90589273931105
C	0.52489532555365	1.82554557374350	3.75217426026271
H	-0.52652791994816	1.99285334266426	3.56883182324181
N	1.01761018254237	4.19186467795513	3.38103381586100
H	1.52612196112878	4.87833552867872	3.93227485722720
C	0.96519737801936	0.54509243000406	4.05988498778819
H	0.24604487893255	-0.26358866115287	4.10301532716030
C	2.30504488277280	0.29893786224556	4.32467351063809
H	2.64842955581078	-0.69550412900952	4.57542942108934
C	3.20447340942444	1.35730984484212	4.25069167356616
H	4.26089669903842	1.19773980601308	4.42691588597371
C	3.92705498108247	4.68273351038859	4.62714097014577
C	3.56507405241987	6.22879778980479	6.08250288397983
C	3.01948407245271	7.13054840918601	6.99125626946763
H	1.95626044776740	7.13140327196215	7.19221122411315
C	3.87917093220371	8.01817872942360	7.62692752467690

H	3.47867370589361	8.72920637706135	8.33795907180062
C	5.25615689177121	8.01329827745655	7.37015947153848
H	5.89534129202889	8.71709955697788	7.88711823067392
C	5.81897638984976	7.12190237824692	6.46217135959917
H	6.88261927058214	7.12414779297736	6.26203797114014
C	4.95396567273376	6.23865126151830	5.83203759548427
C	6.45614693365943	4.72981958008682	4.48648708182971
H	7.13571257628518	4.71720873344004	5.33948381933047
H	6.89470901726411	5.34705434780831	3.69841140714700
H	6.33433640337157	3.71376443537016	4.11765806992689
C	-0.38825717528463	4.50172021759551	3.23135678485364
H	-0.48609423424922	5.56842736636046	3.03418952665775
H	-0.98647767339641	4.25890146783237	4.12049021517577
H	-0.81322604761435	3.96414615266254	2.38090269098549
C	4.14101387905684	3.89214593206156	2.32888367193798
H	3.25794064322350	4.21359352852398	1.76848319208321
H	4.53733580373389	2.98180213413830	1.87893001791714
H	4.89552881855793	4.67367043184020	2.26152394927760

14 Quantum chemical calculations on the cyclisation pathway

14.1 Details of the quantum chemical calculations

Density functional calculations are performed with the program ORCA[1,2]. The B3LYP functional[3,4,5,6] is used with the def2-SV(P) and def2-TZVP basis sets[7]. The calculations include the D3 dispersion correction with Becke-Johnson damping[8,9]. The resolution-of-the-identity (RI) approximation for the coulomb integrals[10] is used with corresponding auxiliary basis sets[11] and the chain-of-spheres approximation for the exchange integrals[12] (RIJCOSX).

The calculations determine the molecular structures and subsequently calculate the harmonic vibrational frequencies. To determine the transition structures, calculations with the method of the nudged elastic band[13] (NEB) are performed, also combined with the procedure of the climbing image[14] (NEB-CI), in which the highest-lying structure of the path is moved uphill along the tangent to the path. Then the transition structure is optimised[15] (NEB-TS).

Further calculations include a dielectric environment using the conductor-like polarisable continuum model[16](C-PCM) with a value for ε_r of 78.4.

References

[1] F. Neese, *WIREs Comput. Mol. Sci*. **2012**, *2*, 73-78.

[2] F. Neese, *Wiley Interdiscip. Rev.: Comput. Mol. Sci.*, **2022**, *12*, *1*, e1606.

[3] A. D. Becke, Phys. Rev. A 1988, 38, 3098-3100.

[4] C. Lee, W. Yang, R. G. Parr, *Phys. Rev. B* **1988**, *37*, 785-789.

[5] A. D. Becke, *J. Chem. Phys*. **1993**, *98*, 5648-5652.

[6] P. J. Stephens, F. J. Devlin, C. F. Chabalowski, M. J. Frisch, *J. Phys. Chem*. **1994**, *98*, 11623-11627.

[7] F. Weigend, R. Ahlrichs, *Phys. Chem. Chem. Phys*. **2005**, *7*, 3297-3305.

[8] S. Grimme, J. Antony, S. Ehrlich, H. Krieg, *J. Chem. Phys*. **2010**, *132*, 154104.

[9] S. Grimme, S. Ehrlich, L. Goerigk, *J. Comput. Chem.* **2011**, *32*, 1456-1465.

[10] F. Neese, *J. Comput. Chem.* **2003**, *24*, 1740-1747.

[11] F. Weigend, *Phys. Chem. Chem. Phys.* **2006**, *8*, 1057-1065.

[12] B. Helmich-Paris, B. de Souza, F. Neese, R. Izsák*, J. Chem. Phys.* **2021**, *155*, 104109.

[13] G. Henkelman, H. Jónsson, *J. Chem. Phys*. **2000**, 113, 9978-9985.

[14] G. Henkelman, B. P. Uberuaga, H. Jónsson, *J. Chem. Phys*. **2000**, *113*, 9901-9904.

[15] V. Ásgeirsson, B. O. Birgisson, R. Bjornsson, U. Becker, F. Neese, C. Riplinger, H. Jónsson, *J. Chem. Theory Comput*. **2021**, 17, 4929-4945.

[16] V. Barone, M. Cossi, *J. Phys. Chem. A* **1998**, *102*, 1995-2001.

14.1.1 dmegab

Illustration of the calculated minimum structure of dmegab.

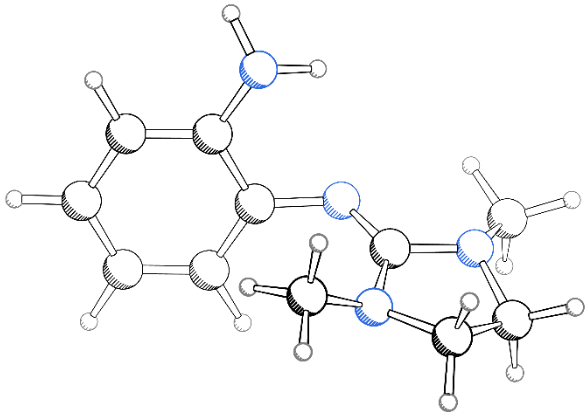

E_{el} = -647.70982018

Cartesian coordinates in Å:

C	3.04367164499130	11.30876112989018	13.49855363401625
C	2.23203949975650	10.27902519781448	12.97855468837921
C	1.12403465026921	10.61034222578078	12.19948298463521
C	0.80991930392487	11.93805764339343	11.93395964376062
C	1.60176447847680	12.95633880766790	12.45132527195675
C	2.70632790941160	12.63526290188609	13.23324017544736
H	0.50939170742516	9.81510379050955	11.79229336578864
H	-0.05579038108911	12.17259116872944	11.32777984221516
H	1.35899529496574	13.99312234901915	12.25759656430159
H	3.32691986249255	13.41641416474096	13.65488457404086
N	4.09709765313487	10.95574484727273	14.34743625215876
C	5.33325635475111	11.16841633092861	14.09840623309435
N	5.95903148666858	11.63383790328391	12.94533495849991
C	5.44454175460607	11.32962952049383	11.61999733347362
H	5.50738155137083	10.25697797429731	11.39770002736016
H	6.04273620091124	11.87376865789441	10.88861351207113
H	4.41172073864959	11.64600077044713	11.52256129837819
C	7.40537947366935	11.51920035199563	13.11012292208469
H	7.92420030315233	12.34065911969715	12.61481710777273

H	7.76927414206573	10.57195184203046	12.68840705505317
C	7.56482756131437	11.53385933926918	14.62401634395613
H	8.42478429358365	10.95779800335558	14.96801477969490
H	7.66170951596296	12.56169883710318	15.00420218930335
N	6.31467557961674	10.93319390334299	15.05092240350159
C	5.98082774960404	10.92091334923361	16.45617185092088
H	5.93396735556738	11.93601592455948	16.87403221107411
H	6.73305853746384	10.35184113846446	17.00457674787973
H	5.00857892927548	10.45195856398744	16.58059589618470
N	2.61265541529339	8.95624671491224	13.20156095379908
H	3.21355971188832	8.85887842152270	14.00902613827178
H	1.85757164527648	8.28827191567084	13.18860181211436

14.1.2 maemabi

Illustration of the calculated minimum structure of maemabi.

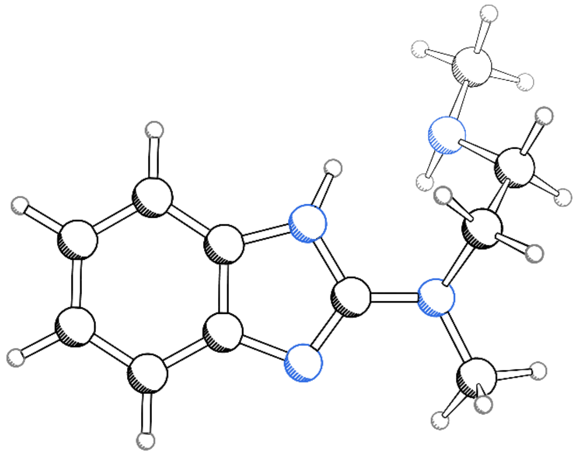

E_{el} = -647.71367373

Cartesian coordinates in Å:

C	-1.93873572640138	0.89920413649933	-1.34093046113843
C	-1.99359367799481	-0.11529193203962	-0.35874807550401
C	-3.18821622182049	-0.58839921635155	0.15858410815865
C	-4.36035191083583	-0.01589734302547	-0.33077773953445
C	-4.32419753762177	0.98414658258589	-1.30819765692610
C	-3.12009669534212	1.45192881529733	-1.82486845061423
H	-3.21641207443057	-1.36792868195299	0.90939591353538
H	-5.31514903626411	-0.35414597372671	0.05027207104223
H	-5.25532474732194	1.40233417453262	-1.66885294009917
H	-3.09275331610873	2.22530596523580	-2.58115811785136
N	-0.61777480913199	1.17320626347026	-1.67411096427310
C	0.09698167465150	0.37315973526088	-0.91880895657776
N	1.46580565351408	0.34232408713315	-0.86017365143793
C	2.19152605555166	1.25390924565741	-1.73005143812202
H	2.28826616822126	0.85833422430467	-2.74812296260438
H	3.18712076796248	1.42221515375308	-1.31827444362428
H	1.65767402348135	2.19855863197215	-1.78378871857658
C	2.13550115489841	-0.90275867384247	-0.49012259794983
H	2.97621876181772	-1.05752161549556	-1.17064383252050

H	1.44740689594230	-1.73817153329865	-0.63500553112950
C	2.64148374291988	-0.91330209930634	0.94805862556206
H	3.05357703473644	-1.90365752431644	1.16153915154704
H	3.46507705561124	-0.19106583129981	1.06483770505943
N	1.55139395071177	-0.65885191725232	1.88566441290804
C	1.92658408672512	-0.86974860789110	3.27967956472234
H	2.81685476983246	-0.29970407449947	3.58408036516692
H	2.13390937313125	-1.92868070862081	3.44481490201333
H	1.09726747760517	-0.58423300315533	3.92723863574640
N	-0.66728238786365	-0.45604719989499	-0.12129374764661
H	1.25293946094884	0.30360984341702	1.76446562326219
H	-0.29569996712555	-0.94283092314993	0.68529920740624

14.1.3 TS(dmegab–maemabi)

Illustration of the calculated transition structure between dmegab and maemabi.

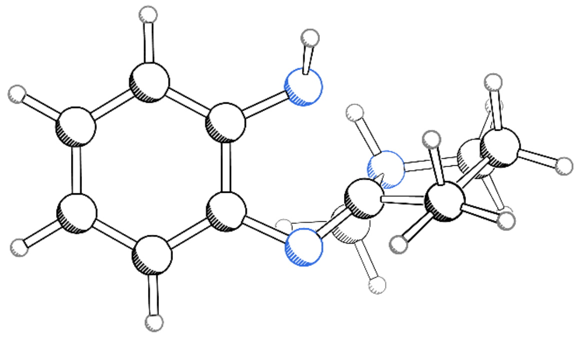

E_{el} = -647.62045921

Cartesian coordinates in Å:

C	-1.41642848824647	0.85199284659573	-0.19179613631075
C	-1.57414266605156	-0.51701768874609	-0.56148074439555
C	-2.86348159611993	-0.99768792204132	-0.79605722580252
C	-3.97236372626455	-0.17540064330292	-0.60881462871284
C	-3.80923881674896	1.14536286354231	-0.20109019003152
C	-2.53151489363846	1.66124994618101	-0.00037941092695
H	-2.99858666099901	-2.03127547070587	-1.09420977453792
H	-4.96616580072112	-0.57254463230013	-0.77505922581645
H	-4.67433868208980	1.77945378496413	-0.05322361504998
H	-2.38614439524626	2.69839353887009	0.27475887519511
N	-0.10255684409371	1.30535464193837	-0.17341194365048
C	0.77877426270982	0.37733284259150	-0.12104697359824
N	2.05257422237453	0.42848161551202	-0.65694721258599
C	2.14418858233754	0.80322856918652	-2.05534728747953
H	1.83752838829196	-0.02425308555703	-2.70998073858090
H	3.17081849803626	1.08542555424476	-2.29027117568553
H	1.48546827018168	1.65012830965942	-2.22867735582655
C	2.86575111863064	-0.68384208834104	-0.21331680871011
H	3.92683989028564	-0.45432946882417	-0.31247888208194

H	2.65387181428052	-1.59529298797476	-0.79186630494677
C	2.45841381436883	-0.84780085460577	1.24662020755225
H	2.62365284390291	-1.85290123222725	1.63285730662642
H	2.98310576651699	-0.12928779252193	1.87540298277552
N	1.02381770145112	-0.50730512445940	1.24860214018510
C	0.49809457982845	0.12608013195202	2.46370176439350
H	1.04162906082129	1.04945341459758	2.65529396979103
H	0.60810750619740	-0.55555019564941	3.30728967139084
H	-0.55184346570110	0.35760758662273	2.30675929111633
N	-0.38934588677502	-1.23374071985464	-0.63216677676642
H	0.42544838360558	-1.30298509193361	0.90350098231676
H	-0.34193278112523	-1.83833064741286	-1.44716477984592

14.1.4 dmegHab⁺, Tautomer 0

Illustration of the calculated minimum structure of dmegHab⁺, Tautomer 0.

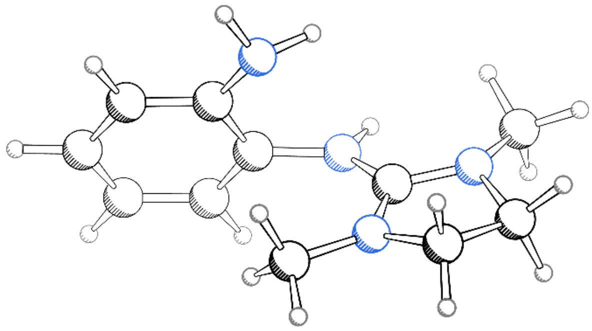

E_{el} = -648.11604519

Cartesian coordinates in Å:

C	2.93025271796320	11.57789520939420	13.59337805211032
C	2.54391823312069	10.41879513307251	12.90698814123142
C	1.47177827895294	10.52174974266532	12.01445686300920
C	0.80235749766729	11.72170924978878	11.84231616221910
C	1.17702128436070	12.85741855336588	12.55692951227034
C	2.24767548696705	12.77746935073429	13.43229944640902
H	1.15995127840593	9.64332660821070	11.46288555768936
H	-0.02785775577507	11.76932036703191	11.15026655641913
H	0.64524558679979	13.78944737750358	12.42932022185900
H	2.57473041153521	13.64718493704817	13.98805959306354
N	4.04306535640791	11.51518305500667	14.50027170942723
C	5.32614328772652	11.31306631160061	14.18261936400329
N	5.88178997469215	11.39484274663690	12.97137066379381
C	5.24717619450450	11.58614323741668	11.67197311827256
H	4.83601798718009	10.64888881931730	11.29526095759682
H	6.01312975138489	11.94573534127133	10.98793991763334
H	4.46163118770284	12.33090934615867	11.73270282501322
C	7.27337897523321	10.92246471003969	13.04316757439782
H	7.91290459153000	11.54311721157182	12.41977145585671

H	7.33334653137342	9.88723477010382	12.69594112261767
C	7.58444292037528	11.04923218655190	14.53372514757647
H	8.20493912846779	10.23900272447348	14.91042768569015
H	8.06009209529652	12.00500332756984	14.77460859254155
N	6.24045914052834	10.98517465247468	15.11946174704266
C	6.03480141495331	11.08975147726708	16.55221621729239
H	6.03485359216543	12.12902873917203	16.89540817335384
H	6.84091262182593	10.55851922549301	17.05344566715539
H	5.10094114421301	10.60770639489069	16.84298396332851
N	3.26441692384705	9.23513406384945	13.04136020323257
H	3.63479787459577	9.05236066954148	13.96208020618862
H	2.80702371538089	8.42052335690426	12.66021246518739
H	3.84059648819783	11.60403238685961	15.48413994677462

14.1.5 dmegHab+, Tautomer 1

Illustration of the calculated minimum structure of dmegHab+, Tautomer 1.

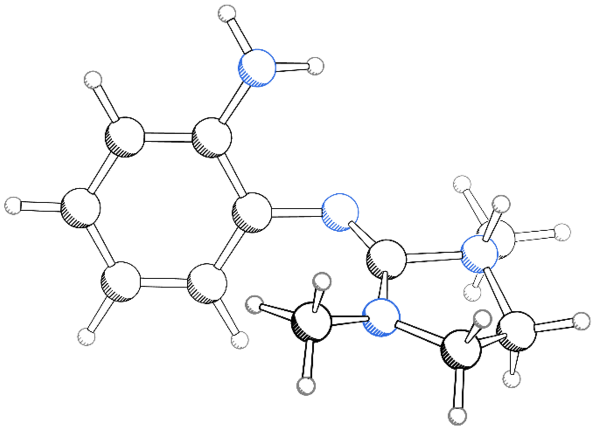

E_{el} = -648.07146857

Cartesian coordinates in Å:

C	3.03088252093105	11.20373704692655	13.42654745701170
C	2.10938510830525	10.20362595712094	13.07448308011583
C	0.97612289877303	10.58001794072486	12.34612841572914
C	0.75266983968967	11.90397356916491	12.00590531923664
C	1.65505541528721	12.89406650252920	12.38575138468348
C	2.79119015107304	12.53645809017707	13.09466885640098
H	0.26464631299733	9.81755008234104	12.05316721386879
H	-0.13760361162286	12.16686212626546	11.45045142492216
H	1.47109266363649	13.93092668745727	12.14172788280186
H	3.49661033535579	13.29362161374332	13.41543010254309
N	4.14996890658326	10.82945924664423	14.20851198736955
C	5.34452105627190	11.07058984767506	13.93469021397163
N	6.07399514269235	11.56891399751512	12.90266550215822
C	5.59205960995630	11.60508905665133	11.52387795394342
H	5.80753012919803	10.66000449970375	11.01805611623126
H	6.09628097211568	12.41569992669523	10.99998673992470
H	4.52256299675565	11.78283098937779	11.50651557610941
C	7.51243421229433	11.52503461090317	13.13038644119647
H	7.99678024908014	12.41982156114047	12.74048607278001

H	7.96287949834063	10.65282013755875	12.64216049942843
C	7.61527574965384	11.44886136278901	14.64498518232973
H	8.49256257946238	10.92573513430867	15.01539691154638
H	7.55175649616461	12.43363611468433	15.10282242081034
N	6.36679045770610	10.70833317618196	15.02461513565541
C	5.87596839968682	10.95631767634476	16.41208177647576
H	5.70293925182388	12.02292736283213	16.52521200615964
H	6.63372979631742	10.61140316366950	17.11205803412889
H	4.94242740652027	10.41852811568578	16.54006284123463
N	2.37382680433805	8.87632729683506	13.37776866375624
H	2.95261127448052	8.73328965063486	14.19130672817001
H	1.57659479713461	8.25982003220006	13.35056007163888
H	6.53221661001794	9.70391173224806	14.92463983100078

14.1.6 dmegHab$^+$, Tautomer 2

Illustration of the calculated minimum structure of dmegHab$^+$, Tautomer 2.

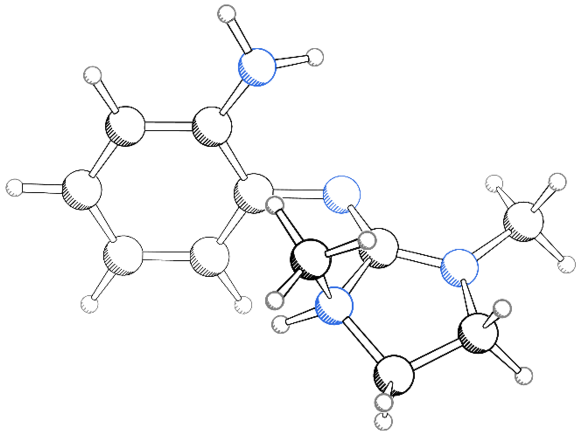

E_{el} = -648.07810304

Cartesian coordinates in Å:

C	2.96218903671384	11.45673732765772	13.59575605853147
C	2.23314229255956	10.42740311740352	12.97698645875663
C	1.26733723593431	10.77689918798797	12.02983069299487
C	1.02800471679401	12.10512319229601	11.70881186314489
C	1.74184895026581	13.12242282758287	12.33285922680554
C	2.70410472064397	12.79162630015399	13.27988547117061
H	0.70181454733554	9.99223959752306	11.54183428367899
H	0.26859465747443	12.34604803566780	10.97723449927766
H	1.53608503401249	14.15935155662198	12.10673080792992
H	3.22451155283331	13.56955943031186	13.82848907595881
N	3.94939119990122	11.11192017527821	14.55387253318664
C	5.16145360342020	11.30991781535054	14.30193521665278
N	5.66866590276973	11.72076387476575	12.91727752614732
C	5.64719496028331	10.54269653705900	11.98427394313341
H	6.35769503085166	9.80459785456609	12.34704065660640
H	5.93001551694721	10.88860647050862	10.99308528492191
H	4.64550848767727	10.12227708048299	11.97866568625069
C	7.03695996252458	12.28050373475168	13.16739351622983
H	6.92411723859126	13.33709063323078	13.40071888355572

H	7.65841208604261	12.15882795107316	12.28496272954065
C	7.50605910722619	11.49075319740807	14.38924746330490
H	8.03719276367382	10.57357007202982	14.11504758776842
H	8.16673447291820	12.08828123932818	15.01714140605763
N	6.25765619860590	11.20888060664478	15.07668724983835
C	6.20713774827212	10.71970424775858	16.44458114809219
H	6.80724558962660	11.36486374502429	17.08617671378240
H	6.58822838237615	9.69781341029679	16.50587108748751
H	5.17276532080914	10.73740207319532	16.77783861928037
N	2.56619708555414	9.10332983707876	13.24222022808849
H	2.92436686261047	8.94093444798986	14.17301286407369
H	1.86742879165434	8.42528512016444	12.97761841571709
H	5.00923748873560	12.41969690885680	12.56093401302672

14.1.7 dmegbNH⁻

Illustration of the calculated minimum structure of dmegbNH⁻.

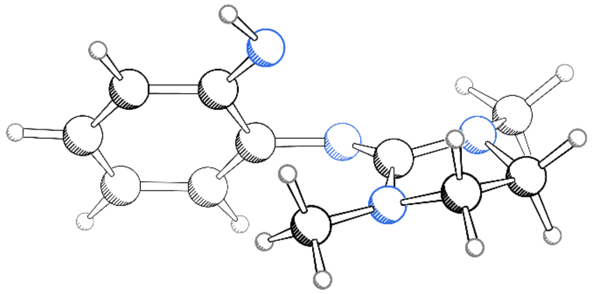

E_{el} = -647.09950525

Cartesian coordinates in Å:

C	3.01410772656290	11.68575469702969	13.58244332998824
C	2.68533836023080	10.48067579885517	12.83391014319404
C	1.48352107029614	10.58424401556555	12.05968357820965
C	0.69658157684504	11.72675451409022	12.01770257281208
C	1.04062805776883	12.86221008293109	12.74719116923190
C	2.19950690738485	12.80212784935872	13.53795991315809
H	1.18916667122904	9.71122448103023	11.48126266010666
H	-0.19828140110274	11.72984637746223	11.39983736536970
H	0.42456239148126	13.75339192118373	12.73194851870006
H	2.49125085485585	13.65572772133158	14.14308096177404
N	4.10702534468982	11.65626023348645	14.47152111258448
C	5.29877744204667	11.37926025114793	14.14367280627124
N	5.99180386779862	11.44543712126087	12.93403160467881
C	5.37763227433701	11.48681182797240	11.62568206596168
H	5.05672749553334	10.49302503929656	11.30168660633010
H	6.10179066371860	11.90947585606012	10.92111324220101
H	4.49992266800626	12.12579297019021	11.65490551185106
C	7.27848724293222	10.80059616707946	13.07374745926234
H	8.01921088098088	11.25167970073051	12.40795722799898

H	7.21891192856589	9.72317175151369	12.85593676389282
C	7.57907846305013	11.04256309693263	14.55028172233001
H	8.22261490581207	10.27413838044441	14.98796997000258
H	8.06753194733540	12.02400104029051	14.69397659969947
N	6.25287220526094	11.00776212375857	15.11134524300232
C	6.03855342671895	11.33437968874843	16.49077322547772
H	6.37546827026079	12.35554658030978	16.73916592331691
H	6.57509488399707	10.62974159567298	17.13333909069651
H	4.96941024373473	11.27031231530377	16.68481334378865
N	3.49603727816365	9.42384112850904	12.88739099982727
H	3.09544454194329	8.68772103677532	12.30441026234681

14.1.8 spiro-dmeN$_2$CN$_2$Hb$^-$

Illustration of the calculated minimum structure of spiro-dmeN$_2$CN$_2$Hb$^-$.

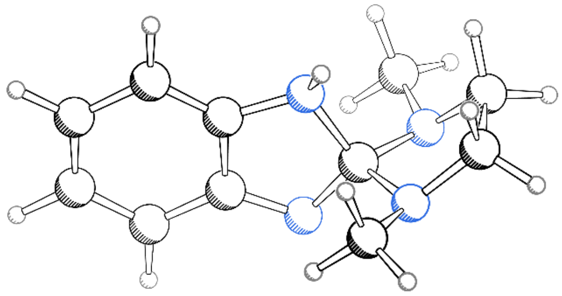

E_{el} = -647.10150541

Cartesian coordinates in Å:

C	3.13296250515528	12.37091187130726	13.49528137082510
C	3.04512639674830	10.96692492729964	13.22379316120453
C	1.87252559182622	10.34873457089861	12.86355790807790
C	0.69702679362193	11.12712436713022	12.75328626780952
C	0.75444808882138	12.48903809800712	13.02480024493101
C	1.94823258109510	13.12140441071969	13.39535763887281
H	1.84371110106021	9.27964835867396	12.67255276434173
H	-0.23757999481151	10.66036828416962	12.46511202042149
H	-0.15099306320831	13.08382447001702	12.94520586147297
H	1.97407850356071	14.18827801376528	13.58729582197257
N	4.38292387745119	12.76631615370805	13.80253503225378
C	5.23077354525025	11.64302023223426	13.70226754034250
N	6.29625204463107	11.74908249028568	12.67582909619632
C	5.85627493353455	11.84113439029782	11.31517766039451
H	5.51453811724068	10.87714367443635	10.88768734681911
H	6.66670011828918	12.21003347459395	10.67693891333562
H	5.02805629781610	12.54906325018508	11.27103943123441
C	7.31062739277869	10.76424933256023	12.97507251673271
H	8.31365111661794	11.14832140951857	12.75198610040341

H	7.18996018269407	9.83564870973250	12.38486383192164
C	7.10920077321896	10.47044613690098	14.47982554765666
H	6.78751969010073	9.42200525179440	14.62248785494399
H	8.02701362373593	10.60644331630808	15.06240984262353
N	6.08990923056356	11.41314262590475	14.88113086078591
C	5.38625476698589	11.09546773299826	16.09398712601866
H	6.06877429541210	11.16041348198379	16.94826040988401
H	4.93675932681113	10.08742973365500	16.08206235337476
H	4.58813197332920	11.82736024607114	16.22351484414969
N	4.32562291797604	10.45091737294506	13.44995423581891
H	4.66029546213087	9.75957897621734	12.79546738924285

14.1.9 TS(dmegbNH$^-$–spiro-dmeN$_2$CN$_2$Hb$^-$)

Illustration of the calculated transition structure between dmegbNH$^-$ and spiro-dmeN$_2$CN$_2$Hb$^-$.

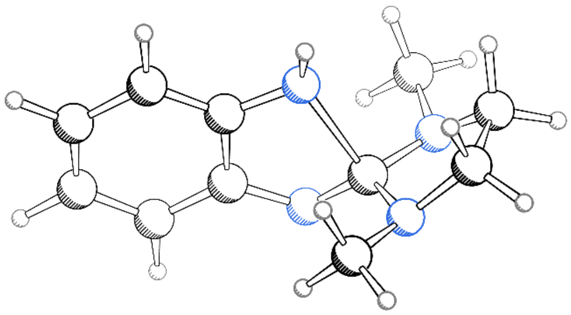

E_{el} = -647.08544895

Cartesian coordinates in Å:

C	-1.32537461224334	0.96353107964593	0.03174852269693
C	-1.44444192641563	-0.45776271592694	-0.20666610802843
C	-2.67064181383973	-0.95952743898322	-0.64842081986391
C	-3.77301741758437	-0.10866403718219	-0.85330685554077
C	-3.65573758981608	1.24978847274305	-0.61083567566193
C	-2.43084468426549	1.78398207857593	-0.16962871037672
H	-2.77482118467430	-2.02772412359743	-0.82155247161290
H	-4.71332537445459	-0.52486307809816	-1.20125712470626
H	-4.50365671991163	1.90915785184489	-0.76607128541544
H	-2.32451731768073	2.84985618302718	0.00341670622834
N	-0.09506890435488	1.37161880781808	0.46375986181189
C	0.89273436968482	0.48784911466069	0.36139099233560
N	1.79756815704854	0.45726032667026	-0.73568220359663
C	1.23775219556470	0.56743278342697	-2.05506543144259
H	0.75037178430520	-0.36579759045293	-2.37491671084990
H	2.02265108432345	0.82519190968224	-2.77299449393343
H	0.48790120102717	1.35699888565589	-2.04447504440392
C	2.74901937733448	-0.60483148589698	-0.49886706458919

H	3.74122164548198	-0.34457802143001	-0.88164347370251
H	2.43276278834738	-1.53776852248601	-0.99075632093157
C	2.72360571913942	-0.77310279212503	1.03752132834024
H	2.46656447295528	-1.80811739223272	1.30429376453254
H	3.68731799831919	-0.53685368046990	1.50254669749762
N	1.69210531115613	0.14608621521005	1.47090133061686
C	1.01478359707628	-0.14701831377630	2.70532676139834
H	1.71878210328618	-0.08142087373930	3.54140905696094
H	0.55575805998567	-1.14584546346677	2.68703525160639
H	0.22340094309608	0.59032934738843	2.83457859209931
N	-0.28928007681938	-1.11704711404425	0.07044324522040
H	-0.19357318607179	-2.01816041244146	-0.38223231668929

14.1.10 dmegHbNH

Illustration of the calculated minimum structure of dmegHbNH.

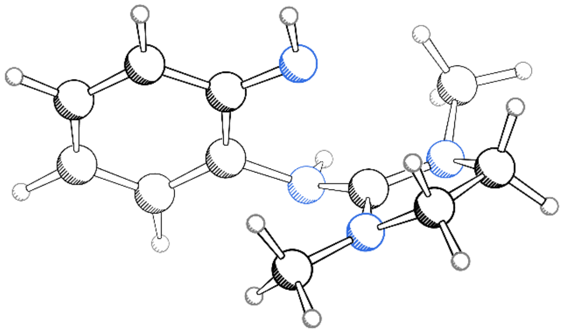

E_{el} = -647.65999061

Cartesian coordinates in Å:

C	3.11109389394339	12.06055379867531	13.55721551707675
C	2.97973321184347	10.65776487795276	13.29030592878112
C	1.77750336564119	10.31831000423704	12.59072045259010
C	0.85476536451670	11.26906739937127	12.20648163992059
C	1.03537923874760	12.63038792649949	12.48360068931257
C	2.18545426688004	13.00882008353354	13.16675134004572
H	1.60171246086439	9.27249318444051	12.36240923024048
H	-0.03519727563390	10.94926881811333	11.67529946561896
H	0.30371185927019	13.36536448951209	12.17901344684046
H	2.37226893503449	14.05053124882122	13.40355316929224
N	4.27370603780192	12.44744961566490	14.32843527868883
C	5.43663347706851	11.82700432078922	14.07890159504161
N	6.01167375443092	11.75528198194607	12.87306737212348
C	5.39447007458586	12.12340191752032	11.61719923151606
H	4.75371972439018	11.31847717164540	11.24840504705663
H	6.18582246548812	12.32609685199783	10.89479403280956
H	4.79376732281118	13.01814951818145	11.74718540895928
C	7.07465627486978	10.75815493205963	12.90576462245112
H	7.93540701483377	11.10203556282945	12.33215579175395

H	6.70671295047655	9.81793122554440	12.48655941897902
C	7.37085341934089	10.63591258038471	14.40998655116967
H	7.37062476648113	9.59469817551792	14.73195211306176
H	8.32294768020203	11.09118122527123	14.68929856294548
N	6.25441152526662	11.35221928397719	15.04674452364302
C	5.70484184689195	10.82503393660230	16.28665743202145
H	5.20686561900234	11.60905918298832	16.85734636193198
H	6.52751946753931	10.45825771770919	16.89771201751394
H	4.99479847908324	10.02177467970460	16.06232917675334
N	3.94887168278614	9.82839955745155	13.67755951193486
H	3.70578007522030	8.86948981107257	13.44940604488007
H	4.09358034597041	12.55671933019585	15.31682788415523

14.1.11 spiro-dme$N_2CN_2H_2$b

Illustration of the calculated minimum structure of spiro-dme$N_2CN_2H_2$b.

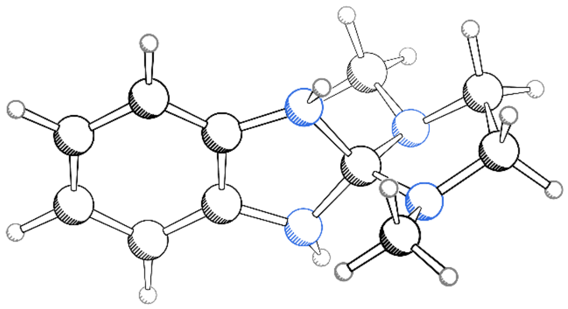

E_{el} = -647.68936308

Cartesian coordinates in Å:

C	3.03577204698739	12.33381643220373	13.52705968406292
C	2.96948194159269	10.97555315571007	13.17880209082608
C	1.76171258928928	10.37828985714689	12.87997181412579
C	0.60003743477107	11.15966151935462	12.94242149398966
C	0.66392573329998	12.49617329058534	13.30979050853151
C	1.89153625754890	13.10427541854825	13.60324371847578
H	1.71114893973381	9.32928338814717	12.61617900032944
H	-0.35617140784246	10.70875389888755	12.71170971149255
H	-0.24367147378152	13.08304374617692	13.36459539292673
H	1.94178540327562	14.15321244614653	13.86681940968794
N	4.37412042970589	12.67400296191630	13.68426673326575
C	5.23039638465605	11.50482456339707	13.61697735095236
N	6.33113005577211	11.66094557434362	12.69782021708057
C	6.03824527443110	11.73976832848501	11.29098765118562
H	5.81980417281946	10.76279405164555	10.82616493661269
H	6.89049203673573	12.17087406188987	10.76203027943474
H	5.17935448179991	12.39316402756558	11.13856523551151
C	7.45422138586753	10.84021701360291	13.12349225208042
H	8.38325769575052	11.41694485359877	13.09805398172934

H	7.59518808465251	9.96364440163322	12.47608352055603
C	7.06878210154706	10.40888417296347	14.54767665307272
H	6.78908933032349	9.34381884488658	14.56536133567095
H	7.87451248894066	10.55111785311218	15.26947218832936
N	5.93277517841331	11.26040137757917	14.86883759934813
C	5.11875902987926	10.79883451779272	15.97388375290457
H	4.27287614099989	11.47156060082087	16.11660892402795
H	5.71783471986279	10.80952665958013	16.88574089230669
H	4.72811242350349	9.78369077542736	15.81927371276010
N	4.25803108142482	10.43852979346197	13.25361878944383
H	4.54810472348958	9.79441330777023	12.53628041370071
H	4.67344464019806	13.40926951582898	14.30184961468454

14.1.12 TS(dmegHbNH–spiro-dme$N_2CN_2H_2$b)

Illustration of the calculated transition structure between dmegHbNH and spiro-dme$N_2CN_2H_2$b

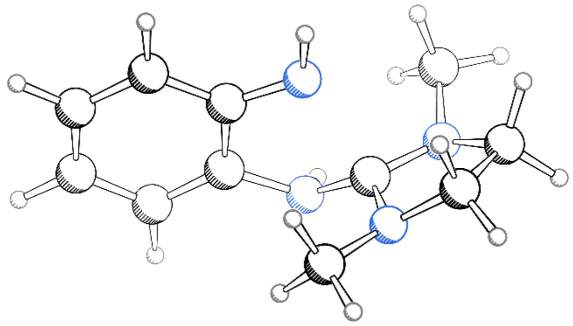

E_{el} = -647.65705920

Cartesian coordinates in Å:

C	-1.37720575907294	0.86014076478868	0.00006786814412
C	-1.47240009438999	-0.52964745759043	-0.28446849302381
C	-2.69456462029104	-0.95804594522683	-0.85385217762803
C	-3.71249225907683	-0.05150015092929	-1.11707523327246
C	-3.57893329022066	1.30668099690301	-0.83301296998247
C	-2.38318037960663	1.75978998795381	-0.27074665425437
H	-2.83124911038011	-2.00949469175521	-1.07908293206944
H	-4.63429977678553	-0.41420812806064	-1.55746330066168
H	-4.38091696789817	1.99944471613735	-1.04701538984756
H	-2.23873062174218	2.80949482641477	-0.04235886893458
N	-0.14477377236205	1.22629635373777	0.64617807371714
C	0.91984226965326	0.37655720210992	0.45484201036557
N	1.67668267656489	0.42751732811531	-0.67828156813728
C	1.09951210925751	0.70975993150272	-1.97236936838466
H	0.53518181642057	-0.15084715862012	-2.34721829281288
H	1.90205569737409	0.95452593371955	-2.66832323871972
H	0.42845400686189	1.56122260934390	-1.89345907357329
C	2.70721538858757	-0.59193996240168	-0.56558077902168

H	3.61438081227300	-0.27951908460028	-1.08208591939895
H	2.35804400952910	-1.53694908616432	-0.99870975118380
C	2.89019349247525	-0.70410864379322	0.95688933326027
H	2.91545578603042	-1.74625713129077	1.28113673757208
H	3.79895949762513	-0.21008155477702	1.30657600951404
N	1.70336736977360	-0.02892682805362	1.50142768402855
C	1.10328736091462	-0.59060786211575	2.69442843086525
H	0.32480420841489	0.06529022696088	3.08054290015074
H	1.87009018468053	-0.68617103820858	3.46285296047248
H	0.64988024110433	-1.56448474171126	2.48097423865767
N	-0.37875179863889	-1.23993786847885	0.02437578237751
H	-0.40839372855739	-2.21983714790337	-0.22261824483352
H	-0.26151474851823	1.45584360399357	1.62343022661478

14.1.13 TS(spiro-dmeN2CN2H2b–maemabi)

Illustration of the calculated transition structure between spiro-dmeN2CN2H2b and maemabi.

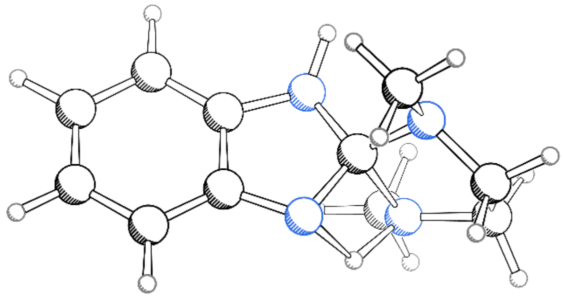

E_{el} = -647.63043993

Cartesian coordinates in Å:

C	-1.31006207026429	1.00003170686341	-0.11737434861793
C	-1.67615360048001	-0.34815558470323	-0.33636240801589
C	-2.98134376299156	-0.74094442100006	-0.52685993086250
C	-3.97421207432370	0.25027377559691	-0.50858634845912
C	-3.63411553397693	1.57892227409959	-0.28591464894700
C	-2.30505060143369	1.96963051580144	-0.09074007203526
H	-3.23916459584497	-1.78205413531865	-0.68027559274949
H	-5.00848426714006	-0.02697859895637	-0.66377900095397
H	-4.41272475450041	2.33152286996092	-0.26821871766762
H	-2.04815074753867	3.00872340502248	0.07059062337206
N	0.06021222304789	1.12057790771211	0.04338784384095
C	0.59328569699884	-0.21998552130202	-0.07525442524868
N	1.72536310860933	-0.49065154443762	-0.94195342480430
C	1.52400676982004	-0.02613510003105	-2.30723828407249
H	2.35979289019626	-0.35803294418949	-2.92431974846308
H	1.44102833485920	1.06605848558986	-2.36189888054093
H	0.60846670014224	-0.45801243679242	-2.71115159921180
C	2.92004296929994	0.04085182599074	-0.30260230622539

H	2.97682126529789	1.13646833421411	-0.38262320507671
H	3.81907736003064	-0.38405302998210	-0.74968468533792
C	2.74089165268956	-0.38150643279930	1.15040865284133
H	3.00509697577977	-1.43451427954669	1.28260752297077
H	3.31678704350677	0.22162324816240	1.85198034732139
N	1.30005809501841	-0.20453491831968	1.36006220825359
C	0.68233026966600	-1.03525690711359	2.38589826340067
H	-0.38217442831563	-0.81409114466179	2.43153673516807
H	1.13744694624736	-0.81403042918003	3.35179089719757
H	0.81264397102627	-2.09525961152148	2.15774089136783
N	-0.49136111647480	-1.11006670360889	-0.25845209037026
H	-0.34189121866934	-1.92810794025553	-0.82534805033930
H	0.78153649971767	0.92768733470602	1.23263378226541
